# Supplementary material for: Reliability of Computing van der Waals Bond Lengths of Some Rare Gas Diatomics
Source: Int J Mol Sci. 2022 Nov 11;23(22):13944. doi: 10.3390/ijms232213944 (PMC9696166; doi:10.3390/ijms232213944)
Supplement: Supplementary file 1 [file ijms-23-13944-s001.zip › ijms-2021140-supplementary.pdf]

SI

# Reliability of computing van der Waals bond lengths of some rare gas diatomics

Yi-Liang Zhang* and Bin Li

(*College of Chemistry, Jilin University, Qianjin Street 2699, Changchun 130012, China*)

(* *Corresponding author:* *yiliang@jlu.edu.cn*)

## Contents

Table S1. The numbers of 80 different basis sets......S2

Table S2. MADs (Å) of 11-RG-Mols calculated by 572 methods and 20 basis sets......S3

Table S3. MDs (Å) of 11-RG-Mols calculated by 572 methods and 20 basis sets......S15

Table S4. Imaginary frequencies ($cm^{-1}$) of 36 methods and 20 basis sets......S26

Table S5. MADs (Å) of 11-RG-Mols calculated by 54 methods and 60 basis sets......S31

Table S6. MDs (Å) of 11-RG-Mols calculated by 54 methods and 60 basis sets......S35

Reference......S38

Table S1. The numbers of 80 different basis sets.

| | | | | |
|---|---|---|---|---|
| BS01 | BS02 | BS03 | BS04 | BS05 |
| Def2TZVP | Def2TZVPP | Def2QZVP | Def2QZVPP | 6-311G** |
| BS06 | BS07 | BS08 | BS09 | BS10 |
| 6-311++G(d,p) | 6-311++G(2d,2p) | 6-311++G(3d,3p) | 6-311++G(df,pd) | 6-311++G(2df,2pd) |
| BS11 | BS12 | BS13 | BS14 | BS15 |
| 6-311++G(3df,3pd) | 6-311++G(3d2f,3p2d) | cc-pVDZ | aug-cc-pVDZ | cc-pVTZ |
| BS16 | BS17 | BS18 | BS19 | BS20 |
| aug-cc-pVTZ | cc-pVQZ | aug-cc-pVQZ | cc-pV5Z | aug-cc-pV5Z |
| | | | | |
| BS21 | BS22 | BS23 | BS24 | BS25 |
| SV | SVP | TZV | TZVP | Def2SV |
| BS26 | BS27 | BS28 | BS29 | BS30 |
| Def2SVP | Def2SVPP | Def2TZV | Def2QZV | DGDZVP |
| BS31 | BS32 | BS33 | BS34 | BS35 |
| CEP-31G | CEP-31++G | CEP-121G | CEP-121++G | SDD |
| BS36 | BS37 | BS38 | BS39 | BS40 |
| SDDAll | LanL2DZ | LanL2MB | STO-3G | STO-6G |
| BS41 | BS42 | BS43 | BS44 | BS45 |
| CBSB7 | CBSB7++ | 3-21G* | 6-31G | 6-311G |
| BS46 | BS47 | BS48 | BS49 | BS50 |
| 6-31+G | 6-311+G | 6-31++G | 6-311++G | 6-31G(d) |
| BS51 | BS52 | BS53 | BS54 | BS55 |
| 6-31G(d,p) | 6-31+G(d) | 6-31++G(d) | 6-31+G(d,p) | 6-31++G(d,p) |
| BS56 | BS57 | BS58 | BS59 | BS60 |
| 6-311+G(d) | 6-311++G(d) | 6-311+G(d,p) | 6-31G(df) | 6-31+G(df) |
| BS61 | BS62 | BS63 | BS64 | BS65 |
| 6-31++G(df) | 6-31+G(df,pd) | 6-311+G(df,pd) | 6-311++G(df) | 6-31++G(2d,2p) |
| BS66 | BS67 | BS68 | BS69 | BS70 |
| 6-31++G(3d,3p) | 6-31++G(df,pd) | 6-31++G(2df,2pd) | 6-31++G(3df,3pd) | 6-31++G(3d2f,3p2d) |
| BS71 | BS72 | BS73 | BS74 | BS75 |
| 6-31G(d',p') | 6-31++G(d',p') | 6-31++G(2d',2p') | 6-31++G(3d',3p') | 6-31++G(d'f,p'd) |
| BS76 | BS77 | BS78 | BS79 | BS80 |
| 6-31++G(2d'f,2p'd) | 6-31++G(3d'f,3p'd) | 6-31++G(3d'2f,3p'2d) | UGBS | UGBS1V++ |

Table S2. MADs (Å) of 11-RG-Mols calculated by 572 methods and 20 basis sets.[a,b,c,d,e,f]

| | BS01 | BS02 | BS03 | BS04 | BS05 | BS06 | BS07 | BS08 | BS09 | BS10 | BS11 | BS12 | BS13 | BS14 | BS15 | BS16 | BS17 | BS18 | BS19 | BS20 | Ave |
|---|---|---|---|---|---|---|---|---|---|---|---|---|---|---|---|---|---|---|---|---|---|
| **HF** | 0.791 | 0.758 | 1.309 | 1.309 | 0.626 | 0.914 | 0.804 | 0.726 | 0.913 | 0.804 | 0.726 | 0.855 | 0.396 | 0.918 | 0.665 | 1.105 | 1.013 | 1.259 | 1.308 | 1.308 | **0.925** |
| **APFD** | **0.094** | 0.100 | **0.012** | **0.012** | 0.178 | **0.005** | **0.045** | **0.054** | **0.006** | **0.047** | **0.056** | **0.012** | 0.271 | **0.025** | 0.127 | **0.023** | **0.053** | **0.012** | **0.012** | **0.013** | **0.058** |
| **wB97** | 0.155 | 0.162 | 0.432 | 0.434 | 0.224 | 0.240 | 0.185 | 0.178 | 0.240 | 0.218 | 0.206 | 0.254 | 0.372 | 0.311 | 0.232 | 0.254 | 0.312 | 0.458 | 0.392 | 0.474 | **0.287** |
| **wB97X** | 0.102 | 0.109 | **0.065** | **0.065** | 0.182 | **0.082** | **0.087** | **0.076** | **0.083** | **0.088** | **0.077** | **0.079** | 0.286 | 0.143 | 0.145 | **0.070** | **0.050** | **0.051** | **0.067** | **0.078** | **0.099** |
| **wB97XD** | 0.294 | 0.292 | 0.381 | 0.381 | 0.210 | 0.355 | 0.335 | 0.319 | 0.355 | 0.334 | 0.319 | 0.357 | 0.143 | 0.343 | 0.251 | 0.370 | 0.333 | 0.393 | 0.378 | 0.393 | **0.327** |
| **B98** | 0.139 | 0.144 | 0.145 | 0.145 | 0.227 | 0.136 | 0.127 | 0.119 | 0.135 | 0.127 | 0.120 | 0.138 | 0.262 | 0.135 | 0.176 | 0.141 | 0.136 | 0.147 | 0.144 | 0.143 | **0.149** |
| **B971** | 0.159 | 0.161 | 0.121 | 0.121 | 0.245 | 0.126 | 0.137 | 0.148 | 0.127 | 0.137 | 0.149 | 0.133 | 0.283 | 0.112 | 0.199 | 0.120 | 0.152 | 0.124 | 0.123 | 0.122 | **0.150** |
| **B972** | 0.367 | 0.359 | 0.495 | 0.495 | 0.308 | 0.471 | 0.431 | 0.397 | 0.471 | 0.429 | 0.396 | 0.484 | 0.235 | 0.456 | 0.310 | 0.507 | 0.401 | 0.514 | 0.494 | 0.506 | **0.426** |
| **BHandH** | 0.527 | 0.528 | 0.519 | 0.519 | 0.555 | 0.521 | 0.534 | 0.532 | 0.523 | 0.536 | 0.534 | 0.507 | 0.610 | 0.540 | 0.534 | 0.525 | 0.523 | 0.522 | 0.521 | 0.522 | **0.532** |
| **BHandHLYP** | 0.205 | 0.205 | 0.605 | 0.604 | 0.364 | 0.662 | 0.279 | 0.259 | 0.584 | 0.266 | 0.259 | 0.450 | 0.344 | 0.278 | 0.452 | 0.604 | 0.590 | 0.665 | 0.651 | 0.654 | **0.449** |
| **HFS** | 0.359 | 0.362 | 0.311 | 0.311 | 0.401 | 0.295 | 0.324 | 0.326 | 0.297 | 0.327 | 0.329 | 0.274 | 0.498 | 0.322 | 0.379 | 0.320 | 0.334 | 0.313 | 0.316 | 0.309 | **0.335** |
| **HFB** | 1.977 | 1.983 | 2.233 | 2.234 | 1.485 | 2.159 | 2.160 | 2.112 | 2.231 | 2.185 | 2.109 | 2.163 | 1.504 | 2.282 | 1.831 | 2.262 | 2.061 | 2.374 | 2.287 | 2.521 | **2.108** |
| **XAlpha** | 0.412 | 0.414 | 0.378 | 0.378 | 0.446 | 0.363 | 0.390 | 0.391 | 0.366 | 0.393 | 0.394 | 0.340 | 0.533 | 0.391 | 0.427 | 0.387 | 0.393 | 0.380 | 0.381 | 0.378 | **0.397** |
| **LSDA** | 0.504 | 0.506 | 0.488 | 0.488 | 0.526 | 0.473 | 0.497 | 0.497 | 0.477 | 0.500 | 0.501 | 0.449 | 0.595 | 0.502 | 0.514 | 0.496 | 0.496 | 0.491 | 0.491 | 0.491 | **0.499** |
| **LC-LSDA** | 0.166 | 0.174 | 0.396 | 0.396 | 0.259 | 0.354 | 0.294 | 0.266 | 0.355 | 0.294 | 0.265 | 0.355 | 0.274 | 0.355 | 0.237 | 0.325 | 0.267 | 0.408 | 0.394 | 0.419 | **0.312** |
| **HCTH** | 0.210 | 0.211 | 0.186 | 0.186 | 0.271 | 0.184 | 0.187 | 0.190 | 0.184 | 0.187 | 0.190 | 0.186 | 0.310 | 0.192 | 0.238 | 0.180 | 0.204 | 0.183 | 0.187 | 0.186 | **0.203** |
| **HCTH93** | 0.853 | 0.850 | 1.098 | 1.099 | 0.612 | 1.097 | 1.080 | 1.022 | 1.093 | 1.083 | 1.024 | 1.065 | 0.490 | 0.951 | 0.735 | 1.179 | 0.882 | 1.195 | 1.184 | 1.256 | **0.992** |
| **HCTH147** | 0.189 | 0.193 | 0.214 | 0.214 | 0.234 | 0.206 | 0.199 | 0.185 | 0.205 | 0.198 | 0.185 | 0.212 | 0.263 | 0.223 | 0.172 | 0.229 | 0.181 | 0.221 | 0.215 | 0.214 | **0.208** |
| **tHCTH** | 0.189 | 0.191 | 0.202 | 0.202 | 0.222 | 0.180 | 0.174 | 0.160 | 0.179 | 0.174 | 0.159 | 0.183 | 0.265 | 0.183 | 0.174 | 0.208 | 0.180 | 0.213 | 0.199 | 0.198 | **0.192** |
| **tHCTHhyb** | 0.212 | 0.213 | 0.307 | 0.307 | 0.225 | 0.284 | 0.253 | 0.239 | 0.283 | 0.251 | 0.238 | 0.300 | 0.223 | 0.291 | 0.183 | 0.315 | 0.228 | 0.309 | 0.296 | 0.300 | **0.263** |
| **VSXC** | 0.198 | 0.198 | 0.160 | 0.160 | 0.265 | 0.163 | 0.199 | 0.200 | 0.163 | 0.200 | 0.200 | 0.161 | 0.333 | 0.209 | 0.228 | 0.185 | 0.159 | 0.179 | 0.162 | 0.185 | **0.195** |
| **BMK** | 1.669 | 1.667 | 1.810 | 1.881 | 1.411 | 1.494 | 1.497 | 1.419 | 1.515 | 1.458 | 1.447 | 1.536 | 1.097 | 1.682 | 1.676 | 1.605 | 1.746 | 1.719 | 1.866 | 1.969 | **1.608** |
| **TPSSh** | 0.232 | 0.233 | 0.280 | 0.280 | 0.277 | 0.264 | 0.248 | 0.235 | 0.262 | 0.247 | 0.235 | 0.271 | 0.267 | 0.274 | 0.233 | 0.299 | 0.248 | 0.312 | 0.277 | 0.277 | **0.263** |
| **APF** | 0.507 | 0.470 | 0.835 | 0.835 | 0.481 | 0.797 | 0.699 | 0.606 | 0.799 | 0.725 | 0.602 | 0.800 | 0.389 | 0.738 | 0.442 | 0.907 | 0.702 | 0.908 | 0.846 | 0.898 | **0.699** |
| **OAPF** | 0.169 | 0.175 | 0.243 | 0.245 | 0.277 | 0.236 | 0.201 | 0.185 | 0.235 | 0.199 | 0.183 | 0.247 | 0.283 | 0.242 | 0.213 | 0.254 | 0.205 | 0.247 | 0.241 | 0.244 | **0.226** |
| **GVB(0)** | 0.768 | 0.768 | 1.287 | 1.287 | 0.632 | 0.923 | 0.792 | 0.718 | 0.923 | 0.792 | 0.717 | 0.865 | 0.386 | 0.897 | 0.664 | 1.090 | 1.016 | 1.247 | 1.285 | 1.276 | **0.917** |
| **B97D** | **0.064** | **0.066** | 0.147 | 0.147 | 0.139 | 0.163 | 0.139 | 0.132 | 0.162 | 0.138 | 0.131 | 0.173 | 0.168 | 0.158 | **0.088** | 0.174 | **0.078** | 0.159 | 0.142 | 0.137 | **0.135** |
| **B97D3** | 0.114 | 0.116 | 0.199 | 0.200 | 0.121 | 0.209 | 0.172 | 0.162 | 0.208 | 0.172 | 0.161 | 0.226 | 0.148 | 0.210 | **0.082** | 0.224 | 0.138 | 0.213 | 0.196 | 0.198 | **0.173** |
| **B3LYP** | 0.982 | 1.057 | 1.864 | 1.910 | 1.099 | 1.416 | 1.190 | 1.069 | 1.380 | 1.195 | 1.074 | 1.349 | 0.636 | 1.142 | 1.099 | 1.772 | 1.174 | 1.910 | 1.828 | 1.990 | **1.357** |
| **B3P86** | 1.725 | 1.698 | 2.019 | 2.020 | 1.307 | 1.734 | 1.661 | 1.580 | 1.697 | 1.656 | 1.581 | 1.737 | 1.363 | 1.767 | 1.721 | 1.861 | 1.807 | 2.106 | 2.034 | 2.151 | **1.761** |
| **B3PW91** | 1.676 | 1.654 | 2.031 | 2.010 | 1.227 | 1.650 | 1.611 | 1.530 | 1.627 | 1.611 | 1.536 | 1.651 | 1.359 | 1.588 | 1.587 | 1.826 | 1.749 | 2.059 | 1.966 | 2.077 | **1.701** |
| **X3LYP** | 0.203 | 0.207 | 0.218 | 0.218 | 0.338 | 0.224 | 0.203 | 0.215 | 0.231 | 0.202 | 0.215 | 0.257 | 0.349 | 0.209 | 0.279 | 0.225 | 0.217 | 0.213 | 0.219 | 0.219 | **0.233** |
| **O3LYP** | 0.290 | 0.290 | 0.400 | 0.400 | 0.332 | 0.385 | 0.369 | 0.352 | 0.385 | 0.371 | 0.351 | 0.387 | 0.315 | 0.350 | 0.289 | 0.455 | 0.332 | 0.421 | 0.409 | 0.411 | **0.365** |
| **B1LYP** | 1.149 | 1.000 | 1.803 | 1.828 | 1.087 | 1.444 | 1.186 | 1.145 | 1.472 | 1.211 | 1.159 | 1.439 | 0.779 | 1.255 | 1.170 | 1.724 | 1.287 | 2.103 | 1.741 | 2.047 | **1.402** |
| **B1B95** | 0.212 | 0.212 | 0.278 | 0.278 | 0.213 | 0.410 | 0.367 | 0.302 | 0.410 | 0.367 | 0.302 | 0.431 | 0.184 | 0.230 | 0.180 | 0.444 | 0.252 | 0.280 | 0.245 | 0.315 | **0.296** |
| **OmPW1LYP** | 0.258 | 0.259 | 0.235 | 0.235 | 0.341 | 0.248 | 0.251 | 0.252 | 0.248 | 0.252 | 0.253 | 0.253 | 0.377 | 0.246 | 0.298 | 0.235 | 0.257 | 0.234 | 0.237 | 0.234 | **0.260** |
| **LG1LYP** | 0.321 | 0.322 | 0.293 | 0.293 | 0.370 | 0.293 | 0.303 | 0.304 | 0.294 | 0.305 | 0.306 | 0.292 | 0.444 | 0.296 | 0.340 | 0.293 | 0.306 | 0.297 | 0.295 | 0.295 | **0.313** |
| **mPW1LYP** | 0.253 | 0.254 | 0.227 | 0.226 | 0.339 | 0.242 | 0.245 | 0.246 | 0.242 | 0.245 | 0.247 | 0.247 | 0.374 | 0.237 | 0.294 | 0.228 | 0.250 | 0.226 | 0.229 | 0.226 | **0.254** |
| **mPW1PW91** | 0.376 | 0.369 | 0.511 | 0.511 | 0.341 | 0.505 | 0.461 | 0.405 | 0.504 | 0.459 | 0.404 | 0.501 | 0.282 | 0.440 | 0.324 | 0.540 | 0.420 | 0.532 | 0.512 | 0.523 | **0.446** |
| **mPW1PBE** | 0.385 | 0.377 | 0.519 | 0.519 | 0.342 | 0.512 | 0.468 | 0.425 | 0.509 | 0.467 | 0.427 | 0.511 | 0.283 | 0.450 | 0.329 | 0.550 | 0.431 | 0.542 | 0.517 | 0.533 | **0.455** |
| **mPW3PBE** | 0.310 | 0.310 | 0.461 | 0.461 | 0.325 | 0.439 | 0.391 | 0.363 | 0.439 | 0.390 | 0.362 | 0.444 | 0.270 | 0.398 | 0.286 | 0.475 | 0.353 | 0.486 | 0.458 | 0.477 | **0.395** |
| **PBE1PBE** | 0.148 | 0.152 | 0.155 | 0.155 | 0.236 | 0.148 | 0.130 | 0.122 | 0.142 | 0.129 | 0.121 | 0.160 | 0.271 | 0.145 | 0.178 | 0.147 | 0.144 | 0.154 | 0.153 | 0.153 | **0.157** |
| **PBEh1PBE** | 0.146 | 0.151 | 0.151 | 0.151 | 0.236 | 0.140 | 0.129 | 0.123 | 0.139 | 0.128 | 0.121 | 0.149 | 0.272 | 0.146 | 0.184 | 0.146 | 0.142 | 0.152 | 0.150 | 0.150 | **0.155** |
| **HSEh1PBE** | 0.145 | 0.147 | 0.144 | 0.144 | 0.237 | 0.136 | 0.128 | 0.123 | 0.136 | 0.128 | 0.123 | 0.146 | 0.273 | 0.137 | 0.185 | 0.141 | 0.139 | 0.145 | 0.141 | 0.143 | **0.152** |
| **OHSE1PBE** | 0.144 | 0.148 | 0.142 | 0.142 | 0.237 | 0.136 | 0.128 | 0.122 | 0.136 | 0.128 | 0.122 | 0.144 | 0.273 | 0.138 | 0.185 | 0.141 | 0.139 | 0.145 | 0.138 | 0.141 | **0.151** |
| **OHSE2PBE** | 0.139 | 0.142 | 0.139 | 0.139 | 0.238 | 0.135 | 0.131 | 0.128 | 0.134 | 0.130 | 0.128 | 0.144 | 0.278 | 0.126 | 0.187 | 0.135 | 0.137 | 0.140 | 0.139 | 0.139 | **0.150** |
| **HISSbPBE** | 0.124 | 0.126 | 0.138 | 0.138 | 0.213 | 0.128 | 0.123 | 0.107 | 0.128 | 0.121 | 0.106 | 0.136 | 0.255 | 0.121 | 0.161 | 0.125 | 0.123 | 0.135 | 0.137 | 0.135 | **0.139** |
| **M06** | **0.065** | **0.065** | 0.141 | 0.141 | **0.064** | **0.072** | **0.072** | **0.070** | **0.072** | **0.072** | **0.070** | **0.074** | **0.074** | **0.054** | **0.051** | **0.068** | 0.165 | 0.170 | 0.140 | 0.152 | **0.093** |
| **M06L** | 0.121 | 0.120 | 0.140 | 0.140 | 0.145 | 0.127 | 0.100 | **0.074** | 0.127 | **0.099** | **0.073** | 0.127 | 0.113 | **0.082** | 0.111 | **0.066** | 0.134 | **0.099** | 0.110 | **0.076** | **0.109** |
| **M06HF** | 0.137 | 0.138 | 0.142 | 0.142 | 0.167 | 0.144 | 0.144 | 0.147 | 0.144 | 0.144 | 0.147 | 0.144 | **0.055** | 0.112 | 0.146 | 0.143 | 0.143 | 0.145 | 0.141 | 0.112 | **0.137** |
| **M062X** | 0.127 | 0.128 | 0.146 | 0.146 | 0.245 | 0.144 | 0.134 | 0.139 | 0.144 | 0.134 | 0.139 | 0.146 | 0.256 | **0.042** | 0.170 | 0.126 | 0.155 | 0.145 | 0.145 | 0.171 | **0.149** |
| **M05** | 0.220 | 0.222 | 0.197 | 0.197 | 0.307 | 0.224 | 0.224 | 0.222 | 0.225 | 0.224 | 0.223 | 0.224 | 0.387 | 0.247 | 0.257 | 0.195 | 0.208 | 0.193 | 0.197 | 0.190 | **0.229** |
| **M052X** | 0.160 | 0.160 | 0.151 | 0.151 | 0.247 | 0.176 | 0.180 | 0.182 | 0.177 | 0.180 | 0.182 | 0.178 | 0.288 | 0.146 | 0.208 | 0.162 | 0.155 | 0.147 | 0.149 | 0.147 | **0.176** |

| | | | | | | | | | | | | | | | | | | | | | |
|---|---|---|---|---|---|---|---|---|---|---|---|---|---|---|---|---|---|---|---|---|---|
| PW6B95 | 0.108 | 0.108 | 0.106 | 0.106 | 0.195 | 0.100 | 0.099 | 0.086 | 0.100 | 0.099 | 0.086 | 0.120 | 0.177 | 0.112 | 0.108 | 0.101 | 0.093 | 0.106 | 0.107 | 0.107 | 0.111 |
| PW6B95D3 | 0.081 | 0.081 | 0.089 | 0.089 | 0.180 | 0.082 | 0.082 | 0.069 | 0.082 | 0.082 | 0.069 | 0.081 | 0.182 | 0.097 | 0.092 | 0.084 | 0.075 | 0.088 | 0.089 | 0.090 | 0.093 |
| M08HX | 0.116 | 0.136 | 0.129 | 0.129 | 0.229 | 0.144 | 0.150 | 0.150 | 0.145 | 0.151 | 0.151 | 0.137 | 0.332 | 0.224 | 0.172 | 0.152 | 0.092 | 0.122 | 0.133 | 0.164 | 0.158 |
| M11 | 0.116 | 0.115 | 0.159 | 0.159 | 0.210 | 0.135 | 0.132 | 0.127 | 0.134 | 0.131 | 0.127 | 0.136 | 0.238 | 0.098 | 0.149 | 0.102 | 0.147 | 0.151 | 0.158 | 0.163 | 0.144 |
| M11L | 0.766 | 0.798 | 1.000 | 1.000 | 0.661 | 0.837 | 0.820 | 0.636 | 0.836 | 0.821 | 0.636 | 0.836 | 0.511 | 0.685 | 0.650 | 0.866 | 0.902 | 1.053 | 0.984 | 1.082 | 0.819 |
| SOGGA11 | 0.385 | 0.385 | 0.341 | 0.341 | 0.376 | 0.335 | 0.338 | 0.343 | 0.336 | 0.339 | 0.343 | 0.333 | 0.733 | 0.339 | 0.357 | 0.343 | 0.356 | 0.374 | 0.328 | 0.348 | 0.369 |
| SOGGA11X | 0.879 | 0.868 | 1.053 | 1.052 | 0.738 | 0.938 | 0.911 | 0.909 | 0.929 | 0.911 | 0.909 | 0.953 | 0.357 | 0.763 | 0.786 | 1.024 | 0.927 | 1.018 | 1.029 | 1.051 | 0.900 |
| N12 | 0.945 | 0.910 | 1.556 | 1.563 | 0.793 | 1.297 | 1.285 | 1.233 | 1.344 | 1.320 | 1.206 | 1.357 | 0.632 | 1.288 | 0.811 | 1.474 | 1.072 | 1.596 | 1.487 | 1.614 | 1.239 |
| N12SX | 0.629 | 0.632 | 1.548 | 1.563 | 0.932 | 1.282 | 0.962 | 0.949 | 1.287 | 0.971 | 0.994 | 1.288 | 0.350 | 0.829 | 0.817 | 1.230 | 0.998 | 1.621 | 1.574 | 1.733 | 1.109 |
| MN12SX | 0.288 | 0.288 | 0.798 | 0.798 | 0.278 | 0.456 | 0.420 | 0.374 | 0.457 | 0.419 | 0.354 | 0.460 | 0.159 | 0.367 | 0.218 | 0.337 | 0.617 | 0.915 | 0.764 | 0.893 | 0.483 |
| MN12L | 0.792 | 0.731 | 1.051 | 1.051 | 0.617 | 0.844 | 0.815 | 0.769 | 0.845 | 0.815 | 0.769 | 0.813 | 0.156 | 0.501 | 0.609 | 0.816 | 0.874 | 1.157 | 0.955 | 1.259 | 0.812 |
| MN15 | 0.094 | 0.093 | 0.081 | 0.081 | 0.130 | 0.075 | 0.078 | 0.066 | 0.075 | 0.078 | 0.066 | 0.080 | 0.197 | 0.097 | 0.129 | 0.066 | 0.080 | 0.072 | 0.082 | 0.088 | 0.090 |
| MN15L | 0.072 | 0.063 | 0.086 | 0.086 | 0.111 | 0.076 | 0.076 | 0.070 | 0.076 | 0.076 | 0.062 | 0.087 | 0.122 | 0.107 | 0.110 | 0.061 | 0.079 | 0.081 | 0.086 | 0.089 | 0.084 |
| LC-HFS | 0.468 | 0.457 | 1.062 | 1.062 | 0.453 | 1.002 | 0.941 | 0.861 | 1.001 | 0.940 | 0.852 | 1.008 | 0.285 | 0.910 | 0.454 | 1.058 | 0.722 | 1.184 | 1.080 | 1.200 | 0.850 |
| LC-HFB | 0.470 | 0.458 | 1.073 | 1.073 | 0.453 | 1.017 | 0.946 | 0.850 | 1.016 | 0.940 | 0.860 | 0.987 | 0.286 | 0.952 | 0.456 | 1.125 | 0.725 | 1.161 | 1.070 | 1.281 | 0.860 |
| LC-XAlpha | 0.472 | 0.460 | 1.047 | 1.047 | 0.442 | 0.981 | 0.923 | 0.814 | 1.004 | 0.929 | 0.813 | 0.945 | 0.284 | 0.916 | 0.457 | 1.094 | 0.714 | 1.093 | 1.048 | 1.200 | 0.834 |
| LC-M06L | 0.722 | 0.723 | 0.712 | 0.712 | 0.734 | 0.712 | 0.722 | 0.723 | 0.713 | 0.723 | 0.724 | 0.703 | 0.688 | 0.712 | 0.721 | 0.718 | 0.718 | 0.719 | 0.716 | 0.718 | 0.717 |
| LC-M11L | 0.766 | 0.798 | 1.000 | 1.001 | 0.661 | 0.837 | 0.820 | 0.636 | 0.836 | 0.821 | 0.636 | 0.836 | 0.511 | 0.685 | 0.650 | 0.866 | 0.902 | 1.053 | 0.984 | 1.084 | 0.819 |
| LC-N12 | 1.735 | 1.735 | 2.143 | 2.145 | 1.811 | 1.828 | 1.812 | 1.787 | 1.829 | 1.806 | 1.785 | 1.829 | 1.343 | 1.779 | 1.827 | 1.792 | 2.001 | 2.170 | 2.071 | 2.139 | 1.868 |
| LC-B97D | 0.658 | 0.658 | 0.651 | 0.651 | 0.672 | 0.648 | 0.652 | 0.651 | 0.648 | 0.653 | 0.652 | 0.641 | 0.705 | 0.648 | 0.659 | 0.651 | 0.653 | 0.651 | 0.651 | 0.651 | 0.655 |
| LC-B97D3 | 0.705 | 0.706 | 0.698 | 0.698 | 0.717 | 0.693 | 0.698 | 0.698 | 0.694 | 0.699 | 0.699 | 0.685 | 0.750 | 0.695 | 0.705 | 0.698 | 0.700 | 0.699 | 0.699 | 0.698 | 0.702 |
| LC-wPBE | 0.248 | 0.244 | 0.631 | 0.629 | 0.309 | 0.581 | 0.524 | 0.510 | 0.581 | 0.519 | 0.510 | 0.572 | 0.215 | 0.531 | 0.266 | 0.537 | 0.406 | 0.679 | 0.639 | 0.723 | 0.493 |
| LC-wHPBE | 0.244 | 0.243 | 0.671 | 0.669 | 0.309 | 0.576 | 0.521 | 0.461 | 0.576 | 0.521 | 0.463 | 0.553 | 0.216 | 0.559 | 0.263 | 0.555 | 0.405 | 0.675 | 0.631 | 0.718 | 0.492 |
| CAM-B3LYP | 0.214 | 0.216 | 0.170 | 0.170 | 0.316 | 0.201 | 0.192 | 0.196 | 0.201 | 0.192 | 0.196 | 0.205 | 0.363 | 0.169 | 0.264 | 0.170 | 0.209 | 0.171 | 0.178 | 0.170 | 0.208 |
| LC-HCTH | 0.558 | 0.558 | 0.541 | 0.541 | 0.579 | 0.538 | 0.541 | 0.540 | 0.538 | 0.542 | 0.541 | 0.532 | 0.623 | 0.534 | 0.559 | 0.537 | 0.546 | 0.540 | 0.540 | 0.539 | 0.548 |
| LC-tHCTH | 0.736 | 0.737 | 0.730 | 0.730 | 0.745 | 0.723 | 0.728 | 0.728 | 0.724 | 0.729 | 0.729 | 0.716 | 0.777 | 0.726 | 0.735 | 0.729 | 0.731 | 0.730 | 0.730 | 0.729 | 0.732 |
| LC-XaVP86 | 0.180 | 0.182 | 0.478 | 0.479 | 0.244 | 0.207 | 0.164 | 0.213 | 0.206 | 0.165 | 0.218 | 0.199 | 0.372 | 0.328 | 0.303 | 0.220 | 0.387 | 0.398 | 0.476 | 0.530 | 0.297 |
| LC-XaVWN | 0.163 | 0.173 | 0.380 | 0.381 | 0.256 | 0.338 | 0.278 | 0.251 | 0.337 | 0.277 | 0.251 | 0.340 | 0.274 | 0.346 | 0.234 | 0.311 | 0.262 | 0.389 | 0.380 | 0.404 | 0.301 |
| LC-XaVWN5 | 0.206 | 0.208 | 0.496 | 0.496 | 0.290 | 0.468 | 0.384 | 0.340 | 0.466 | 0.384 | 0.336 | 0.467 | 0.272 | 0.447 | 0.265 | 0.443 | 0.334 | 0.519 | 0.501 | 0.536 | 0.393 |
| BB95 | 0.551 | 0.724 | 0.636 | 0.636 | 0.499 | 0.693 | 1.040 | 0.634 | 0.705 | 0.598 | 0.650 | 0.593 | 0.335 | 0.464 | 0.658 | 1.383 | 0.839 | 0.874 | 0.657 | 0.646 | 0.691 |
| BBRC | 1.819 | 1.815 | 2.206 | 2.206 | 1.368 | 1.949 | 1.951 | 1.964 | 1.950 | 1.952 | 1.954 | 1.976 | 1.460 | 2.034 | 1.833 | 2.234 | 1.885 | 2.232 | 2.216 | 2.336 | 1.967 |
| BKCIS | 1.829 | 1.827 | 2.208 | 2.208 | 1.399 | 1.905 | 1.903 | 1.897 | 1.908 | 1.901 | 1.896 | 1.944 | 1.397 | 1.964 | 1.814 | 2.204 | 1.917 | 2.232 | 2.205 | 2.288 | 1.942 |
| BLYP | 1.667 | 1.673 | 2.216 | 2.221 | 1.231 | 1.885 | 1.859 | 1.779 | 1.835 | 1.863 | 1.816 | 1.895 | 1.120 | 1.701 | 1.136 | 2.140 | 1.380 | 2.222 | 2.216 | 2.320 | 1.809 |
| BP86 | 1.891 | 1.891 | 2.219 | 2.219 | 1.692 | 1.977 | 2.007 | 2.001 | 1.999 | 2.007 | 2.005 | 1.978 | 1.425 | 2.099 | 1.891 | 2.164 | 1.936 | 2.248 | 2.227 | 2.390 | 2.013 |
| BPBE | 1.801 | 1.822 | 2.169 | 2.164 | 1.398 | 1.906 | 1.873 | 1.822 | 1.875 | 1.872 | 1.867 | 1.938 | 1.519 | 1.962 | 1.845 | 2.143 | 1.869 | 2.232 | 2.165 | 2.341 | 1.929 |
| BPKZB | 1.810 | 1.809 | 2.136 | 2.135 | 1.408 | 1.929 | 1.794 | 1.784 | 1.916 | 1.867 | 1.750 | 1.916 | 1.463 | 1.979 | 1.798 | 2.111 | 1.877 | 2.230 | 2.123 | 2.333 | 1.908 |
| BPL | 1.818 | 1.811 | 2.125 | 2.121 | 1.365 | 1.902 | 1.896 | 1.826 | 1.905 | 1.860 | 1.786 | 1.934 | 1.423 | 1.802 | 1.757 | 2.105 | 1.866 | 2.227 | 2.114 | 2.205 | 1.892 |
| BPW91 | 1.822 | 1.821 | 2.149 | 2.139 | 1.467 | 1.946 | 1.867 | 1.774 | 1.943 | 1.871 | 1.759 | 1.946 | 1.461 | 1.985 | 1.794 | 2.112 | 1.920 | 2.223 | 2.109 | 2.301 | 1.921 |
| BRevTPSS | 1.815 | 1.794 | 2.190 | 2.201 | 1.442 | 1.918 | 1.883 | 1.801 | 1.924 | 1.915 | 1.768 | 1.918 | 1.490 | 2.002 | 1.770 | 2.133 | 1.888 | 2.239 | 2.238 | 2.256 | 1.929 |
| BTPSS | 1.814 | 1.831 | 2.170 | 2.166 | 1.455 | 1.915 | 1.942 | 1.754 | 1.916 | 1.914 | 1.863 | 1.915 | 1.428 | 1.937 | 1.803 | 2.165 | 1.875 | 2.224 | 2.141 | 2.333 | 1.928 |
| BVP86 | 1.882 | 1.883 | 2.217 | 2.220 | 1.684 | 1.997 | 2.007 | 1.995 | 1.975 | 2.011 | 1.971 | 1.997 | 1.431 | 2.110 | 1.910 | 2.165 | 1.982 | 2.287 | 2.222 | 2.389 | 2.017 |
| BVWN | 1.856 | 1.859 | 2.106 | 2.117 | 1.269 | 1.758 | 1.710 | 1.658 | 1.757 | 1.710 | 1.663 | 1.765 | 1.481 | 1.704 | 1.764 | 1.971 | 1.831 | 2.179 | 1.986 | 2.304 | 1.822 |
| BVWN5 | 1.814 | 1.813 | 2.116 | 2.115 | 1.362 | 1.896 | 1.861 | 1.814 | 1.937 | 1.897 | 1.786 | 1.939 | 1.468 | 1.794 | 1.770 | 2.112 | 1.867 | 2.221 | 2.111 | 2.211 | 1.895 |
| BRxB95 | 0.146 | 0.146 | 0.174 | 0.174 | 0.120 | 0.203 | 0.146 | 0.144 | 0.203 | 0.146 | 0.144 | 0.205 | 0.110 | 0.176 | 0.143 | 0.178 | 0.146 | 0.150 | 0.174 | 0.150 | 0.159 |
| BRxBRC | 1.242 | 1.172 | 1.721 | 1.717 | 0.939 | 1.376 | 1.370 | 1.319 | 1.373 | 1.364 | 1.291 | 1.415 | 0.873 | 1.305 | 1.034 | 1.691 | 1.194 | 1.737 | 1.661 | 1.890 | 1.384 |
| BRxKCIS | 1.233 | 1.231 | 1.603 | 1.603 | 0.939 | 1.382 | 1.346 | 1.204 | 1.383 | 1.328 | 1.192 | 1.386 | 0.850 | 1.245 | 0.984 | 1.664 | 1.194 | 1.745 | 1.588 | 1.745 | 1.342 |
| BRxLYP | 0.223 | 0.228 | 0.170 | 0.170 | 0.325 | 0.190 | 0.153 | 0.162 | 0.182 | 0.153 | 0.162 | 0.220 | 0.359 | 0.228 | 0.268 | 0.176 | 0.214 | 0.173 | 0.175 | 0.167 | 0.205 |
| BRxP86 | 1.473 | 1.439 | 2.093 | 2.097 | 1.138 | 1.630 | 1.437 | 1.366 | 1.631 | 1.437 | 1.393 | 1.704 | 1.034 | 1.587 | 0.998 | 1.770 | 1.684 | 2.079 | 2.045 | 2.133 | 1.608 |
| BRxPBE | 1.531 | 1.522 | 1.753 | 1.754 | 0.946 | 1.484 | 1.403 | 1.387 | 1.435 | 1.372 | 1.436 | 1.443 | 0.931 | 1.416 | 1.125 | 1.796 | 1.433 | 1.891 | 1.822 | 1.964 | 1.492 |
| BRxPKZB | 1.392 | 1.445 | 1.792 | 1.792 | 0.990 | 1.466 | 1.365 | 1.380 | 1.438 | 1.454 | 1.406 | 1.487 | 0.888 | 1.419 | 1.152 | 1.698 | 1.476 | 1.930 | 1.849 | 1.935 | 1.488 |
| BRxPL | 1.287 | 1.248 | 1.615 | 1.615 | 0.922 | 1.353 | 1.360 | 1.241 | 1.352 | 1.362 | 1.246 | 1.372 | 0.832 | 1.213 | 1.121 | 1.612 | 1.260 | 1.707 | 1.607 | 1.733 | 1.353 |
| BRxPW91 | 1.476 | 1.442 | 1.820 | 1.820 | 0.987 | 1.513 | 1.414 | 1.364 | 1.479 | 1.402 | 1.374 | 1.529 | 0.883 | 1.443 | 1.246 | 1.652 | 1.412 | 1.799 | 1.786 | 1.988 | 1.492 |
| BRxRevTPSS | 1.386 | 1.371 | 1.935 | 1.899 | 0.993 | 1.480 | 1.476 | 1.395 | 1.447 | 1.496 | 1.392 | 1.485 | 0.866 | 1.377 | 1.160 | 1.658 | 1.335 | 1.882 | 1.741 | 1.920 | 1.485 |
| BRxTPSS | 1.446 | 1.445 | 1.830 | 1.829 | 1.035 | 1.472 | 1.454 | 1.409 | 1.469 | 1.408 | 1.409 | 1.468 | 0.883 | 1.406 | 1.165 | 1.734 | 1.468 | 1.886 | 1.823 | 1.940 | 1.499 |
| BRxVP86 | 1.470 | 1.470 | 2.048 | 2.095 | 1.246 | 1.625 | 1.414 | 1.398 | 1.656 | 1.450 | 1.379 | 1.656 | 0.997 | 1.619 | 0.973 | 1.737 | 1.692 | 2.048 | 2.100 | 2.134 | 1.610 |
| BRxVWN | 1.198 | 1.186 | 1.549 | 1.544 | 0.879 | 1.303 | 1.217 | 1.140 | 1.304 | 1.251 | 1.139 | 1.305 | 0.794 | 1.120 | 1.033 | 1.434 | 1.167 | 1.616 | 1.462 | 1.633 | 1.264 |
| BRxVWN5 | 1.278 | 1.243 | 1.616 | 1.619 | 0.922 | 1.371 | 1.362 | 1.242 | 1.353 | 1.357 | 1.240 | 1.368 | 0.804 | 1.214 | 1.113 | 1.609 | 1.262 | 1.710 | 1.624 | 1.700 | 1.350 |

| | | | | | | | | | | | | | | | | | | | | | |
|---|---|---|---|---|---|---|---|---|---|---|---|---|---|---|---|---|---|---|---|---|---|
| **G96B95** | 2.807 | 2.845 | 3.724 | 3.666 | 2.943 | 3.587 | 3.567 | 3.850 | 3.514 | 3.279 | 3.543 | 3.589 | 2.391 | 4.074 | 2.856 | 3.707 | 3.135 | 3.783 | 3.731 | 3.889 | **3.424** |
| **G96BRC** | 2.822 | 2.825 | 3.376 | 3.383 | 2.587 | 3.230 | 3.340 | 3.334 | 3.332 | 3.334 | 3.329 | 3.282 | 2.176 | 3.658 | 2.687 | 3.416 | 3.035 | 3.635 | 3.267 | 3.588 | **3.182** |
| **G96KCIS** | 2.831 | 2.838 | 3.369 | 3.369 | 2.797 | 3.349 | 3.244 | 3.241 | 3.349 | 3.244 | 3.240 | 3.341 | 2.165 | 3.684 | 2.802 | 3.382 | 3.022 | 3.511 | 3.353 | 3.509 | **3.182** |
| **G96LYP** | 2.812 | 2.812 | 3.437 | 3.443 | 2.660 | 3.376 | 3.376 | 3.319 | 3.376 | 3.369 | 3.318 | 3.369 | 2.158 | 3.988 | 2.702 | 3.428 | 3.040 | 3.527 | 3.426 | 3.487 | **3.221** |
| **G96P86** | 2.764 | 2.766 | 3.348 | 3.357 | 2.681 | 3.046 | 3.048 | 3.045 | 3.060 | 3.054 | 3.022 | 3.053 | 2.176 | 3.606 | 2.639 | 3.215 | 3.017 | 3.394 | 3.251 | 3.397 | **3.047** |
| **G96PBE** | 2.766 | 2.769 | 3.369 | 3.362 | 2.579 | 3.117 | 3.115 | 3.118 | 3.116 | 3.119 | 3.120 | 3.114 | 2.148 | 3.456 | 2.688 | 3.372 | 3.020 | 3.394 | 3.249 | 3.450 | **3.072** |
| **G96PKZB** | 2.770 | 2.770 | 3.367 | 3.363 | 2.575 | 3.110 | 3.112 | 3.119 | 3.118 | 3.112 | 3.116 | 3.112 | 2.147 | 3.517 | 2.684 | 3.372 | 3.026 | 3.390 | 3.289 | 3.388 | **3.073** |
| **G96PL** | 2.766 | 2.765 | 3.362 | 3.359 | 2.628 | 3.111 | 3.101 | 3.083 | 3.112 | 3.102 | 3.086 | 3.108 | 2.147 | 3.568 | 2.689 | 3.381 | 2.998 | 3.421 | 3.257 | 3.386 | **3.071** |
| **G96PW91** | 2.768 | 2.766 | 3.364 | 3.366 | 2.537 | 3.115 | 3.115 | 3.112 | 3.114 | 3.110 | 3.113 | 3.109 | 2.151 | 3.464 | 2.682 | 3.369 | 3.045 | 3.385 | 3.247 | 3.401 | **3.067** |
| **G96RevTPSS** | 2.771 | 2.770 | 3.372 | 3.364 | 2.624 | 3.118 | 3.107 | 3.123 | 3.118 | 3.111 | 3.123 | 3.118 | 2.149 | 3.529 | 2.687 | 3.388 | 3.019 | 3.382 | 3.246 | 3.401 | **3.076** |
| **G96TPSS** | 2.767 | 2.770 | 3.371 | 3.368 | 2.580 | 3.105 | 3.113 | 3.120 | 3.115 | 3.111 | 3.120 | 3.113 | 2.151 | 3.473 | 2.685 | 3.369 | 3.022 | 3.389 | 3.249 | 3.390 | **3.069** |
| **G96VP86** | 2.777 | 2.778 | 3.353 | 3.355 | 2.690 | 3.064 | 3.056 | 3.039 | 3.059 | 3.052 | 3.045 | 3.053 | 2.178 | 3.552 | 2.636 | 3.218 | 3.019 | 3.383 | 3.243 | 3.450 | **3.050** |
| **G96VWN** | 2.794 | 2.791 | 3.255 | 3.259 | 2.597 | 3.069 | 3.077 | 3.076 | 3.070 | 3.072 | 3.091 | 3.071 | 2.149 | 3.405 | 2.679 | 3.222 | 2.981 | 3.308 | 3.248 | 3.384 | **3.030** |
| **G96VWN5** | 2.765 | 2.764 | 3.360 | 3.352 | 2.624 | 3.113 | 3.092 | 3.082 | 3.083 | 3.092 | 3.084 | 3.111 | 2.144 | 3.549 | 2.689 | 3.379 | 2.992 | 3.409 | 3.260 | 3.388 | **3.067** |
| **LGB95** | 0.107 | 0.101 | 0.093 | 0.093 | 0.187 | 0.081 | 0.065 | 0.067 | 0.081 | 0.065 | 0.067 | 0.080 | 0.266 | 0.098 | 0.150 | 0.055 | 0.100 | 0.091 | 0.093 | 0.095 | **0.102** |
| **LGBRC** | 0.146 | 0.154 | 0.135 | 0.134 | 0.274 | 0.157 | 0.145 | 0.125 | 0.155 | 0.133 | 0.116 | 0.171 | 0.305 | 0.136 | 0.201 | 0.137 | 0.148 | 0.140 | 0.133 | 0.142 | **0.159** |
| **LGKCIS** | 0.135 | 0.142 | 0.070 | 0.071 | 0.242 | 0.088 | 0.082 | 0.074 | 0.088 | 0.080 | 0.073 | 0.105 | 0.295 | 0.075 | 0.180 | 0.078 | 0.116 | 0.072 | 0.076 | 0.070 | **0.111** |
| **LGLYP** | 0.326 | 0.328 | 0.293 | 0.293 | 0.378 | 0.284 | 0.295 | 0.298 | 0.285 | 0.296 | 0.299 | 0.284 | 0.459 | 0.289 | 0.348 | 0.289 | 0.309 | 0.295 | 0.294 | 0.294 | **0.312** |
| **LGP86** | 0.220 | 0.225 | 0.130 | 0.130 | 0.277 | 0.094 | 0.171 | 0.187 | 0.111 | 0.174 | 0.189 | 0.090 | 0.330 | 0.101 | 0.250 | 0.160 | 0.172 | 0.152 | 0.144 | 0.141 | **0.172** |
| **LGPBE** | 0.090 | 0.109 | 0.181 | 0.181 | 0.240 | 0.209 | 0.140 | 0.132 | 0.207 | 0.138 | 0.133 | 0.227 | 0.253 | 0.227 | 0.174 | 0.172 | 0.131 | 0.177 | 0.176 | 0.177 | **0.174** |
| **LGPKZB** | 0.090 | 0.111 | 0.189 | 0.189 | 0.243 | 0.217 | 0.148 | 0.140 | 0.216 | 0.147 | 0.142 | 0.234 | 0.251 | 0.233 | 0.180 | 0.187 | 0.138 | 0.200 | 0.179 | 0.187 | **0.181** |
| **LGPL** | 0.166 | 0.173 | 0.232 | 0.232 | 0.252 | 0.256 | 0.218 | 0.185 | 0.255 | 0.217 | 0.185 | 0.264 | 0.278 | 0.268 | 0.202 | 0.266 | 0.192 | 0.246 | 0.228 | 0.229 | **0.227** |
| **LGPW91** | 0.098 | 0.110 | 0.146 | 0.146 | 0.233 | 0.169 | 0.128 | 0.100 | 0.168 | 0.127 | 0.101 | 0.192 | 0.259 | 0.180 | 0.161 | 0.134 | 0.124 | 0.146 | 0.139 | 0.140 | **0.150** |
| **LGRevTPSS** | 0.105 | 0.116 | 0.120 | 0.120 | 0.231 | 0.145 | 0.098 | 0.068 | 0.141 | 0.092 | 0.067 | 0.161 | 0.267 | 0.150 | 0.165 | 0.093 | 0.114 | 0.114 | 0.115 | 0.118 | **0.130** |
| **LGTPSS** | 0.091 | 0.111 | 0.187 | 0.187 | 0.241 | 0.219 | 0.146 | 0.138 | 0.214 | 0.146 | 0.143 | 0.236 | 0.255 | 0.232 | 0.178 | 0.185 | 0.136 | 0.187 | 0.175 | 0.184 | **0.179** |
| **LGVP86** | 0.217 | 0.223 | 0.129 | 0.128 | 0.276 | 0.100 | 0.161 | 0.176 | 0.109 | 0.169 | 0.186 | 0.096 | 0.329 | 0.099 | 0.238 | 0.157 | 0.170 | 0.145 | 0.137 | 0.138 | **0.169** |
| **LGVWN** | 0.156 | 0.169 | 0.189 | 0.189 | 0.259 | 0.204 | 0.187 | 0.160 | 0.203 | 0.182 | 0.164 | 0.212 | 0.280 | 0.218 | 0.194 | 0.189 | 0.158 | 0.194 | 0.183 | 0.184 | **0.194** |
| **LGVWN5** | 0.167 | 0.173 | 0.234 | 0.234 | 0.254 | 0.257 | 0.218 | 0.188 | 0.256 | 0.218 | 0.186 | 0.267 | 0.284 | 0.271 | 0.204 | 0.263 | 0.194 | 0.247 | 0.247 | 0.232 | **0.230** |
| **mPWB95** | 0.128 | 0.127 | 0.166 | 0.166 | 0.174 | 0.152 | 0.151 | 0.150 | 0.152 | 0.150 | 0.150 | 0.153 | 0.172 | 0.168 | 0.137 | 0.160 | 0.162 | 0.167 | 0.166 | 0.168 | **0.156** |
| **mPWBRC** | 0.285 | 0.287 | 0.458 | 0.458 | 0.336 | 0.452 | 0.414 | 0.397 | 0.451 | 0.413 | 0.396 | 0.452 | 0.281 | 0.416 | 0.269 | 0.485 | 0.341 | 0.498 | 0.457 | 0.495 | **0.402** |
| **mPWKCIS** | 0.258 | 0.262 | 0.410 | 0.410 | 0.308 | 0.398 | 0.370 | 0.350 | 0.397 | 0.370 | 0.347 | 0.402 | 0.270 | 0.383 | 0.238 | 0.436 | 0.300 | 0.438 | 0.404 | 0.433 | **0.359** |
| **mPWLYP** | 0.248 | 0.251 | 0.208 | 0.209 | 0.352 | 0.219 | 0.225 | 0.227 | 0.219 | 0.225 | 0.227 | 0.225 | 0.380 | 0.213 | 0.297 | 0.208 | 0.245 | 0.205 | 0.213 | 0.208 | **0.240** |
| **mPWP86** | 0.178 | 0.184 | 0.424 | 0.424 | 0.276 | 0.392 | 0.300 | 0.272 | 0.392 | 0.298 | 0.271 | 0.421 | 0.232 | 0.365 | 0.224 | 0.397 | 0.269 | 0.441 | 0.410 | 0.440 | **0.331** |
| **mPWPBE** | 0.360 | 0.354 | 0.517 | 0.517 | 0.353 | 0.538 | 0.495 | 0.470 | 0.537 | 0.495 | 0.467 | 0.538 | 0.291 | 0.476 | 0.321 | 0.570 | 0.401 | 0.551 | 0.516 | 0.540 | **0.465** |
| **mPWPKZB** | 0.363 | 0.359 | 0.520 | 0.520 | 0.354 | 0.537 | 0.498 | 0.470 | 0.536 | 0.501 | 0.471 | 0.537 | 0.292 | 0.479 | 0.324 | 0.569 | 0.406 | 0.554 | 0.521 | 0.543 | **0.468** |
| **mPWPL** | 0.362 | 0.357 | 0.484 | 0.484 | 0.342 | 0.502 | 0.464 | 0.437 | 0.502 | 0.462 | 0.434 | 0.504 | 0.275 | 0.443 | 0.319 | 0.543 | 0.380 | 0.518 | 0.486 | 0.508 | **0.440** |
| **mPWPW91** | 0.351 | 0.349 | 0.511 | 0.511 | 0.352 | 0.521 | 0.485 | 0.455 | 0.523 | 0.483 | 0.454 | 0.527 | 0.291 | 0.470 | 0.315 | 0.557 | 0.392 | 0.545 | 0.509 | 0.532 | **0.457** |
| **mPWRevTPSS** | 0.328 | 0.323 | 0.491 | 0.491 | 0.345 | 0.497 | 0.452 | 0.420 | 0.496 | 0.451 | 0.420 | 0.501 | 0.280 | 0.452 | 0.299 | 0.519 | 0.375 | 0.519 | 0.489 | 0.510 | **0.433** |
| **mPWTPSS** | 0.362 | 0.357 | 0.522 | 0.522 | 0.354 | 0.539 | 0.500 | 0.473 | 0.538 | 0.500 | 0.473 | 0.540 | 0.291 | 0.481 | 0.323 | 0.574 | 0.406 | 0.554 | 0.520 | 0.543 | **0.469** |
| **mPWVP86** | 0.180 | 0.185 | 0.427 | 0.427 | 0.277 | 0.398 | 0.303 | 0.275 | 0.393 | 0.301 | 0.274 | 0.426 | 0.230 | 0.369 | 0.229 | 0.399 | 0.272 | 0.449 | 0.415 | 0.448 | **0.334** |
| **mPWVWN** | 0.335 | 0.332 | 0.440 | 0.440 | 0.332 | 0.431 | 0.406 | 0.392 | 0.428 | 0.407 | 0.391 | 0.432 | 0.264 | 0.409 | 0.305 | 0.476 | 0.355 | 0.474 | 0.439 | 0.454 | **0.397** |
| **mPWVWN5** | 0.363 | 0.358 | 0.486 | 0.486 | 0.342 | 0.503 | 0.465 | 0.439 | 0.502 | 0.463 | 0.437 | 0.504 | 0.274 | 0.444 | 0.320 | 0.545 | 0.381 | 0.520 | 0.489 | 0.509 | **0.442** |
| **OB95** | 0.310 | 0.310 | 0.407 | 0.407 | 0.266 | 0.415 | 0.415 | 0.370 | 0.415 | 0.415 | 0.370 | 0.452 | 0.225 | 0.379 | 0.303 | 0.432 | 0.368 | 0.400 | 0.394 | 0.339 | **0.370** |
| **OBRC** | 0.478 | 0.474 | 0.711 | 0.711 | 0.389 | 0.709 | 0.695 | 0.672 | 0.708 | 0.695 | 0.673 | 0.709 | 0.294 | 0.633 | 0.407 | 0.763 | 0.566 | 0.781 | 0.706 | 0.752 | **0.626** |
| **OKCIS** | 0.471 | 0.466 | 0.686 | 0.686 | 0.378 | 0.688 | 0.667 | 0.647 | 0.687 | 0.666 | 0.647 | 0.692 | 0.285 | 0.609 | 0.408 | 0.725 | 0.547 | 0.717 | 0.682 | 0.724 | **0.604** |
| **OLYP** | 0.308 | 0.310 | 0.456 | 0.456 | 0.343 | 0.445 | 0.431 | 0.415 | 0.445 | 0.430 | 0.415 | 0.446 | 0.321 | 0.387 | 0.305 | 0.509 | 0.366 | 0.492 | 0.465 | 0.472 | **0.411** |
| **OP86** | 0.559 | 0.553 | 0.877 | 0.875 | 0.444 | 0.775 | 0.768 | 0.713 | 0.775 | 0.768 | 0.712 | 0.803 | 0.337 | 0.760 | 0.529 | 0.942 | 0.650 | 0.943 | 0.803 | 0.904 | **0.725** |
| **OPBE** | 0.535 | 0.531 | 0.761 | 0.761 | 0.420 | 0.729 | 0.707 | 0.684 | 0.728 | 0.706 | 0.684 | 0.727 | 0.326 | 0.668 | 0.481 | 0.833 | 0.599 | 0.822 | 0.725 | 0.822 | **0.662** |
| **OPKZB** | 0.538 | 0.534 | 0.764 | 0.764 | 0.422 | 0.730 | 0.709 | 0.687 | 0.730 | 0.709 | 0.687 | 0.732 | 0.326 | 0.672 | 0.484 | 0.833 | 0.603 | 0.820 | 0.728 | 0.831 | **0.665** |
| **OPL** | 0.523 | 0.515 | 0.708 | 0.708 | 0.410 | 0.713 | 0.692 | 0.672 | 0.712 | 0.692 | 0.672 | 0.714 | 0.312 | 0.635 | 0.468 | 0.755 | 0.584 | 0.771 | 0.705 | 0.731 | **0.635** |
| **OPW91** | 0.533 | 0.529 | 0.725 | 0.725 | 0.419 | 0.726 | 0.702 | 0.682 | 0.726 | 0.702 | 0.681 | 0.726 | 0.323 | 0.661 | 0.479 | 0.829 | 0.599 | 0.820 | 0.721 | 0.806 | **0.656** |
| **ORevTPSS** | 0.527 | 0.522 | 0.718 | 0.718 | 0.411 | 0.720 | 0.696 | 0.675 | 0.719 | 0.696 | 0.675 | 0.718 | 0.313 | 0.654 | 0.471 | 0.815 | 0.591 | 0.804 | 0.713 | 0.798 | **0.648** |
| **OTPSS** | 0.538 | 0.534 | 0.765 | 0.765 | 0.421 | 0.732 | 0.710 | 0.688 | 0.732 | 0.710 | 0.688 | 0.734 | 0.326 | 0.673 | 0.483 | 0.835 | 0.603 | 0.825 | 0.731 | 0.826 | **0.666** |
| **OVP86** | 0.560 | 0.554 | 0.878 | 0.877 | 0.462 | 0.776 | 0.768 | 0.713 | 0.776 | 0.768 | 0.713 | 0.804 | 0.337 | 0.760 | 0.530 | 0.926 | 0.649 | 0.944 | 0.804 | 0.921 | **0.726** |
| **OVWN** | 0.499 | 0.493 | 0.667 | 0.667 | 0.397 | 0.661 | 0.644 | 0.622 | 0.661 | 0.641 | 0.622 | 0.661 | 0.310 | 0.599 | 0.429 | 0.707 | 0.556 | 0.702 | 0.666 | 0.693 | **0.595** |
| **OVWN5** | 0.524 | 0.516 | 0.708 | 0.708 | 0.410 | 0.713 | 0.692 | 0.673 | 0.713 | 0.693 | 0.673 | 0.714 | 0.312 | 0.636 | 0.469 | 0.755 | 0.585 | 0.772 | 0.706 | 0.731 | **0.635** |
| **PBEB95** | 0.119 | 0.120 | 0.109 | 0.109 | 0.213 | 0.057 | 0.064 | 0.065 | 0.057 | 0.064 | 0.065 | 0.061 | 0.300 | 0.037 | 0.173 | 0.084 | 0.114 | 0.092 | 0.109 | 0.097 | **0.106** |

| | | | | | | | | | | | | | | | | | | | | | |
|---|---|---|---|---|---|---|---|---|---|---|---|---|---|---|---|---|---|---|---|---|---|
| **PBEBRC** | 0.181 | 0.185 | 0.158 | 0.159 | 0.288 | 0.160 | 0.153 | 0.159 | 0.159 | 0.153 | 0.159 | 0.167 | 0.325 | 0.147 | 0.233 | 0.162 | 0.177 | 0.161 | 0.160 | 0.160 | **0.180** |
| **PBEKCIS** | 0.180 | 0.183 | 0.128 | 0.128 | 0.273 | 0.129 | 0.129 | 0.137 | 0.127 | 0.129 | 0.137 | 0.138 | 0.321 | 0.116 | 0.224 | 0.125 | 0.167 | 0.127 | 0.130 | 0.127 | **0.158** |
| **PBELYP** | 0.319 | 0.320 | 0.287 | 0.287 | 0.371 | 0.281 | 0.288 | 0.290 | 0.282 | 0.289 | 0.291 | 0.282 | 0.448 | 0.286 | 0.339 | 0.280 | 0.305 | 0.288 | 0.287 | 0.286 | **0.305** |
| **PBEP86** | 0.222 | 0.225 | 0.174 | 0.174 | 0.279 | 0.166 | 0.183 | 0.191 | 0.167 | 0.184 | 0.192 | 0.170 | 0.366 | 0.166 | 0.250 | 0.178 | 0.200 | 0.180 | 0.178 | 0.176 | **0.201** |
| **PBEPBE** | 0.157 | 0.161 | 0.140 | 0.139 | 0.262 | 0.137 | 0.132 | 0.124 | 0.136 | 0.131 | 0.124 | 0.147 | 0.305 | 0.126 | 0.208 | 0.140 | 0.153 | 0.145 | 0.141 | 0.141 | **0.158** |
| **PBEPKZB** | 0.156 | 0.161 | 0.144 | 0.144 | 0.261 | 0.140 | 0.134 | 0.126 | 0.140 | 0.134 | 0.126 | 0.148 | 0.304 | 0.132 | 0.208 | 0.146 | 0.153 | 0.149 | 0.145 | 0.145 | **0.160** |
| **PBEPL** | 0.183 | 0.187 | 0.190 | 0.190 | 0.274 | 0.182 | 0.174 | 0.168 | 0.181 | 0.174 | 0.167 | 0.189 | 0.311 | 0.185 | 0.220 | 0.201 | 0.179 | 0.199 | 0.191 | 0.191 | **0.197** |
| **PBEPW91** | 0.161 | 0.166 | 0.136 | 0.136 | 0.263 | 0.136 | 0.130 | 0.131 | 0.135 | 0.130 | 0.131 | 0.146 | 0.307 | 0.125 | 0.210 | 0.134 | 0.157 | 0.139 | 0.139 | 0.137 | **0.157** |
| **PBERevTPSS** | 0.163 | 0.167 | 0.135 | 0.135 | 0.261 | 0.132 | 0.129 | 0.132 | 0.132 | 0.129 | 0.132 | 0.143 | 0.307 | 0.122 | 0.209 | 0.130 | 0.158 | 0.134 | 0.136 | 0.135 | **0.156** |
| **PBETPSS** | 0.156 | 0.161 | 0.143 | 0.143 | 0.261 | 0.139 | 0.133 | 0.125 | 0.138 | 0.134 | 0.125 | 0.147 | 0.305 | 0.131 | 0.208 | 0.145 | 0.153 | 0.148 | 0.144 | 0.144 | **0.159** |
| **PBEVP86** | 0.221 | 0.224 | 0.172 | 0.172 | 0.278 | 0.165 | 0.181 | 0.190 | 0.166 | 0.182 | 0.191 | 0.169 | 0.365 | 0.165 | 0.249 | 0.177 | 0.199 | 0.178 | 0.177 | 0.175 | **0.200** |
| **PBEVWN** | 0.174 | 0.178 | 0.180 | 0.180 | 0.275 | 0.176 | 0.167 | 0.160 | 0.176 | 0.166 | 0.160 | 0.183 | 0.313 | 0.174 | 0.223 | 0.188 | 0.172 | 0.184 | 0.182 | 0.181 | **0.189** |
| **PBEVWN5** | 0.183 | 0.187 | 0.190 | 0.190 | 0.274 | 0.182 | 0.175 | 0.168 | 0.182 | 0.174 | 0.168 | 0.189 | 0.311 | 0.186 | 0.220 | 0.201 | 0.179 | 0.200 | 0.191 | 0.191 | **0.197** |
| **PBEhB95** | 0.119 | 0.120 | 0.108 | 0.108 | 0.213 | 0.049 | 0.099 | 0.082 | 0.049 | 0.100 | 0.082 | 0.066 | 0.316 | 0.037 | 0.188 | 0.084 | 0.114 | 0.105 | 0.097 | 0.110 | **0.112** |
| **PBEhBRC** | 0.184 | 0.187 | 0.159 | 0.159 | 0.288 | 0.162 | 0.157 | 0.163 | 0.161 | 0.157 | 0.163 | 0.170 | 0.325 | 0.153 | 0.235 | 0.164 | 0.180 | 0.162 | 0.161 | 0.161 | **0.183** |
| **PBEhKCIS** | 0.178 | 0.183 | 0.131 | 0.131 | 0.272 | 0.131 | 0.133 | 0.140 | 0.130 | 0.133 | 0.142 | 0.140 | 0.321 | 0.123 | 0.224 | 0.127 | 0.171 | 0.129 | 0.133 | 0.130 | **0.160** |
| **PBEhLYP** | 0.314 | 0.315 | 0.283 | 0.284 | 0.369 | 0.277 | 0.284 | 0.285 | 0.278 | 0.285 | 0.286 | 0.279 | 0.444 | 0.283 | 0.336 | 0.274 | 0.302 | 0.285 | 0.284 | 0.283 | **0.301** |
| **PBEhP86** | 0.221 | 0.224 | 0.174 | 0.174 | 0.278 | 0.167 | 0.182 | 0.189 | 0.168 | 0.183 | 0.191 | 0.172 | 0.363 | 0.164 | 0.247 | 0.176 | 0.202 | 0.181 | 0.179 | 0.179 | **0.201** |
| **PBEhPBE** | 0.159 | 0.163 | 0.141 | 0.141 | 0.262 | 0.141 | 0.132 | 0.129 | 0.140 | 0.132 | 0.128 | 0.150 | 0.305 | 0.133 | 0.211 | 0.142 | 0.158 | 0.144 | 0.142 | 0.142 | **0.160** |
| **PBEhPKZB** | 0.159 | 0.163 | 0.145 | 0.145 | 0.263 | 0.144 | 0.134 | 0.130 | 0.144 | 0.135 | 0.130 | 0.155 | 0.304 | 0.138 | 0.211 | 0.148 | 0.158 | 0.149 | 0.147 | 0.147 | **0.162** |
| **PBEhPL** | 0.184 | 0.188 | 0.190 | 0.190 | 0.277 | 0.186 | 0.177 | 0.171 | 0.185 | 0.178 | 0.171 | 0.192 | 0.312 | 0.184 | 0.222 | 0.203 | 0.181 | 0.198 | 0.190 | 0.190 | **0.198** |
| **PBEhPW91** | 0.163 | 0.167 | 0.140 | 0.140 | 0.263 | 0.136 | 0.133 | 0.133 | 0.135 | 0.134 | 0.134 | 0.147 | 0.307 | 0.130 | 0.212 | 0.136 | 0.160 | 0.140 | 0.141 | 0.140 | **0.160** |
| **PBEhRevTPSS** | 0.165 | 0.169 | 0.135 | 0.135 | 0.261 | 0.136 | 0.132 | 0.135 | 0.135 | 0.131 | 0.135 | 0.147 | 0.307 | 0.127 | 0.212 | 0.135 | 0.161 | 0.138 | 0.138 | 0.138 | **0.159** |
| **PBEhTPSS** | 0.159 | 0.163 | 0.144 | 0.144 | 0.262 | 0.142 | 0.134 | 0.129 | 0.142 | 0.133 | 0.129 | 0.153 | 0.305 | 0.136 | 0.211 | 0.147 | 0.158 | 0.148 | 0.145 | 0.145 | **0.161** |
| **PBEhVP86** | 0.220 | 0.223 | 0.174 | 0.174 | 0.278 | 0.167 | 0.181 | 0.188 | 0.167 | 0.182 | 0.189 | 0.171 | 0.361 | 0.163 | 0.245 | 0.175 | 0.201 | 0.180 | 0.179 | 0.177 | **0.200** |
| **PBEhVWN** | 0.177 | 0.180 | 0.182 | 0.182 | 0.277 | 0.181 | 0.169 | 0.165 | 0.181 | 0.169 | 0.164 | 0.186 | 0.314 | 0.178 | 0.225 | 0.190 | 0.174 | 0.185 | 0.183 | 0.182 | **0.192** |
| **PBEhVWN5** | 0.184 | 0.188 | 0.190 | 0.190 | 0.276 | 0.186 | 0.178 | 0.171 | 0.186 | 0.178 | 0.172 | 0.192 | 0.312 | 0.185 | 0.222 | 0.203 | 0.182 | 0.199 | 0.190 | 0.191 | **0.199** |
| **PKZBB95** | 0.166 | 0.166 | 0.196 | 0.196 | 0.227 | 0.164 | 0.163 | 0.162 | 0.164 | 0.163 | 0.162 | 0.134 | 0.234 | 0.150 | 0.190 | 0.185 | 0.193 | 0.195 | 0.195 | 0.197 | **0.180** |
| **PKZBBRC** | 0.206 | 0.207 | 0.250 | 0.250 | 0.271 | 0.239 | 0.232 | 0.226 | 0.238 | 0.231 | 0.226 | 0.243 | 0.306 | 0.223 | 0.210 | 0.255 | 0.218 | 0.256 | 0.254 | 0.257 | **0.240** |
| **PKZBKCIS** | 0.195 | 0.198 | 0.218 | 0.218 | 0.262 | 0.207 | 0.203 | 0.194 | 0.207 | 0.202 | 0.195 | 0.214 | 0.299 | 0.200 | 0.203 | 0.225 | 0.197 | 0.223 | 0.221 | 0.222 | **0.215** |
| **PKZBLYP** | 0.244 | 0.244 | 0.212 | 0.212 | 0.339 | 0.229 | 0.232 | 0.232 | 0.229 | 0.232 | 0.233 | 0.230 | 0.369 | 0.230 | 0.288 | 0.212 | 0.240 | 0.210 | 0.214 | 0.212 | **0.242** |
| **PKZBP86** | 0.176 | 0.178 | 0.178 | 0.179 | 0.267 | 0.176 | 0.171 | 0.164 | 0.176 | 0.171 | 0.164 | 0.180 | 0.296 | 0.169 | 0.213 | 0.179 | 0.167 | 0.180 | 0.179 | 0.183 | **0.187** |
| **PKZBPBE** | 0.205 | 0.207 | 0.255 | 0.255 | 0.260 | 0.241 | 0.232 | 0.220 | 0.240 | 0.232 | 0.220 | 0.246 | 0.286 | 0.237 | 0.202 | 0.266 | 0.217 | 0.261 | 0.258 | 0.261 | **0.240** |
| **PKZBPKZB** | 0.205 | 0.208 | 0.260 | 0.260 | 0.260 | 0.244 | 0.236 | 0.224 | 0.244 | 0.235 | 0.224 | 0.249 | 0.285 | 0.241 | 0.202 | 0.270 | 0.220 | 0.265 | 0.262 | 0.265 | **0.243** |
| **PKZBPL** | 0.224 | 0.226 | 0.280 | 0.280 | 0.263 | 0.266 | 0.260 | 0.250 | 0.266 | 0.260 | 0.249 | 0.270 | 0.288 | 0.269 | 0.206 | 0.293 | 0.244 | 0.284 | 0.283 | 0.283 | **0.262** |
| **PKZBPW91** | 0.204 | 0.206 | 0.248 | 0.248 | 0.261 | 0.235 | 0.228 | 0.215 | 0.235 | 0.227 | 0.214 | 0.240 | 0.288 | 0.231 | 0.201 | 0.259 | 0.212 | 0.254 | 0.251 | 0.254 | **0.236** |
| **PKZBRevTPSS** | 0.200 | 0.203 | 0.238 | 0.239 | 0.259 | 0.227 | 0.219 | 0.205 | 0.227 | 0.218 | 0.205 | 0.233 | 0.288 | 0.223 | 0.199 | 0.247 | 0.204 | 0.243 | 0.241 | 0.243 | **0.228** |
| **PKZBTPSS** | 0.205 | 0.207 | 0.260 | 0.260 | 0.259 | 0.244 | 0.235 | 0.223 | 0.243 | 0.235 | 0.223 | 0.249 | 0.285 | 0.241 | 0.202 | 0.270 | 0.220 | 0.265 | 0.262 | 0.265 | **0.243** |
| **PKZBVP86** | 0.176 | 0.178 | 0.178 | 0.178 | 0.267 | 0.176 | 0.171 | 0.164 | 0.176 | 0.171 | 0.164 | 0.180 | 0.296 | 0.169 | 0.213 | 0.179 | 0.168 | 0.181 | 0.179 | 0.183 | **0.187** |
| **PKZBVWN** | 0.217 | 0.219 | 0.252 | 0.252 | 0.261 | 0.242 | 0.237 | 0.225 | 0.241 | 0.236 | 0.224 | 0.246 | 0.291 | 0.244 | 0.203 | 0.265 | 0.223 | 0.255 | 0.256 | 0.255 | **0.242** |
| **PKZBVWN5** | 0.224 | 0.226 | 0.281 | 0.281 | 0.263 | 0.266 | 0.261 | 0.250 | 0.266 | 0.260 | 0.250 | 0.271 | 0.288 | 0.270 | 0.206 | 0.293 | 0.244 | 0.284 | 0.284 | 0.284 | **0.263** |
| **PW91B95** | 0.165 | 0.166 | 0.117 | 0.117 | 0.241 | 0.120 | 0.126 | 0.121 | 0.120 | 0.126 | 0.122 | 0.120 | 0.374 | 0.123 | 0.195 | 0.107 | 0.158 | 0.109 | 0.117 | 0.089 | **0.147** |
| **PW91BRC** | 0.203 | 0.205 | 0.168 | 0.168 | 0.306 | 0.181 | 0.186 | 0.191 | 0.181 | 0.187 | 0.192 | 0.185 | 0.341 | 0.173 | 0.254 | 0.171 | 0.202 | 0.172 | 0.169 | 0.167 | **0.200** |
| **PW91KCIS** | 0.204 | 0.206 | 0.159 | 0.159 | 0.295 | 0.164 | 0.170 | 0.175 | 0.164 | 0.171 | 0.176 | 0.169 | 0.336 | 0.161 | 0.247 | 0.153 | 0.197 | 0.154 | 0.162 | 0.154 | **0.189** |
| **PW91LYP** | 0.325 | 0.325 | 0.290 | 0.290 | 0.379 | 0.289 | 0.295 | 0.297 | 0.290 | 0.296 | 0.298 | 0.291 | 0.463 | 0.292 | 0.345 | 0.285 | 0.309 | 0.290 | 0.291 | 0.288 | **0.312** |
| **PW91P86** | 0.238 | 0.240 | 0.196 | 0.196 | 0.299 | 0.196 | 0.206 | 0.211 | 0.197 | 0.207 | 0.212 | 0.201 | 0.391 | 0.199 | 0.268 | 0.197 | 0.219 | 0.195 | 0.198 | 0.194 | **0.223** |
| **PW91PBE** | 0.189 | 0.191 | 0.156 | 0.156 | 0.286 | 0.157 | 0.163 | 0.169 | 0.156 | 0.163 | 0.169 | 0.162 | 0.327 | 0.152 | 0.236 | 0.154 | 0.186 | 0.161 | 0.157 | 0.158 | **0.182** |
| **PW91PKZB** | 0.187 | 0.190 | 0.161 | 0.160 | 0.286 | 0.157 | 0.163 | 0.168 | 0.157 | 0.163 | 0.169 | 0.164 | 0.326 | 0.153 | 0.236 | 0.158 | 0.186 | 0.164 | 0.161 | 0.162 | **0.184** |
| **PW91PL** | 0.192 | 0.194 | 0.193 | 0.193 | 0.295 | 0.185 | 0.180 | 0.177 | 0.185 | 0.180 | 0.177 | 0.191 | 0.330 | 0.183 | 0.243 | 0.200 | 0.192 | 0.199 | 0.194 | 0.194 | **0.204** |
| **PW91PW91** | 0.191 | 0.193 | 0.153 | 0.153 | 0.287 | 0.160 | 0.167 | 0.173 | 0.161 | 0.167 | 0.173 | 0.164 | 0.328 | 0.155 | 0.237 | 0.150 | 0.189 | 0.159 | 0.155 | 0.153 | **0.183** |
| **PW91RevTPSS** | 0.191 | 0.193 | 0.154 | 0.154 | 0.285 | 0.160 | 0.167 | 0.173 | 0.161 | 0.167 | 0.173 | 0.166 | 0.326 | 0.155 | 0.236 | 0.149 | 0.188 | 0.152 | 0.155 | 0.152 | **0.183** |
| **PW91TPSS** | 0.187 | 0.189 | 0.159 | 0.159 | 0.285 | 0.156 | 0.161 | 0.168 | 0.156 | 0.162 | 0.168 | 0.163 | 0.327 | 0.151 | 0.235 | 0.157 | 0.185 | 0.163 | 0.160 | 0.161 | **0.183** |
| **PW91VP86** | 0.237 | 0.239 | 0.195 | 0.195 | 0.298 | 0.196 | 0.205 | 0.211 | 0.197 | 0.206 | 0.212 | 0.201 | 0.390 | 0.199 | 0.268 | 0.196 | 0.218 | 0.194 | 0.197 | 0.193 | **0.222** |
| **PW91VWN** | 0.196 | 0.198 | 0.185 | 0.185 | 0.293 | 0.180 | 0.178 | 0.184 | 0.180 | 0.179 | 0.184 | 0.184 | 0.330 | 0.177 | 0.245 | 0.192 | 0.196 | 0.190 | 0.186 | 0.185 | **0.201** |
| **PW91VWN5** | 0.191 | 0.193 | 0.194 | 0.194 | 0.294 | 0.185 | 0.181 | 0.177 | 0.185 | 0.181 | 0.177 | 0.191 | 0.330 | 0.184 | 0.243 | 0.200 | 0.191 | 0.198 | 0.195 | 0.194 | **0.204** |
| **RevTPSSB95** | 0.107 | 0.101 | 0.113 | 0.113 | 0.153 | 0.096 | 0.099 | 0.098 | 0.096 | 0.099 | 0.098 | 0.120 | 0.173 | 0.111 | 0.115 | 0.097 | 0.109 | 0.109 | 0.110 | 0.111 | **0.111** |
| **RevTPSSBRC** | 0.223 | 0.224 | 0.314 | 0.314 | 0.297 | 0.300 | 0.278 | 0.265 | 0.300 | 0.277 | 0.263 | 0.309 | 0.278 | 0.280 | 0.233 | 0.329 | 0.254 | 0.334 | 0.308 | 0.327 | **0.285** |

| | | | | | | | | | | | | | | | | | | | | | |
|---|---|---|---|---|---|---|---|---|---|---|---|---|---|---|---|---|---|---|---|---|---|
| **RevTPSSKCIS** | 0.195 | 0.196 | 0.258 | 0.258 | 0.277 | 0.243 | 0.223 | 0.206 | 0.241 | 0.223 | 0.205 | 0.251 | 0.251 | 0.245 | 0.207 | 0.276 | 0.217 | 0.266 | 0.256 | 0.257 | **0.238** |
| **RevTPSSLYP** | 0.275 | 0.276 | 0.249 | 0.249 | 0.355 | 0.253 | 0.258 | 0.261 | 0.254 | 0.259 | 0.262 | 0.257 | 0.392 | 0.256 | 0.314 | 0.246 | 0.272 | 0.249 | 0.252 | 0.249 | **0.272** |
| **RevTPSSP86** | 0.108 | 0.113 | 0.145 | 0.144 | 0.215 | 0.135 | 0.119 | **0.099** | 0.136 | 0.118 | **0.098** | 0.149 | 0.242 | 0.140 | 0.151 | 0.126 | 0.115 | 0.141 | 0.138 | 0.143 | **0.139** |
| **RevTPSSPBE** | 0.251 | 0.261 | 0.364 | 0.364 | 0.304 | 0.332 | 0.294 | 0.282 | 0.330 | 0.293 | 0.282 | 0.353 | 0.281 | 0.350 | 0.244 | 0.379 | 0.269 | 0.398 | 0.352 | 0.364 | **0.317** |
| **RevTPSSPKZB** | 0.254 | 0.265 | 0.365 | 0.365 | 0.304 | 0.338 | 0.295 | 0.285 | 0.337 | 0.296 | 0.285 | 0.373 | 0.285 | 0.354 | 0.246 | 0.399 | 0.271 | 0.401 | 0.354 | 0.369 | **0.322** |
| **RevTPSSPL** | 0.292 | 0.293 | 0.375 | 0.375 | 0.308 | 0.362 | 0.330 | 0.315 | 0.361 | 0.329 | 0.313 | 0.366 | 0.264 | 0.351 | 0.273 | 0.412 | 0.304 | 0.397 | 0.368 | 0.375 | **0.338** |
| **RevTPSSPW91** | 0.245 | 0.255 | 0.350 | 0.350 | 0.303 | 0.322 | 0.287 | 0.276 | 0.321 | 0.287 | 0.276 | 0.326 | 0.273 | 0.345 | 0.239 | 0.366 | 0.263 | 0.384 | 0.346 | 0.349 | **0.308** |
| **RevTPSSRevTPS** | 0.239 | 0.241 | 0.337 | 0.337 | 0.293 | 0.305 | 0.277 | 0.265 | 0.306 | 0.276 | 0.264 | 0.311 | 0.265 | 0.330 | 0.223 | 0.348 | 0.251 | 0.369 | 0.331 | 0.340 | **0.295** |
| **RevTPSSTPSS** | 0.252 | 0.263 | 0.366 | 0.366 | 0.303 | 0.339 | 0.296 | 0.285 | 0.338 | 0.297 | 0.285 | 0.374 | 0.282 | 0.354 | 0.245 | 0.400 | 0.271 | 0.401 | 0.354 | 0.369 | **0.322** |
| **RevTPSSVP86** | 0.109 | 0.114 | 0.147 | 0.148 | 0.214 | 0.138 | 0.121 | 0.101 | 0.137 | 0.119 | 0.100 | 0.152 | 0.241 | 0.144 | 0.151 | 0.128 | 0.116 | 0.144 | 0.142 | 0.145 | **0.141** |
| **RevTPSSVWN** | 0.263 | 0.264 | 0.317 | 0.317 | 0.293 | 0.315 | 0.284 | 0.275 | 0.313 | 0.282 | 0.275 | 0.319 | 0.268 | 0.313 | 0.253 | 0.370 | 0.274 | 0.349 | 0.315 | 0.315 | **0.299** |
| **RevTPSSVWN5** | 0.294 | 0.295 | 0.377 | 0.377 | 0.308 | 0.363 | 0.332 | 0.316 | 0.362 | 0.331 | 0.315 | 0.367 | 0.264 | 0.353 | 0.274 | 0.414 | 0.306 | 0.403 | 0.370 | 0.380 | **0.340** |
| **SB95** | 0.642 | 0.643 | 0.637 | 0.637 | 0.632 | 0.597 | 0.615 | 0.629 | 0.600 | 0.619 | 0.633 | 0.583 | 0.707 | 0.632 | 0.639 | 0.645 | 0.647 | 0.647 | 0.643 | 0.645 | **0.634** |
| **SBRC** | 0.564 | 0.565 | 0.554 | 0.554 | 0.595 | 0.543 | 0.567 | 0.570 | 0.546 | 0.571 | 0.573 | 0.517 | 0.656 | 0.575 | 0.576 | 0.560 | 0.562 | 0.557 | 0.559 | 0.560 | **0.566** |
| **SKCIS** | 0.601 | 0.604 | 0.594 | 0.594 | 0.621 | 0.577 | 0.601 | 0.605 | 0.580 | 0.605 | 0.608 | 0.551 | 0.681 | 0.610 | 0.611 | 0.601 | 0.601 | 0.597 | 0.598 | 0.598 | **0.602** |
| **SLYP** | 0.699 | 0.701 | 0.695 | 0.695 | 0.714 | 0.677 | 0.695 | 0.697 | 0.679 | 0.698 | 0.700 | 0.654 | 0.757 | 0.694 | 0.704 | 0.696 | 0.699 | 0.695 | 0.696 | 0.696 | **0.697** |
| **SP86** | 0.696 | 0.699 | 0.700 | 0.700 | 0.709 | 0.681 | 0.704 | 0.707 | 0.684 | 0.708 | 0.710 | 0.653 | 0.752 | 0.708 | 0.702 | 0.705 | 0.702 | 0.702 | 0.703 | 0.704 | **0.701** |
| **SPBE** | 0.605 | 0.608 | 0.602 | 0.602 | 0.627 | 0.586 | 0.613 | 0.616 | 0.589 | 0.617 | 0.621 | 0.556 | 0.687 | 0.625 | 0.617 | 0.611 | 0.608 | 0.605 | 0.607 | 0.607 | **0.610** |
| **SPKZB** | 0.591 | 0.594 | 0.586 | 0.586 | 0.613 | 0.572 | 0.598 | 0.601 | 0.576 | 0.602 | 0.605 | 0.543 | 0.675 | 0.610 | 0.603 | 0.596 | 0.592 | 0.589 | 0.591 | 0.591 | **0.596** |
| **SPL** | 0.485 | 0.487 | 0.465 | 0.465 | 0.509 | 0.450 | 0.474 | 0.475 | 0.454 | 0.478 | 0.478 | 0.427 | 0.582 | 0.479 | 0.495 | 0.474 | 0.474 | 0.468 | 0.468 | 0.468 | **0.478** |
| **SPW91** | 0.613 | 0.615 | 0.610 | 0.610 | 0.633 | 0.594 | 0.620 | 0.624 | 0.598 | 0.624 | 0.628 | 0.565 | 0.692 | 0.631 | 0.623 | 0.619 | 0.616 | 0.613 | 0.615 | 0.615 | **0.618** |
| **SRevTPSS** | 0.604 | 0.606 | 0.599 | 0.599 | 0.625 | 0.585 | 0.610 | 0.613 | 0.588 | 0.614 | 0.617 | 0.556 | 0.683 | 0.620 | 0.614 | 0.608 | 0.605 | 0.602 | 0.603 | 0.604 | **0.608** |
| **STPSS** | 0.594 | 0.596 | 0.589 | 0.589 | 0.616 | 0.575 | 0.600 | 0.604 | 0.578 | 0.604 | 0.608 | 0.545 | 0.677 | 0.612 | 0.605 | 0.598 | 0.595 | 0.592 | 0.593 | 0.594 | **0.598** |
| **SVP86** | 0.696 | 0.699 | 0.700 | 0.700 | 0.709 | 0.681 | 0.704 | 0.707 | 0.684 | 0.708 | 0.711 | 0.653 | 0.752 | 0.708 | 0.702 | 0.705 | 0.702 | 0.702 | 0.703 | 0.704 | **0.701** |
| **SVWN5** | 0.484 | 0.486 | 0.464 | 0.464 | 0.508 | 0.450 | 0.474 | 0.475 | 0.453 | 0.477 | 0.478 | 0.426 | 0.581 | 0.479 | 0.495 | 0.473 | 0.474 | 0.468 | 0.467 | 0.467 | **0.477** |
| **TPSSB95** | 0.117 | 0.105 | 0.131 | 0.131 | 0.168 | 0.118 | 0.118 | 0.116 | 0.118 | 0.118 | 0.116 | 0.118 | 0.173 | 0.132 | 0.129 | 0.121 | 0.110 | 0.131 | 0.131 | 0.132 | **0.127** |
| **TPSSBRC** | 0.206 | 0.210 | 0.253 | 0.254 | 0.279 | 0.247 | 0.239 | 0.222 | 0.247 | 0.239 | 0.221 | 0.253 | 0.286 | 0.242 | 0.214 | 0.275 | 0.221 | 0.267 | 0.251 | 0.259 | **0.244** |
| **TPSSKCIS** | 0.176 | 0.181 | 0.207 | 0.207 | 0.262 | 0.213 | 0.211 | 0.185 | 0.211 | 0.210 | 0.183 | 0.218 | 0.279 | 0.214 | 0.194 | 0.235 | 0.191 | 0.220 | 0.207 | 0.205 | **0.210** |
| **TPSSLYP** | 0.280 | 0.281 | 0.252 | 0.252 | 0.355 | 0.254 | 0.259 | 0.262 | 0.255 | 0.259 | 0.262 | 0.258 | 0.392 | 0.261 | 0.315 | 0.247 | 0.274 | 0.249 | 0.254 | 0.251 | **0.273** |
| **TPSSP86** | 0.137 | 0.141 | 0.133 | 0.133 | 0.238 | 0.127 | 0.137 | 0.124 | 0.126 | 0.136 | 0.123 | 0.136 | 0.278 | 0.134 | 0.188 | 0.137 | 0.132 | 0.140 | 0.134 | 0.148 | **0.149** |
| **TPSSPBE** | 0.223 | 0.224 | 0.280 | 0.280 | 0.281 | 0.256 | 0.242 | 0.232 | 0.255 | 0.241 | 0.232 | 0.262 | 0.271 | 0.270 | 0.225 | 0.308 | 0.237 | 0.301 | 0.279 | 0.278 | **0.259** |
| **TPSSPKZB** | 0.230 | 0.240 | 0.283 | 0.283 | 0.281 | 0.259 | 0.245 | 0.235 | 0.258 | 0.244 | 0.234 | 0.267 | 0.272 | 0.272 | 0.227 | 0.311 | 0.242 | 0.309 | 0.281 | 0.282 | **0.263** |
| **TPSSPL** | 0.245 | 0.245 | 0.293 | 0.293 | 0.281 | 0.293 | 0.277 | 0.262 | 0.293 | 0.277 | 0.262 | 0.298 | 0.273 | 0.300 | 0.243 | 0.331 | 0.257 | 0.306 | 0.293 | 0.290 | **0.281** |
| **TPSSPW91** | 0.216 | 0.219 | 0.273 | 0.273 | 0.281 | 0.247 | 0.236 | 0.221 | 0.245 | 0.237 | 0.220 | 0.253 | 0.270 | 0.263 | 0.216 | 0.301 | 0.231 | 0.282 | 0.271 | 0.271 | **0.251** |
| **TPSSRevTPSS** | 0.206 | 0.209 | 0.260 | 0.260 | 0.274 | 0.237 | 0.234 | 0.209 | 0.235 | 0.234 | 0.208 | 0.241 | 0.266 | 0.243 | 0.209 | 0.278 | 0.219 | 0.270 | 0.247 | 0.260 | **0.240** |
| **TPSSTPSS** | 0.230 | 0.239 | 0.283 | 0.283 | 0.280 | 0.260 | 0.244 | 0.234 | 0.259 | 0.244 | 0.234 | 0.267 | 0.272 | 0.273 | 0.227 | 0.308 | 0.241 | 0.308 | 0.281 | 0.281 | **0.262** |
| **TPSSVP86** | 0.137 | 0.142 | 0.133 | 0.133 | 0.237 | 0.127 | 0.138 | 0.123 | 0.127 | 0.137 | 0.124 | 0.137 | 0.278 | 0.135 | 0.188 | 0.138 | 0.132 | 0.141 | 0.134 | 0.149 | **0.150** |
| **TPSSVWN** | 0.230 | 0.233 | 0.259 | 0.260 | 0.267 | 0.260 | 0.251 | 0.234 | 0.260 | 0.249 | 0.233 | 0.266 | 0.276 | 0.269 | 0.221 | 0.296 | 0.232 | 0.271 | 0.260 | 0.257 | **0.254** |
| **TPSSVWN5** | 0.246 | 0.248 | 0.294 | 0.294 | 0.281 | 0.294 | 0.278 | 0.263 | 0.294 | 0.277 | 0.263 | 0.298 | 0.273 | 0.301 | 0.244 | 0.333 | 0.258 | 0.308 | 0.294 | 0.292 | **0.282** |
| **wPBEhB95** | 0.119 | 0.120 | 0.108 | 0.108 | 0.213 | **0.049** | **0.099** | **0.082** | **0.049** | 0.100 | **0.082** | **0.066** | 0.316 | **0.037** | 0.188 | **0.084** | 0.114 | 0.105 | **0.097** | 0.110 | **0.112** |
| **wPBEhBRC** | 0.184 | 0.187 | 0.159 | 0.159 | 0.288 | 0.162 | 0.157 | 0.163 | 0.161 | 0.157 | 0.163 | 0.170 | 0.325 | 0.153 | 0.235 | 0.164 | 0.180 | 0.162 | 0.160 | 0.161 | **0.183** |
| **wPBEhKCIS** | 0.178 | 0.183 | 0.131 | 0.131 | 0.272 | 0.131 | 0.133 | 0.140 | 0.130 | 0.133 | 0.142 | 0.140 | 0.321 | 0.123 | 0.224 | 0.127 | 0.171 | 0.129 | 0.133 | 0.130 | **0.160** |
| **wPBEhLYP** | 0.314 | 0.315 | 0.283 | 0.283 | 0.369 | 0.277 | 0.284 | 0.285 | 0.278 | 0.285 | 0.286 | 0.279 | 0.444 | 0.283 | 0.336 | 0.274 | 0.302 | 0.285 | 0.284 | 0.283 | **0.301** |
| **wPBEhP86** | 0.221 | 0.224 | 0.174 | 0.174 | 0.278 | 0.167 | 0.182 | 0.189 | 0.168 | 0.184 | 0.191 | 0.172 | 0.363 | 0.164 | 0.247 | 0.176 | 0.202 | 0.181 | 0.180 | 0.178 | **0.201** |
| **wPBEhPBE** | 0.159 | 0.164 | 0.141 | 0.141 | 0.262 | 0.141 | 0.132 | 0.128 | 0.139 | 0.132 | 0.128 | 0.151 | 0.305 | 0.133 | 0.211 | 0.142 | 0.158 | 0.144 | 0.142 | 0.143 | **0.160** |
| **wPBEhPKZB** | 0.159 | 0.163 | 0.145 | 0.145 | 0.262 | 0.144 | 0.134 | 0.130 | 0.143 | 0.135 | 0.130 | 0.155 | 0.304 | 0.138 | 0.211 | 0.148 | 0.158 | 0.149 | 0.147 | 0.147 | **0.162** |
| **wPBEhPL** | 0.184 | 0.187 | 0.190 | 0.189 | 0.277 | 0.186 | 0.178 | 0.171 | 0.186 | 0.178 | 0.171 | 0.192 | 0.312 | 0.185 | 0.222 | 0.203 | 0.181 | 0.198 | 0.190 | 0.189 | **0.198** |
| **wPBEhPW91** | 0.163 | 0.167 | 0.140 | 0.140 | 0.263 | 0.136 | 0.134 | 0.134 | 0.135 | 0.134 | 0.134 | 0.147 | 0.307 | 0.131 | 0.212 | 0.136 | 0.160 | 0.139 | 0.141 | 0.141 | **0.160** |
| **wPBEhRevTPSS** | 0.165 | 0.169 | 0.135 | 0.135 | 0.261 | 0.136 | 0.131 | 0.135 | 0.136 | 0.131 | 0.135 | 0.147 | 0.307 | 0.127 | 0.212 | 0.135 | 0.161 | 0.137 | 0.137 | 0.138 | **0.158** |
| **wPBEhTPSS** | 0.159 | 0.163 | 0.144 | 0.144 | 0.262 | 0.143 | 0.134 | 0.129 | 0.142 | 0.134 | 0.129 | 0.153 | 0.305 | 0.136 | 0.211 | 0.147 | 0.158 | 0.148 | 0.146 | 0.146 | **0.162** |
| **wPBEhVP86** | 0.220 | 0.223 | 0.174 | 0.174 | 0.278 | 0.167 | 0.181 | 0.188 | 0.167 | 0.182 | 0.189 | 0.171 | 0.361 | 0.163 | 0.245 | 0.175 | 0.201 | 0.180 | 0.179 | 0.177 | **0.200** |
| **wPBEhVWN** | 0.177 | 0.180 | 0.182 | 0.182 | 0.277 | 0.181 | 0.170 | 0.165 | 0.181 | 0.169 | 0.164 | 0.186 | 0.314 | 0.178 | 0.225 | 0.190 | 0.174 | 0.185 | 0.183 | 0.182 | **0.192** |
| **wPBEhVWN5** | 0.184 | 0.187 | 0.190 | 0.190 | 0.276 | 0.186 | 0.178 | 0.171 | 0.186 | 0.178 | 0.171 | 0.192 | 0.312 | 0.185 | 0.222 | 0.203 | 0.182 | 0.199 | 0.190 | 0.191 | **0.199** |
| **XaB95** | 0.679 | 0.680 | 0.679 | 0.679 | 0.692 | 0.646 | 0.683 | 0.688 | 0.650 | 0.686 | 0.691 | 0.610 | 0.755 | 0.675 | 0.683 | 0.684 | 0.682 | 0.683 | 0.682 | 0.684 | **0.680** |
| **XaKCIS** | 0.645 | 0.647 | 0.645 | 0.645 | 0.660 | 0.626 | 0.649 | 0.653 | 0.629 | 0.653 | 0.657 | 0.598 | 0.712 | 0.656 | 0.652 | 0.651 | 0.650 | 0.648 | 0.648 | 0.649 | **0.649** |
| **XaBRC** | 0.608 | 0.609 | 0.606 | 0.606 | 0.634 | 0.595 | 0.618 | 0.621 | 0.598 | 0.621 | 0.624 | 0.568 | 0.686 | 0.623 | 0.618 | 0.612 | 0.611 | 0.609 | 0.610 | 0.612 | **0.614** |
| **XaLYP** | 0.739 | 0.741 | 0.740 | 0.740 | 0.751 | 0.721 | 0.739 | 0.742 | 0.724 | 0.742 | 0.744 | 0.697 | 0.787 | 0.737 | 0.742 | 0.740 | 0.743 | 0.741 | 0.741 | 0.741 | **0.740** |

| | | | | | | | | | | | | | | | | | | | | | |
|---|---|---|---|---|---|---|---|---|---|---|---|---|---|---|---|---|---|---|---|---|---|
| **XaP86** | 0.735 | 0.738 | 0.744 | 0.744 | 0.746 | 0.724 | 0.747 | 0.750 | 0.728 | 0.751 | 0.754 | 0.695 | 0.781 | 0.749 | 0.740 | 0.748 | 0.745 | 0.746 | 0.747 | 0.748 | **0.743** |
| **XaPBE** | 0.649 | 0.651 | 0.654 | 0.654 | 0.666 | 0.635 | 0.662 | 0.666 | 0.640 | 0.666 | 0.670 | 0.604 | 0.719 | 0.672 | 0.658 | 0.661 | 0.657 | 0.656 | 0.658 | 0.659 | **0.658** |
| **XaPKZB** | 0.635 | 0.638 | 0.638 | 0.638 | 0.653 | 0.622 | 0.647 | 0.651 | 0.626 | 0.651 | 0.655 | 0.592 | 0.706 | 0.657 | 0.644 | 0.646 | 0.641 | 0.640 | 0.642 | 0.643 | **0.643** |
| **XaPL** | 0.533 | 0.534 | 0.521 | 0.521 | 0.552 | 0.507 | 0.530 | 0.530 | 0.511 | 0.534 | 0.534 | 0.482 | 0.615 | 0.536 | 0.541 | 0.529 | 0.528 | 0.525 | 0.524 | 0.524 | **0.531** |
| **XaPW91** | 0.656 | 0.659 | 0.662 | 0.662 | 0.672 | 0.643 | 0.669 | 0.672 | 0.647 | 0.673 | 0.676 | 0.612 | 0.723 | 0.678 | 0.665 | 0.668 | 0.664 | 0.664 | 0.665 | 0.666 | **0.665** |
| **XaRevTPSS** | 0.647 | 0.649 | 0.650 | 0.650 | 0.663 | 0.634 | 0.658 | 0.661 | 0.637 | 0.662 | 0.665 | 0.604 | 0.714 | 0.666 | 0.655 | 0.657 | 0.653 | 0.653 | 0.653 | 0.654 | **0.654** |
| **XaTPSS** | 0.638 | 0.640 | 0.641 | 0.641 | 0.655 | 0.624 | 0.649 | 0.653 | 0.628 | 0.654 | 0.657 | 0.594 | 0.708 | 0.659 | 0.647 | 0.648 | 0.644 | 0.643 | 0.645 | 0.645 | **0.646** |
| **XaVP86** | 0.685 | 0.688 | 0.689 | 0.689 | 0.746 | 0.671 | 0.696 | 0.698 | 0.728 | 0.699 | 0.700 | 0.695 | 0.741 | 0.704 | 0.689 | 0.696 | 0.692 | 0.693 | 0.694 | 0.695 | **0.699** |
| **XaVWN** | 0.551 | 0.552 | 0.542 | 0.542 | 0.568 | 0.489 | 0.508 | 0.551 | 0.492 | 0.512 | 0.555 | 0.464 | 0.628 | 0.538 | 0.558 | 0.550 | 0.548 | 0.545 | 0.544 | 0.545 | **0.539** |
| **XaVWN5** | 0.532 | 0.534 | 0.495 | 0.495 | 0.551 | 0.471 | 0.491 | 0.530 | 0.474 | 0.495 | 0.534 | 0.446 | 0.615 | 0.521 | 0.540 | 0.529 | 0.499 | 0.524 | 0.523 | 0.523 | **0.516** |
| **LC-BB95** | **0.070** | **0.060** | **0.099** | **0.099** | 0.158 | **0.086** | **0.086** | **0.084** | **0.086** | **0.086** | **0.085** | **0.085** | 0.229 | 0.107 | 0.111 | **0.086** | **0.096** | **0.097** | **0.099** | 0.101 | **0.100** |
| **LC-BBRC** | 0.120 | 0.124 | 0.361 | 0.361 | 0.265 | 0.324 | 0.251 | 0.223 | 0.329 | 0.249 | 0.222 | 0.348 | 0.304 | 0.316 | 0.233 | 0.268 | 0.242 | 0.378 | 0.360 | 0.437 | **0.286** |
| **LC-BKCIS** | **0.074** | **0.081** | 0.184 | 0.184 | 0.224 | 0.199 | 0.135 | 0.114 | 0.198 | 0.133 | 0.113 | 0.189 | 0.280 | 0.169 | 0.177 | 0.139 | 0.139 | 0.183 | 0.181 | 0.206 | **0.165** |
| **LC-BLYP** | 0.347 | 0.348 | 0.302 | 0.301 | 0.405 | 0.309 | 0.320 | 0.318 | 0.310 | 0.322 | 0.320 | 0.303 | 0.499 | 0.319 | 0.361 | 0.306 | 0.317 | 0.300 | 0.303 | 0.301 | **0.331** |
| **LC-BP86** | 0.173 | 0.174 | 0.463 | 0.459 | 0.238 | 0.205 | 0.160 | 0.167 | 0.204 | 0.162 | 0.176 | 0.199 | 0.364 | 0.326 | 0.289 | 0.207 | 0.388 | 0.419 | 0.467 | 0.493 | **0.287** |
| **LC-BPBE** | 0.146 | 0.142 | 0.571 | 0.570 | 0.285 | 0.488 | 0.458 | 0.378 | 0.484 | 0.458 | 0.379 | 0.485 | 0.237 | 0.435 | 0.265 | 0.456 | 0.345 | 0.543 | 0.572 | 0.588 | **0.414** |
| **LC-BPKZB** | 0.154 | 0.150 | 0.568 | 0.571 | 0.293 | 0.486 | 0.460 | 0.381 | 0.482 | 0.458 | 0.382 | 0.497 | 0.226 | 0.445 | 0.289 | 0.487 | 0.346 | 0.542 | 0.573 | 0.590 | **0.419** |
| **LC-BPL** | 0.209 | 0.211 | 0.494 | 0.494 | 0.295 | 0.457 | 0.397 | 0.354 | 0.457 | 0.389 | 0.353 | 0.456 | 0.272 | 0.458 | 0.269 | 0.480 | 0.329 | 0.514 | 0.522 | 0.558 | **0.398** |
| **LC-BPW91** | 0.129 | 0.124 | 0.563 | 0.563 | 0.258 | 0.476 | 0.409 | 0.328 | 0.470 | 0.392 | 0.314 | 0.472 | 0.237 | 0.418 | 0.248 | 0.410 | 0.332 | 0.539 | 0.591 | 0.581 | **0.393** |
| **LC-BRevTPSS** | 0.103 | 0.100 | 0.517 | 0.519 | 0.230 | 0.444 | 0.304 | 0.250 | 0.437 | 0.308 | 0.243 | 0.437 | 0.236 | 0.390 | 0.226 | 0.392 | 0.310 | 0.463 | 0.509 | 0.543 | **0.348** |
| **LC-BTPSS** | 0.151 | 0.146 | 0.571 | 0.570 | 0.299 | 0.486 | 0.458 | 0.383 | 0.482 | 0.458 | 0.382 | 0.490 | 0.240 | 0.440 | 0.278 | 0.484 | 0.346 | 0.544 | 0.573 | 0.601 | **0.419** |
| **LC-BVP86** | 0.170 | 0.171 | 0.474 | 0.470 | 0.234 | 0.206 | 0.157 | 0.168 | 0.201 | 0.163 | 0.170 | 0.212 | 0.363 | 0.326 | 0.294 | 0.205 | 0.385 | 0.402 | 0.467 | 0.484 | **0.286** |
| **LC-BVWN** | 0.170 | 0.178 | 0.381 | 0.381 | 0.263 | 0.354 | 0.294 | 0.268 | 0.353 | 0.293 | 0.268 | 0.355 | 0.275 | 0.358 | 0.238 | 0.341 | 0.268 | 0.402 | 0.385 | 0.413 | **0.312** |
| **LC-BVWN5** | 0.211 | 0.213 | 0.509 | 0.509 | 0.297 | 0.471 | 0.403 | 0.364 | 0.470 | 0.402 | 0.364 | 0.466 | 0.273 | 0.461 | 0.270 | 0.504 | 0.343 | 0.551 | 0.526 | 0.604 | **0.410** |
| **LC-BRxB95** | **0.061** | **0.061** | 0.101 | 0.101 | 0.158 | **0.088** | **0.088** | **0.086** | **0.088** | **0.088** | **0.086** | **0.087** | 0.228 | 0.107 | 0.112 | **0.088** | **0.098** | **0.098** | 0.101 | 0.102 | **0.101** |
| **LC-BRxBRC** | 0.115 | 0.119 | 0.367 | 0.367 | 0.265 | 0.295 | 0.227 | 0.218 | 0.299 | 0.227 | 0.217 | 0.302 | 0.302 | 0.273 | 0.228 | 0.240 | 0.245 | 0.368 | 0.361 | 0.433 | **0.273** |
| **LC-BRxKCIS** | **0.073** | **0.079** | 0.181 | 0.181 | 0.221 | 0.144 | 0.119 | **0.096** | 0.143 | 0.119 | **0.095** | 0.148 | 0.280 | 0.139 | 0.170 | 0.105 | 0.128 | 0.175 | 0.176 | 0.186 | **0.148** |
| **LC-BRxLYP** | 0.360 | 0.360 | 0.319 | 0.319 | 0.414 | 0.327 | 0.342 | 0.339 | 0.329 | 0.343 | 0.341 | 0.322 | 0.504 | 0.340 | 0.373 | 0.329 | 0.333 | 0.320 | 0.321 | 0.320 | **0.348** |
| **LC-BRxP86** | 0.187 | 0.189 | 0.475 | 0.475 | 0.252 | 0.210 | 0.236 | 0.235 | 0.211 | 0.223 | 0.237 | 0.201 | 0.371 | 0.339 | 0.316 | 0.227 | 0.406 | 0.402 | 0.483 | 0.520 | **0.310** |
| **LC-BRxPBE** | 0.143 | 0.139 | 0.579 | 0.578 | 0.284 | 0.487 | 0.364 | 0.326 | 0.475 | 0.358 | 0.307 | 0.478 | 0.228 | 0.430 | 0.250 | 0.409 | 0.347 | 0.523 | 0.574 | 0.605 | **0.394** |
| **LC-BRxPKZB** | 0.160 | 0.146 | 0.563 | 0.562 | 0.285 | 0.489 | 0.392 | 0.325 | 0.477 | 0.390 | 0.323 | 0.491 | 0.218 | 0.440 | 0.263 | 0.478 | 0.350 | 0.525 | 0.577 | 0.592 | **0.402** |
| **LC-BRxPL** | 0.211 | 0.212 | 0.502 | 0.502 | 0.283 | 0.448 | 0.366 | 0.324 | 0.448 | 0.367 | 0.323 | 0.445 | 0.270 | 0.479 | 0.268 | 0.441 | 0.355 | 0.521 | 0.544 | 0.540 | **0.392** |
| **LC-BRxPW91** | 0.127 | 0.120 | 0.540 | 0.540 | 0.261 | 0.447 | 0.328 | 0.245 | 0.443 | 0.323 | 0.244 | 0.461 | 0.227 | 0.403 | 0.231 | 0.377 | 0.338 | 0.513 | 0.559 | 0.562 | **0.364** |
| **LC-BRxRevTPSS** | **0.098** | **0.095** | 0.489 | 0.490 | 0.228 | 0.370 | 0.269 | 0.217 | 0.368 | 0.264 | 0.216 | 0.388 | 0.228 | 0.353 | 0.219 | 0.302 | 0.309 | 0.459 | 0.500 | 0.545 | **0.320** |
| **LC-BRxTPSS** | 0.148 | 0.143 | 0.577 | 0.579 | 0.286 | 0.489 | 0.392 | 0.329 | 0.478 | 0.388 | 0.308 | 0.493 | 0.218 | 0.440 | 0.251 | 0.451 | 0.348 | 0.539 | 0.580 | 0.591 | **0.401** |
| **LC-BRxVP86** | 0.185 | 0.186 | 0.469 | 0.464 | 0.248 | 0.211 | 0.208 | 0.223 | 0.210 | 0.218 | 0.229 | 0.197 | 0.363 | 0.343 | 0.313 | 0.223 | 0.396 | 0.433 | 0.479 | 0.509 | **0.305** |
| **LC-BRxVWN** | 0.169 | 0.179 | 0.377 | 0.377 | 0.262 | 0.335 | 0.268 | 0.247 | 0.334 | 0.267 | 0.247 | 0.338 | 0.271 | 0.343 | 0.223 | 0.314 | 0.269 | 0.373 | 0.374 | 0.418 | **0.299** |
| **LC-BRxVWN5** | 0.214 | 0.216 | 0.504 | 0.504 | 0.286 | 0.450 | 0.370 | 0.328 | 0.450 | 0.369 | 0.329 | 0.448 | 0.269 | 0.481 | 0.268 | 0.446 | 0.335 | 0.528 | 0.529 | 0.560 | **0.394** |
| **LC-G96B95** | **0.070** | **0.070** | **0.099** | **0.099** | 0.158 | **0.086** | **0.086** | **0.085** | **0.086** | **0.086** | **0.085** | **0.085** | 0.229 | 0.107 | 0.111 | **0.086** | **0.096** | **0.097** | 0.100 | 0.101 | **0.101** |
| **LC-G96BRC** | 0.120 | 0.124 | 0.366 | 0.366 | 0.264 | 0.336 | 0.251 | 0.229 | 0.334 | 0.250 | 0.226 | 0.338 | 0.303 | 0.320 | 0.232 | 0.272 | 0.243 | 0.384 | 0.375 | 0.439 | **0.289** |
| **LC-G96KCIS** | **0.076** | **0.083** | 0.190 | 0.190 | 0.222 | 0.200 | 0.135 | 0.114 | 0.199 | 0.133 | 0.113 | 0.191 | 0.279 | 0.171 | 0.176 | 0.140 | 0.147 | 0.184 | 0.187 | 0.221 | **0.168** |
| **LC-G96LYP** | 0.347 | 0.347 | 0.301 | 0.301 | 0.405 | 0.309 | 0.320 | 0.318 | 0.310 | 0.322 | 0.319 | 0.303 | 0.498 | 0.319 | 0.361 | 0.307 | 0.317 | 0.301 | 0.302 | 0.300 | **0.330** |
| **LC-G96P86** | 0.170 | 0.172 | 0.463 | 0.462 | 0.233 | 0.206 | 0.161 | 0.168 | 0.205 | 0.163 | 0.174 | 0.214 | 0.363 | 0.327 | 0.293 | 0.205 | 0.385 | 0.365 | 0.463 | 0.486 | **0.284** |
| **LC-G96PBE** | 0.154 | 0.141 | 0.571 | 0.571 | 0.284 | 0.485 | 0.458 | 0.379 | 0.499 | 0.457 | 0.374 | 0.482 | 0.237 | 0.438 | 0.272 | 0.469 | 0.346 | 0.543 | 0.572 | 0.590 | **0.416** |
| **LC-G96PKZB** | 0.157 | 0.149 | 0.571 | 0.571 | 0.304 | 0.497 | 0.459 | 0.381 | 0.487 | 0.460 | 0.381 | 0.486 | 0.224 | 0.448 | 0.289 | 0.475 | 0.349 | 0.544 | 0.573 | 0.588 | **0.420** |
| **LC-G96PL** | 0.210 | 0.211 | 0.495 | 0.495 | 0.293 | 0.457 | 0.395 | 0.353 | 0.456 | 0.393 | 0.352 | 0.460 | 0.273 | 0.459 | 0.267 | 0.480 | 0.330 | 0.514 | 0.507 | 0.526 | **0.396** |
| **LC-G96PW91** | 0.131 | 0.124 | 0.565 | 0.564 | 0.274 | 0.474 | 0.419 | 0.328 | 0.471 | 0.416 | 0.336 | 0.471 | 0.235 | 0.421 | 0.240 | 0.444 | 0.336 | 0.536 | 0.575 | 0.586 | **0.397** |
| **LC-G96RevTPSS** | 0.105 | 0.101 | 0.521 | 0.523 | 0.244 | 0.441 | 0.322 | 0.263 | 0.439 | 0.304 | 0.261 | 0.418 | 0.234 | 0.394 | 0.227 | 0.389 | 0.312 | 0.465 | 0.512 | 0.546 | **0.351** |
| **LC-G96TPSS** | 0.160 | 0.149 | 0.572 | 0.571 | 0.292 | 0.487 | 0.475 | 0.381 | 0.482 | 0.459 | 0.383 | 0.487 | 0.238 | 0.441 | 0.286 | 0.476 | 0.356 | 0.544 | 0.574 | 0.616 | **0.421** |
| **LC-G96VP86** | 0.166 | 0.168 | 0.474 | 0.474 | 0.232 | 0.207 | 0.155 | 0.175 | 0.202 | 0.156 | 0.168 | 0.211 | 0.362 | 0.329 | 0.291 | 0.203 | 0.381 | 0.399 | 0.464 | 0.486 | **0.285** |
| **LC-G96VWN** | 0.171 | 0.178 | 0.384 | 0.384 | 0.262 | 0.355 | 0.294 | 0.268 | 0.353 | 0.293 | 0.268 | 0.354 | 0.274 | 0.358 | 0.237 | 0.342 | 0.268 | 0.406 | 0.386 | 0.416 | **0.312** |
| **LC-G96VWN5** | 0.212 | 0.214 | 0.509 | 0.509 | 0.299 | 0.468 | 0.405 | 0.360 | 0.469 | 0.402 | 0.359 | 0.466 | 0.271 | 0.461 | 0.269 | 0.503 | 0.344 | 0.550 | 0.527 | 0.574 | **0.409** |
| **LC-LGB95** | **0.060** | **0.060** | **0.099** | **0.099** | 0.159 | **0.086** | **0.086** | **0.084** | **0.086** | **0.086** | **0.084** | **0.085** | 0.230 | 0.107 | 0.111 | **0.085** | **0.095** | **0.096** | **0.099** | 0.100 | **0.100** |
| **LC-LGBRC** | 0.118 | 0.122 | 0.362 | 0.363 | 0.267 | 0.325 | 0.255 | 0.224 | 0.321 | 0.283 | 0.223 | 0.327 | 0.306 | 0.308 | 0.238 | 0.270 | 0.242 | 0.371 | 0.358 | 0.432 | **0.286** |
| **LC-LGKCIS** | **0.075** | **0.082** | 0.186 | 0.186 | 0.226 | 0.198 | 0.135 | 0.114 | 0.196 | 0.134 | 0.114 | 0.190 | 0.284 | 0.155 | 0.179 | 0.126 | 0.132 | 0.179 | 0.182 | 0.204 | **0.164** |
| **LC-LGLYP** | 0.348 | 0.349 | 0.302 | 0.302 | 0.407 | 0.309 | 0.320 | 0.318 | 0.311 | 0.322 | 0.320 | 0.305 | 0.500 | 0.320 | 0.362 | 0.307 | 0.318 | 0.302 | 0.302 | 0.302 | **0.331** |
| **LC-LGP86** | 0.177 | 0.178 | 0.467 | 0.469 | 0.244 | 0.215 | 0.157 | 0.171 | 0.205 | 0.171 | 0.173 | 0.205 | 0.367 | 0.327 | 0.300 | 0.209 | 0.323 | 0.376 | 0.465 | 0.497 | **0.285** |

| | | | | | | | | | | | | | | | | | | | | | |
|---|---|---|---|---|---|---|---|---|---|---|---|---|---|---|---|---|---|---|---|---|---|
| **LC-LGPBE** | 0.143 | 0.140 | 0.561 | 0.561 | 0.288 | 0.491 | 0.457 | 0.378 | 0.488 | 0.458 | 0.378 | 0.490 | 0.240 | 0.462 | 0.269 | 0.486 | 0.342 | 0.563 | 0.571 | 0.590 | **0.418** |
| **LC-LGPKZB** | 0.149 | 0.145 | 0.562 | 0.563 | 0.281 | 0.484 | 0.459 | 0.387 | 0.491 | 0.460 | 0.386 | 0.487 | 0.229 | 0.492 | 0.291 | 0.490 | 0.345 | 0.563 | 0.574 | 0.594 | **0.421** |
| **LC-LGPL** | 0.208 | 0.209 | 0.509 | 0.509 | 0.289 | 0.470 | 0.398 | 0.360 | 0.470 | 0.398 | 0.382 | 0.462 | 0.275 | 0.485 | 0.272 | 0.484 | 0.330 | 0.532 | 0.527 | 0.548 | **0.406** |
| **LC-LGPW91** | 0.127 | 0.120 | 0.558 | 0.555 | 0.260 | 0.476 | 0.414 | 0.324 | 0.474 | 0.414 | 0.326 | 0.479 | 0.238 | 0.448 | 0.252 | 0.423 | 0.330 | 0.534 | 0.547 | 0.582 | **0.394** |
| **LC-LGRevTPSS** | 0.100 | **0.097** | 0.510 | 0.508 | 0.234 | 0.441 | 0.333 | 0.248 | 0.415 | 0.334 | 0.251 | 0.441 | 0.238 | 0.409 | 0.230 | 0.380 | 0.307 | 0.465 | 0.509 | 0.551 | **0.350** |
| **LC-LGTPSS** | 0.145 | 0.145 | 0.563 | 0.563 | 0.286 | 0.486 | 0.459 | 0.385 | 0.482 | 0.459 | 0.386 | 0.483 | 0.243 | 0.469 | 0.281 | 0.491 | 0.343 | 0.564 | 0.568 | 0.592 | **0.420** |
| **LC-LGVP86** | 0.175 | 0.175 | 0.477 | 0.474 | 0.241 | 0.214 | 0.158 | 0.168 | 0.216 | 0.158 | 0.171 | 0.209 | 0.367 | 0.301 | 0.298 | 0.207 | 0.397 | 0.423 | 0.473 | 0.495 | **0.290** |
| **LC-LGVWN** | 0.169 | 0.179 | 0.391 | 0.391 | 0.266 | 0.356 | 0.298 | 0.272 | 0.354 | 0.298 | 0.272 | 0.363 | 0.277 | 0.356 | 0.234 | 0.341 | 0.268 | 0.401 | 0.388 | 0.417 | **0.315** |
| **LC-LGVWN5** | 0.211 | 0.212 | 0.524 | 0.524 | 0.295 | 0.479 | 0.408 | 0.370 | 0.478 | 0.409 | 0.366 | 0.471 | 0.275 | 0.490 | 0.274 | 0.512 | 0.337 | 0.552 | 0.532 | 0.567 | **0.414** |
| **LC-mPWB95** | **0.060** | **0.060** | **0.099** | **0.099** | 0.158 | **0.086** | **0.086** | **0.085** | **0.086** | **0.086** | **0.085** | **0.085** | 0.229 | 0.107 | 0.111 | **0.086** | **0.096** | **0.097** | **0.099** | 0.101 | **0.100** |
| **LC-mPWBRC** | 0.119 | 0.123 | 0.360 | 0.360 | 0.266 | 0.329 | 0.249 | 0.222 | 0.317 | 0.249 | 0.221 | 0.332 | 0.304 | 0.303 | 0.232 | 0.268 | 0.241 | 0.377 | 0.360 | 0.435 | **0.283** |
| **LC-mPWKCIS** | **0.074** | **0.081** | 0.184 | 0.184 | 0.224 | 0.198 | 0.132 | 0.112 | 0.196 | 0.132 | 0.111 | 0.188 | 0.281 | 0.155 | 0.177 | 0.137 | 0.139 | 0.182 | 0.180 | 0.204 | **0.164** |
| **LC-mPWLYP** | 0.348 | 0.349 | 0.303 | 0.303 | 0.406 | 0.310 | 0.321 | 0.319 | 0.311 | 0.323 | 0.321 | 0.304 | 0.499 | 0.320 | 0.362 | 0.308 | 0.318 | 0.301 | 0.304 | 0.302 | **0.332** |
| **LC-mPWP86** | 0.174 | 0.175 | 0.463 | 0.459 | 0.240 | 0.204 | 0.162 | 0.171 | 0.210 | 0.162 | 0.177 | 0.199 | 0.365 | 0.329 | 0.290 | 0.209 | 0.389 | 0.419 | 0.467 | 0.493 | **0.288** |
| **LC-mPWPBE** | 0.148 | 0.141 | 0.573 | 0.574 | 0.284 | 0.490 | 0.458 | 0.377 | 0.480 | 0.458 | 0.372 | 0.483 | 0.237 | 0.437 | 0.273 | 0.456 | 0.343 | 0.564 | 0.571 | 0.588 | **0.415** |
| **LC-mPWPKZB** | 0.153 | 0.148 | 0.588 | 0.571 | 0.292 | 0.496 | 0.475 | 0.382 | 0.485 | 0.455 | 0.382 | 0.489 | 0.226 | 0.447 | 0.279 | 0.474 | 0.347 | 0.566 | 0.571 | 0.590 | **0.421** |
| **LC-mPWPL** | 0.208 | 0.211 | 0.495 | 0.495 | 0.294 | 0.456 | 0.388 | 0.353 | 0.456 | 0.388 | 0.352 | 0.454 | 0.274 | 0.457 | 0.269 | 0.479 | 0.329 | 0.514 | 0.506 | 0.557 | **0.397** |
| **LC-mPWPW91** | 0.128 | 0.122 | 0.563 | 0.563 | 0.260 | 0.471 | 0.399 | 0.326 | 0.470 | 0.391 | 0.341 | 0.485 | 0.237 | 0.419 | 0.254 | 0.401 | 0.332 | 0.537 | 0.553 | 0.580 | **0.392** |
| **LC-mPWRevTPS** | 0.103 | **0.099** | 0.518 | 0.518 | 0.231 | 0.438 | 0.302 | 0.242 | 0.433 | 0.320 | 0.249 | 0.416 | 0.236 | 0.412 | 0.226 | 0.389 | 0.309 | 0.466 | 0.510 | 0.549 | **0.348** |
| **LC-mPWTPSS** | 0.147 | 0.146 | 0.588 | 0.589 | 0.302 | 0.490 | 0.458 | 0.382 | 0.483 | 0.473 | 0.381 | 0.490 | 0.239 | 0.443 | 0.273 | 0.475 | 0.345 | 0.563 | 0.590 | 0.591 | **0.422** |
| **LC-mPWVP86** | 0.171 | 0.172 | 0.470 | 0.474 | 0.235 | 0.201 | 0.163 | 0.171 | 0.201 | 0.163 | 0.167 | 0.203 | 0.364 | 0.325 | 0.295 | 0.206 | 0.385 | 0.402 | 0.466 | 0.490 | **0.286** |
| **LC-mPWVWN** | 0.171 | 0.177 | 0.380 | 0.381 | 0.263 | 0.353 | 0.293 | 0.267 | 0.352 | 0.293 | 0.267 | 0.354 | 0.275 | 0.356 | 0.238 | 0.340 | 0.267 | 0.396 | 0.384 | 0.412 | **0.311** |
| **LC-mPWVWN5** | 0.211 | 0.212 | 0.508 | 0.508 | 0.297 | 0.470 | 0.402 | 0.361 | 0.469 | 0.401 | 0.359 | 0.465 | 0.273 | 0.460 | 0.270 | 0.504 | 0.338 | 0.550 | 0.527 | 0.579 | **0.408** |
| **LC-OB95** | **0.082** | **0.082** | **0.099** | **0.099** | 0.172 | **0.086** | **0.086** | **0.084** | **0.086** | **0.086** | **0.084** | **0.085** | 0.229 | 0.106 | 0.110 | **0.086** | **0.097** | **0.097** | **0.099** | 0.100 | **0.103** |
| **LC-OBRC** | 0.124 | 0.129 | 0.357 | 0.357 | 0.267 | 0.352 | 0.254 | 0.230 | 0.350 | 0.253 | 0.229 | 0.353 | 0.302 | 0.321 | 0.235 | 0.281 | 0.239 | 0.380 | 0.358 | 0.434 | **0.290** |
| **LC-OKCIS** | **0.082** | **0.088** | 0.185 | 0.185 | 0.223 | 0.201 | 0.136 | 0.116 | 0.200 | 0.135 | 0.115 | 0.193 | 0.278 | 0.178 | 0.178 | 0.147 | 0.137 | 0.182 | 0.186 | 0.202 | **0.167** |
| **LC-OLYP** | 0.345 | 0.346 | 0.300 | 0.301 | 0.404 | 0.307 | 0.318 | 0.317 | 0.309 | 0.321 | 0.318 | 0.302 | 0.495 | 0.322 | 0.359 | 0.303 | 0.317 | 0.300 | 0.301 | 0.300 | **0.329** |
| **LC-OP86** | 0.163 | 0.165 | 0.461 | 0.457 | 0.229 | 0.206 | 0.159 | 0.179 | 0.202 | 0.165 | 0.166 | 0.214 | 0.360 | 0.330 | 0.287 | 0.198 | 0.303 | 0.399 | 0.468 | 0.486 | **0.280** |
| **LC-OPBE** | 0.161 | 0.148 | 0.570 | 0.570 | 0.304 | 0.519 | 0.460 | 0.378 | 0.501 | 0.461 | 0.377 | 0.499 | 0.235 | 0.472 | 0.268 | 0.487 | 0.345 | 0.560 | 0.573 | 0.589 | **0.424** |
| **LC-OPKZB** | 0.171 | 0.168 | 0.571 | 0.571 | 0.305 | 0.504 | 0.463 | 0.383 | 0.500 | 0.462 | 0.385 | 0.503 | 0.223 | 0.480 | 0.290 | 0.488 | 0.345 | 0.541 | 0.574 | 0.592 | **0.426** |
| **LC-OPL** | 0.209 | 0.210 | 0.491 | 0.491 | 0.293 | 0.464 | 0.400 | 0.356 | 0.464 | 0.399 | 0.354 | 0.460 | 0.272 | 0.463 | 0.269 | 0.487 | 0.326 | 0.509 | 0.503 | 0.522 | **0.397** |
| **LC-OPW91** | 0.134 | 0.130 | 0.545 | 0.560 | 0.259 | 0.489 | 0.437 | 0.346 | 0.488 | 0.424 | 0.326 | 0.491 | 0.234 | 0.427 | 0.257 | 0.443 | 0.332 | 0.536 | 0.577 | 0.571 | **0.400** |
| **LC-ORevTPSS** | 0.107 | 0.104 | 0.481 | 0.481 | 0.233 | 0.479 | 0.337 | 0.307 | 0.480 | 0.336 | 0.286 | 0.481 | 0.232 | 0.389 | 0.225 | 0.388 | 0.308 | 0.464 | 0.486 | 0.550 | **0.358** |
| **LC-OTPSS** | 0.158 | 0.150 | 0.572 | 0.572 | 0.299 | 0.501 | 0.463 | 0.387 | 0.503 | 0.461 | 0.385 | 0.503 | 0.237 | 0.480 | 0.290 | 0.491 | 0.352 | 0.542 | 0.578 | 0.591 | **0.426** |
| **LC-OVP86** | 0.161 | 0.162 | 0.464 | 0.460 | 0.227 | 0.220 | 0.151 | 0.161 | 0.210 | 0.162 | 0.162 | 0.210 | 0.359 | 0.342 | 0.285 | 0.196 | 0.379 | 0.396 | 0.486 | 0.518 | **0.286** |
| **LC-OVWN** | 0.170 | 0.178 | 0.377 | 0.377 | 0.262 | 0.353 | 0.294 | 0.267 | 0.352 | 0.293 | 0.266 | 0.355 | 0.273 | 0.362 | 0.239 | 0.349 | 0.265 | 0.391 | 0.408 | 0.410 | **0.312** |
| **LC-OVWN5** | 0.211 | 0.212 | 0.497 | 0.497 | 0.298 | 0.463 | 0.412 | 0.362 | 0.463 | 0.407 | 0.359 | 0.465 | 0.270 | 0.465 | 0.270 | 0.510 | 0.329 | 0.545 | 0.508 | 0.541 | **0.404** |
| **LC-PBEB95** | **0.060** | **0.060** | **0.099** | **0.099** | 0.159 | **0.086** | **0.086** | **0.084** | **0.086** | **0.086** | **0.084** | **0.085** | 0.230 | 0.107 | 0.111 | **0.086** | **0.096** | **0.096** | **0.099** | 0.100 | **0.100** |
| **LC-PBEBRC** | 0.119 | 0.124 | 0.360 | 0.360 | 0.265 | 0.319 | 0.251 | 0.223 | 0.328 | 0.248 | 0.221 | 0.332 | 0.305 | 0.312 | 0.234 | 0.269 | 0.240 | 0.375 | 0.357 | 0.434 | **0.284** |
| **LC-PBEKCIS** | **0.075** | **0.082** | 0.183 | 0.183 | 0.224 | 0.197 | 0.134 | 0.113 | 0.195 | 0.133 | 0.112 | 0.188 | 0.282 | 0.154 | 0.178 | 0.127 | 0.137 | 0.181 | 0.180 | 0.205 | **0.163** |
| **LC-PBELYP** | 0.348 | 0.348 | 0.302 | 0.302 | 0.406 | 0.309 | 0.321 | 0.318 | 0.311 | 0.322 | 0.320 | 0.304 | 0.499 | 0.320 | 0.362 | 0.307 | 0.318 | 0.301 | 0.303 | 0.301 | **0.331** |
| **LC-PBEP86** | 0.175 | 0.176 | 0.461 | 0.461 | 0.241 | 0.222 | 0.160 | 0.172 | 0.214 | 0.161 | 0.174 | 0.215 | 0.366 | 0.325 | 0.297 | 0.213 | 0.393 | 0.421 | 0.468 | 0.495 | **0.290** |
| **LC-PBEPBE** | 0.150 | 0.140 | 0.572 | 0.569 | 0.285 | 0.484 | 0.459 | 0.378 | 0.498 | 0.457 | 0.377 | 0.495 | 0.238 | 0.436 | 0.265 | 0.457 | 0.342 | 0.562 | 0.570 | 0.589 | **0.416** |
| **LC-PBEPKZB** | 0.159 | 0.148 | 0.570 | 0.568 | 0.292 | 0.487 | 0.457 | 0.382 | 0.483 | 0.456 | 0.382 | 0.486 | 0.241 | 0.439 | 0.289 | 0.476 | 0.344 | 0.566 | 0.572 | 0.590 | **0.419** |
| **LC-PBEPL** | 0.207 | 0.209 | 0.494 | 0.494 | 0.289 | 0.463 | 0.389 | 0.354 | 0.457 | 0.389 | 0.353 | 0.455 | 0.274 | 0.457 | 0.269 | 0.480 | 0.328 | 0.529 | 0.523 | 0.558 | **0.399** |
| **LC-PBEPW91** | 0.127 | 0.119 | 0.545 | 0.544 | 0.258 | 0.472 | 0.393 | 0.311 | 0.471 | 0.392 | 0.319 | 0.485 | 0.237 | 0.420 | 0.253 | 0.402 | 0.330 | 0.535 | 0.554 | 0.580 | **0.387** |
| **LC-PBERevTPSS** | 0.102 | **0.098** | 0.498 | 0.494 | 0.232 | 0.434 | 0.321 | 0.251 | 0.434 | 0.301 | 0.237 | 0.435 | 0.237 | 0.392 | 0.227 | 0.389 | 0.307 | 0.463 | 0.506 | 0.546 | **0.345** |
| **LC-PBETPSS** | 0.149 | 0.145 | 0.570 | 0.571 | 0.286 | 0.485 | 0.457 | 0.383 | 0.483 | 0.458 | 0.382 | 0.487 | 0.241 | 0.440 | 0.288 | 0.485 | 0.344 | 0.565 | 0.572 | 0.595 | **0.419** |
| **LC-PBEVP86** | 0.172 | 0.174 | 0.474 | 0.474 | 0.238 | 0.200 | 0.160 | 0.167 | 0.203 | 0.161 | 0.169 | 0.199 | 0.332 | 0.326 | 0.296 | 0.207 | 0.389 | 0.420 | 0.465 | 0.493 | **0.286** |
| **LC-PBEVWN** | 0.169 | 0.176 | 0.383 | 0.383 | 0.263 | 0.353 | 0.294 | 0.268 | 0.353 | 0.293 | 0.268 | 0.355 | 0.276 | 0.356 | 0.239 | 0.337 | 0.267 | 0.395 | 0.383 | 0.413 | **0.311** |
| **LC-PBEVWN5** | 0.209 | 0.211 | 0.508 | 0.508 | 0.298 | 0.471 | 0.408 | 0.364 | 0.470 | 0.407 | 0.363 | 0.466 | 0.274 | 0.460 | 0.271 | 0.504 | 0.331 | 0.550 | 0.529 | 0.598 | **0.410** |
| **LC-PBEhB95** | **0.060** | **0.060** | **0.099** | **0.099** | 0.159 | **0.086** | **0.086** | **0.085** | **0.086** | **0.086** | **0.085** | **0.085** | 0.230 | 0.107 | 0.111 | **0.086** | **0.096** | **0.096** | **0.099** | 0.101 | **0.100** |
| **LC-PBEhBRC** | 0.119 | 0.124 | 0.360 | 0.360 | 0.266 | 0.324 | 0.250 | 0.226 | 0.318 | 0.247 | 0.221 | 0.327 | 0.305 | 0.312 | 0.233 | 0.267 | 0.241 | 0.364 | 0.358 | 0.434 | **0.283** |
| **LC-PBEhKCIS** | **0.075** | **0.081** | 0.183 | 0.183 | 0.224 | 0.197 | 0.135 | 0.114 | 0.196 | 0.133 | 0.113 | 0.188 | 0.281 | 0.155 | 0.178 | 0.126 | 0.135 | 0.176 | 0.177 | 0.205 | **0.163** |
| **LC-PBEhLYP** | 0.348 | 0.348 | 0.302 | 0.302 | 0.406 | 0.309 | 0.321 | 0.318 | 0.311 | 0.322 | 0.320 | 0.304 | 0.500 | 0.319 | 0.361 | 0.307 | 0.318 | 0.301 | 0.302 | 0.301 | **0.331** |
| **LC-PBEhP86** | 0.176 | 0.177 | 0.464 | 0.463 | 0.243 | 0.207 | 0.160 | 0.173 | 0.222 | 0.161 | 0.174 | 0.198 | 0.366 | 0.329 | 0.298 | 0.207 | 0.391 | 0.411 | 0.468 | 0.459 | **0.287** |
| **LC-PBEhPBE** | 0.152 | 0.142 | 0.568 | 0.569 | 0.284 | 0.490 | 0.457 | 0.376 | 0.507 | 0.456 | 0.374 | 0.488 | 0.239 | 0.462 | 0.257 | 0.468 | 0.343 | 0.566 | 0.587 | 0.607 | **0.420** |

| | | | | | | | | | | | | | | | | | | | | | |
|---|---|---|---|---|---|---|---|---|---|---|---|---|---|---|---|---|---|---|---|---|---|
| **LC-PBEhPKZB** | 0.157 | 0.147 | 0.565 | 0.563 | 0.290 | 0.489 | 0.459 | 0.382 | 0.490 | 0.457 | 0.379 | 0.490 | 0.228 | 0.469 | 0.279 | 0.476 | 0.347 | 0.565 | 0.572 | 0.594 | **0.420** |
| **LC-PBEhPL** | 0.207 | 0.208 | 0.505 | 0.505 | 0.294 | 0.478 | 0.397 | 0.360 | 0.478 | 0.393 | 0.357 | 0.456 | 0.274 | 0.458 | 0.269 | 0.477 | 0.328 | 0.532 | 0.523 | 0.547 | **0.402** |
| **LC-PBEhPW91** | 0.127 | 0.120 | 0.535 | 0.536 | 0.258 | 0.476 | 0.415 | 0.325 | 0.479 | 0.385 | 0.334 | 0.478 | 0.237 | 0.420 | 0.249 | 0.402 | 0.330 | 0.534 | 0.548 | 0.582 | **0.389** |
| **LC-PBEhRevTPS** | 0.101 | **0.098** | 0.518 | 0.519 | 0.232 | 0.438 | 0.301 | 0.238 | 0.436 | 0.305 | 0.248 | 0.438 | 0.237 | 0.373 | 0.228 | 0.380 | 0.303 | 0.466 | 0.509 | 0.552 | **0.346** |
| **LC-PBEhTPSS** | 0.156 | 0.144 | 0.571 | 0.573 | 0.285 | 0.491 | 0.459 | 0.382 | 0.489 | 0.458 | 0.383 | 0.484 | 0.242 | 0.440 | 0.271 | 0.474 | 0.346 | 0.565 | 0.568 | 0.611 | **0.420** |
| **LC-PBEhVP86** | 0.173 | 0.175 | 0.473 | 0.471 | 0.239 | 0.200 | 0.160 | 0.168 | 0.204 | 0.162 | 0.172 | 0.198 | 0.365 | 0.343 | 0.295 | 0.207 | 0.388 | 0.385 | 0.427 | 0.494 | **0.285** |
| **LC-PBEhVWN** | 0.169 | 0.176 | 0.387 | 0.387 | 0.264 | 0.353 | 0.293 | 0.268 | 0.352 | 0.293 | 0.268 | 0.354 | 0.276 | 0.356 | 0.239 | 0.338 | 0.267 | 0.401 | 0.383 | 0.417 | **0.312** |
| **LC-PBEhVWN5** | 0.210 | 0.211 | 0.510 | 0.510 | 0.298 | 0.489 | 0.413 | 0.368 | 0.488 | 0.405 | 0.365 | 0.467 | 0.274 | 0.460 | 0.271 | 0.502 | 0.332 | 0.551 | 0.530 | 0.563 | **0.411** |
| **LC-PKZBB95** | **0.080** | **0.081** | **0.098** | **0.098** | 0.157 | **0.085** | **0.085** | **0.084** | **0.085** | **0.085** | **0.084** | **0.085** | 0.227 | 0.105 | 0.110 | **0.085** | **0.095** | **0.096** | **0.099** | 0.100 | **0.101** |
| **LC-PKZBBRC** | 0.125 | 0.129 | 0.371 | 0.371 | 0.268 | 0.355 | 0.288 | 0.239 | 0.354 | 0.276 | 0.238 | 0.356 | 0.300 | 0.331 | 0.237 | 0.288 | 0.246 | 0.387 | 0.372 | 0.443 | **0.299** |
| **LC-PKZBKCIS** | **0.081** | **0.088** | 0.194 | 0.194 | 0.224 | 0.211 | 0.146 | 0.128 | 0.210 | 0.145 | 0.127 | 0.215 | 0.276 | 0.203 | 0.181 | 0.157 | 0.150 | 0.196 | 0.193 | 0.234 | **0.178** |
| **LC-PKZBLYP** | 0.334 | 0.334 | 0.289 | 0.289 | 0.395 | 0.297 | 0.305 | 0.306 | 0.298 | 0.307 | 0.308 | 0.292 | 0.489 | 0.308 | 0.352 | 0.293 | 0.307 | 0.289 | 0.290 | 0.288 | **0.318** |
| **LC-PKZBP86** | 0.157 | 0.159 | 0.457 | 0.455 | 0.226 | 0.221 | 0.150 | 0.157 | 0.220 | 0.151 | 0.159 | 0.235 | 0.355 | 0.337 | 0.276 | 0.191 | 0.377 | 0.394 | 0.447 | 0.482 | **0.280** |
| **LC-PKZBPBE** | 0.159 | 0.151 | 0.561 | 0.562 | 0.305 | 0.492 | 0.464 | 0.388 | 0.488 | 0.464 | 0.389 | 0.498 | 0.234 | 0.452 | 0.272 | 0.491 | 0.348 | 0.566 | 0.580 | 0.601 | **0.423** |
| **LC-PKZBPKZB** | 0.170 | 0.166 | 0.562 | 0.564 | 0.304 | 0.496 | 0.465 | 0.395 | 0.492 | 0.464 | 0.395 | 0.498 | 0.222 | 0.481 | 0.294 | 0.494 | 0.352 | 0.568 | 0.582 | 0.599 | **0.428** |
| **LC-PKZBPL** | 0.214 | 0.215 | 0.510 | 0.510 | 0.301 | 0.475 | 0.422 | 0.377 | 0.474 | 0.426 | 0.377 | 0.471 | 0.270 | 0.469 | 0.270 | 0.497 | 0.339 | 0.538 | 0.529 | 0.567 | **0.413** |
| **LC-PKZBPW91** | 0.138 | 0.131 | 0.556 | 0.557 | 0.278 | 0.492 | 0.451 | 0.371 | 0.478 | 0.443 | 0.368 | 0.490 | 0.233 | 0.425 | 0.252 | 0.479 | 0.336 | 0.561 | 0.569 | 0.592 | **0.410** |
| **LC-PKZBRevTPS** | 0.110 | 0.107 | 0.519 | 0.518 | 0.233 | 0.452 | 0.388 | 0.281 | 0.450 | 0.359 | 0.277 | 0.449 | 0.231 | 0.413 | 0.229 | 0.390 | 0.312 | 0.495 | 0.531 | 0.549 | **0.365** |
| **LC-PKZBTPSS** | 0.173 | 0.170 | 0.580 | 0.566 | 0.303 | 0.501 | 0.466 | 0.394 | 0.498 | 0.465 | 0.394 | 0.532 | 0.222 | 0.483 | 0.290 | 0.494 | 0.350 | 0.566 | 0.574 | 0.630 | **0.433** |
| **LC-PKZBVP86** | 0.155 | 0.156 | 0.465 | 0.464 | 0.222 | 0.215 | 0.159 | 0.150 | 0.218 | 0.159 | 0.177 | 0.215 | 0.354 | 0.361 | 0.281 | 0.189 | 0.373 | 0.390 | 0.455 | 0.484 | **0.282** |
| **LC-PKZBVWN** | 0.176 | 0.184 | 0.421 | 0.421 | 0.265 | 0.376 | 0.311 | 0.278 | 0.374 | 0.309 | 0.278 | 0.374 | 0.272 | 0.371 | 0.243 | 0.361 | 0.270 | 0.408 | 0.409 | 0.421 | **0.326** |
| **LC-PKZBVWN5** | 0.218 | 0.218 | 0.529 | 0.530 | 0.302 | 0.484 | 0.430 | 0.379 | 0.478 | 0.430 | 0.379 | 0.482 | 0.270 | 0.474 | 0.272 | 0.504 | 0.346 | 0.556 | 0.534 | 0.566 | **0.419** |
| **LC-PW91B95** | **0.060** | **0.060** | **0.099** | **0.099** | 0.158 | **0.086** | **0.086** | **0.085** | **0.086** | **0.086** | **0.085** | **0.085** | 0.230 | 0.107 | 0.111 | **0.086** | **0.096** | **0.097** | **0.099** | 0.101 | **0.100** |
| **LC-PW91BRC** | 0.119 | 0.123 | 0.359 | 0.359 | 0.265 | 0.317 | 0.246 | 0.221 | 0.316 | 0.247 | 0.219 | 0.319 | 0.305 | 0.308 | 0.233 | 0.269 | 0.240 | 0.375 | 0.357 | 0.434 | **0.282** |
| **LC-PW91KCIS** | **0.077** | **0.083** | 0.183 | 0.183 | 0.224 | 0.195 | 0.131 | 0.112 | 0.194 | 0.131 | 0.110 | 0.187 | 0.282 | 0.154 | 0.177 | 0.124 | 0.136 | 0.181 | 0.179 | 0.202 | **0.162** |
| **LC-PW91LYP** | 0.349 | 0.349 | 0.304 | 0.304 | 0.407 | 0.311 | 0.322 | 0.320 | 0.312 | 0.324 | 0.322 | 0.305 | 0.500 | 0.321 | 0.363 | 0.309 | 0.319 | 0.302 | 0.304 | 0.303 | **0.333** |
| **LC-PW91P86** | 0.174 | 0.177 | 0.463 | 0.464 | 0.239 | 0.214 | 0.160 | 0.176 | 0.208 | 0.162 | 0.176 | 0.206 | 0.366 | 0.328 | 0.298 | 0.213 | 0.392 | 0.421 | 0.467 | 0.492 | **0.290** |
| **LC-PW91PBE** | 0.154 | 0.141 | 0.569 | 0.569 | 0.284 | 0.494 | 0.456 | 0.374 | 0.482 | 0.456 | 0.372 | 0.496 | 0.238 | 0.459 | 0.250 | 0.451 | 0.344 | 0.563 | 0.570 | 0.588 | **0.416** |
| **LC-PW91PKZB** | 0.151 | 0.146 | 0.570 | 0.570 | 0.290 | 0.488 | 0.457 | 0.379 | 0.481 | 0.457 | 0.375 | 0.483 | 0.241 | 0.445 | 0.272 | 0.474 | 0.347 | 0.565 | 0.571 | 0.607 | **0.419** |
| **LC-PW91PL** | 0.207 | 0.208 | 0.494 | 0.494 | 0.292 | 0.455 | 0.387 | 0.352 | 0.455 | 0.387 | 0.349 | 0.454 | 0.274 | 0.456 | 0.268 | 0.478 | 0.328 | 0.513 | 0.505 | 0.556 | **0.396** |
| **LC-PW91PW91** | 0.126 | 0.121 | 0.544 | 0.543 | 0.257 | 0.483 | 0.390 | 0.338 | 0.469 | 0.359 | 0.311 | 0.485 | 0.238 | 0.419 | 0.256 | 0.400 | 0.330 | 0.536 | 0.557 | 0.580 | **0.387** |
| **LC-PW91RevTPS** | 0.101 | **0.098** | 0.517 | 0.510 | 0.230 | 0.434 | 0.302 | 0.239 | 0.466 | 0.307 | 0.237 | 0.434 | 0.237 | 0.394 | 0.225 | 0.349 | 0.307 | 0.464 | 0.511 | 0.542 | **0.345** |
| **LC-PW91TPSS** | 0.146 | 0.145 | 0.570 | 0.571 | 0.284 | 0.490 | 0.459 | 0.379 | 0.479 | 0.457 | 0.379 | 0.489 | 0.241 | 0.440 | 0.262 | 0.489 | 0.344 | 0.563 | 0.572 | 0.606 | **0.418** |
| **LC-PW91VP86** | 0.173 | 0.174 | 0.467 | 0.467 | 0.237 | 0.200 | 0.161 | 0.170 | 0.203 | 0.163 | 0.173 | 0.200 | 0.365 | 0.334 | 0.295 | 0.207 | 0.388 | 0.419 | 0.466 | 0.491 | **0.288** |
| **LC-PW91VWN** | 0.168 | 0.176 | 0.382 | 0.382 | 0.262 | 0.352 | 0.293 | 0.266 | 0.351 | 0.292 | 0.266 | 0.353 | 0.276 | 0.355 | 0.237 | 0.334 | 0.267 | 0.393 | 0.380 | 0.411 | **0.310** |
| **LC-PW91VWN5** | 0.210 | 0.210 | 0.507 | 0.507 | 0.296 | 0.469 | 0.400 | 0.358 | 0.467 | 0.399 | 0.358 | 0.463 | 0.274 | 0.459 | 0.270 | 0.496 | 0.330 | 0.549 | 0.525 | 0.576 | **0.406** |
| **LC-RevTPSSB95** | **0.081** | **0.081** | **0.099** | **0.099** | 0.156 | **0.086** | **0.086** | **0.085** | **0.086** | **0.086** | **0.085** | **0.085** | 0.225 | 0.107 | 0.110 | **0.086** | **0.082** | **0.097** | 0.100 | 0.101 | **0.101** |
| **LC-RevTPSSBRC** | 0.124 | 0.129 | 0.390 | 0.390 | 0.269 | 0.343 | 0.252 | 0.226 | 0.342 | 0.251 | 0.225 | 0.344 | 0.299 | 0.306 | 0.237 | 0.282 | 0.249 | 0.384 | 0.387 | 0.434 | **0.293** |
| **LC-RevTPSSKCI** | **0.080** | **0.087** | 0.193 | 0.193 | 0.222 | 0.204 | 0.140 | 0.120 | 0.202 | 0.139 | 0.119 | 0.192 | 0.273 | 0.198 | 0.179 | 0.152 | 0.152 | 0.193 | 0.191 | 0.232 | **0.173** |
| **LC-RevTPSSLYP** | 0.341 | 0.342 | 0.300 | 0.300 | 0.401 | 0.308 | 0.318 | 0.317 | 0.310 | 0.320 | 0.318 | 0.303 | 0.492 | 0.320 | 0.358 | 0.304 | 0.314 | 0.298 | 0.300 | 0.298 | **0.328** |
| **LC-RevTPSSP86** | 0.159 | 0.157 | 0.463 | 0.461 | 0.222 | 0.204 | 0.151 | 0.158 | 0.206 | 0.158 | 0.160 | 0.212 | 0.355 | 0.342 | 0.282 | 0.225 | 0.374 | 0.358 | 0.463 | 0.496 | **0.280** |
| **LC-RevTPSSPBE** | 0.169 | 0.159 | 0.544 | 0.544 | 0.305 | 0.531 | 0.459 | 0.380 | 0.512 | 0.457 | 0.374 | 0.514 | 0.219 | 0.427 | 0.273 | 0.480 | 0.377 | 0.566 | 0.576 | 0.572 | **0.422** |
| **LC-RevTPSSPKZ** | 0.188 | 0.185 | 0.547 | 0.546 | 0.304 | 0.518 | 0.462 | 0.384 | 0.517 | 0.461 | 0.380 | 0.540 | 0.220 | 0.439 | 0.276 | 0.498 | 0.378 | 0.565 | 0.578 | 0.573 | **0.428** |
| **LC-RevTPSSPL** | 0.218 | 0.219 | 0.499 | 0.499 | 0.303 | 0.459 | 0.397 | 0.358 | 0.458 | 0.394 | 0.358 | 0.461 | 0.265 | 0.465 | 0.267 | 0.490 | 0.345 | 0.510 | 0.520 | 0.546 | **0.402** |
| **LC-RevTPSSPW9** | 0.147 | 0.147 | 0.564 | 0.563 | 0.284 | 0.476 | 0.393 | 0.342 | 0.476 | 0.385 | 0.320 | 0.476 | 0.230 | 0.425 | 0.255 | 0.430 | 0.342 | 0.542 | 0.570 | 0.587 | **0.398** |
| **LC-RevTPSSRev** | 0.115 | 0.112 | 0.492 | 0.493 | 0.248 | 0.444 | 0.317 | 0.255 | 0.417 | 0.317 | 0.254 | 0.446 | 0.229 | 0.391 | 0.231 | 0.391 | 0.315 | 0.494 | 0.521 | 0.549 | **0.352** |
| **LC-RevTPSSTPS** | 0.176 | 0.178 | 0.545 | 0.544 | 0.304 | 0.515 | 0.460 | 0.383 | 0.515 | 0.460 | 0.382 | 0.544 | 0.220 | 0.441 | 0.272 | 0.481 | 0.376 | 0.568 | 0.579 | 0.570 | **0.426** |
| **LC-RevTPSSVP8** | 0.155 | 0.154 | 0.415 | 0.456 | 0.220 | 0.230 | 0.145 | 0.157 | 0.225 | 0.147 | 0.158 | 0.219 | 0.354 | 0.421 | 0.281 | 0.222 | 0.372 | 0.357 | 0.460 | 0.492 | **0.282** |
| **LC-RevTPSSVW** | 0.179 | 0.187 | 0.394 | 0.394 | 0.266 | 0.363 | 0.295 | 0.268 | 0.362 | 0.294 | 0.267 | 0.365 | 0.269 | 0.375 | 0.239 | 0.356 | 0.270 | 0.410 | 0.396 | 0.420 | **0.318** |
| **LC-RevTPSSVW** | 0.221 | 0.222 | 0.503 | 0.503 | 0.304 | 0.463 | 0.408 | 0.368 | 0.462 | 0.408 | 0.368 | 0.464 | 0.263 | 0.473 | 0.270 | 0.495 | 0.349 | 0.526 | 0.525 | 0.538 | **0.407** |
| **LC-SB95** | **0.070** | **0.060** | **0.099** | **0.099** | 0.182 | **0.085** | **0.085** | **0.084** | **0.085** | **0.085** | **0.084** | **0.085** | 0.233 | 0.107 | 0.111 | **0.085** | **0.096** | **0.096** | **0.099** | 0.100 | **0.101** |
| **LC-SBRC** | 0.117 | 0.121 | 0.379 | 0.379 | 0.262 | 0.343 | 0.252 | 0.227 | 0.338 | 0.248 | 0.226 | 0.363 | 0.304 | 0.314 | 0.232 | 0.261 | 0.241 | 0.381 | 0.372 | 0.439 | **0.290** |
| **LC-SKCIS** | **0.073** | **0.083** | 0.189 | 0.189 | 0.222 | 0.200 | 0.135 | 0.114 | 0.199 | 0.134 | 0.113 | 0.191 | 0.280 | 0.156 | 0.175 | 0.134 | 0.136 | 0.187 | 0.184 | 0.224 | **0.166** |
| **LC-SLYP** | 0.346 | 0.346 | 0.297 | 0.297 | 0.404 | 0.305 | 0.317 | 0.315 | 0.306 | 0.319 | 0.317 | 0.299 | 0.499 | 0.317 | 0.361 | 0.305 | 0.314 | 0.297 | 0.297 | 0.297 | **0.328** |
| **LC-SP86** | 0.179 | 0.180 | 0.476 | 0.476 | 0.244 | 0.204 | 0.162 | 0.213 | 0.205 | 0.170 | 0.215 | 0.200 | 0.386 | 0.349 | 0.312 | 0.220 | 0.314 | 0.398 | 0.472 | 0.513 | **0.294** |
| **LC-SPBE** | 0.141 | 0.136 | 0.576 | 0.576 | 0.283 | 0.485 | 0.457 | 0.357 | 0.484 | 0.458 | 0.355 | 0.485 | 0.236 | 0.422 | 0.257 | 0.463 | 0.343 | 0.559 | 0.564 | 0.590 | **0.411** |
| **LC-SPKZB** | 0.145 | 0.141 | 0.603 | 0.603 | 0.301 | 0.488 | 0.460 | 0.374 | 0.488 | 0.461 | 0.371 | 0.484 | 0.240 | 0.429 | 0.280 | 0.481 | 0.346 | 0.561 | 0.562 | 0.593 | **0.421** |

| | | | | | | | | | | | | | | | | | | | | | |
|---|---|---|---|---|---|---|---|---|---|---|---|---|---|---|---|---|---|---|---|---|---|
| **LC-SPL** | 0.206 | 0.208 | 0.507 | 0.507 | 0.292 | 0.482 | 0.406 | 0.354 | 0.477 | 0.404 | 0.350 | 0.488 | 0.273 | 0.452 | 0.264 | 0.446 | 0.331 | 0.526 | 0.522 | 0.543 | **0.402** |
| **LC-SPW91** | 0.123 | 0.117 | 0.571 | 0.571 | 0.244 | 0.475 | 0.408 | 0.311 | 0.472 | 0.389 | 0.310 | 0.474 | 0.234 | 0.415 | 0.227 | 0.412 | 0.331 | 0.571 | 0.550 | 0.580 | **0.389** |
| **LC-SRevTPSS** | **0.095** | **0.092** | 0.483 | 0.483 | 0.226 | 0.474 | 0.307 | 0.236 | 0.471 | 0.300 | 0.278 | 0.456 | 0.234 | 0.359 | 0.216 | 0.313 | 0.305 | 0.467 | 0.475 | 0.552 | **0.341** |
| **LC-STPSS** | 0.144 | 0.140 | 0.581 | 0.581 | 0.283 | 0.480 | 0.458 | 0.374 | 0.479 | 0.459 | 0.371 | 0.487 | 0.240 | 0.421 | 0.268 | 0.463 | 0.344 | 0.556 | 0.562 | 0.582 | **0.414** |
| **LC-SVP86** | 0.176 | 0.177 | 0.475 | 0.475 | 0.240 | 0.205 | 0.163 | 0.205 | 0.210 | 0.164 | 0.213 | 0.206 | 0.332 | 0.328 | 0.307 | 0.218 | 0.270 | 0.397 | 0.467 | 0.512 | **0.287** |
| **LC-SVWN5** | 0.208 | 0.210 | 0.524 | 0.524 | 0.295 | 0.484 | 0.412 | 0.357 | 0.482 | 0.409 | 0.356 | 0.491 | 0.272 | 0.455 | 0.265 | 0.451 | 0.345 | 0.546 | 0.526 | 0.586 | **0.410** |
| **LC-TPSSB95** | **0.081** | **0.081** | **0.099** | **0.099** | 0.157 | **0.086** | **0.086** | **0.085** | **0.086** | **0.086** | **0.085** | **0.086** | 0.209 | 0.107 | 0.111 | **0.086** | **0.097** | **0.097** | 0.100 | 0.101 | **0.101** |
| **LC-TPSSBRC** | 0.123 | 0.128 | 0.390 | 0.390 | 0.267 | 0.318 | 0.246 | 0.222 | 0.327 | 0.245 | 0.221 | 0.329 | 0.301 | 0.305 | 0.236 | 0.275 | 0.246 | 0.383 | 0.372 | 0.433 | **0.288** |
| **LC-TPSSKCIS** | **0.080** | **0.086** | 0.190 | 0.190 | 0.222 | 0.198 | 0.135 | 0.114 | 0.196 | 0.134 | 0.113 | 0.187 | 0.275 | 0.195 | 0.178 | 0.146 | 0.150 | 0.186 | 0.187 | 0.213 | **0.169** |
| **LC-TPSSLYP** | 0.346 | 0.346 | 0.304 | 0.304 | 0.405 | 0.312 | 0.323 | 0.320 | 0.313 | 0.324 | 0.322 | 0.307 | 0.496 | 0.323 | 0.361 | 0.308 | 0.318 | 0.302 | 0.305 | 0.303 | **0.332** |
| **LC-TPSSP86** | 0.164 | 0.163 | 0.466 | 0.463 | 0.227 | 0.202 | 0.158 | 0.165 | 0.201 | 0.174 | 0.167 | 0.192 | 0.358 | 0.334 | 0.286 | 0.207 | 0.378 | 0.399 | 0.469 | 0.492 | **0.283** |
| **LC-TPSSPBE** | 0.165 | 0.158 | 0.542 | 0.541 | 0.291 | 0.484 | 0.454 | 0.373 | 0.484 | 0.452 | 0.370 | 0.510 | 0.233 | 0.421 | 0.268 | 0.480 | 0.352 | 0.532 | 0.575 | 0.597 | **0.414** |
| **LC-TPSSPKZB** | 0.170 | 0.167 | 0.544 | 0.544 | 0.303 | 0.486 | 0.458 | 0.378 | 0.508 | 0.457 | 0.375 | 0.481 | 0.222 | 0.427 | 0.272 | 0.485 | 0.376 | 0.532 | 0.577 | 0.595 | **0.418** |
| **LC-TPSSPL** | 0.215 | 0.217 | 0.496 | 0.496 | 0.301 | 0.457 | 0.392 | 0.353 | 0.457 | 0.389 | 0.350 | 0.459 | 0.269 | 0.464 | 0.267 | 0.485 | 0.331 | 0.507 | 0.498 | 0.532 | **0.397** |
| **LC-TPSSPW91** | 0.148 | 0.131 | 0.564 | 0.563 | 0.281 | 0.474 | 0.374 | 0.329 | 0.481 | 0.379 | 0.327 | 0.475 | 0.232 | 0.404 | 0.255 | 0.421 | 0.339 | 0.523 | 0.568 | 0.585 | **0.393** |
| **LC-TPSSRevTPS** | 0.112 | 0.109 | 0.488 | 0.486 | 0.235 | 0.448 | 0.304 | 0.242 | 0.474 | 0.304 | 0.218 | 0.444 | 0.231 | 0.382 | 0.229 | 0.391 | 0.312 | 0.498 | 0.516 | 0.549 | **0.349** |
| **LC-TPSSTPSS** | 0.163 | 0.159 | 0.542 | 0.542 | 0.292 | 0.487 | 0.459 | 0.378 | 0.505 | 0.458 | 0.375 | 0.506 | 0.221 | 0.429 | 0.272 | 0.475 | 0.376 | 0.531 | 0.576 | 0.570 | **0.416** |
| **LC-TPSSVP86** | 0.163 | 0.161 | 0.457 | 0.457 | 0.225 | 0.207 | 0.149 | 0.160 | 0.206 | 0.150 | 0.163 | 0.211 | 0.357 | 0.335 | 0.286 | 0.205 | 0.376 | 0.397 | 0.462 | 0.498 | **0.281** |
| **LC-TPSSVWN** | 0.176 | 0.185 | 0.385 | 0.385 | 0.264 | 0.351 | 0.292 | 0.263 | 0.350 | 0.291 | 0.262 | 0.357 | 0.271 | 0.369 | 0.236 | 0.344 | 0.268 | 0.399 | 0.386 | 0.415 | **0.312** |
| **LC-TPSSVWN5** | 0.218 | 0.220 | 0.499 | 0.499 | 0.301 | 0.460 | 0.401 | 0.356 | 0.458 | 0.400 | 0.356 | 0.463 | 0.265 | 0.469 | 0.269 | 0.489 | 0.342 | 0.523 | 0.503 | 0.575 | **0.403** |
| **LC-XaB95** | **0.071** | **0.071** | 0.100 | 0.100 | 0.174 | **0.086** | **0.086** | 0.104 | **0.086** | **0.086** | **0.085** | **0.086** | 0.234 | 0.108 | 0.111 | **0.086** | **0.098** | **0.098** | 0.101 | 0.102 | **0.104** |
| **LC-XaBRC** | 0.116 | 0.120 | 0.368 | 0.368 | 0.260 | 0.310 | 0.235 | 0.216 | 0.307 | 0.233 | 0.215 | 0.313 | 0.305 | 0.294 | 0.229 | 0.256 | 0.238 | 0.382 | 0.368 | 0.432 | **0.278** |
| **LC-XaKCIS** | **0.075** | **0.083** | 0.181 | 0.181 | 0.220 | 0.171 | 0.125 | 0.104 | 0.170 | 0.125 | 0.103 | 0.177 | 0.282 | 0.143 | 0.173 | 0.114 | 0.130 | 0.175 | 0.177 | 0.199 | **0.155** |
| **LC-XaLYP** | 0.361 | 0.361 | 0.318 | 0.318 | 0.417 | 0.322 | 0.337 | 0.335 | 0.324 | 0.339 | 0.337 | 0.316 | 0.508 | 0.336 | 0.374 | 0.324 | 0.333 | 0.318 | 0.319 | 0.319 | **0.346** |
| **LC-XaP86** | 0.182 | 0.184 | 0.479 | 0.479 | 0.248 | 0.208 | 0.167 | 0.214 | 0.208 | 0.186 | 0.223 | 0.199 | 0.373 | 0.358 | 0.304 | 0.225 | 0.390 | 0.410 | 0.450 | 0.494 | **0.299** |
| **LC-XaPBE** | 0.141 | 0.137 | 0.567 | 0.568 | 0.281 | 0.480 | 0.404 | 0.339 | 0.500 | 0.401 | 0.340 | 0.478 | 0.237 | 0.415 | 0.248 | 0.455 | 0.344 | 0.560 | 0.553 | 0.585 | **0.402** |
| **LC-XaPKZB** | 0.146 | 0.142 | 0.569 | 0.569 | 0.282 | 0.478 | 0.455 | 0.345 | 0.478 | 0.440 | 0.345 | 0.478 | 0.240 | 0.422 | 0.267 | 0.460 | 0.346 | 0.563 | 0.554 | 0.587 | **0.408** |
| **LC-XaPL** | 0.204 | 0.206 | 0.489 | 0.489 | 0.281 | 0.464 | 0.381 | 0.335 | 0.463 | 0.380 | 0.334 | 0.461 | 0.273 | 0.445 | 0.264 | 0.439 | 0.328 | 0.501 | 0.497 | 0.518 | **0.387** |
| **LC-XaPW91** | 0.124 | 0.120 | 0.546 | 0.543 | 0.241 | 0.471 | 0.342 | 0.319 | 0.471 | 0.354 | 0.289 | 0.471 | 0.236 | 0.430 | 0.221 | 0.371 | 0.336 | 0.533 | 0.526 | 0.579 | **0.376** |
| **LC-XaRevTPSS** | **0.097** | **0.094** | 0.473 | 0.472 | 0.225 | 0.439 | 0.349 | 0.285 | 0.459 | 0.314 | 0.229 | 0.445 | 0.234 | 0.393 | 0.215 | 0.309 | 0.308 | 0.470 | 0.467 | 0.549 | **0.341** |
| **LC-XaTPSS** | 0.145 | 0.141 | 0.571 | 0.571 | 0.283 | 0.481 | 0.455 | 0.353 | 0.479 | 0.456 | 0.354 | 0.481 | 0.241 | 0.426 | 0.274 | 0.460 | 0.345 | 0.562 | 0.549 | 0.587 | **0.411** |
| **BB1K** | 0.176 | 0.176 | 0.179 | 0.179 | 0.149 | 0.237 | 0.235 | 0.234 | 0.317 | 0.235 | 0.234 | 0.237 | 0.149 | 0.436 | 0.175 | 0.239 | 0.179 | 0.180 | 0.180 | 0.180 | **0.215** |
| **mPW1K** | 0.388 | 0.379 | 0.521 | 0.521 | 0.339 | 0.494 | 0.441 | 0.386 | 0.493 | 0.440 | 0.385 | 0.490 | 0.274 | 0.418 | 0.332 | 0.533 | 0.450 | 0.549 | 0.532 | 0.540 | **0.445** |
| **mPW1B95** | 0.108 | 0.108 | 0.128 | 0.128 | 0.163 | 0.131 | 0.115 | 0.114 | 0.131 | 0.115 | 0.114 | 0.131 | 0.149 | 0.136 | 0.129 | 0.118 | 0.126 | 0.145 | 0.128 | 0.129 | **0.127** |
| **mPWB1K** | 0.113 | 0.113 | 0.108 | 0.108 | 0.143 | **0.094** | **0.097** | **0.095** | **0.094** | **0.097** | **0.095** | 0.118 | 0.133 | **0.094** | 0.110 | 0.105 | 0.106 | 0.108 | 0.108 | 0.109 | **0.107** |
| **mPW1KCIS** | 0.257 | 0.260 | 0.401 | 0.401 | 0.293 | 0.373 | 0.347 | 0.326 | 0.373 | 0.346 | 0.326 | 0.377 | 0.257 | 0.360 | 0.235 | 0.412 | 0.301 | 0.424 | 0.398 | 0.419 | **0.344** |
| **mPWKCIS1K** | 0.244 | 0.246 | 0.367 | 0.367 | 0.262 | 0.320 | 0.282 | 0.261 | 0.318 | 0.281 | 0.260 | 0.324 | 0.232 | 0.313 | 0.226 | 0.357 | 0.293 | 0.386 | 0.367 | 0.374 | **0.304** |
| **TPSS1KCIS** | 0.177 | 0.180 | 0.211 | 0.211 | 0.253 | 0.207 | 0.193 | 0.178 | 0.207 | 0.193 | 0.177 | 0.213 | 0.267 | 0.209 | 0.187 | 0.230 | 0.193 | 0.226 | 0.211 | 0.210 | **0.207** |
| **PBE1KCIS** | 0.151 | 0.154 | 0.117 | 0.117 | 0.250 | 0.119 | 0.116 | 0.122 | 0.118 | 0.116 | 0.122 | 0.127 | 0.296 | 0.108 | 0.199 | 0.116 | 0.133 | 0.117 | 0.119 | 0.117 | **0.142** |
| **mPWLYP1M** | 0.249 | 0.251 | 0.212 | 0.212 | 0.349 | 0.225 | 0.228 | 0.230 | 0.226 | 0.228 | 0.230 | 0.230 | 0.379 | 0.218 | 0.297 | 0.214 | 0.246 | 0.207 | 0.215 | 0.210 | **0.243** |
| **PBE1W** | 0.156 | 0.160 | 0.156 | 0.155 | 0.266 | 0.153 | 0.142 | 0.135 | 0.152 | 0.141 | 0.133 | 0.162 | 0.306 | 0.148 | 0.212 | 0.161 | 0.156 | 0.161 | 0.155 | 0.155 | **0.168** |
| **mPWLYP1W** | 0.230 | 0.232 | 0.201 | 0.201 | 0.346 | 0.197 | 0.203 | 0.210 | 0.197 | 0.204 | 0.210 | 0.213 | 0.370 | 0.188 | 0.284 | 0.210 | 0.230 | 0.209 | 0.203 | 0.204 | **0.227** |
| **PBELYP1W** | 0.247 | 0.249 | 0.217 | 0.217 | 0.325 | 0.217 | 0.221 | 0.224 | 0.217 | 0.221 | 0.224 | 0.222 | 0.368 | 0.222 | 0.284 | 0.212 | 0.243 | 0.216 | 0.220 | 0.218 | **0.239** |
| **TPSSLYP1W** | 0.240 | 0.241 | 0.210 | 0.210 | 0.330 | 0.219 | 0.223 | 0.225 | 0.219 | 0.222 | 0.225 | 0.225 | 0.371 | 0.220 | 0.289 | 0.211 | 0.239 | 0.206 | 0.215 | 0.211 | **0.238** |
| **mPW3LYP** | 0.235 | 0.236 | 0.205 | 0.205 | 0.326 | 0.220 | 0.223 | 0.226 | 0.220 | 0.224 | 0.226 | 0.224 | 0.363 | 0.213 | 0.279 | 0.206 | 0.232 | 0.204 | 0.208 | 0.204 | **0.234** |
| **PBEPBE-D2** | 0.208 | 0.211 | 0.158 | 0.158 | 0.261 | 0.147 | 0.167 | 0.169 | 0.148 | 0.169 | 0.171 | 0.147 | 0.342 | 0.150 | 0.229 | 0.157 | 0.182 | 0.161 | 0.160 | 0.158 | **0.183** |
| **BLYP-D2** | 0.211 | 0.102 | 0.317 | 0.317 | 0.186 | 0.405 | 0.392 | 0.396 | 0.406 | 0.429 | 0.396 | 0.413 | 0.268 | 0.321 | 0.297 | 0.405 | 0.299 | 0.402 | 0.319 | 0.402 | **0.334** |
| **B3LYP-D2** | 0.170 | 0.175 | **0.097** | **0.097** | 0.226 | **0.093** | 0.112 | 0.110 | **0.094** | 0.114 | 0.111 | **0.097** | 0.323 | **0.097** | 0.171 | **0.091** | 0.117 | 0.100 | 0.100 | **0.097** | **0.130** |
| **BP86-D2** | 0.988 | 0.990 | 1.385 | 1.414 | 0.646 | 1.149 | 1.105 | 1.033 | 1.145 | 1.096 | 1.026 | 1.283 | 0.482 | 1.201 | 0.691 | 1.295 | 1.036 | 1.316 | 1.288 | 1.403 | **1.099** |
| **TPSSTPSS-D2** | 0.142 | 0.144 | 0.101 | 0.102 | 0.216 | 0.111 | 0.118 | 0.122 | 0.111 | 0.118 | 0.122 | 0.118 | 0.259 | 0.105 | 0.173 | **0.092** | 0.144 | **0.098** | 0.117 | 0.104 | **0.131** |
| **PBEPBE-D3** | 0.166 | 0.169 | 0.123 | 0.123 | 0.254 | 0.124 | 0.132 | 0.140 | 0.124 | 0.132 | 0.140 | 0.130 | 0.297 | 0.113 | 0.207 | 0.118 | 0.160 | 0.122 | 0.126 | 0.123 | **0.151** |
| **BLYP-D3** | 0.102 | 0.125 | 0.193 | 0.191 | 0.214 | 0.383 | 0.228 | 0.260 | 0.383 | 0.297 | 0.268 | 0.392 | 0.294 | 0.221 | 0.143 | 0.260 | 0.114 | 0.211 | 0.197 | 0.220 | **0.235** |
| **B3LYP-D3** | 0.106 | 0.111 | **0.066** | **0.064** | 0.209 | **0.068** | **0.080** | **0.087** | **0.069** | **0.080** | **0.087** | **0.076** | 0.300 | **0.068** | 0.154 | **0.043** | 0.107 | **0.065** | **0.071** | **0.066** | **0.099** |
| **BP86-D3** | 0.841 | 0.840 | 1.182 | 1.182 | 0.468 | 0.964 | 0.943 | 0.872 | 0.968 | 0.943 | 0.872 | 0.979 | 0.339 | 1.134 | 0.586 | 1.094 | 0.838 | 1.193 | 1.126 | 1.245 | **0.931** |
| **TPSSTPSS-D3** | 0.148 | 0.151 | 0.138 | 0.138 | 0.228 | 0.130 | 0.125 | 0.124 | 0.130 | 0.125 | 0.124 | 0.134 | 0.265 | 0.126 | 0.178 | 0.140 | 0.142 | 0.143 | 0.135 | 0.137 | **0.148** |
| **PBE1PBE-D3** | 0.121 | 0.124 | **0.089** | **0.089** | 0.214 | **0.096** | **0.098** | 0.111 | **0.097** | **0.098** | 0.112 | 0.104 | 0.272 | **0.067** | 0.166 | **0.084** | 0.115 | **0.090** | **0.092** | **0.088** | **0.116** |
| **BPBE-D3** | 0.626 | 0.636 | 1.076 | 1.073 | 0.402 | 0.876 | 0.831 | 0.743 | 0.859 | 0.814 | 0.718 | 0.875 | 0.351 | 0.883 | 0.518 | 1.054 | 0.736 | 1.171 | 0.957 | 1.173 | **0.819** |

| | | | | | | | | | | | | | | | | | | | | | |
|---|---|---|---|---|---|---|---|---|---|---|---|---|---|---|---|---|---|---|---|---|---|
| **B3PW91-D3** | 0.480 | 0.479 | 0.688 | 0.662 | 0.319 | 0.615 | 0.535 | 0.478 | 0.613 | 0.535 | 0.477 | 0.592 | 0.246 | 0.562 | 0.383 | 0.731 | 0.514 | 0.718 | 0.653 | 0.759 | **0.552** |
| **BMK-D3** | 0.873 | 0.854 | 1.063 | 1.063 | 0.604 | 0.975 | 0.857 | 0.849 | 0.832 | 0.855 | 0.869 | 0.832 | 0.450 | 0.958 | 0.740 | 0.966 | 0.921 | 0.988 | 1.019 | 1.045 | **0.881** |
| **CAM-B3LYP-D3** | 0.259 | 0.260 | 0.193 | 0.193 | 0.320 | 0.203 | 0.222 | 0.220 | 0.205 | 0.224 | 0.222 | 0.191 | 0.418 | 0.210 | 0.275 | 0.200 | 0.219 | 0.196 | 0.196 | 0.193 | **0.231** |
| **LC-wPBE-D3** | **0.049** | **0.052** | 0.157 | 0.157 | 0.178 | 0.162 | 0.129 | 0.115 | 0.161 | 0.128 | 0.112 | 0.167 | 0.242 | 0.134 | 0.132 | 0.140 | **0.093** | 0.173 | 0.155 | 0.166 | **0.140** |
| **M05-D3** | 0.235 | 0.235 | 0.216 | 0.216 | 0.318 | 0.233 | 0.235 | 0.231 | 0.234 | 0.237 | 0.232 | 0.231 | 0.404 | 0.280 | 0.273 | 0.207 | 0.221 | 0.195 | 0.205 | 0.199 | **0.242** |
| **M052X-D3** | 0.155 | 0.155 | 0.148 | 0.148 | 0.245 | 0.172 | 0.177 | 0.178 | 0.172 | 0.177 | 0.178 | 0.167 | 0.284 | 0.140 | 0.202 | 0.152 | 0.149 | 0.145 | 0.147 | 0.144 | **0.172** |
| **M06-D3** | **0.068** | **0.067** | 0.172 | 0.172 | **0.069** | 0.136 | **0.076** | **0.073** | 0.136 | **0.076** | **0.073** | 0.137 | **0.077** | **0.057** | **0.053** | 0.123 | 0.167 | 0.172 | 0.171 | 0.118 | **0.110** |
| **M06L-D3** | 0.122 | 0.122 | 0.141 | 0.141 | 0.145 | 0.128 | 0.101 | **0.098** | 0.128 | 0.101 | **0.074** | 0.128 | 0.113 | **0.083** | 0.112 | **0.067** | 0.136 | 0.101 | 0.111 | 0.128 | **0.114** |
| **M06HF-D3** | 0.140 | 0.140 | 0.146 | 0.146 | 0.168 | 0.148 | 0.148 | 0.151 | 0.148 | 0.148 | 0.151 | 0.148 | **0.056** | 0.116 | 0.148 | 0.147 | 0.146 | 0.211 | 0.145 | 0.139 | **0.145** |
| **M062X-D3** | 0.126 | 0.127 | 0.149 | 0.149 | 0.238 | 0.145 | 0.135 | 0.149 | 0.144 | 0.144 | 0.149 | 0.147 | 0.254 | **0.041** | 0.168 | 0.119 | 0.155 | 0.157 | 0.148 | 0.155 | **0.150** |
| **PBEPBE-D3BJ** | 0.150 | 0.153 | **0.097** | **0.097** | 0.237 | **0.098** | 0.110 | 0.118 | **0.098** | 0.111 | 0.119 | 0.104 | 0.292 | **0.091** | 0.188 | **0.090** | 0.140 | **0.095** | 0.104 | **0.097** | **0.129** |
| **BLYP-D3BJ** | 0.141 | 0.115 | 0.403 | 0.404 | 0.284 | 0.430 | 0.336 | 0.313 | 0.464 | 0.338 | 0.312 | 0.478 | 0.288 | 0.358 | 0.292 | 0.528 | 0.219 | 0.561 | 0.393 | 0.540 | **0.360** |
| **B3LYP-D3BJ** | **0.052** | **0.062** | **0.082** | **0.083** | 0.170 | 0.165 | 0.151 | 0.150 | 0.165 | 0.166 | 0.154 | 0.172 | 0.256 | 0.131 | 0.110 | 0.151 | **0.079** | **0.077** | **0.073** | 0.164 | **0.131** |
| **BP86-D3BJ** | 0.893 | 0.893 | 1.153 | 1.152 | 0.588 | 0.982 | 0.944 | 0.902 | 0.958 | 0.936 | 0.898 | 1.002 | 0.536 | 1.003 | 0.788 | 1.103 | 0.933 | 1.130 | 1.163 | 1.207 | **0.958** |
| **TPSSTPSS-D3BJ** | 0.121 | 0.124 | 0.132 | 0.132 | 0.200 | 0.113 | 0.108 | **0.095** | 0.112 | 0.108 | **0.099** | 0.118 | 0.230 | 0.119 | 0.148 | 0.135 | 0.115 | 0.137 | 0.130 | 0.131 | **0.130** |
| **PBE1PBE-D3BJ** | 0.112 | 0.115 | **0.087** | **0.087** | 0.206 | **0.084** | **0.088** | 0.102 | **0.084** | **0.088** | 0.102 | **0.091** | 0.246 | **0.074** | 0.158 | **0.082** | 0.104 | **0.090** | **0.086** | **0.087** | **0.108** |
| **BPBE-D3BJ** | 0.762 | 0.762 | 0.987 | 0.987 | 0.502 | 0.880 | 0.859 | 0.799 | 0.880 | 0.860 | 0.797 | 0.888 | 0.435 | 0.877 | 0.654 | 1.036 | 0.875 | 1.120 | 0.978 | 1.110 | **0.852** |
| **B3PW91-D3BJ** | 0.543 | 0.541 | 0.788 | 0.788 | 0.345 | 0.610 | 0.609 | 0.564 | 0.639 | 0.609 | 0.563 | 0.646 | 0.297 | 0.666 | 0.452 | 0.799 | 0.568 | 0.828 | 0.815 | 0.868 | **0.627** |
| **BMK-D3BJ** | 0.904 | 0.905 | 1.207 | 1.215 | 0.848 | 0.904 | 0.918 | 0.887 | 0.905 | 0.914 | 0.887 | 1.048 | 0.528 | 0.851 | 0.827 | 1.036 | 1.125 | 1.062 | 1.067 | 1.147 | **0.959** |
| **CAM-B3LYP-D3B** | 0.186 | 0.188 | 0.132 | 0.132 | 0.275 | 0.148 | 0.159 | 0.162 | 0.148 | 0.160 | 0.163 | 0.150 | 0.367 | 0.143 | 0.226 | 0.136 | 0.165 | 0.133 | 0.135 | 0.130 | **0.172** |
| **LC-wPBE-D3BJ** | **0.079** | **0.078** | 0.220 | 0.220 | 0.162 | 0.230 | 0.181 | 0.172 | 0.230 | 0.190 | 0.177 | 0.225 | 0.206 | 0.227 | 0.127 | 0.212 | 0.131 | 0.234 | 0.218 | 0.262 | **0.189** |
| **B2PLYP** | 0.218 | 0.218 | 0.472 | 0.471 | 0.641 | 0.599 | 0.432 | 0.316 | 0.628 | 0.486 | 0.311 | 1.082 | 0.287 | 0.256 | 0.314 | 0.280 | 0.312 | 0.214 | 0.338 | 0.199 | **0.404** |
| **B2PLYP(Full)** | 0.199 | 0.202 | 0.449 | 0.449 | 0.633 | 0.752 | 0.419 | 0.269 | 0.731 | 0.383 | 0.264 | 0.998 | 0.286 | 0.241 | 0.304 | 0.141 | 0.302 | 0.175 | 0.283 | 0.122 | **0.380** |
| **B2PLYPD** | 0.108 | 0.118 | **0.065** | **0.065** | 0.200 | 0.115 | **0.097** | 0.103 | 0.101 | **0.095** | 0.103 | 0.174 | 0.271 | **0.086** | 0.139 | **0.089** | **0.083** | **0.099** | **0.069** | **0.095** | **0.114** |
| **B2PLYPD(Full)** | 0.109 | 0.118 | **0.065** | **0.065** | 0.200 | 0.115 | **0.096** | 0.108 | **0.098** | **0.096** | 0.107 | 0.174 | 0.270 | **0.085** | 0.138 | 0.112 | **0.084** | 0.105 | **0.074** | 0.116 | **0.117** |
| **B2PLYP-D3** | **0.062** | **0.071** | **0.041** | **0.041** | 0.208 | 0.117 | 0.101 | 0.101 | 0.114 | **0.098** | **0.099** | 0.186 | 0.257 | **0.077** | 0.145 | **0.049** | **0.083** | **0.048** | **0.055** | **0.045** | **0.100** |
| **B2PLYP-D3(Full)** | **0.061** | **0.071** | **0.037** | **0.032** | 0.207 | 0.120 | 0.102 | **0.082** | 0.117 | 0.101 | **0.085** | 0.185 | 0.255 | **0.076** | 0.131 | **0.074** | **0.079** | **0.055** | **0.042** | **0.068** | **0.099** |
| **B2PLYPD3** | **0.038** | **0.046** | **0.053** | **0.053** | 0.187 | 0.183 | **0.089** | **0.099** | 0.155 | **0.083** | **0.095** | 0.267 | 0.242 | **0.053** | 0.127 | **0.014** | **0.062** | **0.008** | **0.024** | **0.008** | **0.094** |
| **B2PLYPD3(Full)** | **0.037** | **0.045** | **0.040** | **0.038** | 0.186 | 0.179 | **0.086** | **0.085** | 0.154 | **0.080** | **0.083** | 0.265 | 0.241 | **0.051** | 0.113 | **0.020** | **0.046** | **0.007** | **0.012** | **0.010** | **0.089** |
| **mPW2PLYP** | 0.188 | 0.191 | 0.167 | 0.166 | 0.291 | 0.200 | 0.200 | 0.203 | 0.199 | 0.199 | 0.203 | 0.214 | 0.334 | 0.191 | 0.235 | 0.177 | 0.184 | 0.177 | 0.169 | 0.175 | **0.203** |
| **mPW2PLYP(Full)** | 0.187 | 0.190 | 0.167 | 0.166 | 0.289 | 0.200 | 0.199 | 0.202 | 0.199 | 0.198 | 0.202 | 0.214 | 0.333 | 0.191 | 0.232 | 0.178 | 0.183 | 0.177 | 0.168 | 0.173 | **0.202** |
| **mPW2PLYPD** | 0.221 | 0.224 | 0.202 | 0.202 | 0.278 | 0.210 | 0.215 | 0.225 | 0.210 | 0.224 | 0.230 | 0.214 | 0.345 | 0.214 | 0.246 | 0.227 | 0.219 | 0.233 | 0.218 | 0.232 | **0.230** |
| **mPW2PLYPD(Full** | 0.226 | 0.229 | 0.204 | 0.206 | 0.278 | 0.210 | 0.220 | 0.227 | 0.211 | 0.229 | 0.236 | 0.214 | 0.347 | 0.218 | 0.251 | 0.241 | 0.222 | 0.239 | 0.223 | 0.242 | **0.234** |
| **PBE0DH** | 0.122 | 0.127 | 0.161 | 0.161 | 0.213 | 0.149 | 0.128 | 0.112 | 0.145 | 0.126 | 0.106 | 0.208 | 0.232 | 0.137 | 0.138 | 0.118 | 0.129 | 0.134 | 0.146 | 0.131 | **0.146** |
| **PBE0DH(Full)** | 0.117 | 0.123 | 0.156 | 0.155 | 0.211 | 0.148 | 0.124 | 0.104 | 0.142 | 0.121 | 0.100 | 0.207 | 0.231 | 0.134 | 0.134 | 0.100 | 0.127 | 0.127 | 0.139 | 0.117 | **0.141** |
| **DSDPBEP86** | **0.018** | **0.029** | **0.037** | **0.035** | 0.139 | **0.052** | **0.036** | **0.058** | **0.048** | **0.038** | **0.060** | 0.179 | 0.182 | **0.041** | **0.083** | **0.028** | **0.016** | **0.026** | **0.013** | **0.029** | **0.057** |
| **DSDPBEP86(Full)** | **0.018** | **0.027** | **0.029** | **0.025** | 0.137 | **0.050** | **0.040** | **0.061** | **0.040** | **0.045** | **0.066** | 0.163 | 0.180 | **0.036** | **0.078** | **0.072** | **0.018** | **0.041** | **0.014** | **0.056** | **0.060** |
| **revDSDPBEP86** | **0.032** | **0.039** | **0.075** | **0.078** | 0.150 | **0.067** | **0.049** | **0.055** | **0.058** | **0.043** | **0.055** | 0.193 | 0.183 | **0.056** | **0.092** | **0.015** | **0.025** | **0.010** | **0.034** | **0.017** | **0.066** |
| **revDSDPBEP86(F** | **0.023** | **0.035** | **0.061** | **0.059** | 0.144 | **0.064** | **0.046** | **0.060** | **0.054** | **0.036** | **0.060** | 0.179 | 0.182 | **0.047** | **0.082** | **0.049** | **0.018** | **0.019** | **0.022** | **0.033** | **0.064** |
| **PBEQIDH** | 0.130 | 0.128 | 0.175 | 0.172 | 0.212 | 0.184 | 0.135 | 0.107 | 0.173 | 0.122 | **0.095** | 0.329 | 0.190 | 0.127 | 0.128 | **0.084** | 0.138 | 0.103 | 0.141 | **0.098** | **0.149** |
| **PBEQIDH(Full)** | 0.117 | 0.115 | 0.160 | 0.158 | 0.209 | 0.177 | 0.128 | **0.091** | 0.164 | 0.110 | **0.081** | 0.316 | 0.192 | 0.121 | 0.118 | **0.058** | 0.131 | **0.088** | 0.123 | **0.058** | **0.136** |
| **MP2** | 0.219 | 0.188 | 0.203 | 0.198 | 0.283 | 0.273 | 0.150 | **0.091** | 0.241 | 0.120 | **0.076** | 0.636 | 0.207 | 0.138 | 0.178 | **0.057** | 0.144 | **0.061** | 0.153 | **0.063** | **0.184** |
| **MP2(Full)** | 0.182 | 0.149 | 0.165 | 0.158 | 0.271 | 0.253 | 0.130 | **0.067** | 0.224 | 0.106 | **0.055** | 0.599 | 0.201 | 0.117 | 0.133 | **0.079** | 0.123 | **0.064** | 0.130 | **0.096** | **0.165** |
| **MP3** | 0.274 | 0.243 | 0.249 | 0.250 | 0.319 | 0.277 | 0.182 | 0.117 | 0.244 | 0.153 | **0.098** | 0.601 | 0.208 | 0.161 | 0.229 | **0.049** | 0.192 | **0.034** | 0.164 | **0.030** | **0.204** |
| **MP3(Full)** | 0.240 | 0.206 | 0.207 | 0.206 | 0.309 | 0.261 | 0.164 | **0.093** | 0.230 | 0.139 | **0.079** | 0.571 | 0.204 | 0.137 | 0.180 | **0.043** | 0.165 | **0.022** | 0.121 | **0.046** | **0.181** |
| **CISD** | 0.325 | 0.295 | 0.347 | 0.356 | 0.336 | 0.328 | 0.221 | 0.153 | 0.312 | 0.202 | 0.137 | 0.594 | 0.222 | 0.193 | 0.276 | **0.098** | 0.268 | 0.104 | 0.260 | 0.101 | **0.256** |
| **CISD(Full)** | 0.289 | 0.261 | 0.312 | 0.317 | 0.338 | 0.334 | 0.217 | 0.131 | 0.317 | 0.195 | 0.119 | 0.574 | 0.209 | 0.174 | 0.226 | **0.042** | 0.241 | **0.076** | 0.224 | **0.047** | **0.232** |
| **CCSD** | 0.282 | 0.253 | 0.272 | 0.270 | 0.317 | 0.292 | 0.188 | 0.121 | 0.271 | 0.162 | 0.102 | 0.599 | 0.220 | 0.163 | 0.242 | **0.061** | 0.209 | **0.052** | 0.190 | **0.044** | **0.216** |
| **CCSD(Full)** | 0.245 | 0.214 | 0.228 | 0.225 | 0.309 | 0.274 | 0.169 | **0.092** | 0.254 | 0.145 | **0.078** | 0.558 | 0.213 | 0.139 | 0.187 | **0.038** | 0.178 | **0.025** | 0.142 | **0.033** | **0.187** |
| **QCISD** | 0.280 | 0.251 | 0.272 | 0.270 | 0.316 | 0.293 | 0.189 | 0.120 | 0.266 | 0.162 | 0.101 | 0.601 | 0.219 | 0.160 | 0.241 | **0.060** | 0.206 | **0.050** | 0.189 | **0.043** | **0.214** |
| **QCISD(Full)** | 0.243 | 0.212 | 0.228 | 0.228 | 0.309 | 0.275 | 0.168 | **0.091** | 0.249 | 0.144 | **0.080** | 0.570 | 0.214 | 0.136 | 0.186 | **0.038** | 0.176 | **0.025** | 0.140 | **0.033** | **0.187** |
| **BD** | 0.249 | 0.209 | 0.207 | 0.215 | 0.303 | 0.280 | 0.162 | **0.090** | 0.241 | 0.143 | **0.071** | 0.585 | 0.212 | 0.129 | 0.185 | **0.030** | 0.160 | **0.019** | **0.097** | **0.022** | **0.180** |
| **BD(Full)** | 0.249 | 0.209 | 0.207 | 0.212 | 0.303 | 0.280 | 0.162 | **0.090** | 0.241 | 0.143 | **0.071** | 0.585 | 0.212 | 0.129 | 0.185 | **0.030** | 0.160 | **0.019** | **0.097** | **0.022** | **0.180** |
| **MP4** | 0.244 | 0.211 | 0.229 | 0.203 | 0.298 | 0.262 | 0.156 | **0.098** | 0.225 | 0.123 | **0.071** | 0.573 | 0.216 | 0.129 | 0.200 | **0.031** | 0.128 | **0.016** | 0.115 | **0.027** | **0.178** |
| **MP4(Full)** | 0.182 | 0.161 | 0.163 | 0.160 | 0.278 | 0.225 | 0.110 | **0.062** | 0.183 | **0.077** | **0.049** | 0.536 | 0.212 | **0.099** | 0.121 | **0.061** | 0.111 | **0.025** | **0.083** | **0.070** | **0.148** |
| **CCSD(T)** | 0.250 | 0.215 | 0.194 | 0.167 | 0.299 | 0.260 | 0.154 | **0.098** | 0.226 | 0.126 | **0.072** | 0.568 | 0.214 | 0.117 | 0.205 | **0.030** | 0.134 | **0.023** | 0.107 | **0.026** | **0.174** |
| **CCSD(T)(Full)** | 0.201 | 0.167 | 0.164 | 0.166 | 0.278 | 0.244 | 0.110 | **0.056** | 0.204 | **0.078** | **0.049** | 0.532 | 0.210 | **0.099** | 0.141 | **0.060** | 0.117 | **0.025** | **0.083** | **0.060** | **0.152** |

| | | | | | | | | | | | | | | | | | | | | | |
|---|---|---|---|---|---|---|---|---|---|---|---|---|---|---|---|---|---|---|---|---|---|
| **QCISD(T)** | 0.249 | 0.215 | 0.198 | 0.172 | 0.298 | 0.260 | 0.154 | **0.098** | 0.226 | 0.125 | **0.072** | 0.565 | 0.214 | 0.115 | 0.205 | **0.030** | 0.133 | **0.023** | 0.105 | **0.028** | **0.174** |
| **QCISD(T)(Full)** | 0.199 | 0.166 | 0.163 | 0.168 | 0.278 | 0.235 | 0.110 | **0.077** | 0.194 | **0.078** | **0.050** | 0.530 | 0.210 | **0.098** | 0.141 | **0.061** | 0.115 | **0.026** | **0.084** | **0.053** | **0.152** |
| **BD(T)** | 0.207 | 0.186 | 0.187 | 0.150 | 0.251 | 0.224 | 0.121 | **0.048** | 0.187 | **0.067** | **0.038** | 0.508 | 0.100 | **0.063** | 0.157 | **0.008** | **0.077** | 0.013 | **0.098** | 0.016 | **0.135** |
| **BD(T)(Full)** | 0.180 | 0.111 | 0.182 | 0.178 | 0.310 | 0.235 | 0.129 | **0.066** | 0.271 | 0.104 | **0.043** | 0.556 | **0.085** | **0.041** | **0.091** | **0.038** | **0.091** | 0.012 | **0.074** | **0.027** | **0.141** |
| **MP5** | 0.257 | 0.224 | 0.192 | 0.199 | 0.299 | 0.257 | 0.154 | **0.099** | 0.225 | 0.127 | **0.076** | 0.569 | 0.213 | 0.120 | 0.209 | **0.032** | 0.141 | **0.027** | | | **0.190** |
| **MP5(Full)** | 0.213 | 0.177 | 0.180 | 0.176 | 0.276 | 0.231 | 0.441 | **0.063** | 0.201 | **0.078** | **0.049** | 0.498 | 0.208 | 0.101 | 0.150 | **0.058** | 0.138 | **0.030** | | | **0.182** |
| **Ave** | **0.378** | **0.377** | **0.538** | **0.538** | **0.419** | **0.486** | **0.454** | **0.428** | **0.484** | **0.451** | **0.427** | **0.502** | **0.408** | **0.480** | **0.405** | **0.495** | **0.443** | **0.538** | **0.532** | **0.558** | **0.467** |
| **PM7** | 0.528 | | | | | | | | | | | | | | | | | | | | |
| **PM6** | 0.567 | | | | | | | | | | | | | | | | | | | | |
| **Dreiding** | **0.000** | | | | | | | | | | | | | | | | | | | | |
| **UFF** | **0.094** | | | | | | | | | | | | | | | | | | | | |

[a] A blank means that this method/basis set has not been used to calculate these 11-RG-Mols, so cc-pV5Z and aug-cc-pV5Z have not been calculated for MP5 and MP5(Full). For instance, the consumed memories of even MP5(Full)/aug-cc-pVTZ calculating $^{84}Kr_2$, MP5(Full)/6-311++G(3df,3pd) calculating $^{84}Kr_2$, MP5(Full)/cc-pVQZ calculating $^{84}Kr_2$, MP5(Full)/Def2QZVP calculating $^{40}Ar^{84}Kr$, MP5(Full)/aug-cc-pVQZ calculating $^{20}Ne^{84}Kr$, MP5(Full)/aug-cc-pVQZ calculating $^{40}Ar^{84}Kr$, MP3/UGBS1V++ calculating $^{20}Ne^{84}Kr$, and MP3/UGBS1V++ calculating $^{84}Kr_2$ are respectively as large as ～730 GB, ～1.1 TB, ～1.4 TB, ～920 GB, ～1.2 TB, ～1.9 TB, ～1.2 TB, and ～3.1 TB (the latter two are used in Table S5), so some calculations are now impossible.

[b] The definition of 14 special methods are as follows: (1) BB1K/6-311++G**: BB95/6-311++G** Iop(3/76=0580004200); (2) mPW1K/6-311++G**: mPWPW91/6-311++G** Iop(3/76=0572004280); (3) mPW1B95/6-311++G**: mPWB95/6-311++G** Iop(3/76=0690003100); (4) mPWB1K/6-311++G**: mPWB95/6-311++G** Iop(3/76=0560004400); (5) mPW1KCIS/6-311++G**: mPWKCIS/6-311++G** Iop(3/76=0850001500); (6) mPWKCIS1K/6-311++G**: mPWKCIS/6-311++G** Iop(3/76=0590004100); (7) TPSS1KCIS/6-311++G**: TPSSKCIS/6-311++G** Iop(3/76=0870001300); (8) PBE1KCIS/6-311++G**: PBEKCIS/6-311++G** Iop(3/76=0780002200); (9) mPWLYP1M/6-311++G**: mPWLYP/6-311++G** Iop(3/76=0950000500); (10) PBE1W/6-311++G**: PBEPBE/6-311++G** Iop(3/78=0740010000); (11) mPWLYP1W/6-311++G**: mPWV5LYP/6-311++G** Iop(3/78=0880010000); (12) PBELYP1W/6-311++G**: PBEV5LYP/6-311++G** Iop(3/78=0540010000); (13) TPSSLYP1W/6-311++G**: TPSSV5LYP/6-311++G** Iop(3/78=0740010000); (14) mPW3LYP/6-311++G**: mPWLYP/6-311++G** Iop(3/76=1000002000) Iop(3/77=0720008000) Iop(3/78=0810010000).

[c] B2PLYP-D3 is different from B2PLYPD3 in this work, i.e., B2PLYPD3 is D3BJ dispersion corrected by definition (or by adding keyword “EmpiricalDispersion=GD3BJ” or “EM=GD3BJ” to B2PLYP), while B2PLYP-D3 is defined by adding keyword “EM=GD3” to B2PLYP or is D3 dispersion corrected.

[d] The definition of e.g. revDSDPBEP86/6-311++G** is: DSDPBEP86/6-311++G** EM=GD3BJ Iop(3/125=0079905785, 3/78=0429604296, 3/76=0310006900, 3/74=1004, 3/174=0437700, 3/175=-1, 3/176=0, 3/177=-1, 3/178=5500000).

[e] A green or blue value means, respectively, that this MAD is smaller than 0.020 Å (with the one less than 0.010 Å slightly different) or is between 0.020～0.100 Å, while a black one means that this value is larger than 0.100 Å in which the largest one is shown with red.

[f] For some molecules optimized by post-MP3 methods, Gaussian 09 [S1] have to be used, especially for BD(T) and BD(T)(Full), because their successful optimization by Gaussian 16 cannot be attained even after 30 times. Moreover, for some molecules, both Gaussian 16 and Gaussian 09 cannot optimize them. For example, for the several method/basis sets listed in the text, i.e., BD(T)/aug-cc-pVTZ, BD(T)/aug-cc-pVQZ, BD(T)/aug-cc-pV5Z, BD(T)(Full)/aug-cc-pVQZ, MP4/aug-cc-pVQZ, and CCSD(T)/aug-cc-pVQZ, their numbers of the 11-RG-Mols successfully optimized only by Gaussian 09 are respectively 6 (i.e, $^{84}Kr_2$, HeAr, HeKr, $^{20}Ne^{40}Ar$, $^{22}Ne^{36}Ar$, and $^{20}Ne^{84}Kr$), 5 (i.e., $Ne_2$, $^{84}Kr_2$, $^{20}Ne^{40}Ar$, $^{22}Ne^{36}Ar$, and $^{20}Ne^{84}Kr$), 5 (i.e., $^{84}Kr_2$, HeNe, $^{20}Ne^{40}Ar$, $^{22}Ne^{36}Ar$, and $^{20}Ne^{84}Kr$), 3 (i.e., $^{4}Kr_2$, HeNe, and $^{20}Ne^{84}Kr$), 0, and 0, in which HeKr cannot be successfully optimized by MP4/aug-cc-pVQZ. Not only HeKr and $^{20}Ne^{84}Kr$ are successfully optimized by CCSD(T)/aug-cc-pV5Z only using Gaussian 09, but also $^{84}Kr_2$ cannot be successfully optimized by CCSD(T)/aug-cc-pV5 and CCSD(T)(Full)/aug-cc-pV5. Only three molecules

(i.e., $He_2$, HeNe, and $Ne_2$) can be calculated by Dreiding, and 4 molecules containing He cannot be optimized by UFF. For the other methods and basis sets, all the 11 molecules are successfully optimized by Gaussian 16 except LC-N12 which has optimized 6 molecules (i.e., the 6 molecules that do not contain Ne).

Table S3. MDs (Å) of 11-RG-Mols calculated by 572 methods and 20 basis sets.

| | BS01 | BS02 | BS03 | BS04 | BS05 | BS06 | BS07 | BS08 | BS09 | BS10 | BS11 | BS12 | BS13 | BS14 | BS15 | BS16 | BS17 | BS18 | BS19 | BS20 | Ave |
|---|---|---|---|---|---|---|---|---|---|---|---|---|---|---|---|---|---|---|---|---|---|
| **HF** | 0.791 | 0.758 | 1.309 | 1.309 | 0.567 | 0.914 | 0.804 | 0.726 | 0.913 | 0.804 | 0.726 | 0.855 | 0.345 | 0.918 | 0.665 | 1.105 | 1.013 | 1.259 | 1.308 | 1.308 | **0.920** |
| **APFD** | -0.094 | -0.100 | -0.005 | -0.005 | -0.178 | -0.005 | -0.045 | -0.054 | -0.006 | -0.047 | -0.056 | 0.006 | -0.271 | -0.005 | -0.127 | -0.016 | -0.053 | -0.003 | -0.012 | -0.007 | **-0.054** |
| **wB97** | -0.147 | -0.159 | 0.337 | 0.338 | -0.220 | 0.149 | 0.051 | -0.011 | 0.148 | 0.081 | 0.015 | 0.170 | -0.355 | 0.050 | -0.152 | 0.040 | 0.131 | 0.282 | 0.283 | 0.350 | **0.069** |
| **wB97X** | -0.102 | -0.109 | 0.001 | 0.001 | -0.182 | -0.008 | -0.041 | -0.065 | -0.009 | -0.043 | -0.066 | -0.003 | -0.286 | -0.034 | -0.145 | -0.066 | -0.050 | -0.024 | -0.007 | 0.002 | **-0.062** |
| **wB97XD** | 0.294 | 0.292 | 0.381 | 0.381 | 0.192 | 0.355 | 0.335 | 0.319 | 0.355 | 0.334 | 0.319 | 0.357 | 0.109 | 0.343 | 0.251 | 0.370 | 0.333 | 0.393 | 0.378 | 0.393 | **0.324** |
| **B98** | 0.014 | 0.010 | 0.087 | 0.087 | -0.079 | 0.077 | 0.044 | 0.036 | 0.076 | 0.043 | 0.035 | 0.089 | -0.177 | 0.088 | -0.033 | 0.076 | 0.041 | 0.085 | 0.085 | 0.085 | **0.039** |
| **B971** | -0.089 | -0.091 | -0.028 | -0.028 | -0.161 | -0.031 | -0.058 | -0.067 | -0.033 | -0.059 | -0.069 | -0.021 | -0.254 | -0.029 | -0.120 | -0.034 | -0.061 | -0.034 | -0.031 | -0.031 | **-0.066** |
| **B972** | 0.367 | 0.359 | 0.495 | 0.495 | 0.228 | 0.471 | 0.431 | 0.397 | 0.471 | 0.429 | 0.396 | 0.484 | 0.099 | 0.456 | 0.299 | 0.507 | 0.401 | 0.514 | 0.494 | 0.506 | **0.415** |
| **BHandH** | -0.527 | -0.528 | -0.519 | -0.519 | -0.555 | -0.521 | -0.534 | -0.532 | -0.523 | -0.536 | -0.534 | -0.507 | -0.610 | -0.540 | -0.534 | -0.525 | -0.523 | -0.522 | -0.521 | -0.522 | **-0.532** |
| **BHandHLYP** | 0.023 | 0.022 | 0.485 | 0.484 | -0.020 | 0.527 | 0.111 | 0.079 | 0.448 | 0.096 | 0.076 | 0.316 | -0.243 | 0.144 | 0.178 | 0.483 | 0.450 | 0.539 | 0.530 | 0.532 | **0.263** |
| **HFS** | -0.359 | -0.362 | -0.311 | -0.311 | -0.401 | -0.295 | -0.324 | -0.326 | -0.297 | -0.327 | -0.329 | -0.274 | -0.498 | -0.322 | -0.379 | -0.320 | -0.334 | -0.313 | -0.316 | -0.309 | **-0.335** |
| **HFB** | 1.977 | 1.983 | 2.233 | 2.234 | 1.485 | 2.159 | 2.160 | 2.112 | 2.231 | 2.185 | 2.109 | 2.163 | 1.504 | 2.282 | 1.831 | 2.262 | 2.061 | 2.374 | 2.287 | 2.521 | **2.108** |
| **XAlpha** | -0.412 | -0.414 | -0.378 | -0.378 | -0.446 | -0.363 | -0.390 | -0.391 | -0.366 | -0.393 | -0.394 | -0.340 | -0.533 | -0.391 | -0.427 | -0.387 | -0.393 | -0.380 | -0.381 | -0.378 | **-0.397** |
| **LSDA** | -0.504 | -0.506 | -0.488 | -0.488 | -0.526 | -0.473 | -0.497 | -0.497 | -0.477 | -0.500 | -0.501 | -0.449 | -0.595 | -0.502 | -0.514 | -0.496 | -0.496 | -0.491 | -0.491 | -0.491 | **-0.499** |
| **LC-LSDA** | 0.159 | 0.148 | 0.396 | 0.396 | 0.019 | 0.354 | 0.294 | 0.266 | 0.355 | 0.294 | 0.265 | 0.355 | -0.138 | 0.355 | 0.121 | 0.325 | 0.267 | 0.408 | 0.394 | 0.419 | **0.272** |
| **HCTH** | -0.119 | -0.120 | -0.078 | -0.078 | -0.182 | -0.083 | -0.092 | -0.094 | -0.084 | -0.093 | -0.095 | -0.077 | -0.240 | -0.057 | -0.141 | -0.071 | -0.100 | -0.080 | -0.077 | -0.080 | **-0.102** |
| **HCTH93** | 0.853 | 0.850 | 1.098 | 1.099 | 0.612 | 1.097 | 1.080 | 1.022 | 1.093 | 1.083 | 1.024 | 1.065 | 0.479 | 0.951 | 0.735 | 1.179 | 0.882 | 1.195 | 1.184 | 1.256 | **0.992** |
| **HCTH147** | 0.114 | 0.110 | 0.192 | 0.192 | 0.009 | 0.187 | 0.163 | 0.155 | 0.186 | 0.162 | 0.154 | 0.194 | -0.071 | 0.204 | 0.070 | 0.205 | 0.135 | 0.198 | 0.189 | 0.190 | **0.147** |
| **tHCTH** | 0.092 | 0.090 | 0.135 | 0.135 | -0.004 | 0.126 | 0.109 | 0.102 | 0.126 | 0.108 | 0.102 | 0.132 | -0.089 | 0.136 | 0.049 | 0.150 | 0.092 | 0.142 | 0.130 | 0.126 | **0.099** |
| **tHCTHhyb** | 0.205 | 0.198 | 0.307 | 0.307 | 0.079 | 0.284 | 0.253 | 0.239 | 0.283 | 0.251 | 0.238 | 0.300 | -0.014 | 0.291 | 0.143 | 0.315 | 0.228 | 0.309 | 0.296 | 0.300 | **0.241** |
| **VSXC** | -0.176 | -0.176 | -0.132 | -0.132 | -0.147 | -0.139 | -0.177 | -0.178 | -0.140 | -0.178 | -0.179 | -0.137 | -0.049 | -0.174 | -0.202 | -0.165 | -0.138 | -0.159 | -0.134 | -0.159 | **-0.153** |
| **BMK** | 1.669 | 1.667 | 1.810 | 1.881 | 1.411 | 1.494 | 1.497 | 1.419 | 1.515 | 1.458 | 1.447 | 1.536 | 1.097 | 1.682 | 1.676 | 1.605 | 1.746 | 1.719 | 1.866 | 1.969 | **1.608** |
| **TPSSh** | 0.189 | 0.187 | 0.271 | 0.270 | 0.067 | 0.261 | 0.236 | 0.225 | 0.259 | 0.235 | 0.225 | 0.269 | -0.047 | 0.269 | 0.137 | 0.299 | 0.211 | 0.304 | 0.267 | 0.270 | **0.220** |
| **APF** | 0.507 | 0.470 | 0.835 | 0.835 | 0.360 | 0.797 | 0.699 | 0.606 | 0.799 | 0.725 | 0.602 | 0.800 | 0.190 | 0.738 | 0.417 | 0.907 | 0.702 | 0.908 | 0.846 | 0.898 | **0.682** |
| **OAPF** | 0.103 | 0.097 | 0.243 | 0.245 | -0.002 | 0.236 | 0.170 | 0.154 | 0.235 | 0.168 | 0.151 | 0.247 | -0.146 | 0.242 | 0.061 | 0.254 | 0.173 | 0.247 | 0.241 | 0.244 | **0.168** |
| **GVB(0)** | 0.768 | 0.768 | 1.287 | 1.287 | 0.574 | 0.923 | 0.792 | 0.718 | 0.923 | 0.792 | 0.717 | 0.865 | 0.335 | 0.897 | 0.664 | 1.090 | 1.016 | 1.247 | 1.285 | 1.276 | **0.911** |
| **B97D** | 0.059 | 0.055 | 0.147 | 0.147 | -0.041 | 0.163 | 0.139 | 0.132 | 0.162 | 0.138 | 0.131 | 0.173 | -0.119 | 0.158 | 0.015 | 0.174 | 0.078 | 0.159 | 0.142 | 0.137 | **0.108** |
| **B97D3** | 0.114 | 0.109 | 0.199 | 0.200 | 0.000 | 0.209 | 0.172 | 0.162 | 0.208 | 0.172 | 0.161 | 0.226 | -0.078 | 0.210 | 0.061 | 0.224 | 0.138 | 0.213 | 0.196 | 0.198 | **0.155** |
| **B3LYP** | 0.982 | 1.057 | 1.864 | 1.910 | 0.853 | 1.416 | 1.190 | 1.069 | 1.380 | 1.195 | 1.074 | 1.349 | 0.282 | 1.142 | 0.981 | 1.772 | 1.174 | 1.910 | 1.828 | 1.990 | **1.321** |
| **B3P86** | 1.725 | 1.698 | 2.019 | 2.020 | 1.307 | 1.734 | 1.661 | 1.580 | 1.697 | 1.656 | 1.581 | 1.737 | 1.363 | 1.767 | 1.721 | 1.861 | 1.807 | 2.106 | 2.034 | 2.151 | **1.761** |
| **B3PW91** | 1.676 | 1.654 | 2.031 | 2.010 | 1.227 | 1.650 | 1.611 | 1.530 | 1.627 | 1.611 | 1.536 | 1.651 | 1.359 | 1.588 | 1.587 | 1.826 | 1.749 | 2.059 | 1.966 | 2.077 | **1.701** |
| **X3LYP** | -0.005 | -0.010 | 0.107 | 0.107 | -0.075 | 0.104 | 0.067 | 0.052 | 0.111 | 0.066 | 0.050 | 0.138 | -0.252 | 0.101 | -0.025 | 0.121 | 0.054 | 0.097 | 0.105 | 0.108 | **0.051** |
| **O3LYP** | 0.236 | 0.231 | 0.381 | 0.381 | 0.107 | 0.380 | 0.355 | 0.340 | 0.380 | 0.357 | 0.340 | 0.382 | -0.039 | 0.332 | 0.185 | 0.432 | 0.292 | 0.399 | 0.391 | 0.392 | **0.313** |
| **B1LYP** | 1.142 | 0.975 | 1.803 | 1.828 | 0.823 | 1.444 | 1.186 | 1.132 | 1.472 | 1.211 | 1.146 | 1.439 | 0.407 | 1.255 | 1.030 | 1.724 | 1.274 | 2.103 | 1.741 | 2.047 | **1.359** |
| **B1B95** | 0.207 | 0.207 | 0.273 | 0.273 | 0.204 | 0.406 | 0.362 | 0.297 | 0.406 | 0.362 | 0.297 | 0.427 | 0.184 | 0.230 | 0.174 | 0.444 | 0.246 | 0.276 | 0.241 | 0.311 | **0.291** |
| **OmPW1LYP** | -0.175 | -0.176 | -0.117 | -0.117 | -0.244 | -0.126 | -0.145 | -0.146 | -0.127 | -0.146 | -0.147 | -0.114 | -0.355 | -0.125 | -0.199 | -0.117 | -0.141 | -0.115 | -0.117 | -0.115 | **-0.153** |
| **LG1LYP** | -0.316 | -0.316 | -0.283 | -0.283 | -0.363 | -0.282 | -0.298 | -0.297 | -0.283 | -0.300 | -0.299 | -0.270 | -0.444 | -0.284 | -0.331 | -0.284 | -0.295 | -0.286 | -0.284 | -0.284 | **-0.304** |
| **mPW1LYP** | -0.151 | -0.152 | -0.087 | -0.087 | -0.225 | -0.098 | -0.119 | -0.121 | -0.099 | -0.121 | -0.122 | -0.086 | -0.341 | -0.095 | -0.178 | -0.088 | -0.114 | -0.085 | -0.087 | -0.084 | **-0.127** |
| **mPW1PW91** | 0.376 | 0.369 | 0.511 | 0.511 | 0.223 | 0.505 | 0.461 | 0.405 | 0.504 | 0.459 | 0.404 | 0.501 | 0.056 | 0.440 | 0.295 | 0.540 | 0.420 | 0.532 | 0.512 | 0.523 | **0.427** |
| **mPW1PBE** | 0.385 | 0.377 | 0.519 | 0.519 | 0.232 | 0.512 | 0.468 | 0.425 | 0.509 | 0.467 | 0.427 | 0.511 | 0.066 | 0.450 | 0.303 | 0.550 | 0.431 | 0.542 | 0.517 | 0.533 | **0.437** |
| **mPW3PBE** | 0.310 | 0.301 | 0.461 | 0.461 | 0.168 | 0.439 | 0.391 | 0.363 | 0.439 | 0.390 | 0.362 | 0.444 | 0.011 | 0.398 | 0.245 | 0.475 | 0.353 | 0.486 | 0.458 | 0.477 | **0.372** |
| **PBE1PBE** | 0.026 | 0.021 | 0.114 | 0.114 | -0.074 | 0.106 | 0.062 | 0.050 | 0.099 | 0.060 | 0.048 | 0.117 | -0.176 | 0.108 | -0.021 | 0.100 | 0.063 | 0.112 | 0.109 | 0.112 | **0.057** |
| **PBEh1PBE** | 0.018 | 0.013 | 0.104 | 0.104 | -0.079 | 0.086 | 0.053 | 0.041 | 0.085 | 0.052 | 0.038 | 0.099 | -0.181 | 0.104 | -0.027 | 0.092 | 0.054 | 0.098 | 0.101 | 0.102 | **0.048** |
| **HSEh1PBE** | 0.003 | 0.000 | 0.086 | 0.086 | -0.088 | 0.072 | 0.037 | 0.024 | 0.071 | 0.036 | 0.022 | 0.083 | -0.188 | 0.083 | -0.038 | 0.071 | 0.040 | 0.083 | 0.079 | 0.083 | **0.032** |
| **OHSE1PBE** | 0.004 | 0.000 | 0.090 | 0.090 | -0.088 | 0.073 | 0.038 | 0.024 | 0.071 | 0.036 | 0.023 | 0.083 | -0.189 | 0.083 | -0.037 | 0.071 | 0.042 | 0.083 | 0.083 | 0.086 | **0.033** |
| **OHSE2PBE** | -0.018 | -0.021 | 0.054 | 0.054 | -0.104 | 0.041 | 0.006 | -0.001 | 0.040 | 0.005 | -0.003 | 0.053 | -0.198 | 0.056 | -0.056 | 0.045 | 0.009 | 0.048 | 0.051 | 0.050 | **0.006** |
| **HISSbPBE** | 0.021 | 0.017 | 0.095 | 0.095 | -0.081 | 0.081 | 0.046 | 0.034 | 0.080 | 0.045 | 0.033 | 0.090 | -0.183 | 0.080 | -0.031 | 0.073 | 0.054 | 0.088 | 0.091 | 0.090 | **0.041** |
| **M06** | 0.051 | 0.051 | 0.138 | 0.138 | 0.009 | 0.059 | 0.056 | 0.056 | 0.059 | 0.056 | 0.055 | 0.060 | -0.030 | 0.040 | 0.036 | 0.058 | 0.160 | 0.166 | 0.137 | 0.150 | **0.075** |
| **M06L** | 0.104 | 0.104 | 0.131 | 0.131 | 0.049 | 0.103 | 0.076 | 0.049 | 0.103 | 0.076 | 0.049 | 0.105 | -0.053 | 0.067 | 0.088 | 0.048 | 0.129 | 0.087 | 0.102 | 0.064 | **0.081** |
| **M06HF** | 0.111 | 0.111 | 0.123 | 0.123 | 0.072 | 0.122 | 0.118 | 0.115 | 0.122 | 0.118 | 0.115 | 0.123 | -0.028 | 0.090 | 0.103 | 0.120 | 0.120 | 0.125 | 0.120 | 0.090 | **0.106** |
| **M062X** | 0.055 | 0.054 | 0.126 | 0.126 | -0.040 | 0.088 | 0.070 | 0.065 | 0.088 | 0.069 | 0.065 | 0.091 | -0.256 | -0.029 | 0.011 | 0.084 | 0.100 | 0.127 | 0.126 | 0.151 | **0.058** |
| **M05** | -0.193 | -0.196 | -0.162 | -0.162 | -0.276 | -0.181 | -0.191 | -0.188 | -0.183 | -0.192 | -0.189 | -0.172 | -0.387 | -0.247 | -0.220 | -0.150 | -0.173 | -0.154 | -0.158 | -0.152 | **-0.196** |
| **M052X** | -0.123 | -0.123 | -0.092 | -0.092 | -0.203 | -0.122 | -0.133 | -0.133 | -0.123 | -0.134 | -0.134 | -0.116 | -0.285 | -0.116 | -0.164 | -0.118 | -0.096 | -0.090 | -0.093 | -0.094 | **-0.129** |

| | | | | | | | | | | | | | | | | | | | | | |
|---|---|---|---|---|---|---|---|---|---|---|---|---|---|---|---|---|---|---|---|---|---|
| **PW6B95** | 0.026 | 0.026 | 0.063 | 0.063 | -0.128 | 0.057 | 0.055 | 0.042 | 0.057 | 0.055 | 0.042 | 0.085 | -0.177 | 0.071 | -0.026 | 0.060 | 0.048 | 0.062 | 0.063 | 0.064 | **0.030** |
| **PW6B95D3** | -0.017 | -0.017 | 0.031 | 0.031 | -0.160 | 0.024 | 0.022 | 0.010 | 0.024 | 0.022 | 0.010 | 0.026 | -0.182 | 0.039 | -0.059 | 0.027 | 0.016 | 0.031 | 0.031 | 0.032 | **-0.003** |
| **M08HX** | -0.106 | -0.127 | 0.033 | 0.033 | -0.208 | -0.058 | -0.067 | -0.068 | -0.059 | -0.068 | -0.069 | -0.051 | -0.332 | -0.204 | -0.140 | -0.099 | -0.039 | 0.038 | 0.038 | 0.101 | **-0.073** |
| **M11** | 0.087 | 0.087 | 0.153 | 0.153 | -0.058 | 0.126 | 0.122 | 0.118 | 0.126 | 0.122 | 0.118 | 0.128 | -0.229 | 0.064 | 0.001 | 0.095 | 0.138 | 0.144 | 0.153 | 0.159 | **0.090** |
| **M11L** | 0.699 | 0.731 | 0.970 | 0.970 | 0.623 | 0.801 | 0.773 | 0.589 | 0.799 | 0.773 | 0.589 | 0.804 | 0.402 | 0.583 | 0.587 | 0.799 | 0.870 | 1.025 | 0.949 | 1.049 | **0.769** |
| **SOGGA11** | -0.377 | -0.377 | -0.310 | -0.310 | -0.171 | -0.333 | -0.337 | -0.343 | -0.333 | -0.337 | -0.343 | -0.329 | 0.458 | -0.277 | -0.349 | -0.343 | -0.336 | -0.351 | -0.315 | -0.335 | **-0.287** |
| **SOGGA11X** | 0.879 | 0.868 | 1.053 | 1.052 | 0.726 | 0.938 | 0.911 | 0.909 | 0.929 | 0.911 | 0.909 | 0.953 | 0.316 | 0.756 | 0.786 | 1.024 | 0.927 | 1.018 | 1.029 | 1.051 | **0.897** |
| **N12** | 0.945 | 0.910 | 1.556 | 1.563 | 0.548 | 1.297 | 1.285 | 1.233 | 1.344 | 1.320 | 1.206 | 1.357 | 0.251 | 1.288 | 0.717 | 1.474 | 1.072 | 1.596 | 1.487 | 1.614 | **1.203** |
| **N12SX** | 0.599 | 0.602 | 1.548 | 1.563 | 0.627 | 1.282 | 0.962 | 0.875 | 1.287 | 0.971 | 0.914 | 1.288 | 0.012 | 0.829 | 0.574 | 1.230 | 0.968 | 1.621 | 1.574 | 1.733 | **1.053** |
| **MN12SX** | 0.285 | 0.285 | 0.798 | 0.798 | 0.191 | 0.445 | 0.404 | 0.360 | 0.446 | 0.403 | 0.340 | 0.449 | -0.153 | 0.280 | 0.122 | 0.320 | 0.617 | 0.915 | 0.764 | 0.893 | **0.448** |
| **MN12L** | 0.792 | 0.731 | 1.051 | 1.051 | 0.611 | 0.844 | 0.815 | 0.769 | 0.845 | 0.815 | 0.769 | 0.813 | 0.040 | 0.452 | 0.609 | 0.816 | 0.874 | 1.157 | 0.955 | 1.259 | **0.803** |
| **MN15** | -0.079 | -0.083 | -0.016 | -0.016 | -0.130 | -0.016 | -0.031 | -0.041 | -0.017 | -0.032 | -0.041 | -0.001 | -0.186 | -0.014 | -0.124 | -0.044 | -0.073 | -0.031 | -0.022 | -0.028 | **-0.051** |
| **MN15L** | -0.005 | -0.015 | 0.018 | 0.018 | -0.077 | 0.008 | 0.002 | -0.001 | 0.008 | 0.001 | -0.010 | 0.030 | -0.101 | -0.012 | -0.077 | -0.008 | 0.008 | 0.011 | 0.018 | 0.015 | **-0.008** |
| **LC-HFS** | 0.468 | 0.457 | 1.062 | 1.062 | 0.307 | 1.002 | 0.941 | 0.861 | 1.001 | 0.940 | 0.852 | 1.008 | 0.036 | 0.910 | 0.417 | 1.058 | 0.722 | 1.184 | 1.080 | 1.200 | **0.828** |
| **LC-HFB** | 0.470 | 0.458 | 1.073 | 1.073 | 0.305 | 1.017 | 0.946 | 0.850 | 1.016 | 0.940 | 0.860 | 0.987 | 0.036 | 0.952 | 0.417 | 1.125 | 0.725 | 1.161 | 1.070 | 1.281 | **0.838** |
| **LC-XAlpha** | 0.472 | 0.460 | 1.047 | 1.047 | 0.299 | 0.981 | 0.923 | 0.814 | 1.004 | 0.929 | 0.813 | 0.945 | 0.034 | 0.916 | 0.421 | 1.094 | 0.714 | 1.093 | 1.048 | 1.200 | **0.813** |
| **LC-M06L** | -0.722 | -0.723 | -0.712 | -0.712 | -0.734 | -0.712 | -0.722 | -0.723 | -0.713 | -0.723 | -0.724 | -0.703 | -0.673 | -0.712 | -0.721 | -0.718 | -0.718 | -0.719 | -0.716 | -0.718 | **-0.716** |
| **LC-M11L** | 0.699 | 0.731 | 0.971 | 0.971 | 0.623 | 0.801 | 0.773 | 0.589 | 0.799 | 0.773 | 0.589 | 0.804 | 0.402 | 0.583 | 0.587 | 0.799 | 0.870 | 1.025 | 0.948 | 1.051 | **0.769** |
| **LC-N12** | 1.735 | 1.735 | 2.143 | 2.145 | 1.811 | 1.828 | 1.812 | 1.787 | 1.829 | 1.806 | 1.785 | 1.829 | 1.343 | 1.779 | 1.827 | 1.792 | 2.001 | 2.170 | 2.071 | 2.139 | **1.868** |
| **LC-B97D** | -0.658 | -0.658 | -0.651 | -0.651 | -0.672 | -0.648 | -0.652 | -0.651 | -0.648 | -0.653 | -0.652 | -0.641 | -0.705 | -0.648 | -0.659 | -0.651 | -0.653 | -0.651 | -0.651 | -0.651 | **-0.655** |
| **LC-B97D3** | -0.705 | -0.706 | -0.698 | -0.698 | -0.717 | -0.693 | -0.698 | -0.698 | -0.694 | -0.699 | -0.699 | -0.685 | -0.750 | -0.695 | -0.705 | -0.698 | -0.700 | -0.699 | -0.699 | -0.698 | **-0.702** |
| **LC-wPBE** | 0.248 | 0.240 | 0.631 | 0.629 | 0.117 | 0.581 | 0.524 | 0.510 | 0.581 | 0.519 | 0.510 | 0.572 | -0.073 | 0.531 | 0.210 | 0.537 | 0.406 | 0.679 | 0.639 | 0.723 | **0.466** |
| **LC-wHPBE** | 0.244 | 0.238 | 0.671 | 0.669 | 0.117 | 0.576 | 0.521 | 0.461 | 0.576 | 0.521 | 0.463 | 0.553 | -0.073 | 0.559 | 0.208 | 0.555 | 0.405 | 0.675 | 0.631 | 0.718 | **0.464** |
| **CAM-B3LYP** | -0.096 | -0.098 | 0.033 | 0.033 | -0.174 | 0.024 | -0.024 | -0.029 | 0.020 | -0.027 | -0.031 | 0.036 | -0.327 | -0.007 | -0.114 | 0.015 | -0.017 | 0.033 | 0.027 | 0.035 | **-0.034** |
| **LC-HCTH** | -0.558 | -0.558 | -0.541 | -0.541 | -0.579 | -0.538 | -0.541 | -0.540 | -0.538 | -0.542 | -0.541 | -0.532 | -0.623 | -0.534 | -0.559 | -0.537 | -0.546 | -0.540 | -0.540 | -0.539 | **-0.548** |
| **LC-tHCTH** | -0.736 | -0.737 | -0.730 | -0.730 | -0.745 | -0.723 | -0.728 | -0.728 | -0.724 | -0.729 | -0.729 | -0.716 | -0.777 | -0.726 | -0.735 | -0.729 | -0.731 | -0.730 | -0.730 | -0.729 | **-0.732** |
| **LC-XaVP86** | -0.180 | -0.182 | 0.441 | 0.442 | -0.244 | 0.174 | 0.073 | -0.002 | 0.171 | 0.072 | -0.002 | 0.188 | -0.280 | 0.163 | -0.093 | 0.005 | 0.184 | 0.276 | 0.419 | 0.479 | **0.105** |
| **LC-XaVWN** | 0.156 | 0.147 | 0.380 | 0.381 | 0.016 | 0.338 | 0.278 | 0.251 | 0.337 | 0.277 | 0.251 | 0.340 | -0.142 | 0.346 | 0.119 | 0.311 | 0.262 | 0.389 | 0.380 | 0.404 | **0.261** |
| **LC-XaVWN5** | 0.206 | 0.202 | 0.496 | 0.496 | 0.081 | 0.468 | 0.384 | 0.340 | 0.466 | 0.384 | 0.336 | 0.467 | -0.111 | 0.447 | 0.175 | 0.443 | 0.334 | 0.519 | 0.501 | 0.536 | **0.359** |
| **BB95** | 0.549 | 0.721 | 0.636 | 0.636 | 0.499 | 0.693 | 1.040 | 0.634 | 0.705 | 0.598 | 0.650 | 0.593 | 0.335 | 0.464 | 0.654 | 1.383 | 0.836 | 0.874 | 0.657 | 0.646 | **0.690** |
| **BBRC** | 1.819 | 1.815 | 2.206 | 2.206 | 1.368 | 1.949 | 1.951 | 1.964 | 1.950 | 1.952 | 1.954 | 1.976 | 1.460 | 2.034 | 1.833 | 2.234 | 1.885 | 2.232 | 2.216 | 2.336 | **1.967** |
| **BKCIS** | 1.829 | 1.827 | 2.208 | 2.208 | 1.399 | 1.905 | 1.903 | 1.897 | 1.908 | 1.901 | 1.896 | 1.944 | 1.397 | 1.964 | 1.814 | 2.204 | 1.917 | 2.232 | 2.205 | 2.288 | **1.942** |
| **BLYP** | 1.667 | 1.673 | 2.216 | 2.221 | 1.005 | 1.885 | 1.859 | 1.779 | 1.835 | 1.863 | 1.816 | 1.895 | 0.778 | 1.701 | 1.065 | 2.140 | 1.380 | 2.222 | 2.216 | 2.320 | **1.777** |
| **BP86** | 1.891 | 1.891 | 2.219 | 2.219 | 1.692 | 1.977 | 2.007 | 2.001 | 1.999 | 2.007 | 2.005 | 1.978 | 1.425 | 2.099 | 1.891 | 2.164 | 1.936 | 2.248 | 2.227 | 2.390 | **2.013** |
| **BPBE** | 1.801 | 1.822 | 2.169 | 2.164 | 1.398 | 1.906 | 1.873 | 1.822 | 1.875 | 1.872 | 1.867 | 1.938 | 1.519 | 1.962 | 1.845 | 2.143 | 1.869 | 2.232 | 2.165 | 2.341 | **1.929** |
| **BPKZB** | 1.810 | 1.809 | 2.136 | 2.135 | 1.408 | 1.929 | 1.794 | 1.784 | 1.916 | 1.867 | 1.750 | 1.916 | 1.463 | 1.979 | 1.798 | 2.111 | 1.877 | 2.230 | 2.123 | 2.333 | **1.908** |
| **BPL** | 1.818 | 1.811 | 2.125 | 2.121 | 1.365 | 1.902 | 1.896 | 1.826 | 1.905 | 1.860 | 1.786 | 1.934 | 1.423 | 1.802 | 1.757 | 2.105 | 1.866 | 2.227 | 2.114 | 2.205 | **1.892** |
| **BPW91** | 1.822 | 1.821 | 2.149 | 2.139 | 1.467 | 1.946 | 1.867 | 1.774 | 1.943 | 1.871 | 1.759 | 1.946 | 1.461 | 1.985 | 1.794 | 2.112 | 1.920 | 2.223 | 2.109 | 2.301 | **1.921** |
| **BRevTPSS** | 1.815 | 1.794 | 2.190 | 2.201 | 1.442 | 1.918 | 1.883 | 1.801 | 1.924 | 1.915 | 1.768 | 1.918 | 1.490 | 2.002 | 1.770 | 2.133 | 1.888 | 2.239 | 2.238 | 2.256 | **1.929** |
| **BTPSS** | 1.814 | 1.831 | 2.170 | 2.166 | 1.455 | 1.915 | 1.942 | 1.754 | 1.916 | 1.914 | 1.863 | 1.915 | 1.428 | 1.937 | 1.803 | 2.165 | 1.875 | 2.224 | 2.141 | 2.333 | **1.928** |
| **BVP86** | 1.882 | 1.883 | 2.217 | 2.220 | 1.684 | 1.997 | 2.007 | 1.995 | 1.975 | 2.011 | 1.971 | 1.997 | 1.431 | 2.110 | 1.910 | 2.165 | 1.982 | 2.287 | 2.222 | 2.389 | **2.017** |
| **BVWN** | 1.856 | 1.859 | 2.106 | 2.117 | 1.269 | 1.758 | 1.710 | 1.658 | 1.757 | 1.710 | 1.663 | 1.765 | 1.481 | 1.704 | 1.764 | 1.971 | 1.831 | 2.179 | 1.986 | 2.304 | **1.822** |
| **BVWN5** | 1.814 | 1.813 | 2.116 | 2.115 | 1.362 | 1.896 | 1.861 | 1.814 | 1.937 | 1.897 | 1.786 | 1.939 | 1.468 | 1.794 | 1.770 | 2.112 | 1.867 | 2.221 | 2.111 | 2.211 | **1.895** |
| **BRxB95** | 0.136 | 0.136 | 0.168 | 0.168 | 0.078 | 0.199 | 0.141 | 0.139 | 0.198 | 0.141 | 0.139 | 0.200 | 0.045 | 0.170 | 0.135 | 0.176 | 0.140 | 0.144 | 0.168 | 0.143 | **0.148** |
| **BRxBRC** | 1.242 | 1.172 | 1.721 | 1.717 | 0.844 | 1.376 | 1.370 | 1.319 | 1.373 | 1.364 | 1.291 | 1.415 | 0.788 | 1.305 | 1.034 | 1.691 | 1.194 | 1.737 | 1.661 | 1.890 | **1.375** |
| **BRxKCIS** | 1.233 | 1.231 | 1.603 | 1.603 | 0.882 | 1.382 | 1.346 | 1.204 | 1.383 | 1.328 | 1.192 | 1.386 | 0.778 | 1.245 | 0.984 | 1.664 | 1.194 | 1.745 | 1.588 | 1.745 | **1.336** |
| **BRxLYP** | -0.073 | -0.078 | 0.045 | 0.045 | -0.141 | 0.085 | 0.020 | 0.009 | 0.081 | 0.018 | 0.006 | 0.116 | -0.285 | 0.153 | -0.098 | 0.076 | -0.011 | 0.033 | 0.041 | 0.038 | **0.004** |
| **BRxP86** | 1.473 | 1.439 | 2.093 | 2.097 | 1.104 | 1.630 | 1.437 | 1.366 | 1.631 | 1.437 | 1.393 | 1.704 | 0.962 | 1.587 | 0.998 | 1.770 | 1.684 | 2.079 | 2.045 | 2.133 | **1.603** |
| **BRxPBE** | 1.531 | 1.522 | 1.753 | 1.754 | 0.946 | 1.484 | 1.403 | 1.387 | 1.435 | 1.372 | 1.436 | 1.443 | 0.931 | 1.416 | 1.125 | 1.796 | 1.433 | 1.891 | 1.822 | 1.964 | **1.492** |
| **BRxPKZB** | 1.392 | 1.445 | 1.792 | 1.792 | 0.990 | 1.466 | 1.365 | 1.380 | 1.438 | 1.454 | 1.406 | 1.487 | 0.888 | 1.419 | 1.152 | 1.698 | 1.476 | 1.930 | 1.849 | 1.935 | **1.488** |
| **BRxPL** | 1.287 | 1.248 | 1.615 | 1.615 | 0.888 | 1.353 | 1.360 | 1.241 | 1.352 | 1.362 | 1.246 | 1.372 | 0.832 | 1.213 | 1.121 | 1.612 | 1.260 | 1.707 | 1.607 | 1.733 | **1.351** |
| **BRxPW91** | 1.476 | 1.442 | 1.820 | 1.820 | 0.987 | 1.513 | 1.414 | 1.364 | 1.479 | 1.402 | 1.374 | 1.529 | 0.883 | 1.443 | 1.246 | 1.652 | 1.412 | 1.799 | 1.786 | 1.988 | **1.492** |
| **BRxRevTPSS** | 1.386 | 1.371 | 1.935 | 1.899 | 0.993 | 1.480 | 1.476 | 1.395 | 1.447 | 1.496 | 1.392 | 1.485 | 0.829 | 1.377 | 1.160 | 1.658 | 1.335 | 1.882 | 1.741 | 1.920 | **1.483** |
| **BRxTPSS** | 1.446 | 1.445 | 1.830 | 1.829 | 1.035 | 1.472 | 1.454 | 1.409 | 1.469 | 1.408 | 1.409 | 1.468 | 0.883 | 1.406 | 1.165 | 1.734 | 1.468 | 1.886 | 1.823 | 1.940 | **1.499** |
| **BRxVP86** | 1.470 | 1.470 | 2.048 | 2.095 | 1.214 | 1.625 | 1.414 | 1.398 | 1.656 | 1.450 | 1.379 | 1.656 | 0.926 | 1.619 | 0.973 | 1.737 | 1.692 | 2.048 | 2.100 | 2.134 | **1.605** |
| **BRxVWN** | 1.198 | 1.186 | 1.549 | 1.544 | 0.836 | 1.303 | 1.217 | 1.140 | 1.304 | 1.251 | 1.139 | 1.305 | 0.779 | 1.120 | 1.033 | 1.434 | 1.167 | 1.616 | 1.462 | 1.633 | **1.261** |
| **BRxVWN5** | 1.278 | 1.243 | 1.616 | 1.619 | 0.887 | 1.371 | 1.362 | 1.242 | 1.353 | 1.357 | 1.240 | 1.368 | 0.804 | 1.214 | 1.113 | 1.609 | 1.262 | 1.710 | 1.624 | 1.700 | **1.348** |

| | | | | | | | | | | | | | | | | | | | | | |
|---|---|---|---|---|---|---|---|---|---|---|---|---|---|---|---|---|---|---|---|---|---|
| **G96B95** | 2.807 | 2.845 | 3.724 | 3.666 | 2.943 | 3.587 | 3.567 | 3.850 | 3.514 | 3.279 | 3.543 | 3.589 | 2.391 | 4.074 | 2.856 | 3.707 | 3.135 | 3.783 | 3.731 | 3.889 | **3.424** |
| **G96BRC** | 2.822 | 2.825 | 3.376 | 3.383 | 2.587 | 3.230 | 3.340 | 3.334 | 3.332 | 3.334 | 3.329 | 3.282 | 2.176 | 3.658 | 2.687 | 3.416 | 3.035 | 3.635 | 3.267 | 3.588 | **3.182** |
| **G96KCIS** | 2.831 | 2.838 | 3.369 | 3.369 | 2.797 | 3.349 | 3.244 | 3.241 | 3.349 | 3.244 | 3.240 | 3.341 | 2.165 | 3.684 | 2.802 | 3.382 | 3.022 | 3.511 | 3.353 | 3.509 | **3.182** |
| **G96LYP** | 2.812 | 2.812 | 3.437 | 3.443 | 2.660 | 3.376 | 3.376 | 3.319 | 3.376 | 3.369 | 3.318 | 3.369 | 2.158 | 3.988 | 2.702 | 3.428 | 3.040 | 3.527 | 3.426 | 3.487 | **3.221** |
| **G96P86** | 2.764 | 2.766 | 3.348 | 3.357 | 2.681 | 3.046 | 3.048 | 3.045 | 3.060 | 3.054 | 3.022 | 3.053 | 2.176 | 3.606 | 2.639 | 3.215 | 3.017 | 3.394 | 3.251 | 3.397 | **3.047** |
| **G96PBE** | 2.766 | 2.769 | 3.369 | 3.362 | 2.579 | 3.117 | 3.115 | 3.118 | 3.116 | 3.119 | 3.120 | 3.114 | 2.148 | 3.456 | 2.688 | 3.372 | 3.020 | 3.394 | 3.249 | 3.450 | **3.072** |
| **G96PKZB** | 2.770 | 2.770 | 3.367 | 3.363 | 2.575 | 3.110 | 3.112 | 3.119 | 3.118 | 3.112 | 3.116 | 3.112 | 2.147 | 3.517 | 2.684 | 3.372 | 3.026 | 3.390 | 3.289 | 3.388 | **3.073** |
| **G96PL** | 2.766 | 2.765 | 3.362 | 3.359 | 2.628 | 3.111 | 3.101 | 3.083 | 3.112 | 3.102 | 3.086 | 3.108 | 2.147 | 3.568 | 2.689 | 3.381 | 2.998 | 3.421 | 3.257 | 3.386 | **3.071** |
| **G96PW91** | 2.768 | 2.766 | 3.364 | 3.366 | 2.537 | 3.115 | 3.115 | 3.112 | 3.114 | 3.110 | 3.113 | 3.109 | 2.151 | 3.464 | 2.682 | 3.369 | 3.045 | 3.385 | 3.247 | 3.401 | **3.067** |
| **G96RevTPSS** | 2.771 | 2.770 | 3.372 | 3.364 | 2.624 | 3.118 | 3.107 | 3.123 | 3.118 | 3.111 | 3.123 | 3.118 | 2.149 | 3.529 | 2.687 | 3.388 | 3.019 | 3.382 | 3.246 | 3.401 | **3.076** |
| **G96TPSS** | 2.767 | 2.770 | 3.371 | 3.368 | 2.580 | 3.105 | 3.113 | 3.120 | 3.115 | 3.111 | 3.120 | 3.113 | 2.151 | 3.473 | 2.685 | 3.369 | 3.022 | 3.389 | 3.249 | 3.390 | **3.069** |
| **G96VP86** | 2.777 | 2.778 | 3.353 | 3.355 | 2.690 | 3.064 | 3.056 | 3.039 | 3.059 | 3.052 | 3.045 | 3.053 | 2.178 | 3.552 | 2.636 | 3.218 | 3.019 | 3.383 | 3.243 | 3.450 | **3.050** |
| **G96VWN** | 2.794 | 2.791 | 3.255 | 3.259 | 2.597 | 3.069 | 3.077 | 3.076 | 3.070 | 3.072 | 3.091 | 3.071 | 2.149 | 3.405 | 2.679 | 3.222 | 2.981 | 3.308 | 3.248 | 3.384 | **3.030** |
| **G96VWN5** | 2.765 | 2.764 | 3.360 | 3.352 | 2.624 | 3.113 | 3.092 | 3.082 | 3.083 | 3.092 | 3.084 | 3.111 | 2.144 | 3.549 | 2.689 | 3.379 | 2.992 | 3.409 | 3.260 | 3.388 | **3.067** |
| **LGB95** | -0.082 | -0.090 | 0.031 | 0.031 | -0.166 | 0.019 | 0.000 | -0.026 | 0.019 | -0.001 | -0.026 | 0.022 | -0.266 | 0.037 | -0.113 | -0.009 | -0.051 | 0.028 | 0.030 | 0.033 | **-0.029** |
| **LGBRC** | -0.007 | -0.015 | 0.118 | 0.118 | -0.098 | 0.142 | 0.096 | 0.073 | 0.140 | 0.083 | 0.064 | 0.156 | -0.253 | 0.122 | -0.050 | 0.108 | 0.047 | 0.123 | 0.115 | 0.125 | **0.060** |
| **LGKCIS** | -0.053 | -0.060 | 0.049 | 0.050 | -0.132 | 0.071 | 0.028 | 0.024 | 0.071 | 0.025 | 0.023 | 0.088 | -0.260 | 0.066 | -0.087 | 0.052 | -0.003 | 0.050 | 0.043 | 0.049 | **0.005** |
| **LGLYP** | -0.320 | -0.322 | -0.282 | -0.282 | -0.371 | -0.273 | -0.289 | -0.290 | -0.274 | -0.291 | -0.292 | -0.258 | -0.459 | -0.271 | -0.339 | -0.279 | -0.298 | -0.284 | -0.283 | -0.283 | **-0.302** |
| **LGP86** | -0.220 | -0.225 | -0.130 | -0.130 | -0.277 | -0.094 | -0.171 | -0.187 | -0.111 | -0.174 | -0.189 | -0.077 | -0.330 | -0.088 | -0.250 | -0.160 | -0.172 | -0.152 | -0.144 | -0.141 | **-0.171** |
| **LGPBE** | 0.035 | 0.016 | 0.181 | 0.181 | -0.067 | 0.209 | 0.140 | 0.132 | 0.207 | 0.138 | 0.133 | 0.227 | -0.191 | 0.227 | -0.016 | 0.172 | 0.085 | 0.177 | 0.176 | 0.177 | **0.117** |
| **LGPKZB** | 0.045 | 0.024 | 0.189 | 0.189 | -0.056 | 0.217 | 0.148 | 0.140 | 0.216 | 0.147 | 0.142 | 0.234 | -0.180 | 0.233 | -0.004 | 0.187 | 0.098 | 0.200 | 0.179 | 0.187 | **0.127** |
| **LGPL** | 0.115 | 0.107 | 0.219 | 0.219 | -0.008 | 0.256 | 0.209 | 0.185 | 0.255 | 0.208 | 0.185 | 0.264 | -0.100 | 0.268 | 0.050 | 0.254 | 0.141 | 0.231 | 0.214 | 0.216 | **0.174** |
| **LGPW91** | 0.009 | -0.003 | 0.146 | 0.146 | -0.084 | 0.169 | 0.108 | 0.095 | 0.168 | 0.107 | 0.095 | 0.192 | -0.207 | 0.180 | -0.040 | 0.134 | 0.065 | 0.146 | 0.139 | 0.140 | **0.085** |
| **LGRevTPSS** | -0.012 | -0.024 | 0.120 | 0.120 | -0.104 | 0.145 | 0.072 | 0.056 | 0.141 | 0.075 | 0.054 | 0.161 | -0.220 | 0.150 | -0.059 | 0.093 | 0.040 | 0.114 | 0.115 | 0.118 | **0.058** |
| **LGTPSS** | 0.042 | 0.022 | 0.187 | 0.187 | -0.063 | 0.219 | 0.146 | 0.138 | 0.214 | 0.146 | 0.143 | 0.236 | -0.186 | 0.232 | -0.007 | 0.185 | 0.096 | 0.187 | 0.175 | 0.184 | **0.124** |
| **LGVP86** | -0.217 | -0.223 | -0.129 | -0.128 | -0.276 | -0.100 | -0.161 | -0.176 | -0.109 | -0.169 | -0.186 | -0.082 | -0.329 | -0.084 | -0.238 | -0.157 | -0.170 | -0.145 | -0.137 | -0.138 | **-0.168** |
| **LGVWN** | 0.082 | 0.068 | 0.171 | 0.171 | -0.036 | 0.190 | 0.144 | 0.128 | 0.189 | 0.140 | 0.131 | 0.198 | -0.135 | 0.208 | 0.023 | 0.172 | 0.094 | 0.174 | 0.164 | 0.165 | **0.122** |
| **LGVWN5** | 0.117 | 0.109 | 0.221 | 0.221 | -0.004 | 0.257 | 0.210 | 0.188 | 0.256 | 0.210 | 0.186 | 0.267 | -0.095 | 0.271 | 0.053 | 0.259 | 0.143 | 0.233 | 0.232 | 0.218 | **0.178** |
| **mPWB95** | 0.098 | 0.098 | 0.159 | 0.159 | -0.041 | 0.145 | 0.143 | 0.143 | 0.145 | 0.143 | 0.143 | 0.146 | -0.125 | 0.160 | 0.083 | 0.155 | 0.153 | 0.160 | 0.158 | 0.160 | **0.119** |
| **mPWBRC** | 0.272 | 0.266 | 0.458 | 0.458 | 0.138 | 0.452 | 0.414 | 0.397 | 0.451 | 0.413 | 0.396 | 0.452 | -0.047 | 0.416 | 0.209 | 0.485 | 0.341 | 0.498 | 0.457 | 0.495 | **0.371** |
| **mPWKCIS** | 0.247 | 0.239 | 0.410 | 0.410 | 0.109 | 0.398 | 0.370 | 0.350 | 0.397 | 0.370 | 0.347 | 0.402 | -0.034 | 0.383 | 0.181 | 0.436 | 0.300 | 0.438 | 0.404 | 0.433 | **0.329** |
| **mPWLYP** | -0.106 | -0.108 | -0.012 | -0.012 | -0.189 | -0.009 | -0.035 | -0.039 | -0.010 | -0.037 | -0.041 | 0.005 | -0.329 | 0.000 | -0.139 | 0.003 | -0.060 | -0.008 | -0.013 | -0.007 | **-0.057** |
| **mPWP86** | 0.178 | 0.166 | 0.424 | 0.424 | 0.044 | 0.392 | 0.300 | 0.272 | 0.392 | 0.298 | 0.271 | 0.421 | -0.110 | 0.365 | 0.111 | 0.397 | 0.269 | 0.441 | 0.410 | 0.440 | **0.295** |
| **mPWPBE** | 0.360 | 0.354 | 0.517 | 0.517 | 0.212 | 0.538 | 0.495 | 0.470 | 0.537 | 0.495 | 0.467 | 0.538 | 0.043 | 0.476 | 0.289 | 0.570 | 0.401 | 0.551 | 0.516 | 0.540 | **0.444** |
| **mPWPKZB** | 0.363 | 0.359 | 0.520 | 0.520 | 0.216 | 0.537 | 0.498 | 0.470 | 0.536 | 0.501 | 0.471 | 0.537 | 0.046 | 0.479 | 0.292 | 0.569 | 0.406 | 0.554 | 0.521 | 0.543 | **0.447** |
| **mPWPL** | 0.354 | 0.344 | 0.484 | 0.484 | 0.202 | 0.502 | 0.464 | 0.437 | 0.502 | 0.462 | 0.434 | 0.504 | 0.049 | 0.443 | 0.287 | 0.543 | 0.380 | 0.518 | 0.486 | 0.508 | **0.419** |
| **mPWPW91** | 0.351 | 0.343 | 0.511 | 0.511 | 0.205 | 0.521 | 0.485 | 0.455 | 0.523 | 0.483 | 0.454 | 0.527 | 0.035 | 0.470 | 0.280 | 0.557 | 0.392 | 0.545 | 0.509 | 0.532 | **0.434** |
| **mPWRevTPSS** | 0.328 | 0.315 | 0.491 | 0.491 | 0.187 | 0.497 | 0.452 | 0.420 | 0.496 | 0.451 | 0.420 | 0.501 | 0.021 | 0.452 | 0.261 | 0.519 | 0.375 | 0.519 | 0.489 | 0.510 | **0.410** |
| **mPWTPSS** | 0.362 | 0.357 | 0.522 | 0.522 | 0.215 | 0.539 | 0.500 | 0.473 | 0.538 | 0.500 | 0.473 | 0.540 | 0.045 | 0.481 | 0.292 | 0.574 | 0.406 | 0.554 | 0.520 | 0.543 | **0.448** |
| **mPWVP86** | 0.180 | 0.169 | 0.427 | 0.427 | 0.047 | 0.398 | 0.303 | 0.275 | 0.393 | 0.301 | 0.274 | 0.426 | -0.104 | 0.369 | 0.117 | 0.399 | 0.272 | 0.449 | 0.415 | 0.448 | **0.299** |
| **mPWVWN** | 0.323 | 0.316 | 0.440 | 0.440 | 0.181 | 0.431 | 0.406 | 0.392 | 0.428 | 0.407 | 0.391 | 0.432 | 0.027 | 0.409 | 0.262 | 0.476 | 0.355 | 0.474 | 0.439 | 0.454 | **0.374** |
| **mPWVWN5** | 0.356 | 0.345 | 0.486 | 0.486 | 0.203 | 0.503 | 0.465 | 0.439 | 0.502 | 0.463 | 0.437 | 0.504 | 0.050 | 0.444 | 0.288 | 0.545 | 0.381 | 0.520 | 0.489 | 0.509 | **0.421** |
| **OB95** | 0.304 | 0.304 | 0.402 | 0.402 | 0.228 | 0.411 | 0.410 | 0.367 | 0.411 | 0.410 | 0.367 | 0.448 | 0.102 | 0.373 | 0.295 | 0.432 | 0.362 | 0.396 | 0.390 | 0.334 | **0.357** |
| **OBRC** | 0.478 | 0.474 | 0.711 | 0.711 | 0.329 | 0.709 | 0.695 | 0.672 | 0.708 | 0.695 | 0.673 | 0.709 | 0.205 | 0.633 | 0.407 | 0.763 | 0.566 | 0.781 | 0.706 | 0.752 | **0.619** |
| **OKCIS** | 0.471 | 0.466 | 0.686 | 0.686 | 0.321 | 0.688 | 0.667 | 0.647 | 0.687 | 0.666 | 0.647 | 0.692 | 0.208 | 0.609 | 0.408 | 0.725 | 0.547 | 0.717 | 0.682 | 0.724 | **0.597** |
| **OLYP** | 0.257 | 0.253 | 0.444 | 0.444 | 0.122 | 0.445 | 0.424 | 0.409 | 0.445 | 0.423 | 0.409 | 0.446 | -0.027 | 0.383 | 0.207 | 0.494 | 0.333 | 0.475 | 0.454 | 0.460 | **0.365** |
| **OP86** | 0.559 | 0.553 | 0.877 | 0.875 | 0.400 | 0.775 | 0.768 | 0.713 | 0.775 | 0.768 | 0.712 | 0.803 | 0.295 | 0.760 | 0.529 | 0.942 | 0.650 | 0.943 | 0.803 | 0.904 | **0.720** |
| **OPBE** | 0.535 | 0.531 | 0.761 | 0.761 | 0.379 | 0.729 | 0.707 | 0.684 | 0.728 | 0.706 | 0.684 | 0.727 | 0.275 | 0.668 | 0.481 | 0.833 | 0.599 | 0.822 | 0.725 | 0.822 | **0.658** |
| **OPKZB** | 0.538 | 0.534 | 0.764 | 0.764 | 0.382 | 0.730 | 0.709 | 0.687 | 0.730 | 0.709 | 0.687 | 0.732 | 0.276 | 0.672 | 0.484 | 0.833 | 0.603 | 0.820 | 0.728 | 0.831 | **0.661** |
| **OPL** | 0.523 | 0.515 | 0.708 | 0.708 | 0.361 | 0.713 | 0.692 | 0.672 | 0.712 | 0.692 | 0.672 | 0.714 | 0.246 | 0.635 | 0.468 | 0.755 | 0.584 | 0.771 | 0.705 | 0.731 | **0.629** |
| **OPW91** | 0.533 | 0.529 | 0.725 | 0.725 | 0.377 | 0.726 | 0.702 | 0.682 | 0.726 | 0.702 | 0.681 | 0.726 | 0.272 | 0.661 | 0.479 | 0.829 | 0.599 | 0.820 | 0.721 | 0.806 | **0.651** |
| **ORevTPSS** | 0.527 | 0.522 | 0.718 | 0.718 | 0.368 | 0.720 | 0.696 | 0.675 | 0.719 | 0.696 | 0.675 | 0.718 | 0.260 | 0.654 | 0.471 | 0.815 | 0.591 | 0.804 | 0.713 | 0.798 | **0.643** |
| **OTPSS** | 0.538 | 0.534 | 0.765 | 0.765 | 0.382 | 0.732 | 0.710 | 0.688 | 0.732 | 0.710 | 0.688 | 0.734 | 0.275 | 0.673 | 0.483 | 0.835 | 0.603 | 0.825 | 0.731 | 0.826 | **0.661** |
| **OVP86** | 0.560 | 0.554 | 0.878 | 0.877 | 0.418 | 0.776 | 0.768 | 0.713 | 0.776 | 0.768 | 0.713 | 0.804 | 0.297 | 0.760 | 0.530 | 0.926 | 0.649 | 0.944 | 0.804 | 0.921 | **0.722** |
| **OVWN** | 0.499 | 0.493 | 0.667 | 0.667 | 0.346 | 0.661 | 0.644 | 0.622 | 0.661 | 0.641 | 0.622 | 0.661 | 0.234 | 0.599 | 0.429 | 0.707 | 0.556 | 0.702 | 0.666 | 0.693 | **0.589** |
| **OVWN5** | 0.524 | 0.516 | 0.708 | 0.708 | 0.362 | 0.713 | 0.692 | 0.673 | 0.713 | 0.693 | 0.673 | 0.714 | 0.246 | 0.636 | 0.469 | 0.755 | 0.585 | 0.772 | 0.706 | 0.731 | **0.629** |
| **PBEB95** | -0.110 | -0.111 | -0.050 | -0.050 | -0.213 | -0.033 | -0.058 | -0.059 | -0.033 | -0.058 | -0.059 | 0.009 | -0.300 | -0.021 | -0.173 | -0.076 | -0.106 | -0.038 | -0.050 | -0.034 | **-0.081** |

| | | | | | | | | | | | | | | | | | | | | | |
|---|---|---|---|---|---|---|---|---|---|---|---|---|---|---|---|---|---|---|---|---|---|
| **PBEBRC** | -0.048 | -0.052 | 0.036 | 0.036 | -0.135 | 0.041 | 0.016 | 0.008 | 0.039 | 0.014 | 0.007 | 0.051 | -0.266 | 0.031 | -0.087 | 0.042 | -0.010 | 0.044 | 0.035 | 0.040 | **-0.008** |
| **PBEKCIS** | -0.087 | -0.090 | -0.005 | -0.005 | -0.164 | 0.000 | -0.017 | -0.026 | 0.000 | -0.017 | -0.028 | 0.011 | -0.274 | 0.014 | -0.121 | -0.002 | -0.048 | -0.004 | -0.006 | -0.004 | **-0.044** |
| **PBELYP** | -0.307 | -0.308 | -0.265 | -0.265 | -0.359 | -0.259 | -0.274 | -0.275 | -0.260 | -0.276 | -0.276 | -0.246 | -0.448 | -0.252 | -0.325 | -0.260 | -0.284 | -0.266 | -0.265 | -0.264 | **-0.287** |
| **PBEP86** | -0.220 | -0.222 | -0.167 | -0.167 | -0.274 | -0.153 | -0.182 | -0.187 | -0.155 | -0.184 | -0.189 | -0.137 | -0.366 | -0.161 | -0.246 | -0.174 | -0.194 | -0.174 | -0.172 | -0.170 | **-0.195** |
| **PBEPBE** | -0.034 | -0.039 | 0.054 | 0.054 | -0.122 | 0.055 | 0.024 | 0.012 | 0.055 | 0.023 | 0.011 | 0.069 | -0.229 | 0.064 | -0.076 | 0.053 | -0.005 | 0.057 | 0.050 | 0.052 | **0.006** |
| **PBEPKZB** | -0.026 | -0.031 | 0.060 | 0.060 | -0.115 | 0.062 | 0.031 | 0.017 | 0.060 | 0.030 | 0.016 | 0.079 | -0.222 | 0.072 | -0.069 | 0.062 | 0.003 | 0.063 | 0.056 | 0.059 | **0.013** |
| **PBEPL** | 0.035 | 0.031 | 0.106 | 0.106 | -0.062 | 0.110 | 0.087 | 0.078 | 0.109 | 0.087 | 0.078 | 0.117 | -0.171 | 0.125 | -0.009 | 0.122 | 0.057 | 0.114 | 0.105 | 0.105 | **0.067** |
| **PBEPW91** | -0.049 | -0.054 | 0.039 | 0.039 | -0.133 | 0.039 | 0.011 | -0.004 | 0.038 | 0.010 | -0.006 | 0.052 | -0.241 | 0.045 | -0.088 | 0.038 | -0.019 | 0.038 | 0.031 | 0.038 | **-0.009** |
| **PBERevTPSS** | -0.060 | -0.065 | 0.021 | 0.021 | -0.141 | 0.027 | -0.003 | -0.014 | 0.026 | -0.004 | -0.016 | 0.041 | -0.248 | 0.031 | -0.097 | 0.025 | -0.030 | 0.023 | 0.016 | 0.020 | **-0.021** |
| **PBETPSS** | -0.027 | -0.033 | 0.059 | 0.059 | -0.117 | 0.062 | 0.031 | 0.018 | 0.060 | 0.030 | 0.016 | 0.079 | -0.224 | 0.071 | -0.070 | 0.062 | 0.002 | 0.062 | 0.056 | 0.058 | **0.013** |
| **PBEVP86** | -0.218 | -0.221 | -0.165 | -0.165 | -0.273 | -0.152 | -0.179 | -0.186 | -0.153 | -0.182 | -0.187 | -0.135 | -0.365 | -0.159 | -0.244 | -0.173 | -0.193 | -0.172 | -0.170 | -0.169 | **-0.193** |
| **PBEVWN** | 0.005 | 0.002 | 0.069 | 0.069 | -0.083 | 0.072 | 0.052 | 0.043 | 0.071 | 0.050 | 0.042 | 0.080 | -0.188 | 0.088 | -0.034 | 0.084 | 0.027 | 0.073 | 0.067 | 0.066 | **0.033** |
| **PBEVWN5** | 0.036 | 0.032 | 0.107 | 0.107 | -0.060 | 0.111 | 0.088 | 0.080 | 0.110 | 0.088 | 0.079 | 0.118 | -0.169 | 0.127 | -0.008 | 0.124 | 0.059 | 0.115 | 0.107 | 0.106 | **0.068** |
| **PBEhB95** | -0.110 | -0.111 | -0.051 | -0.050 | -0.213 | -0.041 | -0.093 | -0.075 | -0.041 | -0.093 | -0.076 | -0.013 | -0.316 | -0.021 | -0.188 | -0.076 | -0.105 | -0.053 | -0.062 | -0.048 | **-0.092** |
| **PBEhBRC** | -0.049 | -0.052 | 0.029 | 0.029 | -0.136 | 0.034 | 0.012 | 0.005 | 0.032 | 0.011 | 0.004 | 0.044 | -0.264 | 0.028 | -0.088 | 0.041 | -0.013 | 0.036 | 0.028 | 0.033 | **-0.012** |
| **PBEhKCIS** | -0.084 | -0.089 | -0.008 | -0.008 | -0.164 | -0.004 | -0.022 | -0.030 | -0.005 | -0.023 | -0.032 | 0.007 | -0.271 | 0.013 | -0.120 | -0.003 | -0.051 | -0.007 | -0.009 | -0.006 | **-0.046** |
| **PBEhLYP** | -0.302 | -0.303 | -0.261 | -0.261 | -0.355 | -0.253 | -0.268 | -0.268 | -0.255 | -0.269 | -0.270 | -0.240 | -0.444 | -0.246 | -0.319 | -0.253 | -0.279 | -0.262 | -0.260 | -0.260 | **-0.281** |
| **PBEhP86** | -0.218 | -0.221 | -0.165 | -0.165 | -0.272 | -0.154 | -0.180 | -0.184 | -0.155 | -0.182 | -0.187 | -0.138 | -0.363 | -0.159 | -0.242 | -0.172 | -0.193 | -0.173 | -0.171 | -0.170 | **-0.193** |
| **PBEhPBE** | -0.039 | -0.044 | 0.045 | 0.045 | -0.125 | 0.045 | 0.016 | 0.007 | 0.043 | 0.014 | 0.006 | 0.059 | -0.231 | 0.062 | -0.079 | 0.046 | -0.009 | 0.043 | 0.043 | 0.045 | **0.000** |
| **PBEhPKZB** | -0.031 | -0.036 | 0.052 | 0.052 | -0.119 | 0.051 | 0.024 | 0.013 | 0.050 | 0.022 | 0.012 | 0.065 | -0.224 | 0.069 | -0.071 | 0.054 | -0.002 | 0.051 | 0.049 | 0.051 | **0.007** |
| **PBEhPL** | 0.032 | 0.028 | 0.097 | 0.097 | -0.061 | 0.100 | 0.082 | 0.073 | 0.100 | 0.082 | 0.072 | 0.109 | -0.175 | 0.118 | -0.009 | 0.117 | 0.054 | 0.103 | 0.096 | 0.096 | **0.061** |
| **PBEhPW91** | -0.053 | -0.058 | 0.026 | 0.026 | -0.136 | 0.030 | -0.001 | -0.009 | 0.028 | -0.003 | -0.011 | 0.045 | -0.242 | 0.043 | -0.091 | 0.031 | -0.023 | 0.026 | 0.022 | 0.025 | **-0.016** |
| **PBEhRevTPSS** | -0.063 | -0.067 | 0.012 | 0.012 | -0.144 | 0.016 | -0.011 | -0.019 | 0.015 | -0.015 | -0.021 | 0.030 | -0.247 | 0.031 | -0.099 | 0.017 | -0.033 | 0.012 | 0.008 | 0.011 | **-0.028** |
| **PBEhTPSS** | -0.032 | -0.037 | 0.051 | 0.052 | -0.120 | 0.052 | 0.023 | 0.013 | 0.050 | 0.021 | 0.012 | 0.066 | -0.226 | 0.069 | -0.072 | 0.054 | -0.003 | 0.050 | 0.049 | 0.051 | **0.006** |
| **PBEhVP86** | -0.216 | -0.219 | -0.164 | -0.164 | -0.270 | -0.152 | -0.178 | -0.183 | -0.154 | -0.180 | -0.185 | -0.136 | -0.361 | -0.158 | -0.239 | -0.170 | -0.192 | -0.171 | -0.169 | -0.168 | **-0.192** |
| **PBEhVWN** | 0.002 | -0.001 | 0.063 | 0.063 | -0.085 | 0.068 | 0.045 | 0.039 | 0.067 | 0.044 | 0.038 | 0.075 | -0.190 | 0.083 | -0.035 | 0.082 | 0.024 | 0.065 | 0.061 | 0.060 | **0.028** |
| **PBEhVWN5** | 0.033 | 0.030 | 0.099 | 0.099 | -0.060 | 0.102 | 0.083 | 0.074 | 0.101 | 0.083 | 0.073 | 0.109 | -0.173 | 0.120 | -0.008 | 0.117 | 0.055 | 0.105 | 0.098 | 0.097 | **0.062** |
| **PKZBB95** | 0.040 | 0.039 | 0.132 | 0.132 | -0.078 | 0.136 | 0.134 | 0.134 | 0.136 | 0.134 | 0.134 | 0.106 | -0.141 | 0.122 | 0.049 | 0.122 | 0.101 | 0.132 | 0.132 | 0.133 | **0.091** |
| **PKZBBRC** | 0.093 | 0.091 | 0.215 | 0.215 | -0.012 | 0.201 | 0.186 | 0.181 | 0.200 | 0.185 | 0.180 | 0.205 | -0.136 | 0.182 | 0.049 | 0.222 | 0.153 | 0.224 | 0.219 | 0.224 | **0.154** |
| **PKZBKCIS** | 0.079 | 0.076 | 0.182 | 0.182 | -0.021 | 0.169 | 0.153 | 0.149 | 0.168 | 0.152 | 0.149 | 0.176 | -0.136 | 0.164 | 0.039 | 0.190 | 0.126 | 0.188 | 0.184 | 0.187 | **0.128** |
| **PKZBLYP** | -0.086 | -0.087 | -0.012 | -0.012 | -0.174 | -0.025 | -0.033 | -0.034 | -0.026 | -0.034 | -0.035 | -0.018 | -0.295 | -0.029 | -0.118 | -0.003 | -0.044 | -0.009 | -0.010 | -0.011 | **-0.055** |
| **PKZBP86** | 0.023 | 0.020 | 0.106 | 0.106 | -0.069 | 0.100 | 0.083 | 0.076 | 0.100 | 0.082 | 0.075 | 0.106 | -0.189 | 0.097 | -0.016 | 0.111 | 0.059 | 0.112 | 0.107 | 0.113 | **0.060** |
| **PKZBPBE** | 0.116 | 0.114 | 0.225 | 0.225 | 0.010 | 0.210 | 0.196 | 0.187 | 0.210 | 0.196 | 0.186 | 0.215 | -0.096 | 0.208 | 0.074 | 0.238 | 0.165 | 0.232 | 0.227 | 0.232 | **0.168** |
| **PKZBPKZB** | 0.121 | 0.118 | 0.229 | 0.229 | 0.014 | 0.214 | 0.200 | 0.191 | 0.213 | 0.199 | 0.190 | 0.219 | -0.094 | 0.212 | 0.078 | 0.242 | 0.170 | 0.235 | 0.231 | 0.235 | **0.172** |
| **PKZBPL** | 0.142 | 0.140 | 0.249 | 0.249 | 0.033 | 0.235 | 0.221 | 0.214 | 0.234 | 0.220 | 0.213 | 0.239 | -0.079 | 0.239 | 0.098 | 0.263 | 0.191 | 0.254 | 0.252 | 0.252 | **0.193** |
| **PKZBPW91** | 0.110 | 0.107 | 0.215 | 0.215 | 0.004 | 0.202 | 0.188 | 0.178 | 0.202 | 0.187 | 0.177 | 0.208 | -0.103 | 0.200 | 0.065 | 0.229 | 0.158 | 0.222 | 0.217 | 0.222 | **0.160** |
| **PKZBRevTPSS** | 0.102 | 0.100 | 0.204 | 0.205 | -0.003 | 0.193 | 0.178 | 0.169 | 0.192 | 0.177 | 0.168 | 0.199 | -0.108 | 0.190 | 0.059 | 0.216 | 0.147 | 0.211 | 0.207 | 0.210 | **0.151** |
| **PKZBTPSS** | 0.120 | 0.118 | 0.230 | 0.230 | 0.014 | 0.215 | 0.201 | 0.193 | 0.215 | 0.200 | 0.192 | 0.221 | -0.094 | 0.213 | 0.078 | 0.242 | 0.170 | 0.237 | 0.232 | 0.236 | **0.173** |
| **PKZBVP86** | 0.023 | 0.021 | 0.107 | 0.107 | -0.069 | 0.101 | 0.084 | 0.077 | 0.100 | 0.083 | 0.076 | 0.108 | -0.188 | 0.098 | -0.016 | 0.112 | 0.059 | 0.113 | 0.108 | 0.114 | **0.061** |
| **PKZBVWN** | 0.119 | 0.117 | 0.213 | 0.213 | 0.012 | 0.201 | 0.187 | 0.180 | 0.200 | 0.186 | 0.179 | 0.205 | -0.089 | 0.202 | 0.078 | 0.227 | 0.161 | 0.217 | 0.216 | 0.215 | **0.162** |
| **PKZBVWN5** | 0.143 | 0.140 | 0.250 | 0.250 | 0.033 | 0.235 | 0.221 | 0.214 | 0.234 | 0.221 | 0.213 | 0.239 | -0.078 | 0.240 | 0.098 | 0.263 | 0.191 | 0.255 | 0.253 | 0.253 | **0.193** |
| **PW91B95** | -0.159 | -0.160 | -0.104 | -0.104 | -0.241 | -0.114 | -0.124 | -0.119 | -0.115 | -0.124 | -0.119 | -0.070 | -0.374 | -0.110 | -0.195 | -0.101 | -0.153 | -0.097 | -0.105 | -0.077 | **-0.138** |
| **PW91BRC** | -0.083 | -0.085 | -0.013 | -0.013 | -0.172 | -0.012 | -0.029 | -0.035 | -0.013 | -0.031 | -0.036 | -0.002 | -0.296 | -0.026 | -0.123 | -0.004 | -0.051 | -0.004 | -0.011 | -0.009 | **-0.052** |
| **PW91KCIS** | -0.120 | -0.122 | -0.045 | -0.045 | -0.197 | -0.043 | -0.062 | -0.067 | -0.044 | -0.063 | -0.068 | -0.032 | -0.306 | -0.045 | -0.153 | -0.037 | -0.087 | -0.039 | -0.046 | -0.038 | **-0.083** |
| **PW91LYP** | -0.313 | -0.314 | -0.269 | -0.269 | -0.368 | -0.265 | -0.279 | -0.279 | -0.266 | -0.281 | -0.281 | -0.252 | -0.463 | -0.264 | -0.332 | -0.264 | -0.289 | -0.268 | -0.269 | -0.267 | **-0.292** |
| **PW91P86** | -0.236 | -0.237 | -0.189 | -0.189 | -0.293 | -0.181 | -0.202 | -0.205 | -0.183 | -0.204 | -0.207 | -0.166 | -0.391 | -0.199 | -0.264 | -0.192 | -0.213 | -0.189 | -0.191 | -0.187 | **-0.216** |
| **PW91PBE** | -0.081 | -0.083 | -0.014 | -0.014 | -0.164 | -0.014 | -0.034 | -0.040 | -0.015 | -0.036 | -0.042 | -0.004 | -0.273 | -0.022 | -0.119 | -0.006 | -0.054 | -0.007 | -0.015 | -0.011 | **-0.052** |
| **PW91PKZB** | -0.073 | -0.076 | -0.008 | -0.007 | -0.159 | -0.010 | -0.028 | -0.034 | -0.011 | -0.029 | -0.035 | 0.001 | -0.266 | -0.014 | -0.112 | 0.001 | -0.048 | -0.001 | -0.008 | -0.004 | **-0.046** |
| **PW91PL** | -0.016 | -0.018 | 0.043 | 0.043 | -0.107 | 0.042 | 0.028 | 0.025 | 0.041 | 0.027 | 0.024 | 0.050 | -0.216 | 0.051 | -0.054 | 0.061 | 0.008 | 0.051 | 0.044 | 0.044 | **0.009** |
| **PW91PW91** | -0.092 | -0.095 | -0.027 | -0.027 | -0.174 | -0.027 | -0.047 | -0.053 | -0.028 | -0.048 | -0.054 | -0.014 | -0.283 | -0.035 | -0.129 | -0.023 | -0.066 | -0.021 | -0.027 | -0.026 | **-0.065** |
| **PW91RevTPSS** | -0.099 | -0.102 | -0.035 | -0.035 | -0.179 | -0.035 | -0.055 | -0.060 | -0.035 | -0.056 | -0.061 | -0.023 | -0.286 | -0.042 | -0.135 | -0.030 | -0.072 | -0.032 | -0.035 | -0.032 | **-0.072** |
| **PW91TPSS** | -0.074 | -0.077 | -0.007 | -0.007 | -0.159 | -0.009 | -0.028 | -0.034 | -0.010 | -0.029 | -0.035 | 0.002 | -0.267 | -0.014 | -0.112 | 0.001 | -0.048 | -0.001 | -0.008 | -0.004 | **-0.046** |
| **PW91VP86** | -0.234 | -0.236 | -0.188 | -0.188 | -0.292 | -0.180 | -0.201 | -0.204 | -0.181 | -0.203 | -0.206 | -0.165 | -0.390 | -0.197 | -0.262 | -0.190 | -0.212 | -0.188 | -0.190 | -0.186 | **-0.215** |
| **PW91VWN** | -0.040 | -0.042 | 0.012 | 0.012 | -0.129 | 0.013 | -0.001 | -0.007 | 0.012 | -0.003 | -0.008 | 0.021 | -0.231 | 0.024 | -0.077 | 0.030 | -0.020 | 0.017 | 0.012 | 0.011 | **-0.020** |
| **PW91VWN5** | -0.015 | -0.017 | 0.044 | 0.045 | -0.106 | 0.043 | 0.029 | 0.025 | 0.042 | 0.028 | 0.024 | 0.051 | -0.216 | 0.052 | -0.053 | 0.062 | 0.009 | 0.051 | 0.045 | 0.044 | **0.009** |
| **RevTPSSB95** | 0.066 | 0.058 | 0.073 | 0.073 | -0.081 | 0.065 | 0.059 | 0.058 | 0.065 | 0.059 | 0.058 | 0.090 | -0.173 | 0.079 | -0.016 | 0.069 | 0.067 | 0.076 | 0.077 | 0.078 | **0.045** |
| **RevTPSSBRC** | 0.179 | 0.175 | 0.299 | 0.299 | 0.050 | 0.300 | 0.267 | 0.258 | 0.300 | 0.266 | 0.256 | 0.309 | -0.096 | 0.277 | 0.112 | 0.326 | 0.220 | 0.320 | 0.293 | 0.314 | **0.236** |

| | | | | | | | | | | | | | | | | | | | | | |
|---|---|---|---|---|---|---|---|---|---|---|---|---|---|---|---|---|---|---|---|---|---|
| **RevTPSSKCIS** | 0.149 | 0.143 | 0.243 | 0.243 | 0.028 | 0.243 | 0.213 | 0.198 | 0.241 | 0.212 | 0.198 | 0.251 | -0.087 | 0.245 | 0.092 | 0.265 | 0.181 | 0.251 | 0.240 | 0.246 | **0.190** |
| **RevTPSSLYP** | -0.200 | -0.201 | -0.147 | -0.147 | -0.267 | -0.148 | -0.167 | -0.169 | -0.150 | -0.169 | -0.171 | -0.135 | -0.371 | -0.143 | -0.229 | -0.144 | -0.173 | -0.147 | -0.149 | -0.146 | **-0.179** |
| **RevTPSSP86** | 0.039 | 0.033 | 0.145 | 0.144 | -0.081 | 0.135 | 0.102 | 0.088 | 0.136 | 0.100 | 0.087 | 0.149 | -0.175 | 0.140 | -0.029 | 0.126 | 0.084 | 0.141 | 0.138 | 0.143 | **0.082** |
| **RevTPSSPBE** | 0.243 | 0.224 | 0.364 | 0.364 | 0.099 | 0.332 | 0.289 | 0.278 | 0.330 | 0.288 | 0.278 | 0.353 | -0.003 | 0.350 | 0.191 | 0.379 | 0.262 | 0.398 | 0.352 | 0.364 | **0.287** |
| **RevTPSSPKZB** | 0.248 | 0.229 | 0.365 | 0.365 | 0.103 | 0.338 | 0.290 | 0.281 | 0.337 | 0.291 | 0.281 | 0.373 | 0.005 | 0.354 | 0.194 | 0.399 | 0.265 | 0.401 | 0.354 | 0.369 | **0.292** |
| **RevTPSSPL** | 0.262 | 0.257 | 0.360 | 0.360 | 0.125 | 0.362 | 0.325 | 0.312 | 0.361 | 0.324 | 0.310 | 0.366 | 0.005 | 0.351 | 0.193 | 0.406 | 0.277 | 0.379 | 0.353 | 0.360 | **0.302** |
| **RevTPSSPW91** | 0.231 | 0.216 | 0.350 | 0.350 | 0.090 | 0.322 | 0.281 | 0.272 | 0.321 | 0.281 | 0.271 | 0.326 | -0.019 | 0.345 | 0.182 | 0.366 | 0.255 | 0.384 | 0.346 | 0.349 | **0.276** |
| **RevTPSSRevTPSS** | 0.205 | 0.201 | 0.337 | 0.337 | 0.077 | 0.305 | 0.269 | 0.260 | 0.306 | 0.269 | 0.258 | 0.311 | -0.033 | 0.330 | 0.160 | 0.348 | 0.242 | 0.369 | 0.331 | 0.340 | **0.261** |
| **RevTPSSTPSS** | 0.247 | 0.227 | 0.366 | 0.366 | 0.101 | 0.339 | 0.291 | 0.282 | 0.338 | 0.292 | 0.281 | 0.374 | 0.000 | 0.354 | 0.194 | 0.400 | 0.264 | 0.401 | 0.354 | 0.369 | **0.292** |
| **RevTPSSVP86** | 0.041 | 0.035 | 0.147 | 0.148 | -0.079 | 0.138 | 0.104 | 0.091 | 0.137 | 0.102 | 0.089 | 0.152 | -0.173 | 0.144 | -0.027 | 0.128 | 0.086 | 0.144 | 0.142 | 0.145 | **0.085** |
| **RevTPSSVWN** | 0.226 | 0.221 | 0.295 | 0.295 | 0.096 | 0.312 | 0.271 | 0.269 | 0.310 | 0.269 | 0.268 | 0.317 | -0.014 | 0.305 | 0.163 | 0.346 | 0.237 | 0.324 | 0.293 | 0.293 | **0.255** |
| **RevTPSSVWN5** | 0.263 | 0.259 | 0.362 | 0.362 | 0.127 | 0.363 | 0.327 | 0.313 | 0.362 | 0.326 | 0.312 | 0.367 | 0.006 | 0.353 | 0.194 | 0.408 | 0.279 | 0.387 | 0.355 | 0.365 | **0.305** |
| **SB95** | -0.642 | -0.643 | -0.637 | -0.637 | -0.632 | -0.597 | -0.615 | -0.629 | -0.600 | -0.619 | -0.633 | -0.583 | -0.707 | -0.632 | -0.639 | -0.645 | -0.647 | -0.647 | -0.643 | -0.645 | **-0.634** |
| **SBRC** | -0.564 | -0.565 | -0.554 | -0.554 | -0.595 | -0.543 | -0.567 | -0.570 | -0.546 | -0.571 | -0.573 | -0.517 | -0.656 | -0.575 | -0.576 | -0.560 | -0.562 | -0.557 | -0.559 | -0.560 | **-0.566** |
| **SKCIS** | -0.601 | -0.604 | -0.594 | -0.594 | -0.621 | -0.577 | -0.601 | -0.605 | -0.580 | -0.605 | -0.608 | -0.551 | -0.681 | -0.610 | -0.611 | -0.601 | -0.601 | -0.597 | -0.598 | -0.598 | **-0.602** |
| **SLYP** | -0.699 | -0.701 | -0.695 | -0.695 | -0.714 | -0.677 | -0.695 | -0.697 | -0.679 | -0.698 | -0.700 | -0.654 | -0.757 | -0.694 | -0.704 | -0.696 | -0.699 | -0.695 | -0.696 | -0.696 | **-0.697** |
| **SP86** | -0.696 | -0.699 | -0.700 | -0.700 | -0.709 | -0.681 | -0.704 | -0.707 | -0.684 | -0.708 | -0.710 | -0.653 | -0.752 | -0.708 | -0.702 | -0.705 | -0.702 | -0.702 | -0.703 | -0.704 | **-0.701** |
| **SPBE** | -0.605 | -0.608 | -0.602 | -0.602 | -0.627 | -0.586 | -0.613 | -0.616 | -0.589 | -0.617 | -0.621 | -0.556 | -0.687 | -0.625 | -0.617 | -0.611 | -0.608 | -0.605 | -0.607 | -0.607 | **-0.610** |
| **SPKZB** | -0.591 | -0.594 | -0.586 | -0.586 | -0.613 | -0.572 | -0.598 | -0.601 | -0.576 | -0.602 | -0.605 | -0.543 | -0.675 | -0.610 | -0.603 | -0.596 | -0.592 | -0.589 | -0.591 | -0.591 | **-0.596** |
| **SPL** | -0.485 | -0.487 | -0.465 | -0.465 | -0.509 | -0.450 | -0.474 | -0.475 | -0.454 | -0.478 | -0.478 | -0.427 | -0.582 | -0.479 | -0.495 | -0.474 | -0.474 | -0.468 | -0.468 | -0.468 | **-0.478** |
| **SPW91** | -0.613 | -0.615 | -0.610 | -0.610 | -0.633 | -0.594 | -0.620 | -0.624 | -0.598 | -0.624 | -0.628 | -0.565 | -0.692 | -0.631 | -0.623 | -0.619 | -0.616 | -0.613 | -0.615 | -0.615 | **-0.618** |
| **SRevTPSS** | -0.604 | -0.606 | -0.599 | -0.599 | -0.625 | -0.585 | -0.610 | -0.613 | -0.588 | -0.614 | -0.617 | -0.556 | -0.683 | -0.620 | -0.614 | -0.608 | -0.605 | -0.602 | -0.603 | -0.604 | **-0.608** |
| **STPSS** | -0.594 | -0.596 | -0.589 | -0.589 | -0.616 | -0.575 | -0.600 | -0.604 | -0.578 | -0.604 | -0.608 | -0.545 | -0.677 | -0.612 | -0.605 | -0.598 | -0.595 | -0.592 | -0.593 | -0.594 | **-0.598** |
| **SVP86** | -0.696 | -0.699 | -0.700 | -0.700 | -0.709 | -0.681 | -0.704 | -0.707 | -0.684 | -0.708 | -0.711 | -0.653 | -0.752 | -0.708 | -0.702 | -0.705 | -0.702 | -0.702 | -0.703 | -0.704 | **-0.701** |
| **SVWN5** | -0.484 | -0.486 | -0.464 | -0.464 | -0.508 | -0.450 | -0.474 | -0.475 | -0.453 | -0.477 | -0.478 | -0.426 | -0.581 | -0.479 | -0.495 | -0.473 | -0.474 | -0.468 | -0.467 | -0.467 | **-0.477** |
| **TPSSB95** | 0.039 | 0.026 | 0.098 | 0.098 | -0.096 | 0.085 | 0.083 | 0.082 | 0.085 | 0.083 | 0.081 | 0.087 | -0.173 | 0.102 | 0.003 | 0.088 | 0.075 | 0.097 | 0.098 | 0.099 | **0.057** |
| **TPSSBRC** | 0.124 | 0.119 | 0.217 | 0.218 | 0.011 | 0.214 | 0.188 | 0.179 | 0.213 | 0.187 | 0.179 | 0.220 | -0.118 | 0.210 | 0.067 | 0.236 | 0.161 | 0.231 | 0.213 | 0.222 | **0.164** |
| **TPSSKCIS** | 0.109 | 0.103 | 0.175 | 0.175 | -0.003 | 0.183 | 0.155 | 0.138 | 0.181 | 0.153 | 0.136 | 0.188 | -0.119 | 0.191 | 0.052 | 0.202 | 0.124 | 0.187 | 0.174 | 0.174 | **0.134** |
| **TPSSLYP** | -0.190 | -0.191 | -0.141 | -0.141 | -0.257 | -0.140 | -0.155 | -0.157 | -0.141 | -0.157 | -0.159 | -0.128 | -0.354 | -0.124 | -0.216 | -0.130 | -0.166 | -0.140 | -0.141 | -0.141 | **-0.168** |
| **TPSSP86** | -0.013 | -0.017 | 0.078 | 0.078 | -0.106 | 0.073 | 0.050 | 0.027 | 0.072 | 0.049 | 0.027 | 0.083 | -0.191 | 0.088 | -0.063 | 0.061 | 0.007 | 0.067 | 0.068 | 0.091 | **0.026** |
| **TPSSPBE** | 0.172 | 0.166 | 0.249 | 0.249 | 0.054 | 0.253 | 0.229 | 0.222 | 0.252 | 0.228 | 0.222 | 0.258 | -0.057 | 0.264 | 0.121 | 0.279 | 0.194 | 0.275 | 0.246 | 0.246 | **0.206** |
| **TPSSPKZB** | 0.181 | 0.184 | 0.252 | 0.252 | 0.058 | 0.256 | 0.233 | 0.225 | 0.255 | 0.231 | 0.224 | 0.264 | -0.053 | 0.267 | 0.125 | 0.283 | 0.199 | 0.281 | 0.249 | 0.250 | **0.211** |
| **TPSSPL** | 0.197 | 0.188 | 0.260 | 0.260 | 0.075 | 0.266 | 0.238 | 0.229 | 0.266 | 0.238 | 0.228 | 0.271 | -0.036 | 0.280 | 0.142 | 0.298 | 0.211 | 0.275 | 0.259 | 0.257 | **0.220** |
| **TPSSPW91** | 0.162 | 0.158 | 0.240 | 0.240 | 0.048 | 0.243 | 0.221 | 0.210 | 0.241 | 0.222 | 0.209 | 0.249 | -0.067 | 0.257 | 0.110 | 0.268 | 0.187 | 0.249 | 0.237 | 0.237 | **0.196** |
| **TPSSRevTPSS** | 0.149 | 0.145 | 0.225 | 0.226 | 0.035 | 0.229 | 0.196 | 0.197 | 0.227 | 0.195 | 0.196 | 0.233 | -0.079 | 0.233 | 0.096 | 0.244 | 0.173 | 0.235 | 0.211 | 0.225 | **0.180** |
| **TPSSTPSS** | 0.180 | 0.183 | 0.252 | 0.252 | 0.057 | 0.257 | 0.232 | 0.225 | 0.256 | 0.232 | 0.225 | 0.264 | -0.054 | 0.268 | 0.124 | 0.285 | 0.199 | 0.280 | 0.249 | 0.250 | **0.211** |
| **TPSSVP86** | -0.012 | -0.016 | 0.080 | 0.080 | -0.105 | 0.075 | 0.051 | 0.029 | 0.073 | 0.050 | 0.028 | 0.085 | -0.190 | 0.090 | -0.061 | 0.063 | 0.009 | 0.069 | 0.070 | 0.093 | **0.028** |
| **TPSSVWN** | 0.165 | 0.160 | 0.220 | 0.220 | 0.045 | 0.224 | 0.199 | 0.189 | 0.223 | 0.197 | 0.189 | 0.230 | -0.052 | 0.239 | 0.110 | 0.255 | 0.179 | 0.230 | 0.219 | 0.217 | **0.183** |
| **TPSSVWN5** | 0.198 | 0.192 | 0.262 | 0.262 | 0.076 | 0.268 | 0.239 | 0.230 | 0.267 | 0.239 | 0.229 | 0.272 | -0.035 | 0.280 | 0.143 | 0.302 | 0.212 | 0.278 | 0.261 | 0.259 | **0.222** |
| **wPBEhB95** | -0.110 | -0.111 | -0.050 | -0.050 | -0.213 | -0.041 | -0.093 | -0.075 | -0.041 | -0.093 | -0.076 | -0.013 | -0.316 | -0.021 | -0.188 | -0.076 | -0.105 | -0.053 | -0.062 | -0.048 | **-0.092** |
| **wPBEhBRC** | -0.049 | -0.052 | 0.029 | 0.029 | -0.136 | 0.034 | 0.012 | 0.005 | 0.032 | 0.011 | 0.004 | 0.044 | -0.264 | 0.028 | -0.088 | 0.040 | -0.013 | 0.036 | 0.028 | 0.033 | **-0.012** |
| **wPBEhKCIS** | -0.084 | -0.089 | -0.008 | -0.008 | -0.164 | -0.004 | -0.022 | -0.030 | -0.005 | -0.023 | -0.032 | 0.007 | -0.271 | 0.013 | -0.120 | -0.003 | -0.051 | -0.007 | -0.009 | -0.006 | **-0.046** |
| **wPBEhLYP** | -0.302 | -0.303 | -0.261 | -0.261 | -0.355 | -0.253 | -0.268 | -0.268 | -0.255 | -0.269 | -0.270 | -0.240 | -0.444 | -0.246 | -0.319 | -0.253 | -0.279 | -0.262 | -0.260 | -0.259 | **-0.281** |
| **wPBEhP86** | -0.218 | -0.221 | -0.165 | -0.165 | -0.272 | -0.154 | -0.180 | -0.184 | -0.155 | -0.182 | -0.187 | -0.137 | -0.363 | -0.159 | -0.242 | -0.172 | -0.193 | -0.173 | -0.171 | -0.170 | **-0.193** |
| **wPBEhPBE** | -0.039 | -0.044 | 0.045 | 0.045 | -0.125 | 0.045 | 0.016 | 0.007 | 0.044 | 0.014 | 0.006 | 0.058 | -0.231 | 0.062 | -0.079 | 0.046 | -0.009 | 0.043 | 0.043 | 0.044 | **0.000** |
| **wPBEhPKZB** | -0.031 | -0.036 | 0.052 | 0.052 | -0.119 | 0.052 | 0.023 | 0.013 | 0.050 | 0.022 | 0.012 | 0.065 | -0.224 | 0.069 | -0.071 | 0.054 | -0.002 | 0.051 | 0.049 | 0.051 | **0.007** |
| **wPBEhPL** | 0.032 | 0.028 | 0.097 | 0.098 | -0.061 | 0.100 | 0.082 | 0.073 | 0.100 | 0.081 | 0.072 | 0.108 | -0.175 | 0.118 | -0.009 | 0.116 | 0.054 | 0.103 | 0.096 | 0.097 | **0.061** |
| **wPBEhPW91** | -0.054 | -0.058 | 0.026 | 0.027 | -0.136 | 0.030 | -0.001 | -0.010 | 0.029 | -0.003 | -0.011 | 0.044 | -0.241 | 0.043 | -0.091 | 0.030 | -0.023 | 0.027 | 0.022 | 0.025 | **-0.016** |
| **wPBEhRevTPSS** | -0.063 | -0.067 | 0.012 | 0.012 | -0.144 | 0.016 | -0.012 | -0.019 | 0.015 | -0.014 | -0.021 | 0.030 | -0.247 | 0.030 | -0.099 | 0.016 | -0.033 | 0.012 | 0.008 | 0.011 | **-0.028** |
| **wPBEhTPSS** | -0.032 | -0.037 | 0.051 | 0.051 | -0.120 | 0.052 | 0.023 | 0.013 | 0.051 | 0.021 | 0.012 | 0.065 | -0.226 | 0.069 | -0.072 | 0.053 | -0.002 | 0.049 | 0.049 | 0.051 | **0.006** |
| **wPBEhVP86** | -0.216 | -0.219 | -0.164 | -0.164 | -0.270 | -0.152 | -0.178 | -0.183 | -0.154 | -0.180 | -0.185 | -0.136 | -0.361 | -0.158 | -0.239 | -0.170 | -0.192 | -0.171 | -0.169 | -0.168 | **-0.192** |
| **wPBEhVWN** | 0.002 | -0.001 | 0.063 | 0.063 | -0.085 | 0.068 | 0.045 | 0.039 | 0.067 | 0.044 | 0.038 | 0.075 | -0.190 | 0.083 | -0.035 | 0.082 | 0.024 | 0.065 | 0.061 | 0.060 | **0.029** |
| **wPBEhVWN5** | 0.033 | 0.029 | 0.099 | 0.099 | -0.060 | 0.102 | 0.083 | 0.074 | 0.101 | 0.083 | 0.073 | 0.110 | -0.173 | 0.119 | -0.008 | 0.117 | 0.055 | 0.104 | 0.098 | 0.097 | **0.062** |
| **XaB95** | -0.679 | -0.680 | -0.679 | -0.679 | -0.692 | -0.646 | -0.683 | -0.688 | -0.650 | -0.686 | -0.691 | -0.610 | -0.755 | -0.675 | -0.683 | -0.684 | -0.682 | -0.683 | -0.682 | -0.684 | **-0.680** |
| **XaKCIS** | -0.645 | -0.647 | -0.645 | -0.645 | -0.660 | -0.626 | -0.649 | -0.653 | -0.629 | -0.653 | -0.657 | -0.598 | -0.712 | -0.656 | -0.652 | -0.651 | -0.650 | -0.648 | -0.648 | -0.649 | **-0.649** |
| **XaBRC** | -0.608 | -0.609 | -0.606 | -0.606 | -0.634 | -0.595 | -0.618 | -0.621 | -0.598 | -0.621 | -0.624 | -0.568 | -0.686 | -0.623 | -0.618 | -0.612 | -0.611 | -0.609 | -0.610 | -0.612 | **-0.614** |
| **XaLYP** | -0.739 | -0.741 | -0.740 | -0.740 | -0.751 | -0.721 | -0.739 | -0.742 | -0.724 | -0.742 | -0.744 | -0.697 | -0.787 | -0.737 | -0.742 | -0.740 | -0.743 | -0.741 | -0.741 | -0.741 | **-0.740** |

| | | | | | | | | | | | | | | | | | | | | | |
|---|---|---|---|---|---|---|---|---|---|---|---|---|---|---|---|---|---|---|---|---|---|
| **XaP86** | -0.735 | -0.738 | -0.744 | -0.744 | -0.746 | -0.724 | -0.747 | -0.750 | -0.728 | -0.751 | -0.754 | -0.695 | -0.781 | -0.749 | -0.740 | -0.748 | -0.745 | -0.746 | -0.747 | -0.748 | **-0.743** |
| **XaPBE** | -0.649 | -0.651 | -0.654 | -0.654 | -0.666 | -0.635 | -0.662 | -0.666 | -0.640 | -0.666 | -0.670 | -0.604 | -0.719 | -0.672 | -0.658 | -0.661 | -0.657 | -0.656 | -0.658 | -0.659 | **-0.658** |
| **XaPKZB** | -0.635 | -0.638 | -0.638 | -0.638 | -0.653 | -0.622 | -0.647 | -0.651 | -0.626 | -0.651 | -0.655 | -0.592 | -0.706 | -0.657 | -0.644 | -0.646 | -0.641 | -0.640 | -0.642 | -0.643 | **-0.643** |
| **XaPL** | -0.533 | -0.534 | -0.521 | -0.521 | -0.552 | -0.507 | -0.530 | -0.530 | -0.511 | -0.534 | -0.534 | -0.482 | -0.615 | -0.536 | -0.541 | -0.529 | -0.528 | -0.525 | -0.524 | -0.524 | **-0.531** |
| **XaPW91** | -0.656 | -0.659 | -0.662 | -0.662 | -0.672 | -0.643 | -0.669 | -0.672 | -0.647 | -0.673 | -0.676 | -0.612 | -0.723 | -0.678 | -0.665 | -0.668 | -0.664 | -0.664 | -0.665 | -0.666 | **-0.665** |
| **XaRevTPSS** | -0.647 | -0.649 | -0.650 | -0.650 | -0.663 | -0.634 | -0.658 | -0.661 | -0.637 | -0.662 | -0.665 | -0.604 | -0.714 | -0.666 | -0.655 | -0.657 | -0.653 | -0.653 | -0.653 | -0.654 | **-0.654** |
| **XaTPSS** | -0.638 | -0.640 | -0.641 | -0.641 | -0.655 | -0.624 | -0.649 | -0.653 | -0.628 | -0.654 | -0.657 | -0.594 | -0.708 | -0.659 | -0.647 | -0.648 | -0.644 | -0.643 | -0.645 | -0.645 | **-0.646** |
| **XaVP86** | -0.685 | -0.688 | -0.689 | -0.689 | -0.746 | -0.671 | -0.696 | -0.698 | -0.728 | -0.699 | -0.700 | -0.695 | -0.741 | -0.704 | -0.689 | -0.696 | -0.692 | -0.693 | -0.694 | -0.695 | **-0.699** |
| **XaVWN** | -0.551 | -0.552 | -0.542 | -0.542 | -0.568 | -0.466 | -0.486 | -0.551 | -0.469 | -0.490 | -0.555 | -0.441 | -0.628 | -0.471 | -0.558 | -0.550 | -0.548 | -0.545 | -0.544 | -0.545 | **-0.530** |
| **XaVWN5** | -0.532 | -0.534 | -0.433 | -0.433 | -0.551 | -0.443 | -0.465 | -0.530 | -0.447 | -0.468 | -0.534 | -0.418 | -0.615 | -0.449 | -0.540 | -0.529 | -0.440 | -0.524 | -0.523 | -0.523 | **-0.497** |
| **LC-BB95** | -0.004 | -0.015 | 0.031 | 0.031 | -0.126 | 0.018 | 0.017 | 0.016 | 0.018 | 0.016 | 0.016 | 0.019 | -0.213 | 0.005 | -0.067 | 0.019 | -0.001 | 0.029 | 0.031 | 0.033 | **-0.006** |
| **LC-BBRC** | 0.062 | 0.058 | 0.361 | 0.361 | -0.057 | 0.324 | 0.251 | 0.223 | 0.329 | 0.249 | 0.222 | 0.348 | -0.263 | 0.316 | 0.029 | 0.268 | 0.215 | 0.378 | 0.360 | 0.437 | **0.224** |
| **LC-BKCIS** | 0.021 | 0.014 | 0.184 | 0.184 | -0.104 | 0.199 | 0.132 | 0.112 | 0.198 | 0.130 | 0.111 | 0.189 | -0.278 | 0.169 | -0.033 | 0.139 | 0.105 | 0.183 | 0.181 | 0.206 | **0.102** |
| **LC-BLYP** | -0.347 | -0.348 | -0.302 | -0.301 | -0.405 | -0.305 | -0.320 | -0.318 | -0.307 | -0.322 | -0.320 | -0.296 | -0.499 | -0.319 | -0.361 | -0.306 | -0.317 | -0.300 | -0.303 | -0.301 | **-0.330** |
| **LC-BP86** | -0.173 | -0.174 | 0.435 | 0.431 | -0.238 | 0.179 | 0.082 | 0.037 | 0.177 | 0.080 | 0.041 | 0.193 | -0.273 | 0.178 | -0.093 | 0.014 | 0.186 | 0.279 | 0.415 | 0.440 | **0.111** |
| **LC-BPBE** | 0.141 | 0.134 | 0.571 | 0.570 | 0.028 | 0.488 | 0.458 | 0.378 | 0.484 | 0.458 | 0.379 | 0.485 | -0.181 | 0.435 | 0.120 | 0.456 | 0.345 | 0.543 | 0.572 | 0.588 | **0.373** |
| **LC-BPKZB** | 0.149 | 0.142 | 0.568 | 0.571 | 0.041 | 0.486 | 0.460 | 0.381 | 0.482 | 0.458 | 0.382 | 0.497 | -0.155 | 0.445 | 0.151 | 0.487 | 0.346 | 0.542 | 0.573 | 0.590 | **0.380** |
| **LC-BPL** | 0.209 | 0.205 | 0.494 | 0.494 | 0.081 | 0.457 | 0.397 | 0.354 | 0.457 | 0.389 | 0.353 | 0.456 | -0.109 | 0.458 | 0.173 | 0.480 | 0.329 | 0.514 | 0.522 | 0.558 | **0.364** |
| **LC-BPW91** | 0.123 | 0.114 | 0.563 | 0.563 | -0.009 | 0.476 | 0.409 | 0.328 | 0.470 | 0.392 | 0.314 | 0.472 | -0.204 | 0.418 | 0.075 | 0.410 | 0.332 | 0.539 | 0.591 | 0.581 | **0.348** |
| **LC-BRevTPSS** | 0.094 | 0.088 | 0.517 | 0.519 | -0.041 | 0.444 | 0.304 | 0.250 | 0.437 | 0.308 | 0.243 | 0.437 | -0.211 | 0.390 | 0.044 | 0.392 | 0.305 | 0.463 | 0.509 | 0.543 | **0.302** |
| **LC-BTPSS** | 0.145 | 0.139 | 0.571 | 0.570 | 0.047 | 0.486 | 0.458 | 0.383 | 0.482 | 0.458 | 0.382 | 0.490 | -0.172 | 0.440 | 0.139 | 0.484 | 0.346 | 0.544 | 0.573 | 0.601 | **0.378** |
| **LC-BVP86** | -0.170 | -0.171 | 0.447 | 0.443 | -0.234 | 0.189 | 0.080 | 0.042 | 0.184 | 0.084 | 0.040 | 0.210 | -0.272 | 0.183 | -0.086 | 0.016 | 0.189 | 0.295 | 0.420 | 0.443 | **0.117** |
| **LC-BVWN** | 0.163 | 0.150 | 0.381 | 0.381 | 0.019 | 0.354 | 0.294 | 0.268 | 0.353 | 0.293 | 0.268 | 0.355 | -0.139 | 0.358 | 0.121 | 0.341 | 0.263 | 0.402 | 0.385 | 0.413 | **0.271** |
| **LC-BVWN5** | 0.211 | 0.207 | 0.509 | 0.509 | 0.084 | 0.471 | 0.403 | 0.364 | 0.470 | 0.402 | 0.364 | 0.466 | -0.107 | 0.461 | 0.177 | 0.504 | 0.343 | 0.551 | 0.526 | 0.604 | **0.376** |
| **LC-BRxB95** | -0.014 | -0.015 | 0.032 | 0.032 | -0.126 | 0.019 | 0.017 | 0.016 | 0.019 | 0.017 | 0.016 | 0.020 | -0.212 | 0.031 | -0.066 | 0.019 | 0.001 | 0.030 | 0.033 | 0.034 | **-0.005** |
| **LC-BRxBRC** | 0.055 | 0.051 | 0.367 | 0.367 | -0.058 | 0.295 | 0.227 | 0.188 | 0.299 | 0.227 | 0.187 | 0.302 | -0.266 | 0.273 | 0.025 | 0.240 | 0.217 | 0.368 | 0.361 | 0.433 | **0.208** |
| **LC-BRxKCIS** | 0.014 | 0.008 | 0.181 | 0.181 | -0.107 | 0.144 | 0.112 | 0.091 | 0.143 | 0.111 | 0.090 | 0.148 | -0.280 | 0.139 | -0.040 | 0.105 | 0.094 | 0.175 | 0.176 | 0.186 | **0.084** |
| **LC-BRxLYP** | -0.360 | -0.360 | -0.319 | -0.319 | -0.414 | -0.327 | -0.342 | -0.339 | -0.329 | -0.343 | -0.341 | -0.317 | -0.504 | -0.340 | -0.373 | -0.329 | -0.333 | -0.320 | -0.321 | -0.320 | **-0.347** |
| **LC-BRxP86** | -0.187 | -0.189 | 0.435 | 0.435 | -0.252 | 0.164 | 0.031 | -0.036 | 0.163 | 0.011 | -0.038 | 0.173 | -0.280 | 0.168 | -0.108 | -0.015 | 0.180 | 0.230 | 0.413 | 0.429 | **0.086** |
| **LC-BRxPBE** | 0.138 | 0.131 | 0.579 | 0.578 | 0.029 | 0.487 | 0.364 | 0.326 | 0.475 | 0.358 | 0.307 | 0.478 | -0.188 | 0.430 | 0.109 | 0.409 | 0.347 | 0.523 | 0.574 | 0.605 | **0.353** |
| **LC-BRxPKZB** | 0.155 | 0.139 | 0.563 | 0.562 | 0.035 | 0.489 | 0.392 | 0.325 | 0.477 | 0.390 | 0.323 | 0.491 | -0.159 | 0.440 | 0.129 | 0.478 | 0.350 | 0.525 | 0.577 | 0.592 | **0.364** |
| **LC-BRxPL** | 0.211 | 0.207 | 0.502 | 0.502 | 0.073 | 0.448 | 0.366 | 0.324 | 0.448 | 0.367 | 0.323 | 0.445 | -0.105 | 0.479 | 0.178 | 0.441 | 0.355 | 0.521 | 0.544 | 0.540 | **0.358** |
| **LC-BRxPW91** | 0.121 | 0.111 | 0.540 | 0.540 | -0.005 | 0.447 | 0.328 | 0.245 | 0.443 | 0.323 | 0.244 | 0.461 | -0.212 | 0.403 | 0.058 | 0.377 | 0.338 | 0.513 | 0.559 | 0.562 | **0.320** |
| **LC-BRxRevTPSS** | 0.089 | 0.084 | 0.489 | 0.490 | -0.042 | 0.370 | 0.269 | 0.217 | 0.368 | 0.264 | 0.216 | 0.388 | -0.217 | 0.353 | 0.037 | 0.302 | 0.304 | 0.459 | 0.500 | 0.545 | **0.274** |
| **LC-BRxTPSS** | 0.142 | 0.136 | 0.577 | 0.579 | 0.035 | 0.489 | 0.392 | 0.329 | 0.478 | 0.388 | 0.308 | 0.493 | -0.162 | 0.440 | 0.116 | 0.451 | 0.348 | 0.539 | 0.580 | 0.591 | **0.363** |
| **LC-BRxVP86** | -0.185 | -0.186 | 0.431 | 0.426 | -0.248 | 0.167 | 0.015 | -0.035 | 0.167 | 0.017 | -0.035 | 0.181 | -0.286 | 0.170 | -0.105 | -0.011 | 0.181 | 0.265 | 0.410 | 0.441 | **0.089** |
| **LC-BRxVWN** | 0.162 | 0.152 | 0.377 | 0.377 | 0.022 | 0.335 | 0.268 | 0.247 | 0.334 | 0.267 | 0.247 | 0.338 | -0.135 | 0.343 | 0.110 | 0.314 | 0.265 | 0.373 | 0.374 | 0.418 | **0.259** |
| **LC-BRxVWN5** | 0.214 | 0.212 | 0.504 | 0.504 | 0.078 | 0.450 | 0.370 | 0.328 | 0.450 | 0.369 | 0.329 | 0.448 | -0.102 | 0.481 | 0.181 | 0.446 | 0.335 | 0.528 | 0.529 | 0.560 | **0.361** |
| **LC-G96B95** | -0.004 | -0.004 | 0.031 | 0.031 | -0.126 | 0.018 | 0.017 | 0.016 | 0.018 | 0.016 | 0.016 | 0.019 | -0.213 | 0.005 | -0.067 | 0.019 | 0.000 | 0.029 | 0.031 | 0.033 | **-0.006** |
| **LC-G96BRC** | 0.064 | 0.060 | 0.366 | 0.366 | -0.054 | 0.336 | 0.251 | 0.229 | 0.334 | 0.250 | 0.226 | 0.338 | -0.260 | 0.320 | 0.032 | 0.272 | 0.217 | 0.384 | 0.375 | 0.439 | **0.227** |
| **LC-G96KCIS** | 0.020 | 0.013 | 0.190 | 0.190 | -0.102 | 0.200 | 0.133 | 0.112 | 0.199 | 0.131 | 0.111 | 0.191 | -0.277 | 0.171 | -0.032 | 0.140 | 0.114 | 0.184 | 0.187 | 0.221 | **0.105** |
| **LC-G96LYP** | -0.347 | -0.347 | -0.301 | -0.301 | -0.405 | -0.305 | -0.320 | -0.318 | -0.307 | -0.322 | -0.319 | -0.296 | -0.498 | -0.319 | -0.361 | -0.307 | -0.317 | -0.301 | -0.302 | -0.300 | **-0.330** |
| **LC-G96P86** | -0.170 | -0.172 | 0.435 | 0.434 | -0.233 | 0.188 | 0.083 | 0.041 | 0.179 | 0.082 | 0.043 | 0.209 | -0.272 | 0.182 | -0.084 | 0.016 | 0.190 | 0.256 | 0.414 | 0.445 | **0.113** |
| **LC-G96PBE** | 0.149 | 0.134 | 0.571 | 0.571 | 0.031 | 0.485 | 0.458 | 0.379 | 0.499 | 0.457 | 0.374 | 0.482 | -0.179 | 0.438 | 0.132 | 0.469 | 0.346 | 0.543 | 0.572 | 0.590 | **0.375** |
| **LC-G96PKZB** | 0.152 | 0.142 | 0.571 | 0.571 | 0.055 | 0.497 | 0.459 | 0.381 | 0.487 | 0.460 | 0.381 | 0.486 | -0.152 | 0.448 | 0.155 | 0.475 | 0.349 | 0.544 | 0.573 | 0.588 | **0.381** |
| **LC-G96PL** | 0.210 | 0.205 | 0.495 | 0.495 | 0.082 | 0.457 | 0.395 | 0.353 | 0.456 | 0.393 | 0.352 | 0.460 | -0.107 | 0.459 | 0.175 | 0.480 | 0.330 | 0.514 | 0.507 | 0.526 | **0.362** |
| **LC-G96PW91** | 0.124 | 0.115 | 0.565 | 0.564 | 0.010 | 0.474 | 0.419 | 0.328 | 0.471 | 0.416 | 0.336 | 0.471 | -0.198 | 0.421 | 0.078 | 0.444 | 0.336 | 0.536 | 0.575 | 0.586 | **0.354** |
| **LC-G96RevTPSS** | 0.096 | 0.090 | 0.521 | 0.523 | -0.024 | 0.441 | 0.322 | 0.263 | 0.439 | 0.304 | 0.261 | 0.418 | -0.208 | 0.394 | 0.048 | 0.389 | 0.307 | 0.465 | 0.512 | 0.546 | **0.305** |
| **LC-G96TPSS** | 0.155 | 0.141 | 0.572 | 0.571 | 0.042 | 0.487 | 0.475 | 0.381 | 0.482 | 0.459 | 0.383 | 0.487 | -0.168 | 0.441 | 0.150 | 0.476 | 0.356 | 0.544 | 0.574 | 0.616 | **0.381** |
| **LC-G96VP86** | -0.166 | -0.168 | 0.448 | 0.448 | -0.232 | 0.194 | 0.080 | 0.054 | 0.186 | 0.080 | 0.041 | 0.211 | -0.271 | 0.188 | -0.083 | 0.018 | 0.192 | 0.295 | 0.419 | 0.447 | **0.119** |
| **LC-G96VWN** | 0.164 | 0.152 | 0.384 | 0.384 | 0.021 | 0.355 | 0.294 | 0.268 | 0.353 | 0.293 | 0.268 | 0.354 | -0.137 | 0.358 | 0.123 | 0.342 | 0.264 | 0.406 | 0.386 | 0.416 | **0.272** |
| **LC-G96VWN5** | 0.212 | 0.209 | 0.509 | 0.509 | 0.088 | 0.468 | 0.405 | 0.360 | 0.469 | 0.402 | 0.359 | 0.466 | -0.105 | 0.461 | 0.178 | 0.503 | 0.344 | 0.550 | 0.527 | 0.574 | **0.374** |
| **LC-LGB95** | -0.015 | -0.015 | 0.031 | 0.031 | -0.127 | 0.018 | 0.016 | 0.016 | 0.018 | 0.016 | 0.016 | 0.019 | -0.213 | 0.005 | -0.068 | 0.018 | -0.001 | 0.029 | 0.031 | 0.033 | **-0.007** |
| **LC-LGBRC** | 0.058 | 0.053 | 0.362 | 0.363 | -0.061 | 0.325 | 0.255 | 0.224 | 0.321 | 0.283 | 0.223 | 0.327 | -0.267 | 0.308 | 0.030 | 0.270 | 0.213 | 0.371 | 0.358 | 0.432 | **0.222** |
| **LC-LGKCIS** | 0.018 | 0.011 | 0.186 | 0.186 | -0.108 | 0.198 | 0.134 | 0.113 | 0.196 | 0.132 | 0.113 | 0.190 | -0.283 | 0.155 | -0.037 | 0.126 | 0.097 | 0.179 | 0.182 | 0.204 | **0.100** |
| **LC-LGLYP** | -0.348 | -0.349 | -0.301 | -0.301 | -0.407 | -0.305 | -0.320 | -0.318 | -0.307 | -0.322 | -0.320 | -0.296 | -0.500 | -0.320 | -0.362 | -0.307 | -0.318 | -0.302 | -0.302 | -0.302 | **-0.331** |
| **LC-LGP86** | -0.177 | -0.178 | 0.427 | 0.429 | -0.244 | 0.185 | 0.070 | 0.033 | 0.174 | 0.066 | 0.031 | 0.197 | -0.276 | 0.176 | -0.092 | 0.010 | 0.100 | 0.224 | 0.407 | 0.437 | **0.100** |

| | | | | | | | | | | | | | | | | | | | | | |
|---|---|---|---|---|---|---|---|---|---|---|---|---|---|---|---|---|---|---|---|---|---|
| **LC-LGPBE** | 0.138 | 0.131 | 0.561 | 0.561 | 0.023 | 0.491 | 0.457 | 0.378 | 0.488 | 0.458 | 0.378 | 0.490 | -0.188 | 0.462 | 0.114 | 0.486 | 0.342 | 0.563 | 0.571 | 0.590 | **0.375** |
| **LC-LGPKZB** | 0.144 | 0.137 | 0.562 | 0.563 | 0.022 | 0.484 | 0.459 | 0.387 | 0.491 | 0.460 | 0.386 | 0.487 | -0.162 | 0.492 | 0.143 | 0.490 | 0.345 | 0.563 | 0.574 | 0.594 | **0.381** |
| **LC-LGPL** | 0.208 | 0.203 | 0.509 | 0.509 | 0.069 | 0.470 | 0.398 | 0.360 | 0.470 | 0.398 | 0.382 | 0.462 | -0.113 | 0.485 | 0.171 | 0.484 | 0.330 | 0.532 | 0.527 | 0.548 | **0.370** |
| **LC-LGPW91** | 0.120 | 0.110 | 0.558 | 0.555 | -0.015 | 0.476 | 0.414 | 0.324 | 0.474 | 0.414 | 0.326 | 0.479 | -0.212 | 0.448 | 0.069 | 0.423 | 0.330 | 0.534 | 0.547 | 0.582 | **0.348** |
| **LC-LGRevTPSS** | 0.091 | 0.085 | 0.510 | 0.508 | -0.044 | 0.441 | 0.333 | 0.248 | 0.415 | 0.334 | 0.251 | 0.441 | -0.218 | 0.409 | 0.040 | 0.380 | 0.302 | 0.465 | 0.509 | 0.551 | **0.302** |
| **LC-LGTPSS** | 0.139 | 0.137 | 0.563 | 0.563 | 0.027 | 0.486 | 0.459 | 0.385 | 0.482 | 0.459 | 0.386 | 0.483 | -0.178 | 0.469 | 0.132 | 0.491 | 0.343 | 0.564 | 0.568 | 0.592 | **0.377** |
| **LC-LGVP86** | -0.175 | -0.175 | 0.442 | 0.438 | -0.241 | 0.194 | 0.076 | 0.036 | 0.195 | 0.074 | 0.033 | 0.202 | -0.276 | 0.153 | -0.089 | 0.012 | 0.179 | 0.275 | 0.419 | 0.438 | **0.111** |
| **LC-LGVWN** | 0.162 | 0.151 | 0.391 | 0.391 | 0.016 | 0.356 | 0.298 | 0.272 | 0.354 | 0.298 | 0.272 | 0.363 | -0.143 | 0.356 | 0.112 | 0.341 | 0.263 | 0.401 | 0.388 | 0.417 | **0.273** |
| **LC-LGVWN5** | 0.211 | 0.207 | 0.524 | 0.524 | 0.076 | 0.479 | 0.408 | 0.370 | 0.478 | 0.409 | 0.366 | 0.471 | -0.111 | 0.490 | 0.174 | 0.512 | 0.337 | 0.552 | 0.532 | 0.567 | **0.379** |
| **LC-mPWB95** | -0.015 | -0.015 | 0.031 | 0.031 | -0.127 | 0.018 | 0.016 | 0.016 | 0.018 | 0.016 | 0.016 | 0.019 | -0.213 | 0.005 | -0.067 | 0.019 | -0.001 | 0.029 | 0.031 | 0.033 | **-0.007** |
| **LC-mPWBRC** | 0.062 | 0.057 | 0.360 | 0.360 | -0.057 | 0.329 | 0.249 | 0.222 | 0.317 | 0.249 | 0.221 | 0.332 | -0.263 | 0.303 | 0.028 | 0.268 | 0.214 | 0.377 | 0.360 | 0.435 | **0.221** |
| **LC-mPWKCIS** | 0.020 | 0.013 | 0.184 | 0.184 | -0.104 | 0.198 | 0.130 | 0.110 | 0.196 | 0.129 | 0.109 | 0.188 | -0.280 | 0.155 | -0.034 | 0.137 | 0.104 | 0.182 | 0.180 | 0.204 | **0.100** |
| **LC-mPWLYP** | -0.348 | -0.349 | -0.303 | -0.303 | -0.406 | -0.306 | -0.321 | -0.319 | -0.308 | -0.323 | -0.321 | -0.297 | -0.499 | -0.320 | -0.362 | -0.308 | -0.318 | -0.301 | -0.304 | -0.302 | **-0.331** |
| **LC-mPWP86** | -0.174 | -0.175 | 0.434 | 0.430 | -0.240 | 0.177 | 0.080 | 0.039 | 0.182 | 0.078 | 0.040 | 0.191 | -0.274 | 0.179 | -0.095 | 0.012 | 0.184 | 0.277 | 0.415 | 0.439 | **0.110** |
| **LC-mPWPBE** | 0.142 | 0.133 | 0.573 | 0.574 | 0.027 | 0.490 | 0.458 | 0.377 | 0.480 | 0.458 | 0.372 | 0.483 | -0.182 | 0.437 | 0.127 | 0.456 | 0.343 | 0.564 | 0.571 | 0.588 | **0.374** |
| **LC-mPWPKZB** | 0.148 | 0.140 | 0.588 | 0.571 | 0.039 | 0.496 | 0.475 | 0.382 | 0.485 | 0.455 | 0.382 | 0.489 | -0.156 | 0.447 | 0.140 | 0.474 | 0.347 | 0.566 | 0.571 | 0.590 | **0.382** |
| **LC-mPWPL** | 0.208 | 0.205 | 0.495 | 0.495 | 0.079 | 0.456 | 0.388 | 0.353 | 0.456 | 0.388 | 0.352 | 0.454 | -0.110 | 0.457 | 0.172 | 0.479 | 0.329 | 0.514 | 0.506 | 0.557 | **0.362** |
| **LC-mPWPW91** | 0.121 | 0.112 | 0.563 | 0.563 | -0.008 | 0.471 | 0.399 | 0.326 | 0.470 | 0.391 | 0.341 | 0.485 | -0.204 | 0.419 | 0.080 | 0.401 | 0.332 | 0.537 | 0.553 | 0.580 | **0.347** |
| **LC-mPWRevTPSS** | 0.094 | 0.087 | 0.518 | 0.518 | -0.040 | 0.438 | 0.302 | 0.242 | 0.433 | 0.320 | 0.249 | 0.416 | -0.213 | 0.412 | 0.043 | 0.389 | 0.304 | 0.466 | 0.510 | 0.549 | **0.302** |
| **LC-mPWTPSS** | 0.141 | 0.138 | 0.588 | 0.589 | 0.049 | 0.490 | 0.458 | 0.382 | 0.483 | 0.473 | 0.381 | 0.490 | -0.172 | 0.443 | 0.133 | 0.475 | 0.345 | 0.563 | 0.590 | 0.591 | **0.382** |
| **LC-mPWVP86** | -0.171 | -0.172 | 0.442 | 0.446 | -0.235 | 0.183 | 0.085 | 0.044 | 0.182 | 0.083 | 0.037 | 0.197 | -0.273 | 0.180 | -0.086 | 0.015 | 0.187 | 0.294 | 0.417 | 0.440 | **0.115** |
| **LC-mPWVWN** | 0.163 | 0.149 | 0.380 | 0.381 | 0.019 | 0.353 | 0.293 | 0.267 | 0.352 | 0.293 | 0.267 | 0.354 | -0.139 | 0.356 | 0.120 | 0.340 | 0.263 | 0.396 | 0.384 | 0.412 | **0.270** |
| **LC-mPWVWN5** | 0.211 | 0.207 | 0.508 | 0.508 | 0.083 | 0.470 | 0.402 | 0.361 | 0.469 | 0.401 | 0.359 | 0.465 | -0.108 | 0.460 | 0.176 | 0.504 | 0.338 | 0.550 | 0.527 | 0.579 | **0.373** |
| **LC-OB95** | 0.007 | 0.007 | 0.030 | 0.030 | -0.141 | 0.017 | 0.016 | 0.015 | 0.017 | 0.016 | 0.015 | 0.018 | -0.212 | 0.030 | -0.067 | 0.019 | -0.001 | 0.029 | 0.030 | 0.033 | **-0.005** |
| **LC-OBRC** | 0.066 | 0.062 | 0.357 | 0.357 | -0.052 | 0.352 | 0.254 | 0.230 | 0.350 | 0.253 | 0.229 | 0.353 | -0.257 | 0.321 | 0.035 | 0.281 | 0.213 | 0.380 | 0.358 | 0.434 | **0.229** |
| **LC-OKCIS** | 0.023 | 0.016 | 0.185 | 0.185 | -0.101 | 0.201 | 0.134 | 0.114 | 0.200 | 0.132 | 0.113 | 0.193 | -0.274 | 0.178 | -0.030 | 0.147 | 0.104 | 0.182 | 0.186 | 0.202 | **0.105** |
| **LC-OLYP** | -0.345 | -0.346 | -0.300 | -0.301 | -0.404 | -0.304 | -0.318 | -0.317 | -0.305 | -0.321 | -0.318 | -0.294 | -0.495 | -0.322 | -0.359 | -0.303 | -0.317 | -0.300 | -0.301 | -0.300 | **-0.329** |
| **LC-OP86** | -0.163 | -0.165 | 0.436 | 0.432 | -0.229 | 0.195 | 0.090 | 0.061 | 0.190 | 0.092 | 0.044 | 0.214 | -0.268 | 0.182 | -0.080 | 0.024 | 0.111 | 0.297 | 0.425 | 0.448 | **0.117** |
| **LC-OPBE** | 0.156 | 0.140 | 0.570 | 0.570 | 0.052 | 0.519 | 0.460 | 0.378 | 0.501 | 0.461 | 0.377 | 0.499 | -0.172 | 0.472 | 0.128 | 0.487 | 0.345 | 0.560 | 0.573 | 0.589 | **0.383** |
| **LC-OPKZB** | 0.166 | 0.160 | 0.571 | 0.571 | 0.056 | 0.504 | 0.463 | 0.383 | 0.500 | 0.462 | 0.385 | 0.503 | -0.147 | 0.480 | 0.156 | 0.488 | 0.345 | 0.541 | 0.574 | 0.592 | **0.388** |
| **LC-OPL** | 0.209 | 0.204 | 0.491 | 0.491 | 0.079 | 0.464 | 0.400 | 0.356 | 0.464 | 0.399 | 0.354 | 0.460 | -0.105 | 0.463 | 0.173 | 0.487 | 0.326 | 0.509 | 0.503 | 0.522 | **0.362** |
| **LC-OPW91** | 0.128 | 0.120 | 0.545 | 0.560 | -0.004 | 0.489 | 0.437 | 0.346 | 0.488 | 0.424 | 0.326 | 0.491 | -0.194 | 0.427 | 0.094 | 0.443 | 0.332 | 0.536 | 0.577 | 0.571 | **0.357** |
| **LC-ORevTPSS** | 0.098 | 0.093 | 0.481 | 0.481 | -0.035 | 0.479 | 0.337 | 0.307 | 0.480 | 0.336 | 0.286 | 0.481 | -0.203 | 0.389 | 0.047 | 0.388 | 0.303 | 0.464 | 0.486 | 0.550 | **0.312** |
| **LC-OTPSS** | 0.152 | 0.142 | 0.572 | 0.572 | 0.050 | 0.501 | 0.463 | 0.387 | 0.503 | 0.461 | 0.385 | 0.503 | -0.163 | 0.480 | 0.154 | 0.491 | 0.352 | 0.542 | 0.578 | 0.591 | **0.386** |
| **LC-OVP86** | -0.161 | -0.162 | 0.441 | 0.437 | -0.227 | 0.210 | 0.091 | 0.047 | 0.200 | 0.092 | 0.045 | 0.210 | -0.267 | 0.200 | -0.077 | 0.026 | 0.194 | 0.299 | 0.447 | 0.482 | **0.126** |
| **LC-OVWN** | 0.162 | 0.149 | 0.377 | 0.377 | 0.018 | 0.353 | 0.294 | 0.267 | 0.352 | 0.293 | 0.266 | 0.355 | -0.134 | 0.362 | 0.121 | 0.349 | 0.261 | 0.391 | 0.408 | 0.410 | **0.272** |
| **LC-OVWN5** | 0.211 | 0.207 | 0.497 | 0.497 | 0.086 | 0.463 | 0.412 | 0.362 | 0.463 | 0.407 | 0.359 | 0.465 | -0.103 | 0.465 | 0.176 | 0.510 | 0.329 | 0.545 | 0.508 | 0.541 | **0.370** |
| **LC-PBEB95** | -0.015 | -0.015 | 0.031 | 0.031 | -0.127 | 0.018 | 0.016 | 0.016 | 0.018 | 0.016 | 0.016 | 0.019 | -0.213 | 0.005 | -0.068 | 0.019 | -0.001 | 0.029 | 0.031 | 0.033 | **-0.007** |
| **LC-PBEBRC** | 0.060 | 0.055 | 0.360 | 0.360 | -0.060 | 0.319 | 0.251 | 0.223 | 0.328 | 0.248 | 0.221 | 0.332 | -0.265 | 0.312 | 0.028 | 0.269 | 0.212 | 0.375 | 0.357 | 0.434 | **0.221** |
| **LC-PBEKCIS** | 0.019 | 0.012 | 0.183 | 0.183 | -0.105 | 0.197 | 0.132 | 0.111 | 0.195 | 0.131 | 0.111 | 0.188 | -0.281 | 0.154 | -0.035 | 0.127 | 0.102 | 0.181 | 0.180 | 0.205 | **0.099** |
| **LC-PBELYP** | -0.348 | -0.348 | -0.302 | -0.302 | -0.406 | -0.305 | -0.321 | -0.318 | -0.307 | -0.322 | -0.320 | -0.296 | -0.499 | -0.320 | -0.362 | -0.307 | -0.318 | -0.301 | -0.303 | -0.301 | **-0.330** |
| **LC-PBEP86** | -0.175 | -0.176 | 0.431 | 0.432 | -0.241 | 0.195 | 0.078 | 0.038 | 0.187 | 0.077 | 0.036 | 0.207 | -0.275 | 0.173 | -0.089 | 0.009 | 0.180 | 0.276 | 0.414 | 0.439 | **0.111** |
| **LC-PBEPBE** | 0.144 | 0.132 | 0.572 | 0.569 | 0.026 | 0.484 | 0.459 | 0.378 | 0.498 | 0.457 | 0.377 | 0.495 | -0.184 | 0.436 | 0.117 | 0.457 | 0.342 | 0.562 | 0.570 | 0.589 | **0.374** |
| **LC-PBEPKZB** | 0.154 | 0.140 | 0.570 | 0.568 | 0.038 | 0.487 | 0.457 | 0.382 | 0.483 | 0.456 | 0.382 | 0.486 | -0.172 | 0.439 | 0.147 | 0.476 | 0.344 | 0.566 | 0.572 | 0.590 | **0.378** |
| **LC-PBEPL** | 0.207 | 0.203 | 0.494 | 0.494 | 0.073 | 0.463 | 0.389 | 0.354 | 0.457 | 0.389 | 0.353 | 0.455 | -0.111 | 0.457 | 0.171 | 0.480 | 0.328 | 0.529 | 0.523 | 0.558 | **0.363** |
| **LC-PBEPW91** | 0.120 | 0.110 | 0.545 | 0.544 | -0.012 | 0.472 | 0.393 | 0.311 | 0.471 | 0.392 | 0.319 | 0.485 | -0.207 | 0.420 | 0.075 | 0.402 | 0.330 | 0.535 | 0.554 | 0.580 | **0.342** |
| **LC-PBERevTPSS** | 0.092 | 0.086 | 0.498 | 0.494 | -0.042 | 0.434 | 0.321 | 0.251 | 0.434 | 0.301 | 0.237 | 0.435 | -0.214 | 0.392 | 0.041 | 0.389 | 0.302 | 0.463 | 0.506 | 0.546 | **0.298** |
| **LC-PBETPSS** | 0.143 | 0.137 | 0.570 | 0.571 | 0.030 | 0.485 | 0.457 | 0.383 | 0.483 | 0.458 | 0.382 | 0.487 | -0.175 | 0.440 | 0.145 | 0.485 | 0.344 | 0.565 | 0.572 | 0.595 | **0.378** |
| **LC-PBEVP86** | -0.172 | -0.174 | 0.446 | 0.446 | -0.238 | 0.182 | 0.081 | 0.038 | 0.179 | 0.080 | 0.035 | 0.193 | -0.191 | 0.180 | -0.087 | 0.015 | 0.185 | 0.279 | 0.413 | 0.441 | **0.117** |
| **LC-PBEVWN** | 0.162 | 0.149 | 0.383 | 0.383 | 0.016 | 0.353 | 0.294 | 0.268 | 0.353 | 0.293 | 0.268 | 0.355 | -0.140 | 0.356 | 0.119 | 0.337 | 0.262 | 0.395 | 0.383 | 0.413 | **0.270** |
| **LC-PBEVWN5** | 0.209 | 0.206 | 0.508 | 0.508 | 0.082 | 0.471 | 0.408 | 0.364 | 0.470 | 0.407 | 0.363 | 0.466 | -0.109 | 0.460 | 0.174 | 0.504 | 0.331 | 0.550 | 0.529 | 0.598 | **0.375** |
| **LC-PBEhB95** | -0.015 | -0.015 | 0.031 | 0.031 | -0.127 | 0.018 | 0.016 | 0.016 | 0.018 | 0.016 | 0.016 | 0.019 | -0.213 | 0.005 | -0.068 | 0.019 | -0.001 | 0.029 | 0.031 | 0.033 | **-0.007** |
| **LC-PBEhBRC** | 0.060 | 0.055 | 0.360 | 0.360 | -0.059 | 0.324 | 0.250 | 0.226 | 0.318 | 0.247 | 0.221 | 0.327 | -0.265 | 0.312 | 0.027 | 0.267 | 0.213 | 0.364 | 0.358 | 0.434 | **0.220** |
| **LC-PBEhKCIS** | 0.019 | 0.012 | 0.183 | 0.183 | -0.106 | 0.197 | 0.133 | 0.112 | 0.196 | 0.130 | 0.111 | 0.188 | -0.280 | 0.155 | -0.036 | 0.126 | 0.100 | 0.176 | 0.177 | 0.205 | **0.099** |
| **LC-PBEhLYP** | -0.348 | -0.348 | -0.302 | -0.302 | -0.406 | -0.305 | -0.321 | -0.318 | -0.307 | -0.322 | -0.320 | -0.296 | -0.500 | -0.319 | -0.361 | -0.307 | -0.318 | -0.301 | -0.302 | -0.301 | **-0.330** |
| **LC-PBEhP86** | -0.176 | -0.177 | 0.434 | 0.433 | -0.243 | 0.177 | 0.078 | 0.036 | 0.194 | 0.075 | 0.035 | 0.189 | -0.275 | 0.175 | -0.090 | 0.014 | 0.177 | 0.265 | 0.412 | 0.403 | **0.107** |
| **LC-PBEhPBE** | 0.147 | 0.133 | 0.568 | 0.569 | 0.024 | 0.490 | 0.457 | 0.376 | 0.507 | 0.456 | 0.374 | 0.488 | -0.186 | 0.462 | 0.108 | 0.468 | 0.343 | 0.566 | 0.587 | 0.607 | **0.377** |

| | | | | | | | | | | | | | | | | | | | | | |
|---|---|---|---|---|---|---|---|---|---|---|---|---|---|---|---|---|---|---|---|---|---|
| **LC-PBEhPKZB** | 0.152 | 0.140 | 0.565 | 0.563 | 0.035 | 0.489 | 0.459 | 0.382 | 0.490 | 0.457 | 0.379 | 0.490 | -0.158 | 0.469 | 0.136 | 0.476 | 0.347 | 0.565 | 0.572 | 0.594 | **0.380** |
| **LC-PBEhPL** | 0.207 | 0.203 | 0.505 | 0.505 | 0.077 | 0.478 | 0.397 | 0.360 | 0.478 | 0.393 | 0.357 | 0.456 | -0.113 | 0.458 | 0.172 | 0.477 | 0.328 | 0.532 | 0.523 | 0.547 | **0.367** |
| **LC-PBEhPW91** | 0.120 | 0.111 | 0.535 | 0.536 | -0.012 | 0.476 | 0.415 | 0.325 | 0.479 | 0.385 | 0.334 | 0.478 | -0.208 | 0.420 | 0.071 | 0.402 | 0.330 | 0.534 | 0.548 | 0.582 | **0.343** |
| **LC-PBEhRevTPSS** | 0.091 | 0.086 | 0.518 | 0.519 | -0.042 | 0.438 | 0.301 | 0.238 | 0.436 | 0.305 | 0.248 | 0.438 | -0.215 | 0.373 | 0.042 | 0.380 | 0.297 | 0.466 | 0.509 | 0.552 | **0.299** |
| **LC-PBEhTPSS** | 0.151 | 0.136 | 0.571 | 0.573 | 0.029 | 0.491 | 0.459 | 0.382 | 0.489 | 0.458 | 0.383 | 0.484 | -0.175 | 0.440 | 0.127 | 0.474 | 0.346 | 0.565 | 0.568 | 0.611 | **0.378** |
| **LC-PBEhVP86** | -0.173 | -0.175 | 0.446 | 0.443 | -0.239 | 0.181 | 0.081 | 0.038 | 0.177 | 0.079 | 0.038 | 0.192 | -0.274 | 0.202 | -0.087 | 0.014 | 0.186 | 0.242 | 0.375 | 0.443 | **0.109** |
| **LC-PBEhVWN** | 0.161 | 0.149 | 0.387 | 0.387 | 0.017 | 0.353 | 0.293 | 0.268 | 0.352 | 0.293 | 0.268 | 0.354 | -0.141 | 0.356 | 0.119 | 0.338 | 0.262 | 0.401 | 0.383 | 0.417 | **0.271** |
| **LC-PBEhVWN5** | 0.210 | 0.206 | 0.510 | 0.510 | 0.082 | 0.489 | 0.413 | 0.368 | 0.488 | 0.405 | 0.365 | 0.467 | -0.109 | 0.460 | 0.175 | 0.502 | 0.332 | 0.551 | 0.530 | 0.563 | **0.376** |
| **LC-PKZBB95** | 0.007 | 0.007 | 0.031 | 0.031 | -0.125 | 0.019 | 0.017 | 0.016 | 0.019 | 0.017 | 0.016 | 0.020 | -0.210 | 0.032 | -0.067 | 0.019 | -0.001 | 0.030 | 0.032 | 0.034 | **-0.003** |
| **LC-PKZBBRC** | 0.071 | 0.066 | 0.371 | 0.371 | -0.049 | 0.355 | 0.288 | 0.239 | 0.354 | 0.276 | 0.238 | 0.356 | -0.253 | 0.331 | 0.039 | 0.288 | 0.221 | 0.387 | 0.372 | 0.443 | **0.238** |
| **LC-PKZBKCIS** | 0.027 | 0.019 | 0.194 | 0.194 | -0.098 | 0.211 | 0.146 | 0.128 | 0.210 | 0.145 | 0.127 | 0.215 | -0.268 | 0.203 | -0.024 | 0.157 | 0.117 | 0.196 | 0.193 | 0.234 | **0.116** |
| **LC-PKZBLYP** | -0.334 | -0.334 | -0.286 | -0.285 | -0.395 | -0.291 | -0.305 | -0.304 | -0.292 | -0.307 | -0.305 | -0.282 | -0.489 | -0.308 | -0.350 | -0.291 | -0.304 | -0.286 | -0.287 | -0.286 | **-0.316** |
| **LC-PKZBP86** | -0.157 | -0.159 | 0.440 | 0.438 | -0.226 | 0.213 | 0.089 | 0.053 | 0.211 | 0.087 | 0.052 | 0.235 | -0.264 | 0.203 | -0.081 | 0.031 | 0.198 | 0.304 | 0.415 | 0.451 | **0.127** |
| **LC-PKZBPBE** | 0.153 | 0.143 | 0.561 | 0.562 | 0.053 | 0.492 | 0.464 | 0.388 | 0.488 | 0.464 | 0.389 | 0.498 | -0.170 | 0.452 | 0.132 | 0.491 | 0.348 | 0.566 | 0.580 | 0.601 | **0.383** |
| **LC-PKZBPKZB** | 0.165 | 0.159 | 0.562 | 0.564 | 0.056 | 0.496 | 0.465 | 0.395 | 0.492 | 0.464 | 0.395 | 0.498 | -0.144 | 0.481 | 0.160 | 0.494 | 0.352 | 0.568 | 0.582 | 0.599 | **0.390** |
| **LC-PKZBPL** | 0.214 | 0.210 | 0.510 | 0.510 | 0.089 | 0.475 | 0.422 | 0.377 | 0.474 | 0.426 | 0.377 | 0.471 | -0.101 | 0.469 | 0.177 | 0.497 | 0.339 | 0.538 | 0.529 | 0.567 | **0.379** |
| **LC-PKZBPW91** | 0.132 | 0.122 | 0.556 | 0.557 | 0.015 | 0.492 | 0.451 | 0.371 | 0.478 | 0.443 | 0.368 | 0.490 | -0.190 | 0.425 | 0.087 | 0.479 | 0.336 | 0.561 | 0.569 | 0.592 | **0.367** |
| **LC-PKZBRevTPSS** | 0.101 | 0.096 | 0.519 | 0.518 | -0.033 | 0.452 | 0.388 | 0.281 | 0.450 | 0.359 | 0.277 | 0.449 | -0.199 | 0.413 | 0.053 | 0.390 | 0.308 | 0.495 | 0.531 | 0.549 | **0.320** |
| **LC-PKZBTPSS** | 0.168 | 0.162 | 0.580 | 0.566 | 0.054 | 0.501 | 0.466 | 0.394 | 0.498 | 0.465 | 0.394 | 0.532 | -0.147 | 0.483 | 0.155 | 0.494 | 0.350 | 0.566 | 0.574 | 0.630 | **0.394** |
| **LC-PKZBVP86** | -0.155 | -0.156 | 0.450 | 0.449 | -0.222 | 0.210 | 0.106 | 0.057 | 0.211 | 0.105 | 0.075 | 0.215 | -0.263 | 0.265 | -0.072 | 0.033 | 0.203 | 0.306 | 0.426 | 0.454 | **0.135** |
| **LC-PKZBVWN** | 0.169 | 0.158 | 0.421 | 0.421 | 0.024 | 0.376 | 0.311 | 0.278 | 0.374 | 0.309 | 0.278 | 0.374 | -0.130 | 0.371 | 0.127 | 0.361 | 0.266 | 0.408 | 0.409 | 0.421 | **0.286** |
| **LC-PKZBVWN5** | 0.218 | 0.213 | 0.529 | 0.530 | 0.091 | 0.484 | 0.430 | 0.379 | 0.478 | 0.430 | 0.379 | 0.482 | -0.098 | 0.474 | 0.180 | 0.504 | 0.346 | 0.556 | 0.534 | 0.566 | **0.385** |
| **LC-PW91B95** | -0.015 | -0.015 | 0.031 | 0.031 | -0.127 | 0.018 | 0.016 | 0.016 | 0.018 | 0.016 | 0.016 | 0.019 | -0.213 | 0.005 | -0.067 | 0.019 | -0.001 | 0.029 | 0.031 | 0.033 | **-0.007** |
| **LC-PW91BRC** | 0.060 | 0.055 | 0.359 | 0.359 | -0.059 | 0.317 | 0.246 | 0.221 | 0.316 | 0.247 | 0.219 | 0.319 | -0.265 | 0.308 | 0.028 | 0.269 | 0.212 | 0.375 | 0.357 | 0.434 | **0.219** |
| **LC-PW91KCIS** | 0.017 | 0.010 | 0.183 | 0.183 | -0.105 | 0.195 | 0.128 | 0.109 | 0.194 | 0.128 | 0.108 | 0.187 | -0.281 | 0.154 | -0.035 | 0.124 | 0.101 | 0.181 | 0.179 | 0.202 | **0.098** |
| **LC-PW91LYP** | -0.349 | -0.349 | -0.304 | -0.304 | -0.407 | -0.307 | -0.322 | -0.320 | -0.309 | -0.324 | -0.322 | -0.298 | -0.500 | -0.321 | -0.363 | -0.309 | -0.319 | -0.302 | -0.304 | -0.303 | **-0.332** |
| **LC-PW91P86** | -0.174 | -0.177 | 0.433 | 0.434 | -0.239 | 0.187 | 0.078 | 0.041 | 0.178 | 0.075 | 0.037 | 0.197 | -0.275 | 0.177 | -0.090 | 0.008 | 0.180 | 0.275 | 0.413 | 0.439 | **0.110** |
| **LC-PW91PBE** | 0.148 | 0.133 | 0.569 | 0.569 | 0.027 | 0.494 | 0.456 | 0.374 | 0.482 | 0.456 | 0.372 | 0.496 | -0.184 | 0.459 | 0.103 | 0.451 | 0.344 | 0.563 | 0.570 | 0.588 | **0.374** |
| **LC-PW91PKZB** | 0.146 | 0.138 | 0.570 | 0.570 | 0.036 | 0.488 | 0.457 | 0.379 | 0.481 | 0.457 | 0.375 | 0.483 | -0.171 | 0.445 | 0.132 | 0.474 | 0.347 | 0.565 | 0.571 | 0.607 | **0.378** |
| **LC-PW91PL** | 0.207 | 0.203 | 0.494 | 0.494 | 0.076 | 0.455 | 0.387 | 0.352 | 0.455 | 0.387 | 0.349 | 0.454 | -0.111 | 0.456 | 0.172 | 0.478 | 0.328 | 0.513 | 0.505 | 0.556 | **0.360** |
| **LC-PW91PW91** | 0.119 | 0.112 | 0.544 | 0.543 | -0.012 | 0.483 | 0.390 | 0.338 | 0.469 | 0.359 | 0.311 | 0.485 | -0.206 | 0.419 | 0.080 | 0.400 | 0.330 | 0.536 | 0.557 | 0.580 | **0.342** |
| **LC-PW91RevTPSS** | 0.092 | 0.086 | 0.517 | 0.510 | -0.043 | 0.434 | 0.302 | 0.239 | 0.466 | 0.307 | 0.237 | 0.434 | -0.214 | 0.394 | 0.040 | 0.349 | 0.302 | 0.464 | 0.511 | 0.542 | **0.298** |
| **LC-PW91TPSS** | 0.140 | 0.137 | 0.570 | 0.571 | 0.030 | 0.490 | 0.459 | 0.379 | 0.479 | 0.457 | 0.379 | 0.489 | -0.174 | 0.440 | 0.120 | 0.489 | 0.344 | 0.563 | 0.572 | 0.606 | **0.377** |
| **LC-PW91VP86** | -0.173 | -0.174 | 0.439 | 0.438 | -0.237 | 0.181 | 0.082 | 0.039 | 0.176 | 0.081 | 0.038 | 0.194 | -0.274 | 0.188 | -0.088 | 0.014 | 0.187 | 0.278 | 0.414 | 0.439 | **0.112** |
| **LC-PW91VWN** | 0.161 | 0.148 | 0.382 | 0.382 | 0.016 | 0.352 | 0.293 | 0.266 | 0.351 | 0.292 | 0.266 | 0.353 | -0.141 | 0.355 | 0.117 | 0.334 | 0.262 | 0.393 | 0.380 | 0.411 | **0.269** |
| **LC-PW91VWN5** | 0.210 | 0.205 | 0.507 | 0.507 | 0.082 | 0.469 | 0.400 | 0.358 | 0.467 | 0.399 | 0.358 | 0.463 | -0.109 | 0.459 | 0.175 | 0.496 | 0.330 | 0.549 | 0.525 | 0.576 | **0.371** |
| **LC-RevTPSSB95** | 0.010 | 0.010 | 0.033 | 0.033 | -0.122 | 0.021 | 0.019 | 0.019 | 0.021 | 0.019 | 0.019 | 0.022 | -0.209 | 0.007 | -0.064 | 0.022 | 0.016 | 0.032 | 0.034 | 0.036 | **-0.001** |
| **LC-RevTPSSBRC** | 0.074 | 0.070 | 0.390 | 0.390 | -0.046 | 0.343 | 0.252 | 0.226 | 0.342 | 0.251 | 0.225 | 0.344 | -0.251 | 0.306 | 0.041 | 0.282 | 0.225 | 0.384 | 0.387 | 0.434 | **0.233** |
| **LC-RevTPSSKCIS** | 0.029 | 0.020 | 0.193 | 0.193 | -0.098 | 0.204 | 0.137 | 0.118 | 0.202 | 0.137 | 0.117 | 0.192 | -0.265 | 0.198 | -0.023 | 0.152 | 0.121 | 0.193 | 0.191 | 0.232 | **0.112** |
| **LC-RevTPSSLYP** | -0.341 | -0.342 | -0.298 | -0.297 | -0.401 | -0.303 | -0.318 | -0.317 | -0.305 | -0.320 | -0.318 | -0.294 | -0.492 | -0.320 | -0.358 | -0.304 | -0.313 | -0.298 | -0.299 | -0.298 | **-0.327** |
| **LC-RevTPSSP86** | -0.151 | -0.157 | 0.444 | 0.441 | -0.222 | 0.193 | 0.090 | 0.051 | 0.194 | 0.093 | 0.048 | 0.212 | -0.264 | 0.234 | -0.074 | 0.053 | 0.199 | 0.262 | 0.436 | 0.462 | **0.127** |
| **LC-RevTPSSPBE** | 0.163 | 0.153 | 0.544 | 0.544 | 0.055 | 0.531 | 0.459 | 0.380 | 0.512 | 0.457 | 0.374 | 0.514 | -0.153 | 0.427 | 0.138 | 0.480 | 0.377 | 0.566 | 0.576 | 0.572 | **0.383** |
| **LC-RevTPSSPKZB** | 0.184 | 0.178 | 0.547 | 0.546 | 0.058 | 0.518 | 0.462 | 0.384 | 0.517 | 0.461 | 0.380 | 0.540 | -0.141 | 0.439 | 0.147 | 0.498 | 0.378 | 0.565 | 0.578 | 0.573 | **0.391** |
| **LC-RevTPSSPL** | 0.218 | 0.214 | 0.499 | 0.499 | 0.093 | 0.459 | 0.397 | 0.358 | 0.458 | 0.394 | 0.358 | 0.461 | -0.093 | 0.465 | 0.178 | 0.490 | 0.345 | 0.510 | 0.520 | 0.546 | **0.369** |
| **LC-RevTPSSPW91** | 0.142 | 0.139 | 0.564 | 0.563 | 0.023 | 0.476 | 0.393 | 0.342 | 0.476 | 0.385 | 0.320 | 0.476 | -0.187 | 0.425 | 0.092 | 0.430 | 0.342 | 0.542 | 0.570 | 0.587 | **0.355** |
| **LC-RevTPSSRevTPSS** | 0.107 | 0.102 | 0.492 | 0.493 | -0.017 | 0.444 | 0.317 | 0.255 | 0.417 | 0.317 | 0.254 | 0.446 | -0.196 | 0.391 | 0.058 | 0.391 | 0.310 | 0.494 | 0.521 | 0.549 | **0.307** |
| **LC-RevTPSSTPSS** | 0.171 | 0.172 | 0.545 | 0.544 | 0.058 | 0.515 | 0.460 | 0.383 | 0.515 | 0.460 | 0.382 | 0.544 | -0.143 | 0.441 | 0.143 | 0.481 | 0.376 | 0.568 | 0.579 | 0.570 | **0.388** |
| **LC-RevTPSSVP86** | -0.146 | -0.154 | 0.397 | 0.439 | -0.220 | 0.220 | 0.087 | 0.053 | 0.214 | 0.086 | 0.051 | 0.219 | -0.262 | 0.318 | -0.071 | 0.054 | 0.202 | 0.265 | 0.435 | 0.460 | **0.132** |
| **LC-RevTPSSVWN** | 0.172 | 0.161 | 0.394 | 0.394 | 0.026 | 0.363 | 0.295 | 0.268 | 0.362 | 0.294 | 0.267 | 0.365 | -0.126 | 0.375 | 0.128 | 0.356 | 0.265 | 0.410 | 0.396 | 0.420 | **0.279** |
| **LC-RevTPSSVWN5** | 0.221 | 0.218 | 0.503 | 0.503 | 0.095 | 0.463 | 0.408 | 0.368 | 0.462 | 0.408 | 0.368 | 0.464 | -0.090 | 0.473 | 0.183 | 0.495 | 0.349 | 0.526 | 0.525 | 0.538 | **0.374** |
| **LC-SB95** | -0.007 | -0.018 | 0.028 | 0.028 | -0.152 | 0.015 | 0.014 | 0.013 | 0.015 | 0.014 | 0.013 | 0.016 | -0.217 | 0.002 | -0.070 | 0.016 | -0.003 | 0.027 | 0.029 | 0.031 | **-0.010** |
| **LC-SBRC** | 0.061 | 0.056 | 0.379 | 0.379 | -0.058 | 0.343 | 0.252 | 0.227 | 0.338 | 0.248 | 0.226 | 0.363 | -0.264 | 0.314 | 0.027 | 0.261 | 0.215 | 0.381 | 0.372 | 0.439 | **0.228** |
| **LC-SKCIS** | 0.020 | 0.010 | 0.189 | 0.189 | -0.103 | 0.200 | 0.132 | 0.112 | 0.199 | 0.131 | 0.110 | 0.191 | -0.280 | 0.156 | -0.037 | 0.134 | 0.103 | 0.187 | 0.184 | 0.224 | **0.102** |
| **LC-SLYP** | -0.346 | -0.346 | -0.297 | -0.297 | -0.404 | -0.302 | -0.317 | -0.315 | -0.304 | -0.319 | -0.317 | -0.292 | -0.499 | -0.317 | -0.361 | -0.305 | -0.314 | -0.297 | -0.297 | -0.297 | **-0.327** |
| **LC-SP86** | -0.179 | -0.180 | 0.446 | 0.446 | -0.244 | 0.175 | 0.077 | 0.009 | 0.174 | 0.083 | 0.006 | 0.185 | -0.260 | 0.195 | -0.104 | 0.009 | 0.108 | 0.283 | 0.417 | 0.458 | **0.105** |
| **LC-SPBE** | 0.135 | 0.128 | 0.576 | 0.576 | 0.030 | 0.485 | 0.457 | 0.357 | 0.484 | 0.458 | 0.355 | 0.485 | -0.185 | 0.422 | 0.110 | 0.463 | 0.343 | 0.559 | 0.564 | 0.590 | **0.370** |
| **LC-SPKZB** | 0.140 | 0.134 | 0.603 | 0.603 | 0.052 | 0.488 | 0.460 | 0.374 | 0.488 | 0.461 | 0.371 | 0.484 | -0.173 | 0.429 | 0.139 | 0.481 | 0.346 | 0.561 | 0.562 | 0.593 | **0.380** |

| | | | | | | | | | | | | | | | | | | | | | |
|---|---|---|---|---|---|---|---|---|---|---|---|---|---|---|---|---|---|---|---|---|---|
| **LC-SPL** | 0.206 | 0.202 | 0.507 | 0.507 | 0.081 | 0.482 | 0.406 | 0.354 | 0.477 | 0.404 | 0.350 | 0.488 | -0.109 | 0.452 | 0.171 | 0.446 | 0.331 | 0.526 | 0.522 | 0.543 | **0.367** |
| **LC-SPW91** | 0.116 | 0.108 | 0.571 | 0.571 | -0.020 | 0.475 | 0.408 | 0.311 | 0.472 | 0.389 | 0.310 | 0.474 | -0.206 | 0.415 | 0.063 | 0.412 | 0.331 | 0.571 | 0.550 | 0.580 | **0.345** |
| **LC-SRevTPSS** | 0.086 | 0.080 | 0.483 | 0.483 | -0.042 | 0.474 | 0.307 | 0.236 | 0.471 | 0.300 | 0.278 | 0.456 | -0.214 | 0.359 | 0.034 | 0.313 | 0.305 | 0.467 | 0.475 | 0.552 | **0.295** |
| **LC-STPSS** | 0.138 | 0.132 | 0.581 | 0.581 | 0.033 | 0.480 | 0.458 | 0.374 | 0.479 | 0.459 | 0.371 | 0.487 | -0.175 | 0.421 | 0.127 | 0.463 | 0.344 | 0.556 | 0.562 | 0.582 | **0.373** |
| **LC-SVP86** | -0.176 | -0.177 | 0.447 | 0.447 | -0.240 | 0.178 | 0.082 | 0.007 | 0.183 | 0.079 | 0.011 | 0.199 | -0.313 | 0.175 | -0.099 | 0.011 | 0.071 | 0.289 | 0.417 | 0.468 | **0.103** |
| **LC-SVWN5** | 0.208 | 0.205 | 0.524 | 0.524 | 0.085 | 0.484 | 0.412 | 0.357 | 0.482 | 0.409 | 0.356 | 0.491 | -0.106 | 0.455 | 0.174 | 0.451 | 0.345 | 0.546 | 0.526 | 0.586 | **0.376** |
| **LC-TPSSB95** | 0.009 | 0.009 | 0.033 | 0.033 | -0.123 | 0.021 | 0.019 | 0.018 | 0.021 | 0.019 | 0.018 | 0.021 | -0.193 | 0.007 | -0.064 | 0.021 | 0.002 | 0.031 | 0.033 | 0.035 | **-0.001** |
| **LC-TPSSBRC** | 0.069 | 0.065 | 0.390 | 0.390 | -0.050 | 0.318 | 0.246 | 0.222 | 0.327 | 0.245 | 0.221 | 0.329 | -0.256 | 0.305 | 0.037 | 0.275 | 0.221 | 0.383 | 0.372 | 0.433 | **0.227** |
| **LC-TPSSKCIS** | 0.024 | 0.017 | 0.190 | 0.190 | -0.100 | 0.198 | 0.131 | 0.112 | 0.196 | 0.131 | 0.111 | 0.187 | -0.270 | 0.195 | -0.027 | 0.146 | 0.117 | 0.186 | 0.187 | 0.213 | **0.107** |
| **LC-TPSSLYP** | -0.346 | -0.346 | -0.303 | -0.303 | -0.405 | -0.308 | -0.323 | -0.320 | -0.309 | -0.324 | -0.322 | -0.299 | -0.496 | -0.323 | -0.361 | -0.308 | -0.318 | -0.302 | -0.305 | -0.303 | **-0.331** |
| **LC-TPSSP86** | -0.158 | -0.163 | 0.442 | 0.439 | -0.227 | 0.188 | 0.083 | 0.043 | 0.184 | 0.097 | 0.040 | 0.192 | -0.267 | 0.188 | -0.079 | 0.024 | 0.194 | 0.294 | 0.428 | 0.455 | **0.120** |
| **LC-TPSSPBE** | 0.159 | 0.151 | 0.542 | 0.541 | 0.040 | 0.484 | 0.454 | 0.373 | 0.484 | 0.452 | 0.370 | 0.510 | -0.171 | 0.421 | 0.131 | 0.480 | 0.352 | 0.532 | 0.575 | 0.597 | **0.374** |
| **LC-TPSSPKZB** | 0.165 | 0.160 | 0.544 | 0.544 | 0.056 | 0.486 | 0.458 | 0.378 | 0.508 | 0.457 | 0.375 | 0.481 | -0.146 | 0.427 | 0.142 | 0.485 | 0.376 | 0.532 | 0.577 | 0.595 | **0.380** |
| **LC-TPSSPL** | 0.215 | 0.211 | 0.496 | 0.496 | 0.090 | 0.457 | 0.392 | 0.353 | 0.457 | 0.389 | 0.350 | 0.459 | -0.101 | 0.464 | 0.177 | 0.485 | 0.331 | 0.507 | 0.498 | 0.532 | **0.363** |
| **LC-TPSSPW91** | 0.141 | 0.122 | 0.564 | 0.563 | 0.018 | 0.474 | 0.374 | 0.329 | 0.481 | 0.379 | 0.327 | 0.475 | -0.192 | 0.404 | 0.089 | 0.421 | 0.339 | 0.523 | 0.568 | 0.585 | **0.349** |
| **LC-TPSSRevTPSS** | 0.103 | 0.098 | 0.488 | 0.486 | -0.031 | 0.448 | 0.304 | 0.242 | 0.474 | 0.304 | 0.218 | 0.444 | -0.201 | 0.382 | 0.052 | 0.391 | 0.307 | 0.498 | 0.516 | 0.549 | **0.304** |
| **LC-TPSSTPSS** | 0.158 | 0.152 | 0.542 | 0.542 | 0.044 | 0.487 | 0.459 | 0.378 | 0.505 | 0.458 | 0.375 | 0.506 | -0.148 | 0.429 | 0.140 | 0.475 | 0.376 | 0.531 | 0.576 | 0.570 | **0.378** |
| **LC-TPSSVP86** | -0.156 | -0.161 | 0.435 | 0.435 | -0.225 | 0.195 | 0.083 | 0.049 | 0.193 | 0.082 | 0.047 | 0.211 | -0.266 | 0.194 | -0.075 | 0.026 | 0.197 | 0.296 | 0.432 | 0.462 | **0.123** |
| **LC-TPSSVWN** | 0.168 | 0.157 | 0.385 | 0.385 | 0.024 | 0.351 | 0.292 | 0.263 | 0.350 | 0.291 | 0.262 | 0.357 | -0.131 | 0.369 | 0.124 | 0.344 | 0.263 | 0.399 | 0.386 | 0.415 | **0.273** |
| **LC-TPSSVWN5** | 0.218 | 0.215 | 0.499 | 0.499 | 0.092 | 0.460 | 0.401 | 0.356 | 0.458 | 0.400 | 0.356 | 0.463 | -0.095 | 0.469 | 0.181 | 0.489 | 0.342 | 0.523 | 0.503 | 0.575 | **0.370** |
| **LC-XaB95** | -0.007 | -0.007 | 0.029 | 0.029 | -0.145 | 0.015 | 0.014 | -0.006 | 0.015 | 0.014 | 0.013 | 0.016 | -0.218 | 0.026 | -0.069 | 0.017 | -0.003 | 0.027 | 0.029 | 0.030 | **-0.009** |
| **LC-XaBRC** | 0.059 | 0.053 | 0.368 | 0.368 | -0.061 | 0.310 | 0.235 | 0.216 | 0.307 | 0.233 | 0.215 | 0.313 | -0.267 | 0.294 | 0.024 | 0.256 | 0.212 | 0.382 | 0.368 | 0.432 | **0.216** |
| **LC-XaKCIS** | 0.015 | 0.007 | 0.181 | 0.181 | -0.106 | 0.171 | 0.120 | 0.100 | 0.170 | 0.119 | 0.099 | 0.177 | -0.282 | 0.143 | -0.039 | 0.114 | 0.097 | 0.175 | 0.177 | 0.199 | **0.091** |
| **LC-XaLYP** | -0.361 | -0.361 | -0.318 | -0.318 | -0.417 | -0.322 | -0.337 | -0.335 | -0.324 | -0.339 | -0.337 | -0.312 | -0.508 | -0.336 | -0.374 | -0.324 | -0.333 | -0.318 | -0.319 | -0.319 | **-0.346** |
| **LC-XaP86** | -0.182 | -0.184 | 0.440 | 0.440 | -0.248 | 0.171 | 0.074 | -0.007 | 0.170 | 0.052 | -0.001 | 0.179 | -0.281 | 0.188 | -0.095 | 0.000 | 0.181 | 0.284 | 0.387 | 0.442 | **0.101** |
| **LC-XaPBE** | 0.135 | 0.129 | 0.567 | 0.568 | 0.029 | 0.480 | 0.404 | 0.339 | 0.500 | 0.401 | 0.340 | 0.478 | -0.184 | 0.415 | 0.106 | 0.455 | 0.344 | 0.560 | 0.553 | 0.585 | **0.360** |
| **LC-XaPKZB** | 0.141 | 0.135 | 0.569 | 0.569 | 0.035 | 0.478 | 0.455 | 0.345 | 0.478 | 0.440 | 0.345 | 0.478 | -0.171 | 0.422 | 0.132 | 0.460 | 0.346 | 0.563 | 0.554 | 0.587 | **0.368** |
| **LC-XaPL** | 0.204 | 0.200 | 0.489 | 0.489 | 0.070 | 0.464 | 0.381 | 0.335 | 0.463 | 0.380 | 0.334 | 0.461 | -0.113 | 0.445 | 0.172 | 0.439 | 0.328 | 0.501 | 0.497 | 0.518 | **0.353** |
| **LC-XaPW91** | 0.118 | 0.111 | 0.546 | 0.543 | -0.022 | 0.471 | 0.342 | 0.319 | 0.471 | 0.354 | 0.289 | 0.471 | -0.206 | 0.430 | 0.063 | 0.371 | 0.336 | 0.533 | 0.526 | 0.579 | **0.332** |
| **LC-XaRevTPSS** | 0.088 | 0.082 | 0.473 | 0.472 | -0.042 | 0.439 | 0.349 | 0.285 | 0.459 | 0.314 | 0.229 | 0.445 | -0.216 | 0.393 | 0.036 | 0.309 | 0.308 | 0.470 | 0.467 | 0.549 | **0.295** |
| **LC-XaTPSS** | 0.139 | 0.133 | 0.571 | 0.571 | 0.035 | 0.481 | 0.455 | 0.353 | 0.479 | 0.456 | 0.354 | 0.481 | -0.174 | 0.426 | 0.138 | 0.460 | 0.345 | 0.562 | 0.549 | 0.587 | **0.370** |
| **BB1K** | 0.170 | 0.170 | 0.174 | 0.174 | 0.140 | 0.231 | 0.229 | 0.229 | 0.312 | 0.229 | 0.229 | 0.232 | 0.145 | 0.436 | 0.169 | 0.237 | 0.173 | 0.174 | 0.174 | 0.174 | **0.210** |
| **mPW1K** | 0.388 | 0.379 | 0.521 | 0.521 | 0.241 | 0.494 | 0.441 | 0.386 | 0.493 | 0.440 | 0.385 | 0.490 | 0.067 | 0.418 | 0.307 | 0.533 | 0.450 | 0.549 | 0.532 | 0.540 | **0.429** |
| **mPW1B95** | 0.075 | 0.074 | 0.114 | 0.114 | -0.012 | 0.118 | 0.102 | 0.101 | 0.118 | 0.102 | 0.101 | 0.119 | -0.120 | 0.105 | 0.069 | 0.107 | 0.111 | 0.131 | 0.114 | 0.115 | **0.088** |
| **mPWB1K** | 0.071 | 0.071 | 0.086 | 0.086 | -0.040 | 0.080 | 0.075 | 0.074 | 0.080 | 0.074 | 0.073 | 0.104 | -0.104 | 0.054 | 0.043 | 0.074 | 0.083 | 0.085 | 0.086 | 0.086 | **0.062** |
| **mPW1KCIS** | 0.249 | 0.243 | 0.401 | 0.401 | 0.113 | 0.373 | 0.347 | 0.326 | 0.373 | 0.346 | 0.326 | 0.377 | -0.026 | 0.360 | 0.183 | 0.412 | 0.301 | 0.424 | 0.398 | 0.419 | **0.317** |
| **mPWKCIS1K** | 0.241 | 0.236 | 0.367 | 0.367 | 0.112 | 0.320 | 0.281 | 0.261 | 0.318 | 0.280 | 0.260 | 0.324 | -0.026 | 0.313 | 0.182 | 0.357 | 0.293 | 0.386 | 0.367 | 0.374 | **0.281** |
| **TPSS1KCIS** | 0.107 | 0.104 | 0.174 | 0.174 | -0.001 | 0.172 | 0.143 | 0.133 | 0.172 | 0.142 | 0.132 | 0.178 | -0.111 | 0.180 | 0.058 | 0.192 | 0.135 | 0.190 | 0.173 | 0.174 | **0.131** |
| **PBE1KCIS** | -0.044 | -0.047 | 0.036 | 0.036 | -0.129 | 0.029 | 0.008 | 0.001 | 0.027 | 0.006 | 0.000 | 0.038 | -0.238 | 0.029 | -0.082 | 0.030 | 0.000 | 0.035 | 0.031 | 0.034 | **-0.010** |
| **mPWLYP1M** | -0.114 | -0.116 | -0.028 | -0.028 | -0.195 | -0.027 | -0.053 | -0.056 | -0.029 | -0.054 | -0.058 | -0.012 | -0.330 | -0.018 | -0.146 | -0.016 | -0.070 | -0.023 | -0.029 | -0.024 | **-0.071** |
| **PBE1W** | -0.012 | -0.016 | 0.070 | 0.071 | -0.104 | 0.073 | 0.044 | 0.030 | 0.071 | 0.043 | 0.027 | 0.085 | -0.211 | 0.084 | -0.055 | 0.077 | 0.017 | 0.074 | 0.068 | 0.069 | **0.025** |
| **mPWLYP1W** | -0.053 | -0.056 | 0.048 | 0.048 | -0.140 | 0.049 | 0.027 | 0.018 | 0.048 | 0.025 | 0.017 | 0.070 | -0.292 | 0.055 | -0.089 | 0.068 | -0.002 | 0.055 | 0.047 | 0.054 | **0.000** |
| **PBELYP1W** | -0.169 | -0.171 | -0.114 | -0.114 | -0.238 | -0.111 | -0.129 | -0.131 | -0.112 | -0.130 | -0.133 | -0.098 | -0.336 | -0.094 | -0.196 | -0.106 | -0.142 | -0.114 | -0.114 | -0.114 | **-0.143** |
| **TPSSLYP1W** | -0.094 | -0.095 | -0.040 | -0.041 | -0.177 | -0.043 | -0.063 | -0.065 | -0.045 | -0.065 | -0.067 | -0.030 | -0.279 | -0.018 | -0.129 | -0.023 | -0.071 | -0.035 | -0.040 | -0.042 | **-0.073** |
| **mPW3LYP** | -0.131 | -0.132 | -0.062 | -0.061 | -0.207 | -0.072 | -0.096 | -0.098 | -0.073 | -0.098 | -0.100 | -0.059 | -0.326 | -0.067 | -0.160 | -0.064 | -0.092 | -0.061 | -0.063 | -0.060 | **-0.104** |
| **PBEPBE-D2** | -0.208 | -0.211 | -0.158 | -0.158 | -0.261 | -0.147 | -0.167 | -0.169 | -0.148 | -0.169 | -0.171 | -0.132 | -0.342 | -0.150 | -0.229 | -0.157 | -0.182 | -0.161 | -0.160 | -0.158 | **-0.182** |
| **BLYP-D2** | 0.041 | -0.078 | 0.248 | 0.248 | -0.147 | 0.351 | 0.266 | 0.261 | 0.349 | 0.302 | 0.260 | 0.364 | -0.268 | 0.248 | 0.037 | 0.359 | 0.132 | 0.339 | 0.246 | 0.333 | **0.194** |
| **B3LYP-D2** | -0.170 | -0.175 | -0.089 | -0.090 | -0.224 | -0.085 | -0.109 | -0.108 | -0.086 | -0.112 | -0.109 | -0.075 | -0.323 | -0.087 | -0.171 | -0.088 | -0.113 | -0.095 | -0.092 | -0.093 | **-0.125** |
| **BP86-D2** | 0.988 | 0.990 | 1.385 | 1.414 | 0.570 | 1.149 | 1.105 | 1.033 | 1.145 | 1.096 | 1.026 | 1.283 | 0.367 | 1.201 | 0.682 | 1.295 | 1.036 | 1.316 | 1.288 | 1.403 | **1.089** |
| **TPSSTPSS-D2** | -0.109 | -0.112 | -0.044 | -0.045 | -0.171 | -0.054 | -0.077 | -0.077 | -0.056 | -0.079 | -0.079 | -0.040 | -0.255 | -0.052 | -0.132 | -0.039 | -0.090 | -0.043 | -0.061 | -0.050 | **-0.083** |
| **PBEPBE-D3** | -0.115 | -0.118 | -0.050 | -0.050 | -0.188 | -0.046 | -0.068 | -0.076 | -0.047 | -0.070 | -0.077 | -0.035 | -0.284 | -0.043 | -0.148 | -0.050 | -0.090 | -0.050 | -0.053 | -0.051 | **-0.086** |
| **BLYP-D3** | -0.067 | -0.090 | 0.176 | 0.174 | -0.169 | 0.368 | 0.148 | 0.163 | 0.367 | 0.215 | 0.170 | 0.379 | -0.285 | 0.203 | -0.100 | 0.256 | -0.017 | 0.211 | 0.176 | 0.203 | **0.124** |
| **B3LYP-D3** | -0.099 | -0.104 | -0.011 | -0.012 | -0.193 | -0.003 | -0.036 | -0.041 | -0.004 | -0.037 | -0.042 | 0.007 | -0.300 | 0.009 | -0.138 | -0.022 | -0.052 | -0.009 | -0.012 | -0.013 | **-0.056** |
| **BP86-D3** | 0.841 | 0.840 | 1.182 | 1.182 | 0.461 | 0.964 | 0.943 | 0.872 | 0.968 | 0.943 | 0.872 | 0.979 | 0.302 | 1.134 | 0.586 | 1.094 | 0.838 | 1.193 | 1.126 | 1.245 | **0.928** |
| **TPSSTPSS-D3** | -0.021 | -0.025 | 0.035 | 0.035 | -0.115 | 0.020 | 0.005 | -0.008 | 0.019 | 0.004 | -0.009 | 0.027 | -0.181 | 0.043 | -0.066 | 0.037 | -0.010 | 0.037 | 0.030 | 0.033 | **-0.006** |
| **PBE1PBE-D3** | -0.094 | -0.097 | -0.019 | -0.019 | -0.173 | -0.034 | -0.062 | -0.072 | -0.036 | -0.063 | -0.074 | -0.023 | -0.272 | -0.038 | -0.131 | -0.033 | -0.067 | -0.024 | -0.029 | -0.022 | **-0.069** |
| **BPBE-D3** | 0.626 | 0.636 | 1.076 | 1.073 | 0.369 | 0.876 | 0.831 | 0.743 | 0.859 | 0.814 | 0.718 | 0.875 | 0.254 | 0.883 | 0.518 | 1.054 | 0.736 | 1.171 | 0.957 | 1.173 | **0.812** |

| | | | | | | | | | | | | | | | | | | | | | |
|---|---|---|---|---|---|---|---|---|---|---|---|---|---|---|---|---|---|---|---|---|---|
| **B3PW91-D3** | 0.480 | 0.479 | 0.688 | 0.662 | 0.253 | 0.615 | 0.535 | 0.478 | 0.613 | 0.535 | 0.477 | 0.592 | 0.133 | 0.562 | 0.383 | 0.731 | 0.514 | 0.718 | 0.653 | 0.759 | **0.543** |
| **BMK-D3** | 0.770 | 0.750 | 1.011 | 1.011 | 0.553 | 0.926 | 0.775 | 0.769 | 0.769 | 0.772 | 0.788 | 0.788 | 0.329 | 0.848 | 0.641 | 0.858 | 0.867 | 0.924 | 0.962 | 0.969 | **0.804** |
| **CAM-B3LYP-D3** | -0.259 | -0.260 | -0.193 | -0.193 | -0.320 | -0.203 | -0.222 | -0.220 | -0.205 | -0.224 | -0.222 | -0.190 | -0.418 | -0.210 | -0.275 | -0.200 | -0.219 | -0.196 | -0.196 | -0.193 | **-0.231** |
| **LC-wPBE-D3** | 0.006 | 0.003 | 0.157 | 0.157 | -0.113 | 0.162 | 0.125 | 0.112 | 0.161 | 0.124 | 0.108 | 0.167 | -0.242 | 0.131 | -0.039 | 0.140 | 0.084 | 0.173 | 0.155 | 0.166 | **0.087** |
| **M05-D3** | -0.235 | -0.235 | -0.216 | -0.216 | -0.318 | -0.226 | -0.235 | -0.231 | -0.227 | -0.237 | -0.232 | -0.219 | -0.404 | -0.280 | -0.273 | -0.200 | -0.221 | -0.195 | -0.205 | -0.199 | **-0.240** |
| **M052X-D3** | -0.112 | -0.113 | -0.079 | -0.079 | -0.194 | -0.110 | -0.123 | -0.122 | -0.112 | -0.124 | -0.123 | -0.096 | -0.277 | -0.105 | -0.152 | -0.103 | -0.080 | -0.078 | -0.080 | -0.080 | **-0.117** |
| **M06-D3** | 0.054 | 0.054 | 0.171 | 0.171 | 0.015 | 0.125 | 0.062 | 0.060 | 0.125 | 0.061 | 0.060 | 0.126 | -0.030 | 0.043 | 0.040 | 0.116 | 0.162 | 0.168 | 0.170 | 0.117 | **0.093** |
| **M06L-D3** | 0.107 | 0.107 | 0.133 | 0.133 | 0.050 | 0.105 | 0.078 | 0.075 | 0.105 | 0.078 | 0.051 | 0.107 | -0.052 | 0.069 | 0.090 | 0.050 | 0.132 | 0.090 | 0.104 | 0.116 | **0.086** |
| **M06HF-D3** | 0.115 | 0.115 | 0.128 | 0.128 | 0.076 | 0.128 | 0.124 | 0.121 | 0.128 | 0.124 | 0.121 | 0.128 | -0.024 | 0.095 | 0.108 | 0.126 | 0.125 | 0.193 | 0.126 | 0.120 | **0.115** |
| **M062X-D3** | 0.059 | 0.058 | 0.132 | 0.132 | -0.043 | 0.093 | 0.074 | 0.080 | 0.092 | 0.083 | 0.079 | 0.096 | -0.254 | -0.025 | 0.015 | 0.098 | 0.105 | 0.142 | 0.132 | 0.139 | **0.064** |
| **PBEPBE-D3BJ** | -0.120 | -0.124 | -0.047 | -0.047 | -0.194 | -0.044 | -0.070 | -0.077 | -0.045 | -0.072 | -0.079 | -0.033 | -0.292 | -0.045 | -0.152 | -0.045 | -0.094 | -0.048 | -0.055 | -0.049 | **-0.087** |
| **BLYP-D3BJ** | 0.141 | 0.115 | 0.403 | 0.404 | 0.004 | 0.430 | 0.336 | 0.313 | 0.464 | 0.338 | 0.312 | 0.478 | -0.127 | 0.358 | 0.122 | 0.528 | 0.208 | 0.561 | 0.393 | 0.540 | **0.316** |
| **B3LYP-D3BJ** | -0.033 | -0.043 | 0.082 | 0.083 | -0.143 | 0.165 | 0.106 | 0.085 | 0.163 | 0.121 | 0.089 | 0.172 | -0.256 | 0.131 | -0.084 | 0.151 | 0.012 | 0.077 | 0.073 | 0.164 | **0.056** |
| **BP86-D3BJ** | 0.893 | 0.893 | 1.153 | 1.152 | 0.588 | 0.982 | 0.944 | 0.902 | 0.958 | 0.936 | 0.898 | 1.002 | 0.536 | 1.003 | 0.788 | 1.103 | 0.933 | 1.130 | 1.163 | 1.207 | **0.958** |
| **TPSSTPSS-D3BJ** | 0.029 | 0.026 | 0.069 | 0.069 | -0.089 | 0.072 | 0.055 | 0.045 | 0.071 | 0.054 | 0.040 | 0.077 | -0.147 | 0.093 | -0.038 | 0.071 | 0.036 | 0.071 | 0.067 | 0.067 | **0.037** |
| **PBE1PBE-D3BJ** | -0.067 | -0.071 | 0.002 | 0.002 | -0.150 | -0.006 | -0.036 | -0.048 | -0.008 | -0.037 | -0.049 | 0.002 | -0.244 | -0.006 | -0.107 | -0.008 | -0.042 | 0.001 | -0.001 | 0.000 | **-0.044** |
| **BPBE-D3BJ** | 0.762 | 0.762 | 0.987 | 0.987 | 0.502 | 0.880 | 0.859 | 0.799 | 0.880 | 0.860 | 0.797 | 0.888 | 0.435 | 0.877 | 0.654 | 1.036 | 0.875 | 1.120 | 0.978 | 1.110 | **0.852** |
| **B3PW91-D3BJ** | 0.543 | 0.541 | 0.788 | 0.788 | 0.345 | 0.610 | 0.609 | 0.564 | 0.639 | 0.609 | 0.563 | 0.646 | 0.297 | 0.666 | 0.452 | 0.799 | 0.568 | 0.828 | 0.815 | 0.868 | **0.627** |
| **BMK-D3BJ** | 0.867 | 0.868 | 1.186 | 1.193 | 0.827 | 0.883 | 0.880 | 0.850 | 0.885 | 0.876 | 0.849 | 1.029 | 0.482 | 0.804 | 0.791 | 0.995 | 1.103 | 1.023 | 1.045 | 1.107 | **0.927** |
| **CAM-B3LYP-D3BJ** | -0.173 | -0.174 | -0.096 | -0.096 | -0.253 | -0.108 | -0.130 | -0.131 | -0.109 | -0.132 | -0.133 | -0.098 | -0.367 | -0.113 | -0.201 | -0.104 | -0.130 | -0.096 | -0.098 | -0.094 | **-0.142** |
| **LC-wPBE-D3BJ** | 0.070 | 0.064 | 0.220 | 0.220 | -0.054 | 0.230 | 0.181 | 0.172 | 0.230 | 0.190 | 0.177 | 0.225 | -0.180 | 0.227 | 0.022 | 0.212 | 0.128 | 0.234 | 0.218 | 0.262 | **0.152** |
| **B2PLYP** | 0.209 | 0.190 | 0.472 | 0.471 | 0.401 | 0.599 | 0.387 | 0.264 | 0.623 | 0.441 | 0.258 | 1.082 | -0.092 | 0.256 | 0.175 | 0.280 | 0.287 | 0.214 | 0.338 | 0.199 | **0.353** |
| **B2PLYP(Full)** | 0.190 | 0.173 | 0.449 | 0.449 | 0.392 | 0.747 | 0.373 | 0.216 | 0.725 | 0.337 | 0.208 | 0.998 | -0.094 | 0.241 | 0.159 | 0.141 | 0.275 | 0.175 | 0.283 | 0.122 | **0.328** |
| **B2PLYPD** | -0.089 | -0.099 | -0.044 | -0.045 | -0.145 | -0.019 | -0.068 | -0.081 | -0.040 | -0.076 | -0.089 | 0.096 | -0.253 | -0.056 | -0.110 | -0.089 | -0.067 | -0.099 | -0.063 | -0.095 | **-0.077** |
| **B2PLYPD(Full)** | -0.096 | -0.107 | -0.048 | -0.049 | -0.146 | -0.022 | -0.073 | -0.093 | -0.045 | -0.081 | -0.104 | 0.093 | -0.255 | -0.060 | -0.120 | -0.112 | -0.075 | -0.105 | -0.072 | -0.116 | **-0.084** |
| **B2PLYP-D3** | -0.055 | -0.065 | 0.026 | 0.026 | -0.122 | 0.056 | -0.029 | -0.051 | 0.048 | -0.039 | -0.060 | 0.172 | -0.251 | -0.011 | -0.079 | -0.049 | -0.027 | -0.048 | -0.012 | -0.045 | **-0.031** |
| **B2PLYP-D3(Full)** | -0.061 | -0.071 | 0.019 | 0.013 | -0.124 | 0.055 | -0.034 | -0.075 | 0.047 | -0.046 | -0.085 | 0.170 | -0.254 | -0.018 | -0.097 | -0.074 | -0.042 | -0.055 | -0.039 | -0.068 | **-0.042** |
| **B2PLYPD3** | 0.002 | -0.007 | 0.053 | 0.053 | -0.084 | 0.173 | 0.025 | 0.002 | 0.146 | 0.015 | -0.007 | 0.267 | -0.204 | 0.038 | -0.047 | 0.005 | 0.012 | 0.008 | 0.024 | 0.006 | **0.024** |
| **B2PLYPD3(Full)** | -0.004 | -0.013 | 0.040 | 0.038 | -0.086 | 0.169 | 0.018 | -0.017 | 0.143 | 0.009 | -0.026 | 0.265 | -0.207 | 0.030 | -0.066 | -0.016 | -0.006 | 0.001 | 0.012 | -0.010 | **0.014** |
| **mPW2PLYP** | -0.126 | -0.130 | -0.097 | -0.097 | -0.183 | -0.086 | -0.124 | -0.134 | -0.095 | -0.133 | -0.143 | -0.013 | -0.291 | -0.118 | -0.159 | -0.134 | -0.122 | -0.136 | -0.117 | -0.136 | **-0.129** |
| **mPW2PLYP(Full)** | -0.134 | -0.138 | -0.102 | -0.103 | -0.186 | -0.089 | -0.129 | -0.143 | -0.099 | -0.139 | -0.152 | -0.016 | -0.292 | -0.122 | -0.170 | -0.154 | -0.127 | -0.143 | -0.124 | -0.153 | **-0.136** |
| **mPW2PLYPD** | -0.221 | -0.224 | -0.202 | -0.202 | -0.264 | -0.191 | -0.215 | -0.222 | -0.198 | -0.224 | -0.230 | -0.140 | -0.345 | -0.214 | -0.244 | -0.227 | -0.219 | -0.233 | -0.218 | -0.232 | **-0.223** |
| **mPW2PLYPD(Full)** | -0.226 | -0.229 | -0.204 | -0.206 | -0.265 | -0.193 | -0.220 | -0.227 | -0.201 | -0.229 | -0.236 | -0.142 | -0.347 | -0.218 | -0.251 | -0.241 | -0.222 | -0.239 | -0.223 | -0.242 | **-0.228** |
| **PBE0DH** | 0.080 | 0.073 | 0.161 | 0.161 | -0.014 | 0.149 | 0.101 | 0.075 | 0.145 | 0.097 | 0.068 | 0.208 | -0.120 | 0.129 | 0.039 | 0.112 | 0.116 | 0.133 | 0.146 | 0.130 | **0.099** |
| **PBE0DH(Full)** | 0.075 | 0.069 | 0.156 | 0.155 | -0.016 | 0.148 | 0.096 | 0.066 | 0.142 | 0.092 | 0.062 | 0.207 | -0.121 | 0.125 | 0.031 | 0.093 | 0.113 | 0.126 | 0.139 | 0.115 | **0.094** |
| **DSDPBEP86** | 0.004 | -0.007 | 0.037 | 0.035 | -0.067 | 0.052 | -0.005 | -0.037 | 0.041 | -0.020 | -0.049 | 0.179 | -0.152 | 0.025 | -0.051 | -0.028 | -0.004 | -0.026 | 0.008 | -0.029 | **-0.005** |
| **DSDPBEP86(Full)** | -0.008 | -0.018 | 0.029 | 0.025 | -0.070 | 0.050 | -0.019 | -0.053 | 0.030 | -0.031 | -0.065 | 0.163 | -0.155 | 0.017 | -0.064 | -0.072 | -0.012 | -0.041 | -0.007 | -0.056 | **-0.018** |
| **revDSDPBEP86** | 0.026 | 0.017 | 0.075 | 0.078 | -0.049 | 0.067 | 0.012 | -0.019 | 0.058 | 0.002 | -0.029 | 0.193 | -0.142 | 0.048 | -0.033 | -0.013 | 0.019 | -0.006 | 0.034 | -0.004 | **0.017** |
| **revDSDPBEP86(Full)** | 0.016 | 0.003 | 0.061 | 0.059 | -0.057 | 0.064 | 0.004 | -0.035 | 0.054 | -0.010 | -0.047 | 0.179 | -0.145 | 0.037 | -0.051 | -0.049 | 0.008 | -0.019 | 0.019 | -0.033 | **0.003** |
| **PBEQIDH** | 0.130 | 0.121 | 0.175 | 0.172 | 0.057 | 0.184 | 0.117 | 0.074 | 0.173 | 0.102 | 0.061 | 0.329 | -0.061 | 0.127 | 0.071 | 0.084 | 0.131 | 0.103 | 0.141 | 0.098 | **0.120** |
| **PBEQIDH(Full)** | 0.117 | 0.108 | 0.160 | 0.158 | 0.051 | 0.177 | 0.108 | 0.057 | 0.164 | 0.089 | 0.045 | 0.316 | -0.066 | 0.121 | 0.057 | 0.046 | 0.123 | 0.088 | 0.123 | 0.058 | **0.105** |
| **MP2** | 0.219 | 0.188 | 0.203 | 0.198 | 0.190 | 0.273 | 0.150 | 0.072 | 0.241 | 0.120 | 0.046 | 0.636 | 0.089 | 0.138 | 0.147 | 0.024 | 0.143 | 0.012 | 0.138 | 0.003 | **0.161** |
| **MP2(Full)** | 0.182 | 0.149 | 0.165 | 0.158 | 0.177 | 0.253 | 0.120 | 0.033 | 0.224 | 0.086 | 0.002 | 0.599 | 0.080 | 0.117 | 0.099 | -0.054 | 0.114 | -0.022 | 0.094 | -0.061 | **0.126** |
| **MP3** | 0.274 | 0.243 | 0.249 | 0.250 | 0.233 | 0.277 | 0.171 | 0.089 | 0.244 | 0.140 | 0.058 | 0.601 | 0.106 | 0.161 | 0.205 | 0.034 | 0.192 | 0.025 | 0.164 | 0.016 | **0.187** |
| **MP3(Full)** | 0.240 | 0.206 | 0.207 | 0.206 | 0.222 | 0.261 | 0.150 | 0.054 | 0.230 | 0.116 | 0.023 | 0.571 | 0.101 | 0.137 | 0.153 | -0.043 | 0.165 | 0.000 | 0.121 | -0.046 | **0.154** |
| **CISD** | 0.325 | 0.295 | 0.347 | 0.356 | 0.250 | 0.328 | 0.211 | 0.127 | 0.312 | 0.190 | 0.109 | 0.594 | 0.122 | 0.193 | 0.252 | 0.093 | 0.268 | 0.104 | 0.260 | 0.101 | **0.242** |
| **CISD(Full)** | 0.289 | 0.261 | 0.312 | 0.317 | 0.251 | 0.334 | 0.203 | 0.101 | 0.317 | 0.179 | 0.086 | 0.574 | 0.116 | 0.174 | 0.201 | 0.012 | 0.241 | 0.076 | 0.224 | 0.030 | **0.215** |
| **CCSD** | 0.282 | 0.253 | 0.272 | 0.270 | 0.229 | 0.292 | 0.179 | 0.096 | 0.271 | 0.151 | 0.073 | 0.599 | 0.116 | 0.163 | 0.216 | 0.049 | 0.209 | 0.047 | 0.190 | 0.033 | **0.200** |
| **CCSD(Full)** | 0.245 | 0.214 | 0.228 | 0.225 | 0.221 | 0.274 | 0.156 | 0.062 | 0.254 | 0.126 | 0.031 | 0.558 | 0.107 | 0.139 | 0.159 | -0.033 | 0.178 | 0.011 | 0.142 | -0.033 | **0.163** |
| **QCISD** | 0.280 | 0.251 | 0.272 | 0.270 | 0.228 | 0.293 | 0.180 | 0.096 | 0.266 | 0.151 | 0.074 | 0.601 | 0.114 | 0.160 | 0.214 | 0.047 | 0.206 | 0.045 | 0.189 | 0.032 | **0.198** |
| **QCISD(Full)** | 0.243 | 0.212 | 0.228 | 0.228 | 0.220 | 0.275 | 0.155 | 0.062 | 0.249 | 0.126 | 0.033 | 0.570 | 0.107 | 0.136 | 0.157 | -0.034 | 0.176 | 0.009 | 0.140 | -0.033 | **0.163** |
| **BD** | 0.249 | 0.209 | 0.207 | 0.215 | 0.215 | 0.280 | 0.159 | 0.060 | 0.241 | 0.127 | 0.037 | 0.585 | 0.109 | 0.129 | 0.165 | -0.029 | 0.160 | 0.009 | 0.097 | -0.022 | **0.160** |
| **BD(Full)** | 0.249 | 0.209 | 0.207 | 0.212 | 0.215 | 0.280 | 0.159 | 0.059 | 0.241 | 0.127 | 0.037 | 0.585 | 0.109 | 0.129 | 0.165 | -0.029 | 0.160 | 0.009 | 0.097 | -0.022 | **0.160** |
| **MP4** | 0.244 | 0.211 | 0.229 | 0.203 | 0.205 | 0.262 | 0.146 | 0.072 | 0.224 | 0.109 | 0.036 | 0.573 | 0.101 | 0.129 | 0.166 | 0.006 | 0.128 | 0.010 | 0.115 | -0.009 | **0.158** |
| **MP4(Full)** | 0.182 | 0.161 | 0.163 | 0.160 | 0.184 | 0.225 | 0.092 | 0.025 | 0.178 | 0.052 | -0.020 | 0.536 | 0.094 | 0.099 | 0.084 | -0.052 | 0.111 | -0.020 | 0.067 | -0.065 | **0.113** |
| **CCSD(T)** | 0.250 | 0.215 | 0.194 | 0.167 | 0.208 | 0.260 | 0.143 | 0.069 | 0.224 | 0.110 | 0.036 | 0.568 | 0.104 | 0.117 | 0.173 | 0.004 | 0.134 | -0.001 | 0.107 | -0.010 | **0.154** |
| **CCSD(T)(Full)** | 0.200 | 0.167 | 0.164 | 0.166 | 0.187 | 0.244 | 0.090 | 0.016 | 0.198 | 0.052 | -0.021 | 0.532 | 0.097 | 0.099 | 0.107 | -0.052 | 0.117 | -0.022 | 0.070 | -0.057 | **0.118** |

| | | | | | | | | | | | | | | | | | | | | | |
|---|---|---|---|---|---|---|---|---|---|---|---|---|---|---|---|---|---|---|---|---|---|
| **QCISD(T)** | 0.249 | 0.215 | 0.198 | 0.172 | 0.207 | 0.260 | 0.143 | 0.069 | 0.224 | 0.110 | 0.036 | 0.565 | 0.103 | 0.115 | 0.174 | 0.004 | 0.133 | -0.002 | 0.105 | -0.013 | **0.153** |
| **QCISD(T)(Full)** | 0.199 | 0.166 | 0.163 | 0.168 | 0.186 | 0.235 | 0.090 | 0.037 | 0.189 | 0.051 | -0.021 | 0.530 | 0.097 | 0.098 | 0.107 | -0.053 | 0.115 | -0.023 | 0.084 | -0.049 | **0.118** |
| **BD(T）** | 0.207 | 0.186 | 0.187 | 0.150 | 0.160 | 0.224 | 0.114 | 0.033 | 0.187 | 0.058 | 0.021 | 0.508 | 0.100 | 0.063 | 0.127 | 0.007 | 0.077 | -0.006 | 0.098 | -0.013 | **0.124** |
| **BD(T)(Full)** | 0.180 | 0.111 | 0.182 | 0.178 | 0.209 | 0.235 | 0.119 | 0.046 | 0.271 | 0.090 | 0.003 | 0.556 | 0.085 | 0.041 | 0.057 | -0.029 | 0.091 | -0.009 | 0.074 | -0.024 | **0.123** |
| **MP5** | 0.257 | 0.224 | 0.192 | 0.199 | 0.210 | 0.257 | 0.142 | 0.069 | 0.222 | 0.111 | 0.033 | 0.569 | 0.105 | 0.120 | 0.180 | 0.007 | 0.141 | 0.015 | | | **0.170** |
| **MP5(Full)** | 0.213 | 0.177 | 0.180 | 0.176 | 0.177 | 0.231 | -0.241 | 0.023 | 0.193 | 0.052 | -0.021 | 0.498 | 0.098 | 0.101 | 0.118 | -0.050 | 0.138 | -0.025 | | | **0.113** |
| **Ave** | **0.192** | **0.185** | **0.401** | **0.401** | **0.078** | **0.351** | **0.302** | **0.271** | **0.347** | **0.299** | **0.268** | **0.373** | **-0.056** | **0.333** | **0.149** | **0.343** | **0.277** | **0.392** | **0.391** | **0.415** | **0.286** |
| **PM7** | 0.228 | | | | | | | | | | | | | | | | | | | | |
| **PM6** | 0.270 | | | | | | | | | | | | | | | | | | | | |
| **Dreiding** | 0.000 | | | | | | | | | | | | | | | | | | | | |
| **UFF** | 0.094 | | | | | | | | | | | | | | | | | | | | |

Table S4. Imaginary frequencies ($cm^{-1}$) of 36 methods and 20 basis sets.

| Molecules | Method/basis set and imaginary frequency |
|---|---|
| $^{4}He_2$ (62)[a] | B3LYP/6-311++G(2d,2p) (4.23*i*, SGG);<br>B3LYP/6-311++G(2df,2pd) (7.30*i*, SGG);<br>B3LYP/6-311++G(3d2f,3p2d) (2.32*i*, SGG);<br>B3LYP/6-311++G(df,pd) (6.80*i*, SGG);<br>B3LYP/6-311++G** (2.32*i*, SGG);<br>B3LYP/aug-cc-pVDZ (8.80*i*, SGG);<br>B3LYP/cc-pVDZ (28.21*i*, SGG);<br>B3LYP/cc-pVTZ (11.45*i*, SGG);<br>B3LYP-D2/6-311++G(2d,2p) (4.78*i*, SGG);<br>B3LYP-D2/6-311++G(2df,2pd) (5.53*i*, SGG);<br>B3LYP-D2/6-311++G(3d2f,3p2d) (16.90*i*, SGG);<br>B3LYP-D2/6-311++G(3d,3p) (13.10*i*, SGG);<br>B3LYP-D2/6-311++G(3df,3pd) (13.40*i*, SGG);<br>B3LYP-D2/6-311++G(df,pd) (17.12*i*, SGG);<br>B3LYP-D2/6-311++G** (16.90*i*, SGG);<br>B3LYP-D2/aug-cc-pV5Z (14.93*i*, SGG);<br>B3LYP-D2/aug-cc-pVDZ (1.93*i*, SGG);<br>B3LYP-D2/aug-cc-pVQZ (17.73*i*, SGG);<br>B3LYP-D2/aug-cc-pVTZ (17.07*i*, SGG);<br>B3LYP-D2/cc-pV5Z (14.90*i*, SGG);<br>B3LYP-D2/cc-pVQZ (17.97*i*, SGG);<br>B3LYP-D2/Def2QZVP (14.64*i*, SGG);<br>B3LYP-D2/Def2QZVPP (14.64*i*, SGG);<br>B3LYP-D3/6-311++G(3d,3p) (15.58*i*, SGG);<br>B3LYP-D3/6-311++G(3df,3pd) (15.83*i*, SGG);<br>B3LYP-D3/aug-cc-pVDZ (8.65*i*, SGG);<br>B3LYP-D3BJ/6-311++G(2d,2p) (16.85*i*, SGG);<br>B3LYP-D3BJ/6-311++G(3df,3pd) (11.09*i*, SGG);<br>B3LYP-D3BJ/aug-cc-pVQZ (15.71*i*, SGG);<br>B3LYP-D3BJ/cc-pV5Z (19.68*i*, SGG);<br>B3LYP-D3BJ/Def2QZVP (20.73*i*, SGG);<br>B3LYP-D3BJ/Def2QZVPP (20.73*i*, SGG);<br>BD(T)/6-311++G(3d2f,3p2d) (20.25*i*, SGG);<br>BD(T)/6-311++G(3d,3p) (40.83*i*, SGG);<br>BD(T)/6-311++G(3df,3pd) (27.07*i*, SGG);<br>BD(T)/6-311++G** (20.25*i*, SGG);<br>BD(T)/aug-cc-pVDZ (23.51*i*, SGG);<br>BD(T)/Def2TZVP (23.72*i*, SGG); |

| | |
|---|---|
| | BD(T)(Full)/6-311++G(3d2f,3p2d) (20.25*i*, SGG);<br>BD(T)(Full)/6-311++G(3d,3p) (40.83*i*, SGG);<br>BD(T)(Full)/6-311++G(3df,3pd) (27.07*i*, SGG);<br>BD(T)(Full)/6-311++G** (20.25*i*, SGG);<br>BD(T)(Full)/aug-cc-pVDZ (23.51*i*, SGG);<br>BD(T)(Full)/Def2TZVP (23.73*i*, SGG);<br>HF/aug-cc-pVDZ (1.27*i*, SGG);<br>HF/Def2TZVP (1.10*i*, SGG);<br>M06-D3/cc-pVDZ (91.51*i*, SGG);<br>PBEB95/6-311++G(2d,2p) (112.50*i*, SGG);<br>PBEB95/6-311++G(2df,2pd) (112.54*i*, SGG);<br>PBEB95/6-311++G(3d2f,3p2d) (115.49*i*, SGG);<br>PBEB95/6-311++G(3d,3p) (112.85*i*, SGG);<br>PBEB95/6-311++G(3df,3pd) (112.89*i*, SGG);<br>PBEB95/6-311++G(df,pd) (115.53*i*, SGG);<br>PBEB95/6-311++G** (115.49*i*, SGG);<br>PBEB95/aug-cc-pVDZ (116.96*i*, SGG);<br>PW6B95D3/aug-cc-pV5Z (104.00*i*, SGG);<br>PW6B95D3/aug-cc-pVQZ (104.42*i*, SGG);<br>PW6B95D3/cc-pV5Z (103.49*i*, SGG);<br>PW6B95D3/cc-pVQZ (103.49*i*, SGG);<br>PW6B95D3/cc-pVTZ (104.37*i*, SGG);<br>PW6B95D3/Def2QZVP (103.58*i*, SGG);<br>PW6B95D3/Def2QZVPP (103.58*i*, SGG) |
| $Ne_2$ (27) | B3LYP/aug-cc-pV5Z (8.21*i*, SGG);<br>B3LYP/Def2QZVPP (1.05*i*, SGG);<br>BD(T)/aug-cc-pVQZ (7.62*i*, SGG);<br>BD(T)/cc-pV5Z (14.71*i*, SGG);<br>BD(T)/cc-pVDZ (14.57*i*, SGG);<br>BD(T)/Def2QZVP (16.30*i*, SGG);<br>BD(T)/Def2QZVPP (16.30*i*, SGG);<br>BD(T)(Full)/cc-pV5Z (13.70*i*, SGG);<br>BD(T)(Full)/Def2QZVP (14.58*i*, SGG);<br>BD(T)(Full)/Def2QZVPP (14.58*i*, SGG);<br>LC-PBEB95/Def2TZVP (58.41*i*, SGG);<br>LC-PBEB95/Def2TZVPP (58.41*i*, SGG);<br>M06/6-311G** (65.44*i*, SGG);<br>MN15/cc-pVQZ (40.52*i*, SGG);<br>PBEB95/6-311++G(3d2f,3p2d) (39.52*i*, SGG);<br>PBEB95/aug-cc-pV5Z (49.62*i*, SGG);<br>PBEB95/aug-cc-pVDZ (29.33*i*, SGG); |

| | |
|---|---|
| | PBEB95/aug-cc-pVQZ (61.42*i*, SGG);<br>PBEB95/cc-pVTZ (59.38*i*, SGG);<br>PW6B95D3/6-311++G(3d,3p) (31.77*i*, SGG);<br>PW6B95D3/6-311++G(3df,3pd) (31.79*i*, SGG);<br>PW6B95D3/cc-pVQZ (47.10*i*, SGG);<br>PW6B95D3/Def2TZVP (48.57*i*, SGG);<br>PW6B95D3/Def2TZVPP (48.57*i*, SGG);<br>wB97X/6-311G** (15.09*i*, SGG);<br>wB97X/Def2QZVP (51.78*i*, SGG);<br>wB97X/Def2QZVPP (51.78*i*, SGG) |
| $^{40}Ar_2$ (7) | B3LYP/6-311++G(2d,2p) (4.95*i*, SGG);<br>B3LYP/6-311++G(2df,2pd) (5.37*i*, SGG);<br>B3LYP/cc-pVDZ (5.85*i*, SGG);<br>BD(T)/6-311++G(3d2f,3p2d) (3.77*i*, SGG);<br>BD(T)/6-311G** (4.65*i*, SGG);<br>BD(T)/aug-cc-pVTZ (70.07*i*, SGG);<br>BD(T)(Full)/6-311++G(3d2f,3p2d) (11.44*i*, SGG) |
| $^{84}Kr_2$ (2) | B3LYP/6-311G** (3.06*i*, SGG);<br>B3LYP/cc-pVTZ (3.66*i*, SGG) |
| HeNe (19) | B3LYP/cc-pV5Z (11.86*i*, SG);<br>BD(T)/aug-cc-pV5Z (19.09*i*, SG);<br>BD(T)/aug-cc-pVQZ (15.45*i*, SG);<br>BD(T)/cc-pV5Z (25.41*i*, SG);<br>BD(T)/Def2QZVP (28.70*i*, SG);<br>BD(T)/Def2QZVPP (28.70*i*, SG);<br>BD(T)(Full)/aug-cc-pV5Z (11.09*i*, SG);<br>BD(T)(Full)/aug-cc-pVQZ (12.91*i*, SG);<br>BD(T)(Full)/cc-pV5Z (24.56*i*, SG);<br>BD(T)(Full)/Def2QZVP (27.44*i*, SG);<br>BD(T)(Full)/Def2QZVPP (27.44*i*, SG);<br>HF/aug-cc-pVTZ (1.77*i*, SG);<br>LC-PBEB95/cc-pVDZ (69.86*i*, SG);<br>LC-wPBE-D3/aug-cc-pVDZ (1.93*i*, SG);<br>M06-D3/aug-cc-pV5Z (21.82*i*, SG);<br>wB97X/6-311++G(2d,2p) (49.31*i*, SG);<br>wB97X/6-311++G(2df,2pd) (57.38*i*, SG);<br>wB97X/cc-pVDZ (43.15*i*, SG);<br>wB97X/cc-pVQZ (47.94*i*, SG) |
| HeAr (10) | B3LYP/6-311G** (12.66*i*, SG);<br>B3LYP/Def2TZVP (21.29*i*, SG);<br>BD(T)/6-311++G(3df,3pd) (41.91*i*, SG); |

| | |
|---|---|
| | BD(T)/aug-cc-pVTZ (41.41*i*, SG);<br>BD(T)(Full)/6-311++G(2d,2p) (4.99*i*, SG);<br>BD(T)(Full)/6-311++G(2df,2pd) (37.65*i*, SG);<br>BD(T)(Full)/6-311++G(3d2f,3p2d) (36.94*i*, SG);<br>BD(T)(Full)/6-311++G(3d,3p) (48.84*i*, SG);<br>M06/aug-cc-pVQZ (21.96*i*, SG);<br>M06-D3/aug-cc-pVQZ (5.61*i*, SG) |
| HeKr (14) | B3LYP-D2/6-311++G(df,pd) (6.97*i*, SG);<br>B3LYP-D2/6-311++G** (6.23*i*, SG);<br>BD(T)/6-311++G(2df,2pd) (35.13*i*, SG);<br>BD(T)(Full)/6-311G** (19.67*i*, SG);<br>BD(T)(Full)/aug-cc-pV5Z (8.45*i*, SG);<br>MN15L/6-311++G(3df,3pd) (89.83*i*, SG);<br>MN15L/Def2TZVPP (106.44*i*, SG);<br>PBEB95/6-311++G(2d,2p) (72.67*i*, SG);<br>PBEB95/6-311++G(2df,2pd) (72.75*i*, SG);<br>PBEB95/6-311++G(3d,3p) (72.89*i*, SG);<br>PBEB95/6-311++G(3df,3pd) (72.96*i*, SG);<br>PBEB95/aug-cc-pVDZ (78.07*i*, SG);<br>PW6B95D3/Def2TZVP (66.25*i*, SG);<br>PW6B95D3/Def2TZVPP (66.18*i*, SG) |
| $^{20}Ne^{40}Ar$ (10) | LC-PBEB95/Def2TZVP (61.94*i*, SG);<br>LC-PBEB95/Def2TZVPP (61.93*i*, SG);<br>MN15L/aug-cc-pVTZ (25.88*i*, SG);<br>MN15L/Def2TZVP (59.52*i*, SG);<br>MN15L/Def2TZVPP (59.51*i*, SG);<br>PBEB95/6-311++G(3d2f,3p2d) (68.56*i*, SG);<br>PBEB95/6-311++G(df,pd) (68.93*i*, SG);<br>PBEB95/6-311++G** (68.86*i*, SG);<br>PBEB95/aug-cc-pVDZ (61.14*i*, SG);<br>PBEB95/aug-cc-pVTZ (59.09*i*, SG) |
| $^{22}Ne^{36}Ar$ (10) | B3LYP/aug-cc-pV5Z (6.73*i*, SG);<br>LC-PBEB95/Def2TZVP (60.30*i*, SG);<br>LC-PBEB95/Def2TZVPP (60.29*i*, SG);<br>LC-wPBE-D3/aug-cc-pVQZ (6.61*i*, SG);<br>MN15L/aug-cc-pVTZ (23.67*i*, SG);<br>PBEB95/6-311++G(3d2f,3p2d) (67.46*i*, SG);<br>PBEB95/6-311++G(df,pd) (67.83*i*, SG);<br>PBEB95/6-311++G** (67.76*i*, SG);<br>PBEB95/aug-cc-pVDZ (58.92*i*, SG);<br>PBEB95/aug-cc-pVTZ (58.33*i*, SG) |

| | |
|---|---|
| $^{20}Ne^{84}Kr$ (1) | B3LYP/cc-pV5Z (3.68*i*, SG) |
| $^{40}Ar^{84}Kr$ (4) | B3LYP/Def2TZVP (1.52*i*, SG);<br>B3LYP/Def2TZVPP (1.42*i*, SG);<br>BD(T)/6-311++G(df,pd) (7.57*i*, SG);<br>BD(T)(Full)/6-311++G(3d2f,3p2d) (4.86*i*, SG) |

[a] The number in the bracket is the total number of method/basis sets with imaginary frequencies when calculating this molecule.

Table S5. MADs (Å) of 11-RG-Mols calculated by 54 methods and 60 basis sets.[a,b,c,d]

| | BS21 | BS22 | BS23 | BS24 | BS25 | BS26 | BS27 | BS28 | BS29 | BS30 | BS31 | BS32 | BS33 | BS34 | BS35 | BS36 | BS37 | BS38 | BS39 | BS40 | |
|---|---|---|---|---|---|---|---|---|---|---|---|---|---|---|---|---|---|---|---|---|---|
| | BS41 | BS42 | BS43 | BS44 | BS45 | BS46 | BS47 | BS48 | BS49 | BS50 | BS51 | BS52 | BS53 | BS54 | BS55 | BS56 | BS57 | BS58 | BS59 | BS60 | |
| | BS61 | BS62 | BS63 | BS64 | BS65 | BS66 | BS67 | BS68 | BS69 | BS70 | BS71 | BS72 | BS73 | BS74 | BS75 | BS76 | BS77 | BS78 | BS79 | BS80 | Ave |
| **MP2** | 0.178 | 0.153 | 0.715 | 0.366 | 0.153 | 0.153 | 0.153 | 0.715 | 1.064 | 0.469 | 1.192 | 0.971 | 0.675 | 0.641 | 0.310 | 0.134 | 0.981 | | | | |
| | 0.200 | 0.259 | 0.226 | 0.270 | 0.585 | 0.381 | 0.623 | 0.501 | 0.718 | 0.258 | 0.242 | 0.170 | 0.295 | 0.130 | 0.237 | 0.334 | 0.379 | 0.253 | 0.269 | 0.162 | |
| | 0.285 | 0.124 | 0.224 | 0.349 | 0.153 | 0.140 | 0.219 | 0.147 | 0.140 | 0.468 | 0.251 | 0.294 | 0.174 | 0.071 | 0.283 | 0.154 | 0.073 | 0.099 | 0.893 | 0.096 | **0.354** |
| **MP2(Full)** | 0.175 | 0.153 | 0.636 | 0.319 | 0.153 | 0.153 | 0.153 | 0.647 | 0.831 | 0.430 | 1.192 | 0.971 | 0.675 | 0.641 | 0.307 | 0.134 | 0.981 | | | | |
| | 0.188 | 0.230 | 0.228 | 0.267 | 0.565 | 0.369 | 0.599 | 0.490 | 0.692 | 0.266 | 0.247 | 0.161 | 0.279 | 0.122 | 0.226 | 0.320 | 0.364 | 0.237 | 0.275 | 0.159 | |
| | 0.274 | 0.122 | 0.207 | 0.339 | 0.149 | 0.159 | 0.211 | 0.138 | 0.207 | 0.159 | 0.254 | 0.285 | 0.150 | 0.074 | 0.221 | 0.133 | 0.088 | 0.147 | 0.892 | 0.092 | **0.336** |
| **DSDPBEP86** | 0.207 | 0.295 | 0.150 | 0.054 | 0.295 | 0.295 | 0.295 | 0.149 | 0.269 | 0.043 | 0.760 | 0.669 | 0.135 | 0.198 | 0.232 | 0.040 | 0.753 | | | | |
| | 0.140 | 0.045 | 0.420 | 0.215 | 0.180 | 0.177 | 0.152 | 0.254 | 0.231 | 0.267 | 0.298 | 0.081 | 0.177 | 0.099 | 0.129 | 0.054 | 0.121 | 0.038 | 0.273 | 0.083 | |
| | 0.173 | 0.100 | 0.037 | 0.118 | 0.053 | 0.114 | 0.124 | 0.047 | 0.162 | 0.222 | 0.337 | 0.122 | 0.082 | 0.063 | 0.121 | 0.089 | 0.075 | 0.101 | 0.271 | 0.001 | **0.187** |
| **DSDPBEP86(Full)** | 0.208 | 0.299 | 0.135 | 0.044 | 0.299 | 0.299 | 0.299 | 0.139 | 0.256 | 0.035 | 0.760 | 0.669 | 0.135 | 0.198 | 0.233 | 0.040 | 0.754 | | | | |
| | 0.137 | 0.037 | 0.423 | 0.217 | 0.179 | 0.175 | 0.146 | 0.253 | 0.226 | 0.270 | 0.301 | 0.085 | 0.172 | 0.103 | 0.132 | 0.050 | 0.118 | 0.035 | 0.276 | 0.092 | |
| | 0.179 | 0.110 | 0.032 | 0.111 | 0.052 | 0.151 | 0.130 | 0.066 | 0.190 | 0.217 | 0.339 | 0.121 | 0.079 | 0.075 | 0.121 | 0.087 | 0.090 | 0.134 | 0.272 | 0.001 | **0.189** |
| **revDSDPBEP86** | 0.205 | 0.287 | 0.161 | 0.072 | 0.287 | 0.287 | 0.287 | 0.161 | 0.285 | 0.050 | 0.769 | 0.678 | 0.142 | 0.210 | 0.232 | 0.058 | 0.757 | | | | |
| | 0.144 | 0.060 | 0.415 | 0.212 | 0.186 | 0.183 | 0.159 | 0.262 | 0.242 | 0.264 | 0.293 | 0.084 | 0.183 | 0.100 | 0.136 | 0.064 | 0.132 | 0.046 | 0.270 | 0.084 | |
| | 0.179 | 0.101 | 0.041 | 0.126 | 0.059 | 0.121 | 0.128 | 0.053 | 0.132 | 0.231 | 0.332 | 0.134 | 0.076 | 0.061 | 0.128 | 0.087 | 0.067 | 0.084 | 0.271 | 0.014 | **0.191** |
| **revDSDPBEP86(Full)** | 0.205 | 0.290 | 0.146 | 0.054 | 0.290 | 0.290 | 0.290 | 0.150 | 0.271 | 0.045 | 0.769 | 0.678 | 0.142 | 0.209 | 0.233 | 0.058 | 0.757 | | | | |
| | 0.142 | 0.056 | 0.418 | 0.214 | 0.184 | 0.181 | 0.156 | 0.259 | 0.236 | 0.267 | 0.296 | 0.086 | 0.180 | 0.102 | 0.135 | 0.061 | 0.129 | 0.043 | 0.273 | 0.088 | |
| | 0.178 | 0.105 | 0.037 | 0.122 | 0.062 | 0.137 | 0.130 | 0.058 | 0.175 | 0.227 | 0.334 | 0.132 | 0.071 | 0.066 | 0.128 | 0.084 | 0.076 | 0.105 | 0.271 | 0.014 | **0.191** |
| **B2PLYPD** | 0.307 | 0.373 | 0.127 | 0.103 | 0.373 | 0.373 | 0.373 | 0.126 | 0.147 | 0.108 | 0.754 | 0.647 | 0.164 | 0.285 | 0.317 | 0.147 | 0.790 | | | | |
| | 0.210 | 0.096 | 0.524 | 0.311 | 0.229 | 0.178 | 0.127 | 0.220 | 0.289 | 0.343 | 0.365 | 0.192 | 0.279 | 0.206 | 0.191 | 0.092 | 0.259 | 0.128 | 0.347 | 0.193 | |
| | 0.277 | 0.197 | 0.128 | 0.264 | 0.138 | 0.161 | 0.192 | 0.139 | 0.177 | 0.162 | 0.390 | 0.164 | 0.105 | 0.119 | 0.161 | 0.134 | 0.133 | 0.155 | 0.352 | 0.134 | **0.245** |
| **B2PLYPD(Full)** | 0.307 | 0.376 | 0.118 | 0.103 | 0.376 | 0.376 | 0.376 | 0.120 | 0.143 | 0.101 | 0.754 | 0.647 | 0.164 | 0.285 | 0.317 | 0.147 | 0.790 | | | | |
| | 0.209 | 0.094 | 0.525 | 0.312 | 0.228 | 0.178 | 0.127 | 0.221 | 0.296 | 0.344 | 0.366 | 0.194 | 0.277 | 0.207 | 0.193 | 0.093 | 0.262 | 0.129 | 0.348 | 0.195 | |
| | 0.277 | 0.200 | 0.129 | 0.256 | 0.142 | 0.169 | 0.203 | 0.140 | 0.196 | 0.166 | 0.391 | 0.163 | 0.112 | 0.130 | 0.161 | 0.143 | 0.143 | 0.170 | 0.353 | 0.132 | **0.247** |
| **B2PLYPD-D3** | 0.301 | 0.378 | 0.155 | 0.080 | 0.378 | 0.378 | 0.378 | 0.153 | 0.216 | 0.138 | 0.732 | 0.665 | 0.168 | 0.332 | 0.299 | 0.110 | 0.774 | | | | |
| | 0.189 | 0.094 | 0.534 | 0.300 | 0.247 | 0.202 | 0.147 | 0.287 | 0.347 | 0.335 | 0.363 | 0.161 | 0.262 | 0.176 | 0.237 | 0.089 | 0.242 | 0.083 | 0.340 | 0.163 | |
| | 0.262 | 0.167 | 0.083 | 0.230 | 0.154 | 0.180 | 0.188 | 0.155 | 0.175 | 0.233 | 0.391 | 0.170 | 0.136 | 0.083 | 0.155 | 0.118 | 0.095 | 0.132 | 0.059 | 0.035 | **0.239** |
| **B2PLYPD-D3(Full)** | 0.302 | 0.380 | 0.142 | 0.081 | 0.380 | 0.379 | 0.380 | 0.145 | 0.209 | 0.138 | 0.732 | 0.665 | 0.168 | 0.332 | 0.300 | 0.110 | 0.774 | | | | |
| | 0.187 | 0.096 | 0.535 | 0.301 | 0.251 | 0.201 | 0.145 | 0.257 | 0.305 | 0.337 | 0.364 | 0.163 | 0.263 | 0.179 | 0.228 | 0.092 | 0.234 | 0.084 | 0.342 | 0.166 | |
| | 0.264 | 0.170 | 0.083 | 0.236 | 0.157 | 0.180 | 0.188 | 0.163 | 0.197 | 0.196 | 0.392 | 0.169 | 0.148 | 0.091 | 0.155 | 0.126 | 0.118 | 0.152 | 0.060 | 0.036 | **0.239** |
| **B2PLYPD3** | 0.271 | 0.345 | 0.169 | 0.086 | 0.345 | 0.345 | 0.345 | 0.168 | 0.339 | 0.126 | 0.761 | 0.696 | 0.153 | 0.368 | 0.276 | 0.083 | 0.769 | | | | |
| | 0.177 | 0.176 | 0.518 | 0.271 | 0.242 | 0.194 | 0.139 | 0.270 | 0.335 | 0.313 | 0.342 | 0.134 | 0.253 | 0.152 | 0.222 | 0.064 | 0.226 | 0.061 | 0.318 | 0.135 | |
| | 0.250 | 0.144 | 0.056 | 0.258 | 0.147 | 0.162 | 0.226 | 0.092 | 0.154 | 0.261 | 0.374 | 0.195 | 0.092 | 0.054 | 0.191 | 0.063 | 0.054 | 0.092 | 0.397 | 0.000 | **0.236** |
| **B2PLYPD3(Full)** | 0.271 | 0.347 | 0.160 | 0.077 | 0.347 | 0.347 | 0.347 | 0.161 | 0.332 | 0.118 | 0.761 | 0.696 | 0.153 | 0.368 | 0.277 | 0.083 | 0.769 | | | | |
| | 0.175 | 0.151 | 0.519 | 0.272 | 0.242 | 0.193 | 0.136 | 0.266 | 0.331 | 0.314 | 0.344 | 0.136 | 0.256 | 0.155 | 0.225 | 0.062 | 0.224 | 0.058 | 0.320 | 0.139 | |
| | 0.244 | 0.148 | 0.056 | 0.223 | 0.157 | 0.158 | 0.228 | 0.105 | 0.164 | 0.259 | 0.375 | 0.195 | 0.103 | 0.061 | 0.191 | 0.073 | 0.063 | 0.107 | 0.396 | 0.000 | **0.236** |
| **MP3** | 0.174 | 0.143 | 0.728 | 0.409 | 0.143 | 0.143 | 0.143 | 0.729 | 1.049 | 0.487 | 1.196 | 0.954 | 0.687 | 0.627 | 0.325 | 0.167 | 0.985 | | | | |
| | 0.223 | 0.284 | 0.219 | 0.258 | 0.588 | 0.368 | 0.597 | 0.492 | 0.699 | 0.249 | 0.233 | 0.159 | 0.288 | 0.122 | 0.224 | 0.337 | 0.379 | 0.257 | 0.257 | 0.150 | |
| | 0.277 | 0.116 | 0.226 | 0.353 | 0.174 | 0.164 | 0.205 | 0.157 | 0.156 | 0.451 | 0.239 | 0.283 | 0.180 | 0.077 | 0.269 | 0.161 | 0.065 | 0.065 | 0.916 | 0.792 | **0.365** |
| **MP3(Full)** | 0.172 | 0.143 | 0.655 | 0.360 | 0.143 | 0.143 | 0.143 | 0.667 | 0.837 | 0.463 | 1.196 | 0.954 | 0.687 | 0.627 | 0.323 | 0.167 | 0.985 | | | | |
| | 0.211 | 0.259 | 0.220 | 0.254 | 0.572 | 0.358 | 0.575 | 0.482 | 0.676 | 0.253 | 0.235 | 0.151 | 0.272 | 0.115 | 0.211 | 0.324 | 0.366 | 0.242 | 0.262 | 0.158 | |
| | 0.277 | 0.124 | 0.211 | 0.344 | 0.162 | 0.187 | 0.208 | 0.148 | 0.183 | 0.438 | 0.242 | 0.274 | 0.159 | 0.071 | 0.201 | 0.143 | 0.063 | 0.095 | 0.902 | 0.087 | **0.340** |
| **CCSD** | 0.166 | 0.142 | 0.710 | 0.409 | 0.142 | 0.142 | 0.142 | 0.710 | 1.030 | 0.476 | 1.184 | 0.947 | 0.667 | 0.622 | 0.313 | 0.163 | 0.984 | | | | |
| | 0.230 | 0.292 | 0.220 | 0.252 | 0.581 | 0.354 | 0.594 | 0.492 | 0.699 | 0.244 | 0.236 | 0.155 | 0.298 | 0.130 | 0.237 | 0.342 | 0.400 | 0.269 | 0.251 | 0.141 | |
| | 0.282 | 0.118 | 0.243 | 0.380 | 0.180 | 0.166 | 0.209 | 0.168 | 0.163 | 0.453 | 0.240 | 0.288 | 0.191 | 0.083 | 0.281 | 0.178 | 0.069 | 0.073 | 0.901 | 0.107 | **0.353** |
| **CCSD(Full)** | 0.163 | 0.142 | 0.641 | 0.358 | 0.142 | 0.142 | 0.142 | 0.652 | 0.837 | 0.451 | 1.184 | 0.947 | 0.667 | 0.622 | 0.314 | 0.163 | 0.984 | | | | |
| | 0.219 | 0.264 | 0.221 | 0.248 | 0.558 | 0.349 | 0.568 | 0.484 | 0.674 | 0.244 | 0.236 | 0.136 | 0.277 | 0.117 | 0.218 | 0.329 | 0.384 | 0.254 | 0.257 | 0.141 | |
| | 0.277 | 0.121 | 0.227 | 0.365 | 0.170 | 0.188 | 0.207 | 0.163 | 0.189 | 0.442 | 0.244 | 0.279 | 0.168 | 0.075 | 0.219 | 0.159 | 0.076 | 0.092 | 0.900 | 0.103 | **0.340** |
| **QCISD** | 0.172 | 0.142 | 0.707 | 0.406 | 0.142 | 0.142 | 0.142 | 0.707 | 1.030 | 0.472 | 1.182 | 0.950 | 0.665 | 0.627 | 0.319 | 0.164 | 0.983 | | | | |
| | 0.232 | 0.293 | 0.220 | 0.256 | 0.579 | 0.360 | 0.596 | 0.494 | 0.701 | 0.246 | 0.237 | 0.159 | 0.299 | 0.130 | 0.237 | 0.343 | 0.401 | 0.270 | 0.253 | 0.141 | |
| | 0.283 | 0.118 | 0.244 | 0.381 | 0.179 | 0.166 | 0.210 | 0.166 | 0.163 | 0.455 | 0.241 | 0.288 | 0.190 | 0.082 | 0.282 | 0.172 | 0.068 | 0.072 | 0.887 | 0.104 | **0.353** |

| | | | | | | | | | | | | | | | | | | | | | |
|---|---|---|---|---|---|---|---|---|---|---|---|---|---|---|---|---|---|---|---|---|---|
| **QCISD(Full)** | 0.171 | 0.142 | 0.638 | 0.357 | 0.142 | 0.142 | 0.142 | 0.649 | 0.836 | 0.449 | 1.182 | 0.950 | 0.665 | 0.627 | 0.314 | 0.164 | 0.983 | | | | |
| | 0.220 | 0.264 | 0.222 | 0.254 | 0.556 | 0.352 | 0.571 | 0.485 | 0.677 | 0.252 | 0.237 | 0.147 | 0.279 | 0.119 | 0.220 | 0.329 | 0.383 | 0.254 | 0.261 | 0.144 | |
| | 0.277 | 0.121 | 0.228 | 0.365 | 0.168 | 0.188 | 0.207 | 0.158 | 0.190 | 0.442 | 0.245 | 0.279 | 0.168 | 0.075 | 0.219 | 0.153 | 0.076 | 0.094 | 0.900 | 0.101 | **0.341** |
| **BD** | 0.171 | 0.141 | 0.608 | 0.368 | 0.141 | 0.141 | 0.141 | 0.623 | 0.800 | 0.445 | 1.186 | 0.945 | 0.629 | 0.594 | 0.316 | 0.163 | 0.985 | | | | |
| | 0.217 | 0.279 | 0.222 | 0.260 | 0.526 | 0.355 | 0.540 | 0.485 | 0.666 | 0.259 | 0.235 | 0.148 | 0.280 | 0.116 | 0.210 | 0.299 | 0.355 | 0.241 | 0.269 | 0.151 | |
| | 0.279 | 0.120 | 0.221 | 0.330 | 0.171 | 0.185 | 0.206 | 0.158 | 0.184 | 0.432 | 0.243 | 0.214 | 0.163 | 0.050 | 0.198 | 0.150 | 0.059 | 0.088 | 0.940 | 0.049 | **0.332** |
| **BD(Full)** | 0.171 | 0.141 | 0.608 | 0.368 | 0.141 | 0.141 | 0.141 | 0.623 | 0.800 | 0.445 | 1.186 | 0.945 | 0.629 | 0.594 | 0.316 | 0.163 | 0.985 | | | | |
| | 0.217 | 0.279 | 0.222 | 0.260 | 0.526 | 0.355 | 0.540 | 0.485 | 0.666 | 0.259 | 0.235 | 0.148 | 0.280 | 0.116 | 0.210 | 0.299 | 0.355 | 0.241 | 0.269 | 0.151 | |
| | 0.279 | 0.120 | 0.221 | 0.330 | 0.171 | 0.185 | 0.205 | 0.158 | 0.184 | 0.432 | 0.243 | 0.214 | 0.163 | 0.050 | 0.198 | 0.150 | 0.059 | 0.088 | 0.940 | 0.050 | **0.332** |
| **MP4** | 0.175 | 0.153 | 0.672 | 0.392 | 0.153 | 0.153 | 0.153 | 0.670 | 1.058 | 0.483 | 1.132 | 0.955 | 0.551 | 0.598 | 0.309 | 0.124 | 0.981 | | | | |
| | 0.223 | 0.257 | 0.311 | 0.262 | 0.552 | 0.354 | 0.559 | 0.494 | 0.603 | 0.251 | 0.247 | 0.138 | 0.288 | 0.118 | 0.205 | 0.305 | 0.309 | 0.242 | 0.258 | 0.146 | |
| | 0.292 | 0.121 | 0.208 | 0.234 | 0.149 | 0.160 | 0.208 | 0.136 | 0.153 | 0.441 | 0.253 | 0.222 | 0.159 | 0.045 | 0.191 | 0.138 | 0.030 | 0.048 | 0.937 | 0.069 | **0.334** |
| **MP4(Full)** | 0.170 | 0.152 | 0.582 | 0.328 | 0.152 | 0.152 | 0.152 | 0.608 | 0.854 | 0.454 | 1.132 | 0.955 | 0.551 | 0.598 | 0.306 | 0.124 | 0.980 | | | | |
| | 0.195 | 0.218 | 0.313 | 0.255 | 0.524 | 0.344 | 0.534 | 0.480 | 0.607 | 0.255 | 0.251 | 0.147 | 0.280 | 0.129 | 0.210 | 0.268 | 0.278 | 0.206 | 0.264 | 0.145 | |
| | 0.276 | 0.122 | 0.198 | 0.248 | 0.149 | 0.155 | 0.203 | 0.148 | 0.143 | 0.428 | 0.256 | 0.207 | 0.154 | 0.026 | 0.188 | 0.130 | 0.041 | 0.084 | 0.995 | 0.514 | **0.330** |
| **CCSD(T)** | 0.174 | 0.151 | 0.621 | 0.380 | 0.151 | 0.151 | 0.151 | 0.676 | 1.171 | 0.500 | 1.136 | 0.948 | 0.621 | 0.589 | 0.310 | 0.122 | 0.982 | | | | |
| | 0.219 | 0.257 | 0.310 | 0.259 | 0.552 | 0.349 | 0.551 | 0.491 | 0.597 | 0.248 | 0.244 | 0.134 | 0.281 | 0.117 | 0.203 | 0.299 | 0.307 | 0.239 | 0.255 | 0.141 | |
| | 0.289 | 0.119 | 0.208 | 0.234 | 0.151 | 0.162 | 0.206 | 0.138 | 0.154 | 0.438 | 0.251 | 0.221 | 0.159 | 0.045 | 0.191 | 0.139 | 0.038 | 0.045 | 1.004 | 0.128 | **0.337** |
| **CCSD(T)(Full)** | 0.169 | 0.150 | 0.590 | 0.332 | 0.150 | 0.150 | 0.150 | 0.609 | 0.829 | 0.457 | 1.136 | 0.948 | 0.555 | 0.589 | 0.307 | 0.122 | 0.982 | | | | |
| | 0.208 | 0.217 | 0.312 | 0.252 | 0.554 | 0.339 | 0.529 | 0.477 | 0.602 | 0.251 | 0.247 | 0.142 | 0.278 | 0.127 | 0.207 | 0.284 | 0.289 | 0.224 | 0.260 | 0.142 | |
| | 0.275 | 0.123 | 0.188 | 0.238 | 0.150 | 0.156 | 0.203 | 0.149 | 0.145 | 0.425 | 0.254 | 0.206 | 0.161 | 0.026 | 0.188 | 0.132 | 0.041 | 0.075 | 0.989 | 0.437 | **0.329** |
| **QCISD(T)** | 0.174 | 0.151 | 0.622 | 0.386 | 0.151 | 0.151 | 0.151 | 0.658 | 1.133 | 0.489 | 1.135 | 0.948 | 0.555 | 0.591 | 0.310 | 0.122 | 0.982 | | | | |
| | 0.219 | 0.257 | 0.310 | 0.259 | 0.549 | 0.349 | 0.551 | 0.491 | 0.566 | 0.248 | 0.244 | 0.134 | 0.281 | 0.117 | 0.203 | 0.299 | 0.307 | 0.240 | 0.255 | 0.141 | |
| | 0.289 | 0.119 | 0.207 | 0.235 | 0.150 | 0.162 | 0.206 | 0.138 | 0.155 | 0.438 | 0.251 | 0.220 | 0.158 | 0.044 | 0.190 | 0.139 | 0.038 | 0.045 | 0.985 | 0.081 | **0.333** |
| **QCISD(T)(Full)** | 0.169 | 0.150 | 0.590 | 0.332 | 0.150 | 0.150 | 0.150 | 0.608 | 0.834 | 0.456 | 1.135 | 0.948 | 0.555 | 0.591 | 0.307 | 0.122 | 0.982 | | | | |
| | 0.208 | 0.217 | 0.312 | 0.252 | 0.553 | 0.339 | 0.528 | 0.477 | 0.654 | 0.252 | 0.248 | 0.142 | 0.278 | 0.127 | 0.208 | 0.284 | 0.290 | 0.224 | 0.261 | 0.142 | |
| | 0.275 | 0.123 | 0.178 | 0.197 | 0.150 | 0.156 | 0.203 | 0.150 | 0.145 | 0.425 | 0.254 | 0.205 | 0.154 | 0.026 | 0.188 | 0.132 | 0.041 | 0.075 | 1.084 | 0.060 | **0.324** |
| **BD(T)** | 0.117 | 0.063 | 0.654 | 0.309 | 0.092 | 0.077 | 0.077 | 0.633 | 0.742 | 0.418 | 1.153 | 0.956 | 0.627 | 0.625 | 0.257 | 0.132 | 0.973 | | | | |
| | 0.162 | 0.187 | 0.232 | 0.301 | 0.440 | 0.320 | 0.521 | 0.392 | 0.645 | 0.227 | 0.160 | 0.145 | 0.459 | 0.093 | 0.101 | 0.224 | 0.289 | 0.186 | 0.260 | 0.155 | |
| | 0.239 | 0.077 | 0.166 | 0.265 | 0.064 | 0.018 | 0.148 | 0.055 | 0.028 | 0.351 | 0.205 | 0.168 | 0.058 | 0.020 | 0.194 | 0.057 | 0.024 | 0.041 | 0.991 | 0.062 | **0.292** |
| **BD(T)(Full)** | 0.156 | 0.095 | 0.563 | 0.303 | 0.081 | 0.081 | 0.065 | 0.602 | 0.773 | 0.311 | 1.153 | 0.956 | 0.627 | 0.626 | 0.255 | 0.132 | 0.973 | | | | |
| | 0.162 | 0.232 | 0.181 | 0.310 | 0.527 | 0.368 | 0.529 | 0.548 | 0.602 | 0.268 | 0.221 | 0.278 | 0.398 | 0.222 | 0.274 | 0.254 | 0.291 | 0.207 | 0.269 | 0.189 | |
| | 0.294 | 0.109 | 0.236 | 0.370 | 0.122 | 0.133 | 0.213 | 0.088 | 0.158 | 0.482 | 0.215 | 0.197 | 0.117 | 0.278 | 0.182 | 0.142 | 0.017 | 0.484 | 0.981 | 0.084 | **0.333** |
| **APFD** | 0.309 | 0.375 | 0.043 | 0.076 | 0.375 | 0.375 | 0.375 | 0.044 | 0.026 | 0.066 | 0.677 | 0.561 | 0.080 | 0.043 | 0.331 | 0.095 | 0.740 | 0.174 | 0.402 | 0.367 | |
| | 0.208 | 0.028 | 0.536 | 0.337 | 0.141 | 0.116 | 0.034 | 0.058 | 0.017 | 0.354 | 0.372 | 0.149 | 0.113 | 0.158 | 0.108 | 0.030 | 0.011 | 0.049 | 0.357 | 0.153 | |
| | 0.116 | 0.152 | 0.050 | 0.011 | 0.077 | 0.158 | 0.110 | 0.069 | 0.173 | 0.056 | 0.407 | 0.088 | 0.079 | 0.083 | 0.091 | 0.083 | 0.085 | 0.102 | 0.019 | 0.023 | **0.182** |
| **wB97X** | 0.322 | 0.387 | 0.067 | 0.090 | 0.387 | 0.387 | 0.387 | 0.066 | 0.066 | 0.102 | 0.641 | 0.528 | 0.067 | 0.022 | 0.360 | 0.140 | 0.763 | 0.174 | 0.082 | 0.095 | |
| | 0.213 | 0.092 | 0.429 | 0.334 | 0.143 | 0.161 | 0.078 | 0.102 | 0.076 | 0.357 | 0.369 | 0.202 | 0.134 | 0.212 | 0.137 | 0.085 | 0.080 | 0.093 | 0.358 | 0.205 | |
| | 0.136 | 0.208 | 0.094 | 0.080 | 0.155 | 0.172 | 0.144 | 0.161 | 0.181 | 0.104 | 0.397 | 0.130 | 0.188 | 0.170 | 0.133 | 0.196 | 0.172 | 0.184 | 0.137 | 0.111 | **0.204** |
| **PW6B95D3** | 0.254 | 0.269 | 0.090 | 0.081 | 0.269 | 0.269 | 0.269 | 0.090 | 0.088 | 0.078 | 0.679 | 0.597 | 0.094 | 0.099 | 0.237 | 0.122 | 0.726 | 0.081 | 0.118 | 0.201 | |
| | 0.175 | 0.082 | 0.484 | 0.255 | 0.153 | 0.139 | 0.093 | 0.117 | 0.081 | 0.301 | 0.328 | 0.111 | 0.090 | 0.099 | 0.090 | 0.094 | 0.082 | 0.085 | 0.324 | 0.112 | |
| | 0.091 | 0.113 | 0.085 | 0.082 | 0.099 | 0.134 | 0.092 | 0.093 | 0.130 | 0.117 | 0.338 | 0.092 | 0.102 | 0.100 | 0.093 | 0.096 | 0.101 | 0.101 | 0.084 | 0.084 | **0.171** |
| **MN15** | 0.186 | 0.255 | 0.057 | 0.067 | 0.255 | 0.255 | 0.255 | 0.057 | 0.071 | 0.100 | 0.702 | 0.585 | 0.100 | 0.096 | 0.310 | 0.110 | 0.726 | 0.123 | 0.061 | 0.052 | |
| | 0.171 | 0.076 | 0.374 | 0.267 | 0.107 | 0.126 | 0.083 | 0.102 | 0.079 | 0.307 | 0.313 | 0.135 | 0.107 | 0.137 | 0.108 | 0.079 | 0.074 | 0.078 | 0.310 | 0.137 | |
| | 0.109 | 0.137 | 0.078 | 0.074 | 0.114 | 0.180 | 0.110 | 0.116 | 0.186 | 0.104 | 0.330 | 0.095 | 0.120 | 0.122 | 0.098 | 0.123 | 0.123 | 0.129 | 0.072 | 0.071 | **0.168** |
| **MN15L** | 0.122 | 0.157 | 0.091 | 0.082 | 0.157 | 0.157 | 0.157 | 0.091 | 0.084 | 0.129 | 0.665 | 0.554 | 0.114 | 0.067 | 0.266 | 0.121 | 0.715 | 0.118 | 0.074 | 0.054 | |
| | 0.142 | 0.075 | 0.288 | 0.173 | 0.121 | 0.135 | 0.088 | 0.096 | 0.087 | 0.284 | 0.286 | 0.130 | 0.104 | 0.132 | 0.106 | 0.076 | 0.075 | 0.076 | 0.286 | 0.132 | |
| | 0.105 | 0.133 | 0.076 | 0.076 | 0.107 | 0.189 | 0.107 | 0.108 | 0.193 | 0.116 | 0.272 | 0.113 | 0.151 | 0.150 | 0.115 | 0.152 | 0.151 | 0.139 | 0.080 | 0.080 | **0.158** |
| **M06** | 0.133 | 0.146 | 0.066 | 0.065 | 0.146 | 0.146 | 0.146 | 0.067 | 0.142 | 0.065 | 0.677 | 0.635 | 0.068 | 0.124 | 0.145 | 0.128 | 0.694 | 0.166 | 0.163 | 0.099 | |
| | 0.065 | 0.072 | 0.350 | 0.087 | 0.064 | 0.056 | 0.073 | 0.059 | 0.074 | 0.142 | 0.142 | 0.053 | 0.057 | 0.053 | 0.057 | 0.072 | 0.072 | 0.072 | 0.142 | 0.053 | |
| | 0.056 | 0.055 | 0.072 | 0.072 | 0.059 | 0.055 | 0.056 | 0.058 | 0.055 | 0.059 | 0.147 | 0.056 | 0.057 | 0.059 | 0.056 | 0.057 | 0.058 | 0.059 | 0.077 | 0.078 | **0.119** |
| **M06L** | 0.181 | 0.181 | 0.123 | 0.121 | 0.181 | 0.181 | 0.181 | 0.123 | 0.141 | 0.063 | 0.772 | 0.654 | 0.120 | 0.139 | 0.197 | 0.140 | 0.713 | 0.171 | 0.067 | 0.040 | |
| | 0.122 | 0.100 | 0.344 | 0.143 | 0.143 | 0.085 | 0.127 | 0.077 | 0.127 | 0.159 | 0.160 | 0.068 | 0.061 | 0.069 | 0.061 | 0.126 | 0.127 | 0.127 | 0.164 | 0.070 | |
| | 0.063 | 0.071 | 0.126 | 0.127 | 0.049 | 0.085 | 0.063 | 0.050 | 0.085 | 0.077 | 0.185 | 0.108 | 0.057 | 0.048 | 0.083 | 0.058 | 0.049 | 0.057 | 0.078 | 0.078 | **0.142** |
| **PBE1PBE** | 0.272 | 0.315 | 0.161 | 0.158 | 0.315 | 0.315 | 0.315 | 0.159 | 0.174 | 0.127 | 0.781 | 0.707 | 0.166 | 0.188 | 0.329 | 0.197 | 0.787 | 0.220 | 0.118 | 0.076 | |
| | 0.248 | 0.120 | 0.432 | 0.298 | 0.221 | 0.212 | 0.170 | 0.134 | 0.154 | 0.328 | 0.347 | 0.213 | 0.160 | 0.224 | 0.157 | 0.161 | 0.142 | 0.175 | 0.331 | 0.215 | |
| | 0.161 | 0.217 | 0.174 | 0.140 | 0.133 | 0.150 | 0.158 | 0.131 | 0.152 | 0.135 | 0.379 | 0.143 | 0.116 | 0.112 | 0.148 | 0.128 | 0.112 | 0.115 | 0.080 | 0.096 | **0.222** |

| | | | | | | | | | | | | | | | | | | | | | |
|---|---|---|---|---|---|---|---|---|---|---|---|---|---|---|---|---|---|---|---|---|---|
| **PBEB95** | 0.339 | 0.381 | 0.113 | 0.118 | 0.381 | 0.381 | 0.381 | 0.114 | 0.077 | 0.138 | 0.708 | 0.578 | 0.138 | 0.082 | 0.310 | 0.181 | 0.763 | 0.076 | 0.054 | 0.046 | |
| | 0.247 | 0.071 | 0.497 | 0.358 | 0.193 | 0.204 | 0.090 | 0.114 | 0.066 | 0.389 | 0.395 | 0.184 | 0.095 | 0.165 | 0.095 | 0.088 | 0.063 | 0.089 | 0.391 | 0.185 | |
| | 0.096 | 0.184 | 0.089 | 0.063 | 0.060 | 0.145 | 0.097 | 0.061 | 0.166 | 0.105 | 0.449 | 0.076 | 0.056 | 0.063 | 0.071 | 0.076 | 0.065 | 0.073 | 0.019 | 0.086 | **0.191** |
| **LGB95** | 0.283 | 0.318 | 0.083 | 0.100 | 0.318 | 0.318 | 0.318 | 0.097 | 0.092 | 0.106 | 0.684 | 0.554 | 0.096 | 0.072 | 0.294 | 0.112 | 0.753 | 0.116 | 0.122 | 0.070 | |
| | 0.230 | 0.065 | 0.373 | 0.300 | 0.153 | 0.142 | 0.081 | 0.116 | 0.080 | 0.334 | 0.359 | 0.119 | 0.093 | 0.120 | 0.093 | 0.073 | 0.081 | 0.066 | 0.336 | 0.119 | |
| | 0.094 | 0.119 | 0.066 | 0.081 | 0.096 | 0.157 | 0.095 | 0.098 | 0.160 | 0.116 | 0.411 | 0.102 | 0.100 | 0.098 | 0.104 | 0.102 | 0.099 | 0.088 | 0.085 | 0.085 | **0.177** |
| **LGKCIS** | 0.317 | 0.387 | 0.115 | 0.124 | 0.387 | 0.387 | 0.387 | 0.115 | 0.092 | 0.118 | 0.757 | 0.644 | 0.163 | 0.116 | 0.361 | 0.177 | 0.799 | 0.437 | 0.167 | 0.198 | |
| | 0.258 | 0.065 | 0.468 | 0.339 | 0.219 | 0.205 | 0.132 | 0.099 | 0.100 | 0.368 | 0.385 | 0.195 | 0.114 | 0.207 | 0.112 | 0.118 | 0.081 | 0.144 | 0.371 | 0.196 | |
| | 0.114 | 0.185 | 0.144 | 0.080 | 0.069 | 0.136 | 0.112 | 0.068 | 0.138 | 0.095 | 0.421 | 0.104 | 0.056 | 0.041 | 0.105 | 0.061 | 0.041 | 0.045 | 0.001 | 0.031 | **0.208** |
| **LC-PBEB95** | 0.227 | 0.275 | 0.081 | 0.081 | 0.275 | 0.275 | 0.275 | 0.081 | 0.098 | 0.106 | 0.689 | 0.577 | 0.072 | 0.083 | 0.355 | 0.140 | 0.761 | 0.210 | 0.066 | 0.069 | |
| | 0.197 | 0.086 | 0.410 | 0.324 | 0.156 | 0.141 | 0.086 | 0.095 | 0.085 | 0.353 | 0.368 | 0.144 | 0.099 | 0.137 | 0.099 | 0.087 | 0.086 | 0.087 | 0.354 | 0.146 | |
| | 0.100 | 0.138 | 0.087 | 0.086 | 0.097 | 0.140 | 0.099 | 0.095 | 0.140 | 0.095 | 0.426 | 0.121 | 0.100 | 0.107 | 0.120 | 0.099 | 0.096 | 0.110 | 0.088 | 0.087 | **0.179** |
| **LC-PKZBB95** | 0.224 | 0.272 | 0.081 | 0.081 | 0.272 | 0.272 | 0.272 | 0.081 | 0.097 | 0.106 | 0.688 | 0.576 | 0.071 | 0.083 | 0.350 | 0.139 | 0.760 | 0.192 | 0.066 | 0.068 | |
| | 0.176 | 0.085 | 0.387 | 0.320 | 0.155 | 0.140 | 0.086 | 0.096 | 0.084 | 0.349 | 0.363 | 0.143 | 0.100 | 0.136 | 0.101 | 0.086 | 0.085 | 0.101 | 0.351 | 0.144 | |
| | 0.101 | 0.137 | 0.086 | 0.085 | 0.098 | 0.148 | 0.099 | 0.095 | 0.139 | 0.104 | 0.403 | 0.130 | 0.109 | 0.107 | 0.120 | 0.098 | 0.096 | 0.109 | 0.088 | 0.087 | **0.178** |
| **LC-LGB95** | 0.249 | 0.253 | 0.080 | 0.081 | 0.253 | 0.253 | 0.253 | 0.080 | 0.098 | 0.107 | 0.689 | 0.577 | 0.072 | 0.083 | 0.354 | 0.140 | 0.761 | 0.190 | 0.068 | 0.105 | |
| | 0.198 | 0.086 | 0.392 | 0.324 | 0.156 | 0.140 | 0.086 | 0.096 | 0.085 | 0.361 | 0.369 | 0.144 | 0.100 | 0.136 | 0.109 | 0.087 | 0.086 | 0.087 | 0.363 | 0.145 | |
| | 0.102 | 0.137 | 0.087 | 0.086 | 0.106 | 0.125 | 0.099 | 0.095 | 0.115 | 0.105 | 0.426 | 0.123 | 0.110 | 0.108 | 0.120 | 0.099 | 0.098 | 0.110 | 0.087 | 0.087 | **0.179** |
| **LC-LGKCIS** | 0.344 | 0.417 | 0.091 | 0.085 | 0.417 | 0.417 | 0.417 | 0.091 | 0.215 | 0.134 | 0.770 | 0.703 | 0.153 | 0.218 | 0.406 | 0.223 | 0.772 | 0.173 | 0.080 | 0.204 | |
| | 0.240 | 0.159 | 0.513 | 0.368 | 0.204 | 0.251 | 0.143 | 0.195 | 0.211 | 0.378 | 0.400 | 0.225 | 0.205 | 0.238 | 0.187 | 0.152 | 0.219 | 0.168 | 0.381 | 0.227 | |
| | 0.207 | 0.221 | 0.167 | 0.218 | 0.137 | 0.130 | 0.180 | 0.132 | 0.127 | 0.188 | 0.432 | 0.193 | 0.184 | 0.146 | 0.193 | 0.180 | 0.102 | 0.126 | 0.146 | 0.101 | **0.248** |
| **B3LYP-D2** | 0.361 | 0.412 | 0.122 | 0.152 | 0.412 | 0.412 | 0.412 | 0.122 | 0.087 | 0.119 | 0.721 | 0.593 | 0.147 | 0.220 | 0.378 | 0.199 | 0.776 | 0.199 | 0.379 | 0.351 | |
| | 0.253 | 0.105 | 0.552 | 0.363 | 0.186 | 0.224 | 0.122 | 0.242 | 0.207 | 0.380 | 0.398 | 0.227 | 0.278 | 0.243 | 0.278 | 0.124 | 0.202 | 0.159 | 0.383 | 0.229 | |
| | 0.276 | 0.231 | 0.160 | 0.204 | 0.224 | 0.250 | 0.244 | 0.145 | 0.181 | 0.213 | 0.422 | 0.232 | 0.124 | 0.169 | 0.233 | 0.140 | 0.167 | 0.242 | 0.151 | 0.163 | **0.265** |
| **M06-D3** | 0.126 | 0.155 | 0.069 | 0.067 | 0.155 | 0.155 | 0.155 | 0.070 | 0.173 | 0.071 | 0.681 | 0.637 | 0.065 | 0.126 | 0.147 | 0.146 | 0.697 | 0.166 | 0.128 | 0.107 | |
| | 0.070 | 0.077 | 0.309 | 0.087 | 0.068 | 0.060 | 0.137 | 0.062 | 0.137 | 0.143 | 0.151 | 0.057 | 0.060 | 0.066 | 0.060 | 0.136 | 0.136 | 0.136 | 0.143 | 0.057 | |
| | 0.059 | 0.067 | 0.136 | 0.136 | 0.061 | 0.058 | 0.059 | 0.061 | 0.058 | 0.062 | 0.156 | 0.059 | 0.060 | 0.061 | 0.059 | 0.060 | 0.061 | 0.061 | 0.074 | 0.075 | **0.129** |
| **M062X-D3** | 0.329 | 0.371 | 0.137 | 0.129 | 0.371 | 0.371 | 0.371 | 0.136 | 0.153 | 0.143 | 0.777 | 0.722 | 0.166 | 0.186 | 0.359 | 0.208 | 0.722 | 0.186 | 0.134 | 0.099 | |
| | 0.247 | 0.133 | 0.539 | 0.342 | 0.225 | 0.154 | 0.148 | 0.098 | 0.145 | 0.345 | 0.355 | 0.152 | 0.102 | 0.155 | 0.101 | 0.147 | 0.143 | 0.150 | 0.347 | 0.155 | |
| | 0.105 | 0.151 | 0.150 | 0.143 | 0.079 | 0.138 | 0.103 | 0.082 | 0.150 | 0.098 | 0.376 | 0.123 | 0.088 | 0.086 | 0.122 | 0.094 | 0.090 | 0.101 | 0.040 | 0.036 | **0.210** |
| **B3LYP-D3** | 0.346 | 0.406 | 0.083 | 0.098 | 0.406 | 0.406 | 0.406 | 0.084 | 0.077 | 0.095 | 0.700 | 0.581 | 0.122 | 0.071 | 0.348 | 0.141 | 0.769 | 0.248 | 0.412 | 0.369 | |
| | 0.225 | 0.074 | 0.559 | 0.350 | 0.186 | 0.173 | 0.077 | 0.138 | 0.068 | 0.372 | 0.391 | 0.172 | 0.172 | 0.183 | 0.154 | 0.074 | 0.061 | 0.090 | 0.375 | 0.173 | |
| | 0.173 | 0.170 | 0.091 | 0.060 | 0.109 | 0.140 | 0.125 | 0.114 | 0.161 | 0.118 | 0.418 | 0.119 | 0.106 | 0.072 | 0.119 | 0.113 | 0.073 | 0.087 | 0.066 | 0.128 | **0.213** |
| **PBE1PBE-D3** | 0.314 | 0.372 | 0.108 | 0.115 | 0.372 | 0.372 | 0.372 | 0.108 | 0.097 | 0.114 | 0.696 | 0.586 | 0.143 | 0.101 | 0.342 | 0.136 | 0.755 | 0.189 | 0.348 | 0.295 | |
| | 0.226 | 0.092 | 0.524 | 0.337 | 0.200 | 0.161 | 0.113 | 0.094 | 0.093 | 0.352 | 0.367 | 0.166 | 0.106 | 0.175 | 0.110 | 0.106 | 0.084 | 0.117 | 0.355 | 0.168 | |
| | 0.108 | 0.169 | 0.118 | 0.085 | 0.105 | 0.145 | 0.111 | 0.107 | 0.158 | 0.100 | 0.393 | 0.125 | 0.116 | 0.099 | 0.124 | 0.121 | 0.102 | 0.113 | 0.119 | 0.134 | **0.209** |
| **LC-wPBE-D3** | 0.293 | 0.344 | 0.076 | 0.070 | 0.344 | 0.344 | 0.344 | 0.076 | 0.170 | 0.086 | 0.703 | 0.669 | 0.092 | 0.204 | 0.329 | 0.159 | 0.734 | 0.121 | 0.315 | 0.204 | |
| | 0.180 | 0.147 | 0.512 | 0.303 | 0.168 | 0.211 | 0.109 | 0.187 | 0.171 | 0.324 | 0.340 | 0.175 | 0.171 | 0.181 | 0.169 | 0.105 | 0.167 | 0.117 | 0.327 | 0.176 | |
| | 0.172 | 0.165 | 0.116 | 0.166 | 0.156 | 0.143 | 0.167 | 0.153 | 0.127 | 0.184 | 0.369 | 0.162 | 0.167 | 0.172 | 0.162 | 0.175 | 0.169 | 0.193 | 0.153 | 0.144 | **0.222** |
| **PBEPBE-D3BJ** | 0.331 | 0.390 | 0.134 | 0.144 | 0.390 | 0.390 | 0.390 | 0.134 | 0.095 | 0.136 | 0.713 | 0.578 | 0.169 | 0.095 | 0.370 | 0.168 | 0.769 | 0.164 | 0.294 | 0.287 | |
| | 0.251 | 0.103 | 0.550 | 0.352 | 0.219 | 0.166 | 0.120 | 0.086 | 0.091 | 0.372 | 0.388 | 0.187 | 0.100 | 0.199 | 0.106 | 0.115 | 0.085 | 0.128 | 0.375 | 0.188 | |
| | 0.101 | 0.189 | 0.129 | 0.085 | 0.111 | 0.157 | 0.112 | 0.114 | 0.162 | 0.093 | 0.415 | 0.117 | 0.106 | 0.107 | 0.121 | 0.110 | 0.109 | 0.123 | 0.108 | 0.154 | **0.217** |
| **TPSSTPSS-D3BJ** | 0.237 | 0.264 | 0.131 | 0.129 | 0.264 | 0.264 | 0.264 | 0.130 | 0.127 | 0.109 | 0.725 | 0.658 | 0.129 | 0.124 | 0.257 | 0.148 | 0.758 | 0.159 | 0.265 | 0.275 | |
| | 0.214 | 0.103 | 0.466 | 0.256 | 0.187 | 0.149 | 0.138 | 0.093 | 0.100 | 0.293 | 0.308 | 0.149 | 0.108 | 0.155 | 0.105 | 0.134 | 0.096 | 0.140 | 0.296 | 0.150 | |
| | 0.109 | 0.153 | 0.140 | 0.095 | 0.100 | 0.111 | 0.105 | 0.098 | 0.113 | 0.094 | 0.340 | 0.100 | 0.098 | 0.089 | 0.100 | 0.100 | 0.089 | 0.101 | 0.095 | 0.104 | **0.190** |
| **B3LYP-D3BJ** | 0.303 | 0.373 | 0.030 | 0.042 | 0.373 | 0.373 | 0.373 | 0.030 | 0.168 | 0.174 | 0.684 | 0.605 | 0.079 | 0.259 | 0.311 | 0.090 | 0.752 | 0.181 | 0.343 | 0.284 | |
| | 0.187 | 0.157 | 0.551 | 0.315 | 0.151 | 0.120 | 0.042 | 0.173 | 0.191 | 0.334 | 0.365 | 0.128 | 0.209 | 0.150 | 0.206 | 0.036 | 0.158 | 0.054 | 0.337 | 0.129 | |
| | 0.210 | 0.139 | 0.056 | 0.159 | 0.162 | 0.218 | 0.204 | 0.158 | 0.197 | 0.166 | 0.398 | 0.206 | 0.190 | 0.146 | 0.206 | 0.111 | 0.092 | 0.127 | 0.406 | 0.358 | **0.229** |
| **PBE1PBE-D3BJ** | 0.288 | 0.350 | 0.101 | 0.103 | 0.350 | 0.350 | 0.350 | 0.101 | 0.095 | 0.100 | 0.689 | 0.596 | 0.131 | 0.098 | 0.333 | 0.127 | 0.751 | 0.116 | 0.239 | 0.228 | |
| | 0.219 | 0.078 | 0.517 | 0.318 | 0.191 | 0.150 | 0.103 | 0.071 | 0.081 | 0.337 | 0.355 | 0.161 | 0.087 | 0.172 | 0.091 | 0.098 | 0.074 | 0.110 | 0.340 | 0.162 | |
| | 0.088 | 0.166 | 0.110 | 0.074 | 0.093 | 0.139 | 0.091 | 0.096 | 0.148 | 0.079 | 0.384 | 0.106 | 0.101 | 0.084 | 0.106 | 0.103 | 0.086 | 0.090 | 0.088 | 0.089 | **0.192** |
| **LC-wPBE-D3BJ** | 0.228 | 0.291 | 0.103 | 0.093 | 0.291 | 0.291 | 0.291 | 0.103 | 0.230 | 0.132 | 0.728 | 0.691 | 0.112 | 0.258 | 0.298 | 0.162 | 0.732 | 0.093 | 0.143 | 0.105 | |
| | 0.171 | 0.208 | 0.477 | 0.256 | 0.164 | 0.187 | 0.132 | 0.231 | 0.233 | 0.286 | 0.313 | 0.152 | 0.216 | 0.173 | 0.214 | 0.134 | 0.232 | 0.130 | 0.289 | 0.154 | |
| | 0.217 | 0.161 | 0.129 | 0.232 | 0.183 | 0.195 | 0.215 | 0.181 | 0.175 | 0.228 | 0.344 | 0.222 | 0.211 | 0.260 | 0.223 | 0.190 | 0.241 | 0.241 | 0.331 | 0.335 | **0.237** |
| **Ave** | **0.232** | **0.259** | **0.286** | **0.181** | **0.259** | **0.259** | **0.259** | **0.291** | **0.408** | **0.220** | **0.869** | **0.737** | **0.292** | **0.316** | **0.302** | **0.135** | **0.830** | **0.171** | **0.181** | **0.167** | |
| | **0.196** | **0.148** | **0.392** | **0.275** | **0.302** | **0.227** | **0.264** | **0.265** | **0.324** | **0.294** | **0.301** | **0.149** | **0.202** | **0.145** | **0.165** | **0.163** | **0.201** | **0.145** | **0.300** | **0.148** | |
| | **0.196** | **0.139** | **0.136** | **0.191** | **0.125** | **0.151** | **0.159** | **0.117** | **0.154** | **0.237** | **0.324** | **0.169** | **0.126** | **0.090** | **0.160** | **0.120** | **0.085** | **0.112** | **0.417** | **0.116** | **0.246** |

[a] The three rows after a method correspond to the results of this method calculated with the first 20 basis sets (ie. BS21 to BS40), the second 20 ones (i.e. BS41 to BS60), and the third 20 ones (i.e. BS61 to BS80) and Ave.

[b] Likewise, a blank means that this method/basis set does not calculate these 11-RG-Mols, so LanL2MB (or BS38), STO-3G (or BS39), and STO-6G (or BS40) cannot calculate these 11-RG-Mols when using *ab initio* and double-hybrid methods.

[c] For basis sets UGBS (or BS79) and UGBS1V++ (BS80), these DFT methods can calculate only three molecules (i.e., $He_2$, HeNe, and $Ne_2$), just as Dreiding in Table S2.

[d] As in Table S2, a green or blue value means, respectively, that this MAD is smaller than 0.020 Å or is between 0.020～0.100 Å, while a black one means that this value is larger than 0.100 Å in which the largest one is shown with red.

Table S6. MDs (Å) of 11-RG-Mols calculated by 54 methods and 60 basis sets.

| | BS21 | BS22 | BS23 | BS24 | BS25 | BS26 | BS27 | BS28 | BS29 | BS30 | BS31 | BS32 | BS33 | BS34 | BS35 | BS36 | BS37 | BS38 | BS39 | BS40 | |
|---|---|---|---|---|---|---|---|---|---|---|---|---|---|---|---|---|---|---|---|---|---|
| | BS41 | BS42 | BS43 | BS44 | BS45 | BS46 | BS47 | BS48 | BS49 | BS50 | BS51 | BS52 | BS53 | BS54 | BS55 | BS56 | BS57 | BS58 | BS59 | BS60 | |
| | BS61 | BS62 | BS63 | BS64 | BS65 | BS66 | BS67 | BS68 | BS69 | BS70 | BS71 | BS72 | BS73 | BS74 | BS75 | BS76 | BS77 | BS78 | BS79 | BS80 | Ave |
| **MP2** | 0.051 | -0.140 | 0.715 | 0.366 | -0.140 | -0.140 | -0.140 | 0.715 | 1.064 | 0.469 | -0.050 | -0.060 | 0.675 | 0.641 | 0.091 | 0.134 | -0.343 | | | | |
| | 0.067 | 0.259 | -0.192 | 0.149 | 0.523 | 0.381 | 0.623 | 0.501 | 0.718 | -0.070 | -0.132 | 0.147 | 0.272 | 0.107 | 0.215 | 0.334 | 0.379 | 0.253 | -0.081 | 0.114 | |
| | 0.237 | 0.076 | 0.224 | 0.349 | 0.114 | -0.015 | 0.171 | 0.090 | -0.042 | 0.468 | -0.154 | 0.267 | 0.125 | 0.057 | 0.240 | 0.085 | 0.012 | -0.044 | 0.893 | 0.093 | **0.206** |
| **MP2(Full)** | 0.046 | -0.147 | 0.636 | 0.319 | -0.147 | -0.147 | -0.147 | 0.647 | 0.831 | 0.430 | -0.050 | -0.060 | 0.675 | 0.641 | 0.086 | 0.134 | -0.343 | | | | |
| | 0.050 | 0.230 | -0.198 | 0.144 | 0.503 | 0.369 | 0.599 | 0.490 | 0.692 | -0.080 | -0.145 | 0.116 | 0.233 | 0.077 | 0.180 | 0.320 | 0.364 | 0.237 | -0.095 | 0.087 | |
| | 0.202 | 0.050 | 0.207 | 0.339 | 0.065 | -0.088 | 0.140 | 0.034 | -0.161 | 0.087 | -0.165 | 0.254 | 0.078 | 0.008 | 0.174 | 0.043 | -0.037 | -0.099 | 0.892 | 0.089 | **0.170** |
| **DSDPBEP86** | -0.194 | -0.295 | 0.150 | 0.054 | -0.295 | -0.295 | -0.295 | 0.149 | 0.269 | 0.017 | -0.498 | -0.368 | 0.106 | 0.198 | -0.226 | -0.024 | -0.615 | | | | |
| | -0.111 | 0.039 | -0.420 | -0.183 | 0.027 | 0.116 | 0.152 | 0.254 | 0.231 | -0.252 | -0.282 | -0.041 | 0.099 | -0.059 | 0.052 | 0.054 | 0.121 | 0.034 | -0.260 | -0.054 | |
| | 0.083 | -0.072 | 0.024 | 0.110 | -0.023 | -0.104 | 0.034 | -0.038 | -0.152 | 0.222 | -0.321 | 0.050 | -0.023 | -0.048 | 0.038 | -0.040 | -0.062 | -0.089 | 0.271 | 0.001 | **-0.049** |
| **DSDPBEP86(Full)** | -0.197 | -0.299 | 0.135 | 0.044 | -0.299 | -0.299 | -0.299 | 0.139 | 0.256 | 0.007 | -0.498 | -0.368 | 0.106 | 0.198 | -0.227 | -0.024 | -0.615 | | | | |
| | -0.116 | 0.027 | -0.423 | -0.186 | 0.025 | 0.114 | 0.146 | 0.253 | 0.226 | -0.257 | -0.288 | -0.049 | 0.084 | -0.066 | 0.044 | 0.050 | 0.118 | 0.031 | -0.265 | -0.067 | |
| | 0.068 | -0.085 | 0.018 | 0.101 | -0.041 | -0.144 | 0.019 | -0.059 | -0.183 | 0.217 | -0.326 | 0.043 | -0.039 | -0.064 | 0.032 | -0.058 | -0.079 | -0.125 | 0.272 | 0.001 | **-0.057** |
| **revDSDPBEP86** | -0.189 | -0.287 | 0.161 | 0.072 | -0.287 | -0.287 | -0.287 | 0.161 | 0.285 | 0.031 | -0.489 | -0.359 | 0.114 | 0.210 | -0.220 | -0.006 | -0.607 | | | | |
| | -0.100 | 0.057 | -0.415 | -0.178 | 0.037 | 0.122 | 0.159 | 0.262 | 0.242 | -0.246 | -0.274 | -0.032 | 0.112 | -0.048 | 0.064 | 0.064 | 0.132 | 0.044 | -0.253 | -0.044 | |
| | 0.097 | -0.061 | 0.037 | 0.126 | -0.001 | -0.070 | 0.046 | -0.017 | -0.116 | 0.231 | -0.314 | 0.069 | -0.002 | -0.025 | 0.051 | -0.024 | -0.043 | -0.071 | 0.271 | 0.014 | **-0.036** |
| **revDSDPBEP86(Full)** | -0.192 | -0.290 | 0.146 | 0.054 | -0.290 | -0.290 | -0.290 | 0.150 | 0.271 | 0.022 | -0.489 | -0.359 | 0.114 | 0.209 | -0.221 | -0.006 | -0.608 | | | | |
| | -0.105 | 0.048 | -0.418 | -0.181 | 0.033 | 0.120 | 0.156 | 0.259 | 0.236 | -0.251 | -0.280 | -0.038 | 0.101 | -0.054 | 0.056 | 0.061 | 0.129 | 0.041 | -0.259 | -0.051 | |
| | 0.086 | -0.069 | 0.034 | 0.122 | -0.022 | -0.123 | 0.038 | -0.036 | -0.165 | 0.227 | -0.318 | 0.063 | -0.019 | -0.049 | 0.046 | -0.042 | -0.064 | -0.096 | 0.271 | 0.014 | **-0.045** |
| **B2PLYPD** | -0.302 | -0.373 | 0.063 | -0.051 | -0.373 | -0.373 | -0.373 | 0.062 | 0.129 | -0.039 | -0.558 | -0.415 | 0.035 | 0.258 | -0.312 | -0.129 | -0.683 | | | | |
| | -0.187 | -0.038 | -0.524 | -0.287 | -0.035 | -0.045 | 0.054 | 0.161 | 0.227 | -0.332 | -0.354 | -0.146 | 0.076 | -0.160 | -0.023 | -0.036 | 0.141 | -0.072 | -0.337 | -0.157 | |
| | 0.062 | -0.161 | -0.080 | 0.140 | -0.070 | -0.117 | -0.034 | -0.080 | -0.158 | 0.090 | -0.379 | -0.037 | -0.090 | -0.111 | -0.052 | -0.122 | -0.128 | -0.155 | 0.286 | -0.134 | **-0.120** |
| **B2PLYPD(Full)** | -0.304 | -0.376 | 0.053 | -0.057 | -0.376 | -0.376 | -0.376 | 0.055 | 0.124 | -0.049 | -0.558 | -0.415 | 0.035 | 0.258 | -0.313 | -0.129 | -0.683 | | | | |
| | -0.191 | -0.044 | -0.525 | -0.288 | -0.036 | -0.048 | 0.051 | 0.158 | 0.231 | -0.334 | -0.356 | -0.151 | 0.065 | -0.164 | -0.028 | -0.039 | 0.140 | -0.075 | -0.340 | -0.161 | |
| | 0.055 | -0.166 | -0.083 | 0.129 | -0.080 | -0.150 | -0.030 | -0.093 | -0.181 | 0.085 | -0.381 | -0.042 | -0.098 | -0.124 | -0.056 | -0.131 | -0.141 | -0.170 | 0.286 | -0.132 | **-0.125** |
| **B2PLYPD-D3** | -0.299 | -0.378 | 0.128 | 0.010 | -0.378 | -0.378 | -0.378 | 0.126 | 0.216 | 0.032 | -0.552 | -0.376 | 0.077 | 0.332 | -0.297 | -0.104 | -0.685 | | | | |
| | -0.178 | 0.036 | -0.534 | -0.279 | -0.011 | 0.038 | 0.117 | 0.268 | 0.333 | -0.328 | -0.356 | -0.128 | 0.103 | -0.144 | 0.079 | 0.013 | 0.181 | -0.041 | -0.334 | -0.139 | |
| | 0.093 | -0.143 | -0.048 | 0.164 | -0.022 | -0.100 | 0.019 | -0.035 | -0.146 | 0.215 | -0.384 | 0.029 | -0.002 | -0.070 | 0.002 | -0.056 | -0.095 | -0.132 | 0.059 | -0.035 | **-0.086** |
| **B2PLYPD-D3(Full)** | -0.301 | -0.380 | 0.114 | -0.001 | -0.380 | -0.379 | -0.380 | 0.118 | 0.209 | 0.029 | -0.552 | -0.376 | 0.077 | 0.332 | -0.298 | -0.104 | -0.685 | | | | |
| | -0.180 | 0.031 | -0.535 | -0.282 | -0.008 | 0.036 | 0.113 | 0.238 | 0.290 | -0.331 | -0.359 | -0.133 | 0.097 | -0.148 | 0.063 | 0.010 | 0.169 | -0.044 | -0.338 | -0.144 | |
| | 0.087 | -0.148 | -0.051 | 0.166 | -0.036 | -0.135 | 0.011 | -0.048 | -0.167 | 0.177 | -0.387 | 0.024 | -0.019 | -0.081 | -0.001 | -0.066 | -0.118 | -0.152 | 0.060 | -0.036 | **-0.093** |
| **B2PLYPD3** | -0.260 | -0.345 | 0.169 | 0.060 | -0.345 | -0.345 | -0.345 | 0.168 | 0.339 | 0.077 | -0.510 | -0.344 | 0.100 | 0.368 | -0.264 | -0.044 | -0.644 | | | | |
| | -0.134 | 0.158 | -0.518 | -0.242 | 0.023 | 0.071 | 0.139 | 0.264 | 0.335 | -0.296 | -0.325 | -0.070 | 0.152 | -0.088 | 0.121 | 0.054 | 0.217 | 0.020 | -0.302 | -0.081 | |
| | 0.137 | -0.090 | 0.013 | 0.248 | 0.070 | -0.049 | 0.112 | -0.003 | -0.076 | 0.255 | -0.357 | 0.111 | 0.022 | -0.004 | 0.097 | -0.015 | -0.036 | -0.059 | 0.397 | 0.000 | **-0.033** |
| **B2PLYPD3(Full)** | -0.262 | -0.347 | 0.160 | 0.050 | -0.347 | -0.347 | -0.347 | 0.161 | 0.332 | 0.067 | -0.510 | -0.344 | 0.100 | 0.368 | -0.264 | -0.044 | -0.644 | | | | |
| | -0.137 | 0.130 | -0.519 | -0.243 | 0.022 | 0.070 | 0.136 | 0.259 | 0.331 | -0.299 | -0.329 | -0.076 | 0.146 | -0.094 | 0.115 | 0.052 | 0.214 | 0.016 | -0.306 | -0.088 | |
| | 0.121 | -0.097 | 0.011 | 0.213 | 0.054 | -0.083 | 0.105 | -0.021 | -0.129 | 0.253 | -0.359 | 0.107 | 0.001 | -0.017 | 0.094 | -0.042 | -0.047 | -0.077 | 0.396 | 0.000 | **-0.041** |
| **MP3** | 0.058 | -0.117 | 0.728 | 0.409 | -0.117 | -0.117 | -0.117 | 0.729 | 1.049 | 0.487 | -0.046 | -0.077 | 0.687 | 0.627 | 0.111 | 0.167 | -0.336 | | | | |
| | 0.119 | 0.284 | -0.180 | 0.163 | 0.534 | 0.368 | 0.597 | 0.492 | 0.699 | -0.053 | -0.114 | 0.149 | 0.278 | 0.112 | 0.214 | 0.337 | 0.379 | 0.257 | -0.064 | 0.118 | |
| | 0.244 | 0.083 | 0.226 | 0.353 | 0.136 | -0.004 | 0.172 | 0.105 | -0.028 | 0.451 | -0.136 | 0.273 | 0.147 | 0.077 | 0.242 | 0.108 | 0.036 | -0.016 | 0.916 | -0.646 | **0.203** |
| **MP3(Full)** | 0.055 | -0.123 | 0.655 | 0.360 | -0.123 | -0.123 | -0.123 | 0.667 | 0.837 | 0.463 | -0.046 | -0.077 | 0.687 | 0.627 | 0.107 | 0.167 | -0.336 | | | | |
| | 0.103 | 0.259 | -0.185 | 0.157 | 0.517 | 0.358 | 0.575 | 0.482 | 0.676 | -0.063 | -0.126 | 0.123 | 0.244 | 0.087 | 0.184 | 0.324 | 0.366 | 0.242 | -0.078 | 0.092 | |
| | 0.212 | 0.059 | 0.211 | 0.344 | 0.087 | -0.053 | 0.142 | 0.054 | -0.082 | 0.438 | -0.146 | 0.260 | 0.104 | 0.040 | 0.170 | 0.070 | -0.011 | -0.069 | 0.902 | 0.087 | **0.190** |
| **CCSD** | 0.047 | -0.115 | 0.710 | 0.409 | -0.115 | -0.115 | -0.115 | 0.710 | 1.030 | 0.476 | -0.057 | -0.084 | 0.667 | 0.622 | 0.093 | 0.163 | -0.339 | | | | |
| | 0.119 | 0.292 | -0.179 | 0.152 | 0.524 | 0.354 | 0.594 | 0.492 | 0.699 | -0.062 | -0.115 | 0.145 | 0.288 | 0.119 | 0.226 | 0.342 | 0.400 | 0.269 | -0.073 | 0.109 | |
| | 0.251 | 0.086 | 0.243 | 0.380 | 0.145 | 0.001 | 0.178 | 0.122 | -0.014 | 0.453 | -0.136 | 0.278 | 0.160 | 0.083 | 0.255 | 0.128 | 0.058 | 0.002 | 0.901 | 0.107 | **0.217** |
| **CCSD(Full)** | 0.043 | -0.122 | 0.641 | 0.358 | -0.122 | -0.122 | -0.122 | 0.652 | 0.837 | 0.451 | -0.057 | -0.084 | 0.667 | 0.622 | 0.092 | 0.163 | -0.339 | | | | |
| | 0.101 | 0.264 | -0.184 | 0.146 | 0.501 | 0.349 | 0.568 | 0.484 | 0.674 | -0.076 | -0.129 | 0.106 | 0.247 | 0.087 | 0.188 | 0.329 | 0.384 | 0.254 | -0.087 | 0.086 | |
| | 0.222 | 0.066 | 0.227 | 0.365 | 0.097 | -0.050 | 0.152 | 0.073 | -0.070 | 0.442 | -0.147 | 0.265 | 0.114 | 0.048 | 0.189 | 0.087 | 0.007 | -0.065 | 0.900 | 0.103 | **0.191** |
| **QCISD** | 0.052 | -0.116 | 0.707 | 0.406 | -0.116 | -0.116 | -0.116 | 0.707 | 1.030 | 0.472 | -0.060 | -0.081 | 0.665 | 0.627 | 0.096 | 0.164 | -0.340 | | | | |
| | 0.116 | 0.293 | -0.179 | 0.154 | 0.522 | 0.360 | 0.596 | 0.494 | 0.701 | -0.064 | -0.116 | 0.148 | 0.288 | 0.119 | 0.226 | 0.343 | 0.401 | 0.270 | -0.075 | 0.109 | |
| | 0.250 | 0.086 | 0.244 | 0.381 | 0.144 | 0.001 | 0.177 | 0.120 | -0.015 | 0.455 | -0.137 | 0.278 | 0.159 | 0.082 | 0.255 | 0.121 | 0.056 | 0.001 | 0.887 | 0.104 | **0.216** |

| | | | | | | | | | | | | | | | | | | | | | |
|---|---|---|---|---|---|---|---|---|---|---|---|---|---|---|---|---|---|---|---|---|---|
| **QCISD(Full)** | 0.051 | -0.122 | 0.638 | 0.357 | -0.122 | -0.122 | -0.122 | 0.649 | 0.836 | 0.449 | -0.060 | -0.081 | 0.665 | 0.627 | 0.089 | 0.164 | -0.341 | | | | |
| | 0.099 | 0.264 | -0.185 | 0.146 | 0.499 | 0.352 | 0.571 | 0.485 | 0.677 | -0.072 | -0.130 | 0.116 | 0.248 | 0.088 | 0.189 | 0.329 | 0.383 | 0.254 | -0.086 | 0.088 | |
| | 0.221 | 0.065 | 0.228 | 0.365 | 0.095 | -0.050 | 0.151 | 0.068 | -0.071 | 0.442 | -0.148 | 0.265 | 0.113 | 0.046 | 0.189 | 0.081 | 0.005 | -0.066 | 0.900 | 0.101 | **0.191** |
| **BD** | 0.052 | -0.121 | 0.608 | 0.368 | -0.121 | -0.121 | -0.121 | 0.623 | 0.800 | 0.445 | -0.055 | -0.086 | 0.629 | 0.594 | 0.095 | 0.163 | -0.338 | | | | |
| | 0.104 | 0.279 | -0.185 | 0.159 | 0.469 | 0.355 | 0.540 | 0.485 | 0.666 | -0.057 | -0.126 | 0.121 | 0.253 | 0.088 | 0.182 | 0.299 | 0.355 | 0.241 | -0.072 | 0.097 | |
| | 0.225 | 0.066 | 0.221 | 0.330 | 0.099 | -0.052 | 0.152 | 0.069 | -0.073 | 0.432 | -0.146 | 0.201 | 0.109 | 0.037 | 0.169 | 0.079 | -0.008 | -0.061 | 0.940 | 0.049 | **0.184** |
| **BD(Full)** | 0.052 | -0.121 | 0.608 | 0.368 | -0.121 | -0.121 | -0.121 | 0.623 | 0.800 | 0.445 | -0.055 | -0.086 | 0.629 | 0.594 | 0.095 | 0.163 | -0.338 | | | | |
| | 0.104 | 0.279 | -0.185 | 0.159 | 0.469 | 0.355 | 0.540 | 0.485 | 0.666 | -0.057 | -0.126 | 0.121 | 0.253 | 0.088 | 0.182 | 0.299 | 0.355 | 0.241 | -0.072 | 0.097 | |
| | 0.225 | 0.066 | 0.221 | 0.330 | 0.099 | -0.052 | 0.151 | 0.069 | -0.073 | 0.432 | -0.146 | 0.201 | 0.109 | 0.037 | 0.169 | 0.079 | -0.008 | -0.061 | 0.940 | 0.050 | **0.184** |
| **MP4** | 0.039 | -0.121 | 0.672 | 0.392 | -0.121 | -0.121 | -0.121 | 0.670 | 1.058 | 0.483 | -0.248 | -0.075 | 0.551 | 0.598 | 0.082 | 0.124 | -0.345 | | | | |
| | 0.090 | 0.257 | -0.264 | 0.137 | 0.491 | 0.354 | 0.559 | 0.494 | 0.603 | -0.070 | -0.120 | 0.135 | 0.285 | 0.113 | 0.202 | 0.305 | 0.309 | 0.242 | -0.086 | 0.099 | |
| | 0.245 | 0.072 | 0.206 | 0.232 | 0.110 | -0.019 | 0.161 | 0.074 | -0.045 | 0.441 | -0.154 | 0.206 | 0.118 | 0.045 | 0.156 | 0.075 | 0.016 | -0.024 | 0.937 | 0.048 | **0.185** |
| **MP4(Full)** | 0.033 | -0.129 | 0.582 | 0.328 | -0.129 | -0.129 | -0.129 | 0.608 | 0.854 | 0.454 | -0.248 | -0.075 | 0.551 | 0.598 | 0.078 | 0.124 | -0.345 | | | | |
| | 0.057 | 0.218 | -0.269 | 0.128 | 0.462 | 0.344 | 0.534 | 0.480 | 0.607 | -0.083 | -0.133 | 0.105 | 0.238 | 0.083 | 0.168 | 0.268 | 0.278 | 0.206 | -0.100 | 0.076 | |
| | 0.207 | 0.049 | 0.193 | 0.243 | 0.063 | -0.088 | 0.134 | 0.028 | -0.113 | 0.428 | -0.164 | 0.187 | 0.088 | 0.024 | 0.142 | 0.038 | -0.016 | -0.061 | 0.995 | -0.431 | **0.151** |
| **CCSD(T)** | 0.042 | -0.118 | 0.621 | 0.380 | -0.118 | -0.118 | -0.118 | 0.676 | 1.171 | 0.500 | -0.244 | -0.083 | 0.621 | 0.589 | 0.086 | 0.122 | -0.341 | | | | |
| | 0.093 | 0.257 | -0.263 | 0.140 | 0.493 | 0.349 | 0.551 | 0.491 | 0.597 | -0.068 | -0.117 | 0.131 | 0.278 | 0.111 | 0.201 | 0.299 | 0.307 | 0.239 | -0.083 | 0.095 | |
| | 0.243 | 0.070 | 0.206 | 0.232 | 0.109 | -0.021 | 0.160 | 0.076 | -0.044 | 0.438 | -0.151 | 0.205 | 0.118 | 0.045 | 0.158 | 0.078 | 0.025 | -0.023 | 1.004 | 0.108 | **0.190** |
| **CCSD(T)(Full)** | 0.036 | -0.126 | 0.590 | 0.332 | -0.126 | -0.126 | -0.126 | 0.609 | 0.829 | 0.457 | -0.244 | -0.083 | 0.555 | 0.589 | 0.082 | 0.122 | -0.342 | | | | |
| | 0.078 | 0.217 | -0.269 | 0.131 | 0.494 | 0.339 | 0.529 | 0.477 | 0.602 | -0.080 | -0.129 | 0.100 | 0.236 | 0.080 | 0.166 | 0.284 | 0.289 | 0.224 | -0.097 | 0.075 | |
| | 0.208 | 0.050 | 0.183 | 0.232 | 0.063 | -0.090 | 0.135 | 0.031 | -0.113 | 0.425 | -0.162 | 0.187 | 0.095 | 0.023 | 0.143 | 0.041 | -0.013 | -0.053 | 0.989 | -0.354 | **0.154** |
| **QCISD(T)** | 0.042 | -0.119 | 0.622 | 0.386 | -0.119 | -0.119 | -0.119 | 0.658 | 1.133 | 0.489 | -0.244 | -0.082 | 0.555 | 0.591 | 0.086 | 0.122 | -0.342 | | | | |
| | 0.093 | 0.257 | -0.264 | 0.140 | 0.490 | 0.349 | 0.551 | 0.491 | 0.566 | -0.068 | -0.117 | 0.131 | 0.278 | 0.111 | 0.200 | 0.299 | 0.307 | 0.240 | -0.084 | 0.094 | |
| | 0.243 | 0.070 | 0.206 | 0.233 | 0.108 | -0.021 | 0.160 | 0.075 | -0.044 | 0.438 | -0.152 | 0.205 | 0.117 | 0.044 | 0.157 | 0.077 | 0.024 | -0.023 | 0.985 | 0.069 | **0.185** |
| **QCISD(T)(Full)** | 0.036 | -0.126 | 0.590 | 0.332 | -0.127 | -0.127 | -0.127 | 0.608 | 0.834 | 0.456 | -0.244 | -0.082 | 0.555 | 0.591 | 0.082 | 0.122 | -0.342 | | | | |
| | 0.077 | 0.217 | -0.269 | 0.131 | 0.494 | 0.339 | 0.528 | 0.477 | 0.654 | -0.080 | -0.130 | 0.100 | 0.235 | 0.080 | 0.165 | 0.284 | 0.290 | 0.224 | -0.097 | 0.075 | |
| | 0.208 | 0.050 | 0.173 | 0.191 | 0.062 | -0.090 | 0.135 | 0.030 | -0.113 | 0.425 | -0.162 | 0.186 | 0.087 | 0.023 | 0.143 | 0.041 | -0.013 | -0.053 | 1.084 | 0.036 | **0.163** |
| **BD(T)** | 0.037 | -0.057 | 0.654 | 0.309 | -0.085 | -0.070 | -0.070 | 0.633 | 0.742 | 0.418 | -0.089 | -0.075 | 0.627 | 0.625 | 0.053 | 0.132 | -0.339 | | | | |
| | 0.052 | 0.187 | -0.187 | 0.185 | 0.347 | 0.320 | 0.521 | 0.392 | 0.645 | -0.050 | -0.068 | 0.145 | 0.459 | 0.090 | 0.101 | 0.224 | 0.289 | 0.186 | -0.024 | 0.155 | |
| | 0.221 | 0.055 | 0.166 | 0.265 | 0.053 | -0.010 | 0.148 | 0.043 | -0.006 | 0.351 | -0.125 | 0.168 | 0.058 | 0.020 | 0.194 | 0.034 | 0.024 | 0.000 | 0.991 | 0.062 | **0.178** |
| **BD(T)(Full)** | -0.030 | -0.089 | 0.563 | 0.303 | -0.075 | -0.075 | -0.057 | 0.602 | 0.773 | 0.311 | -0.089 | -0.075 | 0.627 | 0.626 | 0.048 | 0.132 | -0.339 | | | | |
| | 0.035 | 0.232 | -0.172 | 0.167 | 0.446 | 0.368 | 0.529 | 0.548 | 0.602 | -0.031 | -0.106 | 0.278 | 0.375 | 0.199 | 0.274 | 0.254 | 0.291 | 0.207 | -0.040 | 0.189 | |
| | 0.294 | 0.099 | 0.236 | 0.370 | 0.061 | -0.126 | 0.213 | -0.010 | -0.152 | 0.482 | -0.125 | 0.197 | 0.085 | 0.278 | 0.182 | 0.089 | 0.015 | 0.413 | 0.981 | 0.063 | **0.201** |
| **APFD** | -0.309 | -0.375 | -0.034 | -0.076 | -0.375 | -0.375 | -0.375 | -0.035 | 0.024 | -0.066 | -0.635 | -0.483 | -0.050 | 0.043 | -0.331 | -0.095 | -0.730 | -0.154 | -0.402 | -0.367 | |
| | -0.208 | -0.028 | -0.536 | -0.337 | -0.130 | -0.087 | -0.016 | 0.012 | 0.014 | -0.354 | -0.372 | -0.149 | -0.035 | -0.158 | -0.039 | -0.030 | 0.003 | -0.049 | -0.357 | -0.153 | |
| | -0.037 | -0.152 | -0.050 | 0.003 | -0.049 | -0.144 | -0.043 | -0.061 | -0.160 | 0.002 | -0.407 | -0.043 | -0.069 | -0.059 | -0.047 | -0.074 | -0.062 | -0.090 | 0.019 | 0.006 | **-0.162** |
| **wB97X** | -0.322 | -0.387 | -0.064 | -0.090 | -0.387 | -0.387 | -0.387 | -0.063 | 0.029 | -0.102 | -0.627 | -0.524 | -0.064 | -0.019 | -0.360 | -0.118 | -0.763 | 0.096 | 0.060 | 0.082 | |
| | -0.213 | -0.032 | -0.402 | -0.334 | -0.143 | -0.097 | -0.017 | -0.021 | 0.006 | -0.357 | -0.369 | -0.147 | -0.064 | -0.157 | -0.067 | -0.023 | -0.001 | -0.032 | -0.358 | -0.150 | |
| | -0.066 | -0.153 | -0.032 | -0.001 | -0.076 | -0.161 | -0.076 | -0.083 | -0.170 | -0.023 | -0.397 | -0.052 | -0.087 | -0.090 | -0.057 | -0.092 | -0.094 | -0.106 | 0.137 | 0.111 | **-0.149** |
| **PW6B95D3** | -0.254 | -0.269 | -0.002 | -0.015 | -0.269 | -0.269 | -0.269 | -0.002 | 0.033 | -0.010 | -0.609 | -0.462 | -0.016 | 0.055 | -0.237 | -0.027 | -0.709 | -0.081 | -0.117 | -0.201 | |
| | -0.175 | 0.023 | -0.484 | -0.255 | -0.132 | 0.003 | 0.001 | 0.059 | 0.027 | -0.301 | -0.316 | -0.041 | 0.016 | -0.018 | 0.015 | -0.001 | 0.025 | -0.011 | -0.324 | -0.042 | |
| | 0.015 | -0.043 | -0.011 | 0.025 | 0.029 | -0.049 | 0.013 | 0.021 | -0.059 | 0.059 | -0.338 | 0.014 | 0.027 | 0.029 | 0.012 | 0.019 | 0.027 | 0.029 | 0.049 | 0.049 | **-0.096** |
| **MN15** | -0.175 | -0.245 | -0.034 | -0.048 | -0.245 | -0.245 | -0.245 | -0.034 | -0.001 | -0.096 | -0.574 | -0.472 | -0.007 | 0.024 | -0.298 | -0.044 | -0.701 | 0.100 | 0.033 | 0.024 | |
| | -0.171 | -0.028 | -0.372 | -0.256 | -0.090 | -0.019 | -0.002 | 0.005 | 0.002 | -0.295 | -0.302 | -0.088 | -0.043 | -0.091 | -0.045 | -0.018 | -0.014 | -0.023 | -0.299 | -0.091 | |
| | -0.045 | -0.092 | -0.024 | -0.014 | -0.054 | -0.171 | -0.048 | -0.056 | -0.178 | 0.003 | -0.320 | -0.006 | -0.052 | -0.054 | -0.011 | -0.055 | -0.055 | -0.064 | 0.023 | 0.017 | **-0.113** |
| **MN15L** | -0.108 | -0.143 | 0.031 | 0.006 | -0.143 | -0.143 | -0.143 | 0.031 | 0.022 | -0.098 | -0.599 | -0.500 | -0.045 | 0.010 | -0.253 | -0.043 | -0.705 | 0.040 | 0.021 | 0.020 | |
| | -0.114 | 0.003 | -0.288 | -0.156 | -0.051 | -0.016 | 0.030 | 0.022 | 0.031 | -0.267 | -0.270 | -0.049 | -0.002 | -0.051 | -0.004 | 0.008 | 0.009 | 0.007 | -0.270 | -0.051 | |
| | -0.004 | -0.052 | 0.006 | 0.009 | -0.018 | -0.161 | -0.006 | -0.018 | -0.165 | 0.002 | -0.256 | -0.002 | -0.059 | -0.059 | -0.003 | -0.060 | -0.060 | -0.048 | 0.039 | 0.038 | **-0.085** |
| **M06** | -0.078 | -0.095 | 0.054 | 0.051 | -0.095 | -0.095 | -0.095 | 0.055 | 0.139 | 0.053 | -0.572 | -0.412 | 0.054 | 0.113 | -0.104 | 0.112 | -0.628 | -0.089 | -0.142 | -0.098 | |
| | 0.006 | 0.056 | -0.320 | -0.054 | 0.012 | 0.035 | 0.059 | 0.042 | 0.060 | -0.111 | -0.111 | 0.029 | 0.037 | 0.029 | 0.036 | 0.058 | 0.059 | 0.058 | -0.111 | 0.029 | |
| | 0.036 | 0.030 | 0.058 | 0.059 | 0.039 | 0.028 | 0.035 | 0.038 | 0.027 | 0.042 | -0.119 | 0.038 | 0.037 | 0.040 | 0.037 | 0.036 | 0.039 | 0.044 | 0.077 | 0.078 | **-0.021** |
| **M06L** | -0.121 | -0.128 | 0.109 | 0.105 | -0.128 | -0.128 | -0.128 | 0.109 | 0.133 | 0.050 | -0.479 | -0.389 | 0.101 | 0.122 | -0.100 | 0.100 | -0.629 | -0.034 | 0.024 | 0.005 | |
| | 0.021 | 0.076 | -0.314 | -0.110 | 0.053 | 0.042 | 0.106 | 0.055 | 0.105 | -0.132 | -0.133 | 0.000 | 0.012 | -0.001 | 0.012 | 0.104 | 0.104 | 0.104 | -0.137 | -0.003 | |
| | 0.009 | -0.002 | 0.103 | 0.104 | 0.022 | 0.042 | 0.008 | 0.020 | 0.041 | 0.054 | -0.158 | 0.023 | 0.013 | 0.023 | -0.005 | 0.011 | 0.022 | 0.017 | 0.078 | 0.078 | **-0.016** |
| **PBE1PBE** | -0.249 | -0.314 | 0.084 | 0.048 | -0.313 | -0.313 | -0.314 | 0.082 | 0.141 | 0.048 | -0.492 | -0.336 | 0.058 | 0.161 | -0.267 | 0.034 | -0.615 | 0.185 | -0.054 | 0.029 | |
| | -0.106 | 0.077 | -0.432 | -0.263 | -0.030 | 0.043 | 0.093 | 0.129 | 0.127 | -0.296 | -0.316 | -0.016 | 0.085 | -0.027 | 0.083 | 0.079 | 0.115 | 0.066 | -0.300 | -0.020 | |
| | 0.082 | -0.021 | 0.064 | 0.113 | 0.067 | -0.001 | 0.078 | 0.061 | -0.002 | 0.126 | -0.347 | 0.086 | 0.044 | 0.061 | 0.078 | 0.030 | 0.057 | 0.041 | -0.030 | -0.051 | **-0.046** |

| | | | | | | | | | | | | | | | | | | | | | |
|---|---|---|---|---|---|---|---|---|---|---|---|---|---|---|---|---|---|---|---|---|---|
| **PBEB95** | -0.339 | -0.381 | -0.101 | -0.107 | -0.381 | -0.381 | -0.381 | -0.102 | -0.017 | -0.134 | -0.646 | -0.483 | -0.082 | 0.031 | -0.310 | -0.169 | -0.763 | -0.052 | -0.027 | -0.032 | |
| | -0.247 | -0.049 | -0.497 | -0.358 | -0.193 | -0.095 | -0.037 | 0.027 | 0.016 | -0.389 | -0.395 | -0.184 | -0.061 | -0.165 | -0.062 | -0.080 | -0.026 | -0.081 | -0.391 | -0.185 | |
| | -0.062 | -0.184 | -0.081 | -0.026 | -0.041 | -0.128 | -0.064 | -0.043 | -0.150 | 0.017 | -0.449 | -0.031 | -0.023 | -0.053 | -0.040 | -0.043 | -0.055 | -0.063 | -0.019 | -0.086 | **-0.166** |
| **LGB95** | -0.283 | -0.318 | -0.033 | -0.052 | -0.318 | -0.318 | -0.318 | -0.047 | 0.034 | -0.059 | -0.670 | -0.509 | -0.051 | 0.022 | -0.294 | -0.099 | -0.735 | 0.066 | 0.095 | 0.045 | |
| | -0.230 | 0.000 | -0.373 | -0.300 | -0.116 | -0.006 | 0.020 | 0.058 | 0.022 | -0.334 | -0.359 | -0.048 | 0.016 | -0.049 | 0.015 | 0.007 | 0.020 | 0.000 | -0.336 | -0.049 | |
| | 0.015 | -0.049 | 0.000 | 0.020 | 0.022 | -0.123 | 0.012 | 0.019 | -0.127 | 0.057 | -0.411 | 0.021 | 0.021 | 0.020 | 0.018 | 0.018 | 0.017 | 0.005 | 0.052 | 0.050 | **-0.104** |
| **LGKCIS** | -0.311 | -0.387 | 0.024 | -0.017 | -0.387 | -0.387 | -0.387 | 0.024 | 0.085 | -0.004 | -0.556 | -0.402 | -0.013 | 0.116 | -0.320 | -0.072 | -0.661 | 0.437 | 0.167 | 0.198 | |
| | -0.179 | 0.047 | -0.468 | -0.326 | -0.080 | -0.015 | 0.060 | 0.099 | 0.100 | -0.360 | -0.377 | -0.082 | 0.051 | -0.093 | 0.048 | 0.042 | 0.080 | 0.015 | -0.363 | -0.086 | |
| | 0.047 | -0.075 | 0.015 | 0.080 | 0.033 | -0.031 | 0.045 | 0.025 | -0.037 | 0.095 | -0.413 | 0.063 | 0.011 | 0.019 | 0.060 | 0.004 | 0.013 | 0.001 | -0.001 | -0.031 | **-0.080** |
| **LC-PBEB95** | -0.210 | -0.275 | 0.010 | 0.008 | -0.275 | -0.275 | -0.275 | 0.009 | 0.032 | -0.063 | -0.619 | -0.499 | -0.024 | 0.020 | -0.338 | -0.030 | -0.742 | 0.148 | 0.023 | 0.043 | |
| | -0.166 | 0.017 | -0.410 | -0.307 | -0.123 | -0.027 | 0.017 | 0.037 | 0.019 | -0.336 | -0.368 | -0.035 | 0.028 | -0.044 | 0.028 | 0.016 | 0.018 | 0.015 | -0.338 | -0.037 | |
| | 0.027 | -0.045 | 0.015 | 0.018 | 0.031 | -0.033 | 0.028 | 0.032 | -0.033 | 0.036 | -0.426 | 0.008 | 0.004 | 0.020 | 0.008 | 0.005 | 0.031 | 0.018 | 0.055 | 0.055 | **-0.091** |
| **LC-PKZBB95** | -0.208 | -0.272 | 0.011 | 0.009 | -0.272 | -0.272 | -0.272 | 0.011 | 0.032 | -0.063 | -0.618 | -0.498 | -0.023 | 0.021 | -0.333 | -0.028 | -0.741 | 0.131 | 0.024 | 0.042 | |
| | -0.145 | 0.017 | -0.387 | -0.304 | -0.122 | -0.025 | 0.017 | 0.037 | 0.020 | -0.333 | -0.363 | -0.033 | 0.029 | -0.042 | 0.027 | 0.016 | 0.019 | 0.001 | -0.335 | -0.035 | |
| | 0.028 | -0.043 | 0.016 | 0.019 | 0.031 | -0.041 | 0.029 | 0.032 | -0.032 | 0.028 | -0.403 | 0.000 | 0.021 | 0.022 | 0.009 | 0.031 | 0.031 | 0.020 | 0.055 | 0.055 | **-0.089** |
| **LC-LGB95** | -0.232 | -0.253 | 0.010 | 0.008 | -0.253 | -0.253 | -0.253 | 0.009 | 0.032 | -0.063 | -0.619 | -0.499 | -0.025 | 0.020 | -0.338 | -0.030 | -0.743 | 0.128 | 0.025 | 0.079 | |
| | -0.167 | 0.017 | -0.392 | -0.307 | -0.123 | -0.026 | 0.017 | 0.036 | 0.019 | -0.344 | -0.369 | -0.035 | 0.027 | -0.044 | 0.018 | 0.016 | 0.018 | 0.015 | -0.346 | -0.036 | |
| | 0.026 | -0.045 | 0.015 | 0.018 | 0.022 | -0.068 | 0.027 | 0.031 | -0.059 | 0.027 | -0.426 | 0.007 | -0.005 | -0.005 | 0.008 | 0.004 | 0.005 | 0.018 | 0.055 | 0.055 | **-0.092** |
| **LC-LGKCIS** | -0.344 | -0.417 | 0.065 | 0.031 | -0.417 | -0.417 | -0.417 | 0.066 | 0.215 | 0.044 | -0.513 | -0.340 | 0.088 | 0.218 | -0.354 | 0.004 | -0.710 | 0.173 | 0.080 | 0.204 | |
| | -0.145 | 0.159 | -0.513 | -0.352 | -0.060 | 0.023 | 0.139 | 0.179 | 0.211 | -0.366 | -0.388 | -0.060 | 0.125 | -0.074 | 0.106 | 0.148 | 0.219 | 0.131 | -0.369 | -0.064 | |
| | 0.121 | -0.058 | 0.131 | 0.218 | 0.098 | 0.023 | 0.095 | 0.091 | 0.015 | 0.171 | -0.419 | 0.141 | 0.154 | 0.129 | 0.137 | 0.147 | 0.084 | 0.096 | 0.146 | 0.101 | **-0.035** |
| **B3LYP-D2** | -0.361 | -0.412 | -0.104 | -0.152 | -0.412 | -0.412 | -0.412 | -0.105 | -0.063 | -0.117 | -0.685 | -0.557 | -0.121 | 0.081 | -0.378 | -0.192 | -0.762 | -0.170 | -0.379 | -0.351 | |
| | -0.253 | -0.102 | -0.552 | -0.363 | -0.170 | -0.182 | -0.100 | 0.036 | 0.049 | -0.380 | -0.398 | -0.216 | 0.028 | -0.233 | 0.026 | -0.116 | 0.039 | -0.151 | -0.383 | -0.219 | |
| | 0.022 | -0.221 | -0.153 | 0.038 | -0.014 | -0.071 | -0.014 | -0.096 | -0.161 | -0.001 | -0.422 | -0.011 | -0.119 | -0.110 | -0.014 | -0.136 | -0.126 | -0.076 | -0.151 | -0.163 | **-0.195** |
| **M06-D3** | -0.069 | -0.102 | 0.057 | 0.054 | -0.102 | -0.102 | -0.102 | 0.058 | 0.173 | 0.060 | -0.567 | -0.408 | 0.051 | 0.116 | -0.102 | 0.129 | -0.625 | -0.084 | -0.105 | -0.107 | |
| | 0.012 | 0.062 | -0.276 | -0.052 | 0.018 | 0.037 | 0.125 | 0.046 | 0.126 | -0.109 | -0.118 | 0.031 | 0.040 | 0.023 | 0.040 | 0.124 | 0.125 | 0.124 | -0.110 | 0.031 | |
| | 0.039 | 0.024 | 0.123 | 0.125 | 0.041 | 0.031 | 0.038 | 0.040 | 0.031 | 0.045 | -0.125 | 0.041 | 0.040 | 0.042 | 0.040 | 0.038 | 0.041 | 0.046 | 0.074 | 0.075 | **-0.010** |
| **M062X-D3** | -0.329 | -0.371 | 0.082 | 0.064 | -0.371 | -0.371 | -0.371 | 0.081 | 0.137 | 0.041 | -0.501 | -0.320 | 0.076 | 0.172 | -0.278 | 0.060 | -0.722 | -0.186 | -0.117 | -0.071 | |
| | -0.068 | 0.075 | -0.539 | -0.300 | -0.020 | -0.036 | 0.089 | 0.023 | 0.098 | -0.320 | -0.330 | -0.092 | -0.030 | -0.096 | -0.032 | 0.086 | 0.095 | 0.083 | -0.323 | -0.096 | |
| | -0.033 | -0.091 | 0.082 | 0.095 | -0.010 | -0.083 | -0.037 | -0.015 | -0.094 | 0.020 | -0.351 | 0.005 | -0.017 | -0.013 | 0.001 | -0.022 | -0.018 | -0.031 | -0.004 | -0.013 | **-0.094** |
| **B3LYP-D3** | -0.346 | -0.406 | -0.046 | -0.081 | -0.406 | -0.406 | -0.406 | -0.047 | 0.029 | -0.076 | -0.639 | -0.465 | -0.077 | 0.071 | -0.346 | -0.119 | -0.736 | -0.160 | -0.412 | -0.369 | |
| | -0.221 | -0.028 | -0.559 | -0.350 | -0.151 | -0.109 | -0.034 | 0.051 | 0.024 | -0.372 | -0.391 | -0.153 | 0.019 | -0.164 | -0.002 | -0.047 | 0.013 | -0.063 | -0.375 | -0.155 | |
| | 0.017 | -0.151 | -0.065 | 0.013 | -0.035 | -0.098 | -0.035 | -0.047 | -0.120 | 0.023 | -0.418 | -0.026 | -0.033 | -0.051 | -0.032 | -0.044 | -0.055 | -0.067 | -0.066 | -0.128 | **-0.165** |
| **PBE1PBE-D3** | -0.314 | -0.372 | -0.046 | -0.076 | -0.372 | -0.372 | -0.372 | -0.046 | 0.005 | -0.079 | -0.603 | -0.457 | -0.066 | 0.011 | -0.332 | -0.090 | -0.707 | -0.112 | -0.348 | -0.295 | |
| | -0.202 | -0.056 | -0.524 | -0.331 | -0.138 | -0.083 | -0.032 | -0.017 | -0.013 | -0.352 | -0.367 | -0.145 | -0.069 | -0.153 | -0.073 | -0.044 | -0.023 | -0.054 | -0.355 | -0.147 | |
| | -0.070 | -0.148 | -0.057 | -0.025 | -0.083 | -0.128 | -0.078 | -0.088 | -0.138 | -0.023 | -0.393 | -0.062 | -0.084 | -0.082 | -0.066 | -0.090 | -0.086 | -0.097 | -0.119 | -0.134 | **-0.171** |
| **LC-wPBE-D3** | -0.293 | -0.344 | 0.057 | 0.031 | -0.344 | -0.344 | -0.344 | 0.057 | 0.170 | 0.041 | -0.572 | -0.375 | 0.033 | 0.204 | -0.318 | 0.000 | -0.727 | -0.069 | -0.315 | -0.204 | |
| | -0.147 | 0.147 | -0.512 | -0.294 | -0.077 | 0.021 | 0.094 | 0.161 | 0.171 | -0.319 | -0.335 | -0.063 | 0.092 | -0.069 | 0.090 | 0.090 | 0.167 | 0.077 | -0.322 | -0.065 | |
| | 0.091 | -0.054 | 0.077 | 0.166 | 0.101 | 0.036 | 0.086 | 0.096 | 0.009 | 0.158 | -0.363 | 0.115 | 0.115 | 0.143 | 0.112 | 0.111 | 0.138 | 0.151 | 0.153 | 0.144 | **-0.053** |
| **PBEPBE-D3BJ** | -0.331 | -0.390 | -0.070 | -0.101 | -0.390 | -0.390 | -0.390 | -0.071 | -0.019 | -0.091 | -0.622 | -0.467 | -0.096 | 0.000 | -0.354 | -0.123 | -0.708 | -0.070 | -0.294 | -0.287 | |
| | -0.224 | -0.063 | -0.550 | -0.350 | -0.155 | -0.106 | -0.048 | -0.026 | -0.019 | -0.372 | -0.388 | -0.157 | -0.070 | -0.169 | -0.076 | -0.061 | -0.031 | -0.074 | -0.375 | -0.160 | |
| | -0.074 | -0.161 | -0.075 | -0.031 | -0.085 | -0.117 | -0.084 | -0.090 | -0.123 | -0.033 | -0.415 | -0.070 | -0.091 | -0.091 | -0.075 | -0.097 | -0.096 | -0.111 | -0.108 | -0.154 | **-0.182** |
| **TPSSTPSS-D3BJ** | -0.214 | -0.264 | 0.057 | 0.041 | -0.264 | -0.264 | -0.264 | 0.056 | 0.089 | 0.043 | -0.540 | -0.385 | 0.020 | 0.124 | -0.219 | 0.033 | -0.624 | -0.011 | -0.265 | -0.275 | |
| | -0.110 | 0.061 | -0.466 | -0.227 | -0.065 | 0.025 | 0.058 | 0.088 | 0.095 | -0.267 | -0.281 | -0.016 | 0.055 | -0.022 | 0.052 | 0.051 | 0.088 | 0.044 | -0.270 | -0.019 | |
| | 0.052 | -0.022 | 0.043 | 0.088 | 0.058 | 0.015 | 0.048 | 0.055 | 0.014 | 0.084 | -0.313 | 0.060 | 0.040 | 0.062 | 0.057 | 0.036 | 0.059 | 0.039 | -0.021 | -0.033 | **-0.064** |
| **B3LYP-D3BJ** | -0.303 | -0.373 | 0.017 | -0.014 | -0.373 | -0.373 | -0.373 | 0.016 | 0.168 | 0.104 | -0.617 | -0.441 | -0.024 | 0.259 | -0.302 | -0.059 | -0.709 | -0.027 | -0.294 | -0.233 | |
| | -0.171 | 0.144 | -0.551 | -0.313 | -0.106 | -0.049 | 0.026 | 0.151 | 0.191 | -0.334 | -0.365 | -0.104 | 0.113 | -0.126 | 0.111 | 0.015 | 0.158 | -0.005 | -0.337 | -0.107 | |
| | 0.111 | -0.117 | -0.007 | 0.156 | 0.095 | 0.051 | 0.105 | 0.089 | 0.013 | 0.144 | -0.398 | 0.115 | 0.125 | 0.101 | 0.112 | 0.044 | 0.044 | 0.061 | 0.406 | 0.358 | **-0.067** |
| **PBE1PBE-D3BJ** | -0.288 | -0.350 | -0.026 | -0.049 | -0.350 | -0.350 | -0.350 | -0.027 | 0.024 | -0.050 | -0.590 | -0.448 | -0.047 | 0.031 | -0.313 | -0.067 | -0.688 | -0.031 | -0.239 | -0.228 | |
| | -0.177 | -0.026 | -0.517 | -0.310 | -0.118 | -0.063 | -0.010 | 0.016 | 0.011 | -0.332 | -0.350 | -0.118 | -0.028 | -0.129 | -0.034 | -0.020 | 0.004 | -0.032 | -0.335 | -0.121 | |
| | -0.032 | -0.125 | -0.033 | 0.003 | -0.053 | -0.087 | -0.041 | -0.057 | -0.093 | 0.009 | -0.379 | -0.036 | -0.057 | -0.053 | -0.044 | -0.063 | -0.056 | -0.073 | -0.062 | -0.089 | **-0.142** |
| **LC-wPBE-D3BJ** | -0.228 | -0.291 | 0.100 | 0.087 | -0.291 | -0.291 | -0.291 | 0.100 | 0.230 | 0.116 | -0.538 | -0.352 | 0.077 | 0.258 | -0.263 | 0.058 | -0.662 | 0.085 | -0.135 | -0.055 | |
| | -0.084 | 0.208 | -0.477 | -0.233 | -0.026 | 0.066 | 0.132 | 0.217 | 0.233 | -0.265 | -0.292 | 0.006 | 0.171 | -0.015 | 0.169 | 0.128 | 0.232 | 0.122 | -0.268 | 0.003 | |
| | 0.169 | -0.005 | 0.121 | 0.232 | 0.173 | 0.113 | 0.166 | 0.170 | 0.089 | 0.214 | -0.323 | 0.179 | 0.188 | 0.248 | 0.177 | 0.165 | 0.228 | 0.217 | 0.331 | 0.335 | **0.016** |
| **Ave** | **-0.155** | **-0.249** | **0.241** | **0.118** | **-0.249** | **-0.249** | **-0.248** | **0.246** | **0.385** | **0.144** | **-0.420** | **-0.308** | **0.220** | **0.297** | **-0.158** | **0.020** | **-0.571** | **0.010** | **-0.119** | **-0.092** | |
| | **-0.070** | **0.107** | **-0.377** | **-0.126** | **0.121** | **0.115** | **0.224** | **0.234** | **0.299** | **-0.224** | **-0.256** | **-0.008** | **0.122** | **-0.025** | **0.082** | **0.117** | **0.170** | **0.084** | **-0.231** | **-0.021** | |
| | **0.102** | **-0.036** | **0.074** | **0.158** | **0.035** | **-0.060** | **0.064** | **0.016** | **-0.081** | **0.201** | **-0.285** | **0.094** | **0.034** | **0.012** | **0.075** | **0.013** | **-0.011** | **-0.027** | **0.385** | **0.003** | **0.004** |

## Reference

[S1] Frisch, M.J.; Trucks, G.W.; Schlegel, H.B.; Scuseria, G.E.; Robb, M.A.; Cheeseman, J.R.; Scalmani, G.; Barone, V.; Mennucci, B.; Petersson, G.A.; Nakatsuji, H.; Caricato, M.; Li, X.; Hratchian, H.P.; Izmaylov, A.F.; Bloino, J.; Zheng, G.; Sonnenberg, J.L.; Hada, M.; Ehara, M.; Toyota, K.; Fukuda, R.; Hasegawa, J.; Ishida, M.; Nakajima, T.; Honda, Y.; Kitao, O.; Nakai, H.; Vreven, T.; Montgomery, J.A., Jr.; Peralta, J.E.; Ogliaro, F.; Bearpark, M.; Heyd, J.J.; Brothers, E.; Kudin, K.N.; Staroverov, V.N.; Kobayashi, R.; Normand, J.; Raghavachari, K.; Rendell, A.; Burant, J.C.; Iyengar, S.S.; Tomasi, J.; Cossi, M.; Rega, N.; Millam, J.M.; Klene, M.; Knox, J.E.; Cross, J.B.; Bakken, V.; Adamo, C.; Jaramillo, J.; Gomperts, R.; Stratmann, R.E.; Yazyev, O.; Austin, A.J.; Cammi, R.; Pomelli, C.; Ochterski, J.W.; Martin, R.L.; Morokuma, K.; Zakrzewski, V.G.; Voth, G.A.; Salvador, P.; Dannenberg, J.J.; Dapprich, S.; Daniels, A.D.; Farkas, O.; Foresman, J.B.; Ortiz, J.V.; Cioslowski, J.; Fox, D.J. *Gaussian 09*, Revision A.01; Gaussian, Inc.: Wallingford CT, USA, 2009.
